# Cobalt-catalysed asymmetric hydrovinylation of 1,3-dienes†
†Electronic supplementary information (ESI) available. See DOI: 10.1039/c5sc00929d


**DOI:** 10.1039/c5sc00929d

**Published:** 2015-04-23

**Authors:** Yam N. Timsina, Rakesh K. Sharma, T. V. RajanBabu

**Affiliations:** a Department of Chemistry and Biochemistry , The Ohio State University , 100 West 18th Avenue , Columbus , Ohio 43210 , USA . Email: rajanbabu.1@osu.edu ; Fax: +1 614 292 1685 ; Tel: +1 614 688 3543

## Abstract

In the presence of bidentate 1,*n*-bis-diphenylphosphinoalkane-CoCl_2_ complexes {Cl_2_Co[P ∼ P]} and Me_3_Al or methylaluminoxane, acyclic (*E*)-1,3-dienes react with ethylene (1 atmosphere) to give excellent yields of hydrovinylation products. The regioselectivity (1,4- or 1,2-addition) and the alkene configuration (*E*- or *Z*-) of the resulting product depend on the nature of the ligand and temperature at which the reaction is carried out. Cobalt(ii)-complexes of 1,1-diphenylphosphinomethane and similar ligands with narrow bite angles give mostly 1,2-addition, retaining the *E*-geometry of the original diene. Complexes of most other ligands at low temperature (–40 °C) give almost exclusively a single branched product, (*Z*)-3-alkylhexa-1,4-diene, which arises from a 1,4-hydrovinylation reaction. A minor product is the linear adduct, a 6-alkyl-hexa-1,4-diene, also arising from a 1,4-addition of ethylene. As the temperature is increased, a higher proportion of the major branched-1,4-adduct appears as the (*E*)-isomer. The unexpectedly high selectivity seen in the Co-catalysed reaction as compared to the corresponding Ni-catalysed reaction can be rationalized by invoking the intermediacy of an η^4^-[(diene)[P ∼ P]CoH]^+^-complex and its subsequent reactions. The enhanced reactivity of terminal *E*-1,3-dienes over the corresponding *Z*-dienes can also be explained on the basis of the ease of formation of this η^4^-complex in the former case. The lack of reactivity of the X_2_Co(dppb) (X = Cl, Br) complexes in the presence of Zn/ZnI_2_ makes the Me_3_Al-mediated reaction different from the previously reported hydroalkenylation of dienes. Electron-rich phospholanes, bis-oxazolines and *N*-heterocyclic carbenes appear to be poor ligands for the Co(ii)-catalysed hydrovinylation of 1,3-dienes. An extensive survey of chiral ligands reveals that complexes of DIOP, BDPP and Josiphos ligands are quite effective for these reactions even at –45 °C and enantioselectivities in the range of 90–99% ee can be realized for a variety of 1,3-dienes. Cobalt(ii)-complex of an electron-deficient Josiphos ligand is especially active, requiring only <1 mol% catalyst to effect the reactions.

## Abstract 2

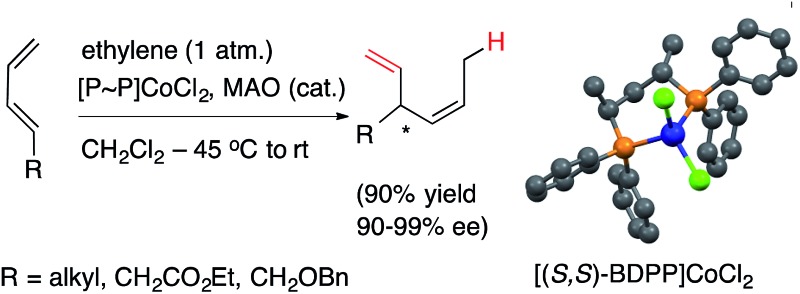
Excellent selectivity with complexes of DIOP, BDPP and Josiphos with *E*-1,3-dienes reacting faster than the *Z*-isomers at low temperatures.

## Introduction

**Fig. 1:**
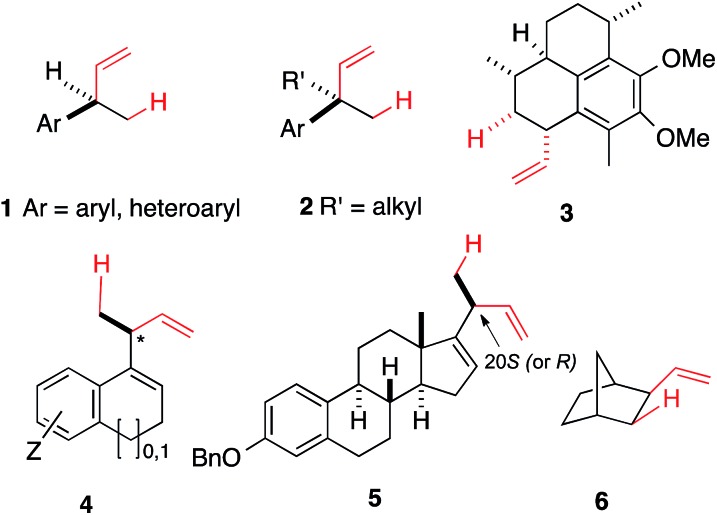
Examples of products of Ni(ii)-catalysed asymmetric hydrovinylation.

Heterodimerization reactions involving ethylene and other alkenes catalysed by late transition metals including iron,^1^–^3^ cobalt,^4^–^8^ nickel,^9^–^13^ rhodium,^14^ palladium^15^ and ruthenium14a,^16^ had a long history^17^ even before the more recent discoveries of applications of related chemistry in olefin polymerization reactions emerged.^18^–^21^ Among these, palladium,^15^ nickel and cobalt, and to a limited extent, ruthenium catalysed reactions^22^–^29^ have attracted the most attention in the stereoselective synthesis of small molecules. Through optimizations of ligands, promoters and reaction conditions, the Ni-catalysed asymmetric hydrovinylations (eqn (1)) of vinylarenes,^30^–^36^ selected dienes,^37^–^39^ and strained alkenes^40^,^41^ have been accomplished with excellent regio- and stereoselectivities giving highly valuable intermediates. Prototypical examples of the products of these reactions (**1–6**) are shown in Fig. 1. In a related reaction, Ho *et al.* reported^42^ dimerization of vinylarenes and 1-alkenes catalysed by *N*-heterocyclic carbene complexes of Ni(ii)-salts to give uncommon tail-to-tail dimerization products (**7**, eqn (2)).
1

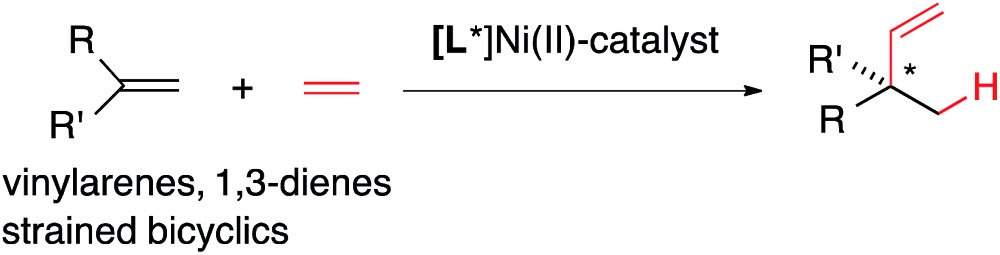




In sharp contrast, stereoselective dimerization reactions catalysed by cobalt-complexes are much less developed,^43^,^44^ even though a high pressure reaction between 1,3-butadiene and ethylene in the presence of Cl_2_Co(dppe)_2_ [dppe = 1,2-bis-diphenylphosphinoethane] and Et_3_Al to give hexadiene isomers (eqn (3)) has been known since 1967.^45^
2

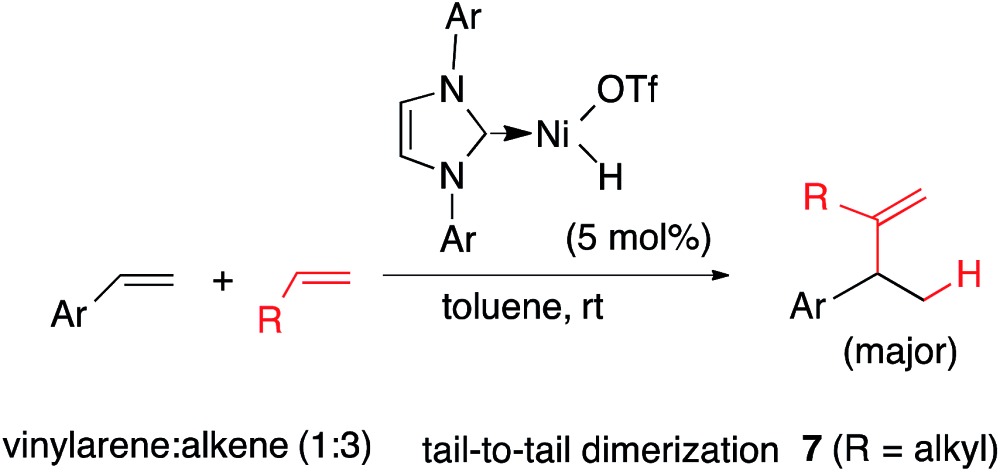



3

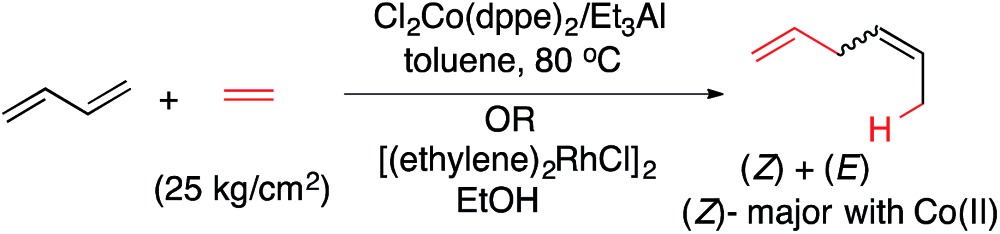




**Scheme 1:**
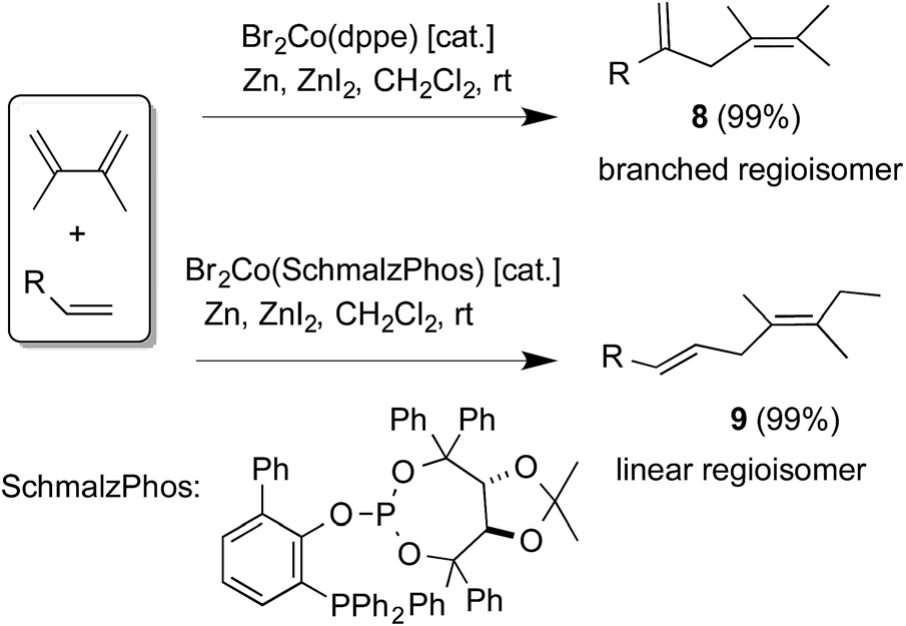
Hydroalkenylation of dienes catalysed by Br_2_Co(P ∼ P)/Zn/ZnI_2_. Ligand effects.

In 2001 Hilt *et al.* reported^46^–^48^ a remarkable hydroalkenylation reaction between 2,3-dimethylbuta-1,3-diene and a terminal alkene in the presence of Br_2_Co(dppe)/Zn/ZnI_2_ to give 1,4-addition products in very high yields and selectivities. Expansion of the scope of this reaction and several applications of this and related heterodimerization reactions have since been reported.^49^–^52^ A notable result in this area relevant to the present discussion is the ligand control of regio- and stereoselectivity in the reactions of substituted butadienes with terminal alkenes to give either the branched (**8**) or linear (**9**) adduct (Scheme 1).^48^,^53^

Expanded scope of substrates in the cobalt-catalysed hydrovinylation (HV, addition of ethylene) of alkenes and related reactions have been the subject of several recent publications. In 2006 Vogt reported the first example of a Co-catalysed asymmetric hydrovinylation of styrene using a Co(ii)-complex of the Trost ligand **10** giving modest yield and selectivity (eqn (4)).^54^,^55^ Similarly norbornene has been reported to undergo a highly efficient alkylation–hydrovinylation catalysed by a pyridine–imine cobalt complex **11** (eqn (5)).^56^ Incidentally, styrene does not undergo HV under these conditions.
4

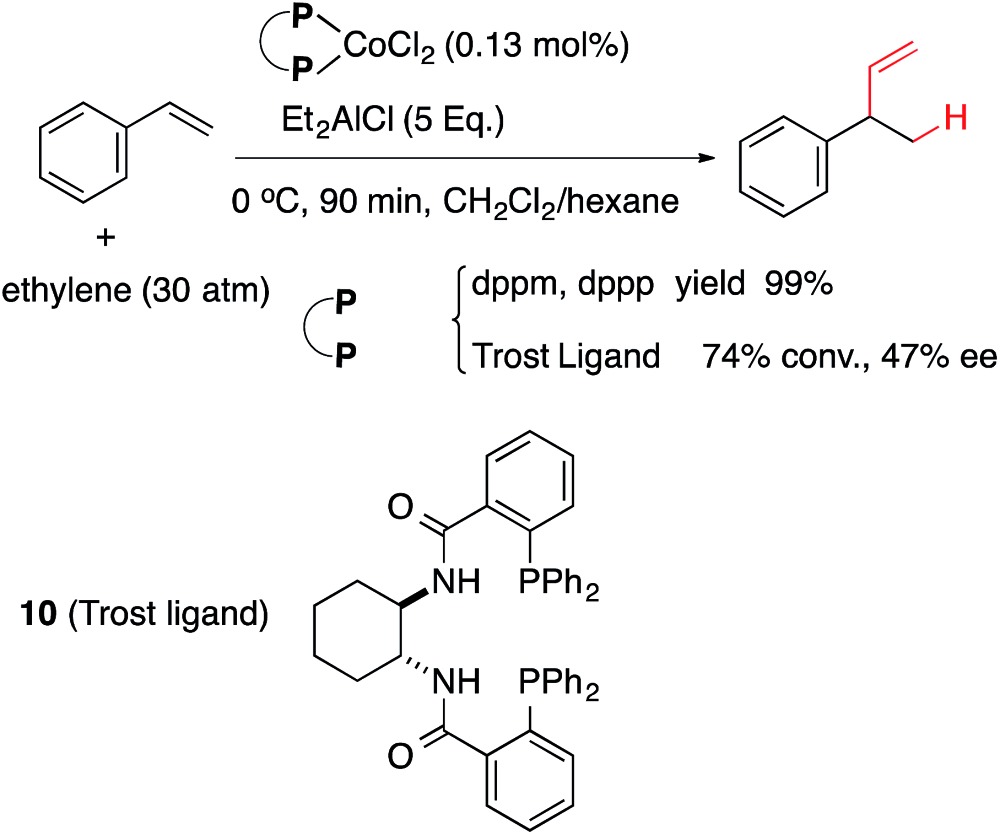



5

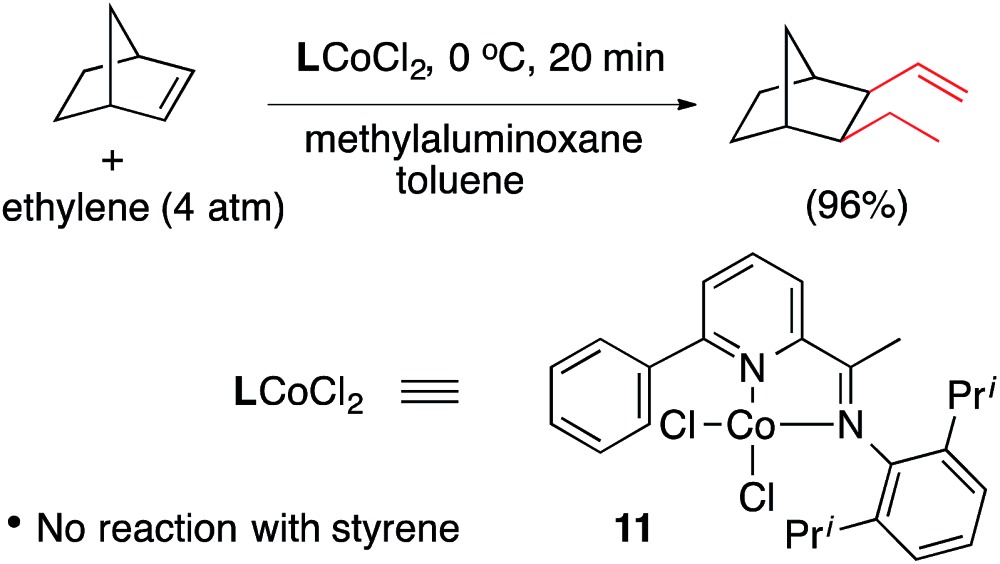



6

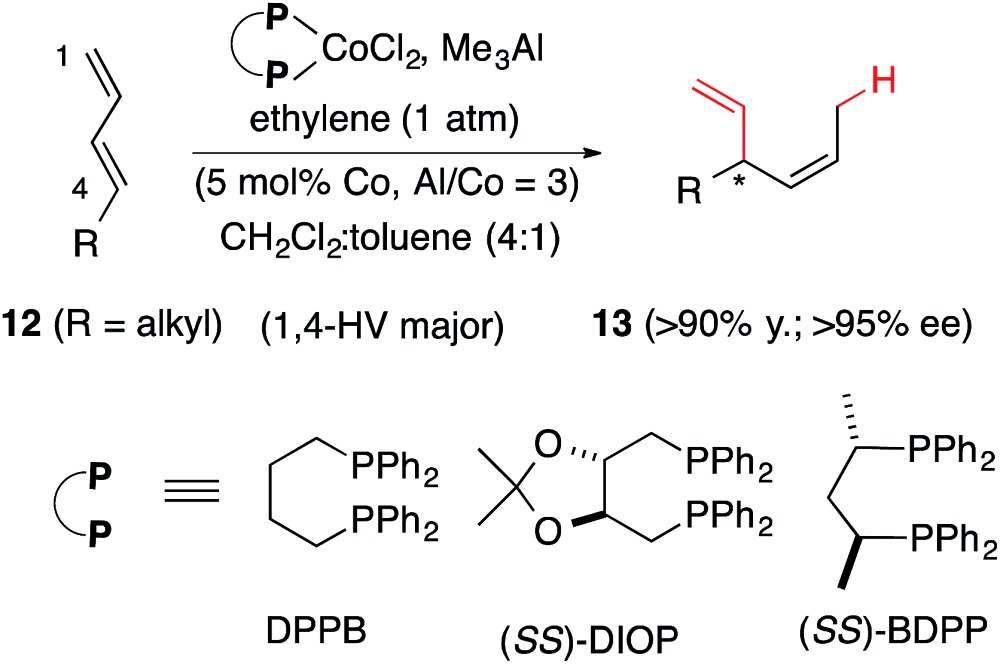




In 2010 we reported a novel protocol for the cobalt-catalysed asymmetric hydrovinylation of unactivated linear 1,3-dienes (**12**) under *one atmosphere of ethylene and temperatures in the range of –45 °C to 25 °C* (eqn (6)).57*a* This class of substrates gave unacceptable results in all previously reported HV reactions using iron,^3^ ruthenium,^25^ and nickel.^13^,^58^ We have since found that ligands and promoters play significant and, perhaps more importantly, predictable roles in the control of regio- and stereoselectivities of these reactions. Some expansion of the scope of this reaction57*b* and remarkable reactivity differences between (*Z*)- and (*E*)-terminal 1,3-dienes have also been reported.57*c* With some modifications, the new protocols for the cobalt-catalysed reactions are more broadly applicable and more substrates than originally reported undergo the reaction under moderate conditions. The full details of these studies are reported in this paper.

## Results

### Hydrovinylation of linear 1,3-dienes

**Scheme 2:**
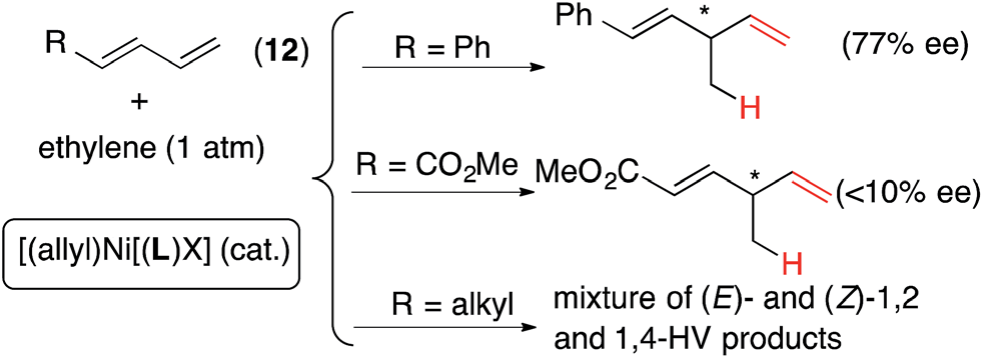
Limitations of Ni(ii)-catalysed asymmetric HV of 1,3-dienes.

1,3-Dienes are among the most readily available^59^ yet under-utilized precursors in enantioselective carbon–carbon bond-forming reactions. Thanks to advances in Wittig reactions and its alternatives, elimination, cross-coupling and cross-metathesis reactions, many structural and stereochemical variations in the dienes are possible. However, except for Diels–Alder reactions,^60^ few useful asymmetric-catalysed *intermolecular C–C bond-forming reactions* involving these substrates are known. In these instances synthetically acceptable enantioselectivities have been achieved only for *limited* reaction types [*e.g.*, cyclopropanation,^61^,^62^ and hydroformylation,^63^,^64^] and that too for a *limited* set of substrates. Ni(ii)-33,37 and Ru(ii)^25^-catalysed hydrovinylation has been moderately successful in 1-arylbutadiene and methyl 2,4-pentadienoate, yet the enantioselctivities for these substrates remain unacceptably low (Scheme 2). Attempts to carry out the Ni-catalysed asymmetric hydrovinylation of an unactivated 1,3-diene (R = alkyl in **12**, Scheme 2) such as (*E*)-1,3-octadiene under a variety of conditions led to a mixture of several products including 1,2- and 1,4-HV products.

A careful review of the extensive literature on the codimerization of butadiene and ethylene^1^,^5^,^10^,^14^,^65^,^66^ revealed that while most reactions were carried out at higher temperatures (typically >60 °C) and pressures (>20 kg cm^–3^), remarkable selectivity for the formation of the *Z*- or *E*-1,4-hexadiene can be achieved by selection of the appropriate metal/ligand combinations. While highest selectivity for the *E*-1,4-hexadiene was obtained with [(ethylene)_2_RhCl]_2_ in alcoholic medium, Cl_2_Co(ii) (P ∼ P) [(P ∼ P) = 1,2-bis-diphenylphosphinoethane) provided the best selectivity for the *Z*-1,4-hexadiene (eqn (3)). The most selective catalysts appeared to be those based on Fe(iii)^65^ and Co(ii)^5^,^7^ and we decided to concentrate our efforts on these two metals in our initial explorations.

While we have convincingly demonstrated that the Ni(ii)-catalysed hydrovinylation of vinylarenes was completely inhibited by chelating bis-phosphines,^24^,^67^ many experiments in the literature suggested that chelating ligands can be used in the cobalt- or iron-mediated reactions since these metals are capable of higher coordination numbers.^54^ Our investigations started with an examination of 1,*n*-bis-diphenylphosphinoalkane-complexes of cobalt(ii) X_2_Co[(Ph_2_P–(CH_2_)_*n*_–PPh_2_)] as catalysts in the presence of various additives in the reactions of ethylene with prototypical 1,3-dienes. Based on our work on the mechanism of the Ni(ii)-catalysed HV,^68^ we set out to generate a cobalt-hydride or an equivalent species in solution (more on this later) which could serve as a catalyst in these reactions. Several cobalt hydride species carrying bisphosphines (*e.g.* (dppe)_2_CoH,^69^,^70^ [HCo(dppe)_2_(CH_3_CN)]^+^)^71^], which could serve such a role have been fully characterized. Further we wondered whether the expanded coordination sites could have other advantages such as reducing the conformational mobility of a 1,3-diene *via* an η^4^-coordination at critical stages in the catalytic cycle (*vide infra*), and if so, could this contribute to increased selectivity in the reactions of the 1,3-dienes.

### Synthesis of the X_2_Co(P ∼ P)

The bis-phosphine complexes were readily prepared by modification of a procedure described in the literature.^70^,^72^ We^57^ and others^55^ have previously described the full characterization of the several Cl_2_Co(P ∼ P) complexes including their solid-state structures. Several more have been characterized recently.^73^ Anhydrous CoCl_2_ dissolved in freshly distilled THF (12 mL per mmol) was treated with 1.05 equivalents of the bis-phosphine for 15 minutes under argon to get a blue solution to which was added 12 mL mmol^–1^ of degassed ether to form a turbid solution. The mixture was stirred for 12–24 h and excess degassed hexane was added to precipitate the complex, which was collected and washed with 1 : 1 ether/hexane. This solid was used directly for the subsequent hydrovinylation reactions.

### Optimization of a protocol for Co-catalysed hydrovinylation. Effect of ligands

For our initial studies we chose (*E*)-1,3-nonadiene (**12a**) as a prototypical, unactivated 1,3-diene substrate. After an initial series of experiments in which the reaction parameters such as temperature, time, solvents and sequence of addition of various reagents were systematically varied, we settled on a general protocol that is outlined in eqn (7).
7

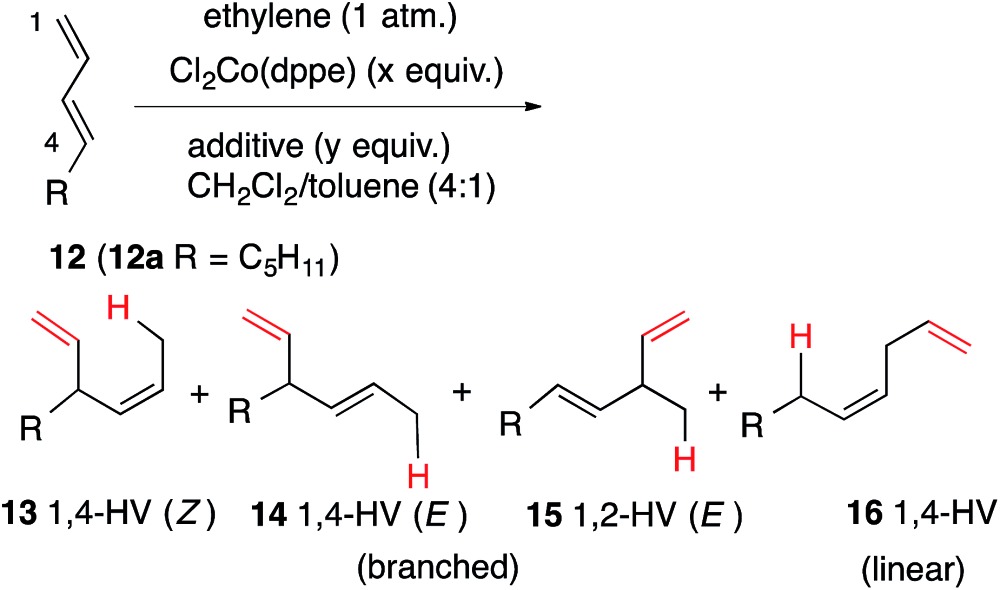




The reaction conditions and the distribution of products obtained are shown in Table 1. The results for the HV of (*E*)-1,3-nonadiene using Cl_2_Co(dppe) and trimethylaluminum as an additive (Al : Co = 3) at various temperatures are shown in entries 1–4.^74^ Thus using 10 mol% of the cobalt complex at –40 °C a clean reaction ensues giving one major (**13a**) and two minor (**14a** and **16a**) products. These products, arising from 1,4-addition across the 1,3-diene, were identified by comparison of gas chromatographic retention times and spectroscopic data with those of authentic samples, whose structures were rigorously established earlier.^57^,^74^ As the temperature is raised, the proportion of the 1,4-linear adduct, **16a**, remains constant, but increasing amounts of a new geometric isomer of the major branched product, *viz.*, **14a**, is formed (entries 2–4). The reactivity of the substrate and the distribution of products are also dependent on the bite angle of the ligand^75^,^76^ employed. The Cl_2_Co(dppp) complex is slightly better than the corresponding dppe complex in both reactivity and selectivity (compare entries 1 and 5). The most reactive and selective ligand in this series was identified as 1,4-bis-diphenylphosphinobutane (dppb), which yielded the (*Z*)-1,4-HV product **13a** in 96% isomeric purity (entry 9) in less than 0.5 h at 0 °C. Most notably, the (*E*)-isomer of the 1,4-HV (**14a**) was not detected by GC (limits of detection <0.5%). The reaction can be done equally effectively using Br_2_Co(dppb) complex using Me_3_Al as a promoter (entry 10); but the reaction fails completely when Zn/ZnI_2_ is used (entry 11), clearly establishing this procedure as distinctly different from the more reducing Hilt protocol (Scheme 1). Yet another remarkable observation, as shown in entries 12 and 13, is the effect of the narrow bite-angle (*β* = 72) ligand bis-diphenylphosphinomethane (dppm), which yielded a 1,2-HV product **15a** as the major product with very little of the 1,4-*Z* adduct (**13a**) or the 1,4-linear adduct (**16a**). The minor component in this reaction was identified as the 1,4-*E* adduct, **14a**. Catalyst derived from dppm is also very reactive, requiring only 3 mol% catalyst to complete the reaction in less than 12 h at –20 °C (entry 13). BISBI (2,2′-(diphenylphospinomethyl)-1,1′-biphenyl), a ligand with a large bite angle (*β* = 122°) also gives the 1,4-Z-adduct **13a** as the major (65%) product, but with up to 34% of the 1,2-adduct **15a**. Finally reaction of Cl_2_Co(Ph_3_P)_2_ in the presence of Me_3_Al gave mostly polymeric materials (entry 15).

**Table 1:** Hydrovinylation of **12a** (R = C_5_H_11_) catalysed by Cl_2_Co(P ∼ P). Effect of ligands and temperature^*a*^

Entry	P ∼ P	Bite angle	Cat. (mol%) Al/Co	Temp. (°C), time (h), conversion	Product, yield^*b*^ (%)			
					**13a** (1,4-*Z*)	**14a** (1,4-*E*)	**15a** (1,2-*E*)	**16a** (1,4-Linear)
1	dppe	85	10/3	–40/14/93^*c*^	78	0	0	15
2	dppe	85	10/3	–20/28/>99	64	19	0	12
3	dppe	85	10/3	–12/15/>99	66	22	0	11
4	dppe	85	10/3	0/6/>99	73	15	0	12
5^*d*^	dppp	91	10/5	–40/8/>99	85	0	0	15
6^*d*^	dppp	91	10/5	13/5/>99	76	20	0	4
7^*d*^	dppp	91	10/5	Rt/1/>99	86	8	0	5
8^*d*^	dppb	98	10/5	–10/8/>99	93	7	0	0
9^*d*^	dppb	98	10/5	0/0.5/>99	96	0	0	4
10^*d*^ ^,^^*e*^	dppb	98	10/5	–20/20/>99	94	0	0	5
11^*d*^ ^,^^*e*^ ^,^^*f*^	dppb	98	—^*f*^	Rt/4/0	0	0	0	0
12^*d*^	dppm	72	10/3	Rt/2/>99	3	30	67	0
13^*d*^	dppm	72	3/5	–20/12/>99	2	33	64	0
14	BISBI^*g*^	122	100/10	–12/6/100	65	0	34	0
15	2 Ph_3_P	—	3/5	–10/12/—^*h*^	0	0	0	0

^*a*^See eqn (7) and ESI for details.

^*b*^Estimated by GC and NMR.

^*c*^The rest is starting material.

^*d*^Entries 5–12 in neat CH_2_Cl_2_.

^*e*^Using Br_2_Co(dppb).

^*f*^Use of 20 mol% Zn, 20 mol% ZnI_2_ at 0 °C-rt for 4 h returns starting material.

^*g*^2,2′-(Diphenylphospinomethyl)-1,1′-biphenyl.

^*h*^Polymerisation.

### Identification of the hydrovinylation products

For an understanding of the mechanism of the reaction and for any further applications of the products, rigorous identification of all of the isomers of the hydrovinylation is critical. Fortunately, the remarkable ligand effects seen in these reactions enable preparation of several of the products in nearly pure state, which make their identification, and, the identification of minor products sometimes formed along with these compounds, fairly straight forward. We have also established conditions for gas chromatographic separation of all compounds including those of the enantiomers on appropriate columns.^74^ All estimates of isomeric ratios derived from proton NMR data have been corroborated by GC analysis.

**Fig. 2:**
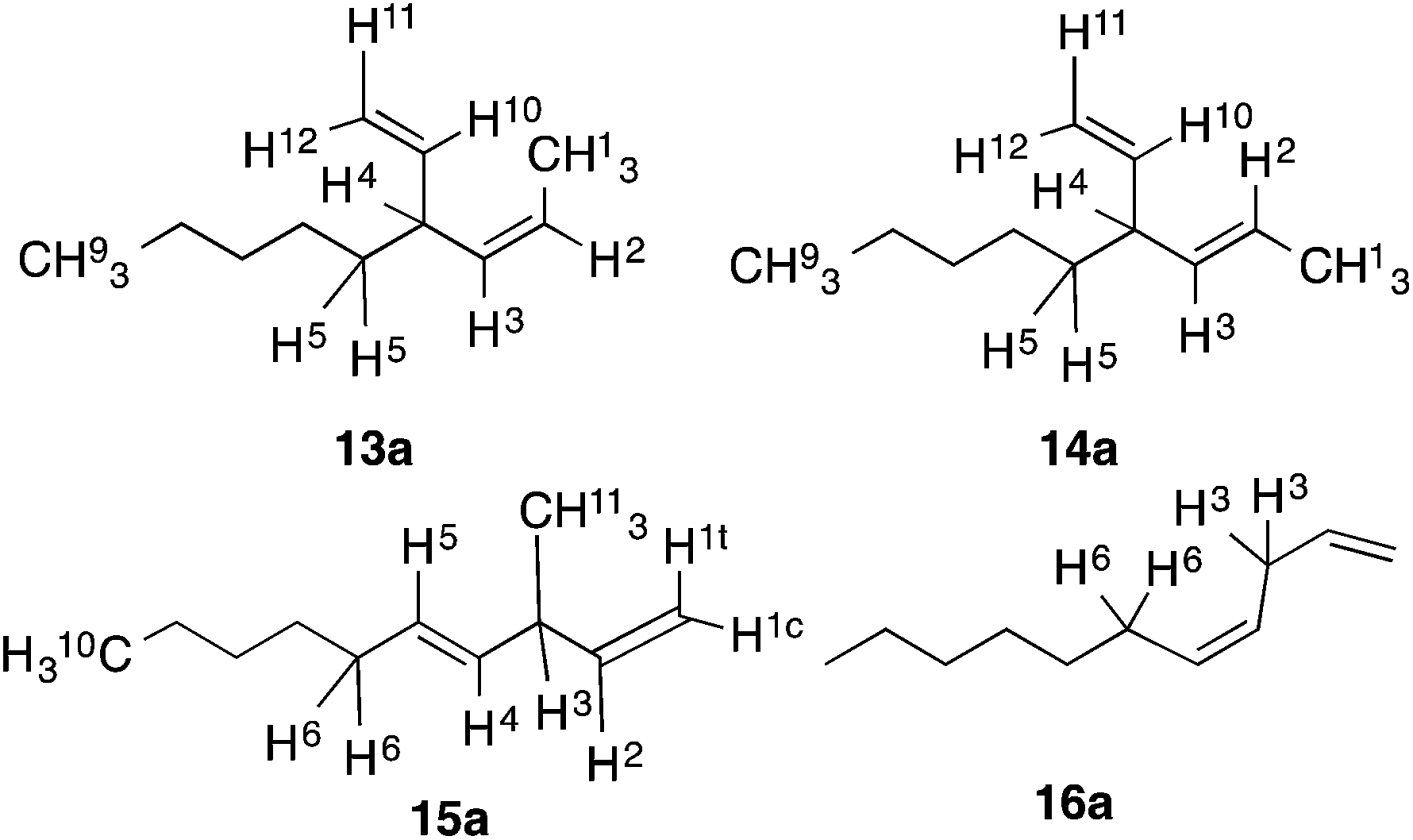
Labeling of protons in ^1^H NMR of HV products.

The products **13a**, **14a**, **15a** and **16a** derived from hydrovinylation of (*E*)-1,3-nonadiene (eqn (7)) are typical. The assignments of signals described below have been confirmed by COSY, NOESY and appropriate decoupling experiments (see Fig. 2). The major product seen in most hydrovinylation reaction, **13a**, is characterized by, among other signals, the vinyl–CH_3_–hydrogens (H^1^) which appear at *δ* 1.622 (dd, 6.5 Hz, 2.0 Hz) and the bis-allylic hydrogen (H^4^) at *δ* 3.011 (m, NOE with H^1^). The ^13^C signal for the methyl carbon linked to this *cis*-double bond appears at *δ* 14.07 ppm. In **14a**, the corresponding *trans*-1,4-HV adduct, this methyl carbon appears at *δ* 17.96, in keeping with the generally observed trend of lower field for *trans* alkene–methyls.77*a* This is also reflected in the ^1^H NMR signals of the CH_3_ groups of the *cis*- and *trans* compounds. The *trans* CH_3_ signals are slightly lower field compared to the *cis*-CH_3_ signals. In the present case the ^1^H NMR the methyl signal in the *trans*-compound **14a** appears at *δ* 1.669 (dd, 6.0 Hz, 0.5 Hz) and of the *cis*-compound (**13a**) at *δ* 1.622.77*b* The bis allylic hydrogen H^4^ in **14a** appears at *δ* 2.619 (tdd, 7.5 Hz, 7.5 Hz, 7.5 Hz). This signal, corresponding to the bis-allylic hydrogens, is distinctly different for all 4 isomers and is highly diagnostic. The 1,2-HV-adduct **15a** is characterized by an up-field methyl doublet at *δ* 1.075 (d, *J* = 6.5 Hz, H^11^) and a bis-allylic hydrogen (H^3^) signal at *δ* 2.807 (ddq, 6.0 Hz, 6.0 Hz, 6.0, Hz). The (*E*)-geometry of the alkene is obvious from the large *J*_4,5_ value in **15a** (15.5 Hz). Closely related compounds have been described in the literature.^78^,^79^ The linear adduct **16a** is characterized by bis-allylic H's (H^3^) at *δ* 2.778 (t, 2H) and allylic H's (H^6^) at *δ* 2.019 (q, 2H). The GC retention time of this isomer (**16**) in every case is significantly longer than the other three isomers on a methylsilicone column. In addition, **16a** is the only compound among the adducts that is achiral, and thus showing no resolution on the chiral stationary phase (CSP) GC columns. On a typical 30 m polymethylsiloxane column at 80 °C the following retention times are observed for the various adducts: **13a**: 21.169 min.; **14a**: 21.93 min.; **15a**: 22.77 min.; at 75 °C: **13a**: 28.22 min, **16a**: 40.77 min. CSP GC (Cyclodex B) 60 °C **13a**: *R*_T_ = 26.07 min. (*S*), 27.25 (*R*); **16a**: 44.79 min. The assignment of the absolute configuration is based on the observation that the product from Fe-catalysed asymmetric hydrovinylation of 1,3-pentadiene^3^ gave the laevorotatory product (*S*) as the major one and the same product was obtained in the asymmetric HV of 1,3-pentadiene using Cl_2_Co[(*R*,*R*) (–)-DIOP]. Accordingly, the levorotatory enantiomer was assumed to have the (*S*)-configurations. Configurations of all other 1,4-adducts were assigned by analogy (Fig. 2).

### Promoters

Even though methyl–aluminum reagents such as methylaluminoxane have been widely used for the activation of cobalt–halide complexes in polymerisation reactions,^19^,^20^,^80^ the use of trimethylaluminum in our reactions is novel and noteworthy, since it has been reported that in cobalt-catalysed hydrovinylation of styrene this additive is ineffective.^55^ Our protocol is also distinctly different from the hydroalkenylation of 1,3-dienes reported by Hilt in which a bromide complex Br_2_Co(P ∼ P) [(P ∼ P) = dppm, dppe, dppp] is used along with Zn and ZnI_2_ as additives (Scheme 1) to get high yields of the adducts (**8**). While we find that Br_2_Co(dppb) complex works just as well as the corresponding chloride complex in our protocol using Me_3_Al (eqn (8)), this bromide complex does not catalyse the HV reaction in the presence of Zn and ZnI_2_ (eqn (9), Hilt conditions, also entry 17, Table 2).^81^
8

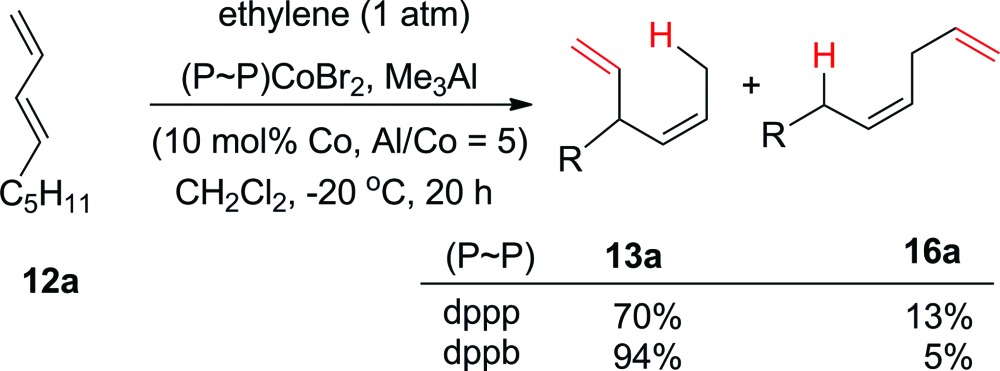



9

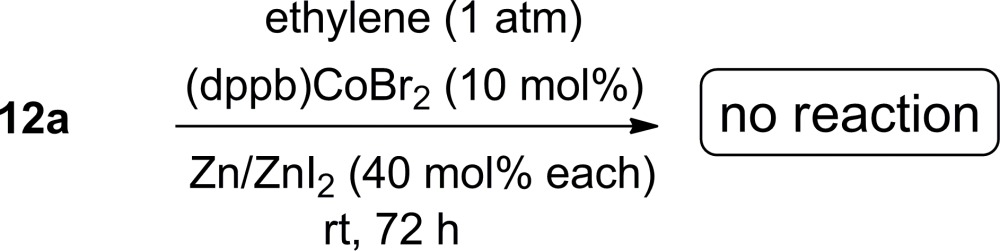




**Table 2:** Effect of promoters in the X_2_Co(P ∼ P)-catalysed hydrovinylation of 1,3-dienes^*a*^

Entry	P ∼ P	Additive (mol%)	Temp (°C), time (h)	Conversion [products]
1	dppp	No additive	Rt (2)	0
2	dppp	Me_3_B (100)	Rt (2)	0
3	dppp	Et_3_B (100)	Rt (2)	0
4	dppp	Ph_3_B (100)	Rt (2)	0
5	dppp	*i*-BuAlH (100)	Rt (2)	0
6	dppp	LiEt_3_BH (100)	Rt (2)	0
7	dppb	Zn/ZnI_2_ (5, 5)	Rt (16)	0
8	dppe	PhMgBr (400)	Rt (7)	0
9	dppe	Mn (100)	Rt (14 h)	0
10	dppe	InI_3_ (100)	–10 – rt (10)	0
11	dppe	Et_2_AlOEt (100)	Rt (2)	<2%
12	dppm	MeMgBr, AgOTf (100, 100)	0 – rt (4)	0
13	dppb	Et_2_AlCl (50)	–10 (2)	0^*b*^
14	dppb	EtAlCl_2_ (50)	–10 (2)	0^*b*^
15	dppp	Zn/ZnI_2_ (20, 20)	0 – rt (5)	0
16	dppe	Zn/ZnI_2_ (20, 20)	0 – rt (5)	72 [56% 1,4-*Z* (**13a**); 11% 1,4-*Z*-lin (**16a**)]^*c*^
17	Br_2_Co (dppb)	Zn/ZnI_2_ (20, 20)	0 – rt (4)	0
18^*d*^	Br_2_Co (dppb)	Zn/ZnI_2_ (10, 10)	Rt (16)	0
19	Br_2_Co (dppe)	Zn/ZnI_2_ (20, 20)	0 – rt (4)	100 [85% 1,4-*Z* (**13a**); 15% 1,4-*Z*-lin (**16a**)]
20	Br_2_Co (dppp)	Zn/ZnI_2_ (20, 20)	0 – rt (4)	100 [79% 1,4-*Z* (**13a**); 21% 1,4-*Z*-lin (**16a**)]

^*a*^See eqn (7) and ESI for details. All using Cl_2_Co(P ∼ P) unless indicated otherwise. Entries 13–20 using (*E*)-C_8_H_17_–CH=CH–CH=CH_2_.

^*b*^No volatile products (polymers?).

^*c*^2% Each 2 other isomers.

^*d*^ Ref. 81 (in hydroalkenylation of isoprene).

In light of the observations documented in the previous paragraph, it is of interest to note that several of the commonly used promoters (hydride reagents, alkylating agents, Lewis acids) also do not promote the hydrovinylation reactions in the presence of Cl_2_Co(P ∼ P) complexes (Table 2, entries 1–15). Combination of Zn/ZnI_2_ does affect the reaction giving moderate yields of **13a** and **16a** if Cl_2_Co(dppe) is used instead of Cl_2_Co(dppp) (entries 15 *versus* 16, Table 2). The corresponding complexes with CoBr_2_ catalyze the reaction, but with significant proportion of the linear 1,4-adduct **16** (entries 19 and 20). This stark difference between various bis-diphenylphosphine ligands (dppm, dppe *vs.* dppb) has been noted earlier by Hilt in his hydroalkenylation studies (entry 18, Table 2).^81^

### Ligands

**Fig. 3:**
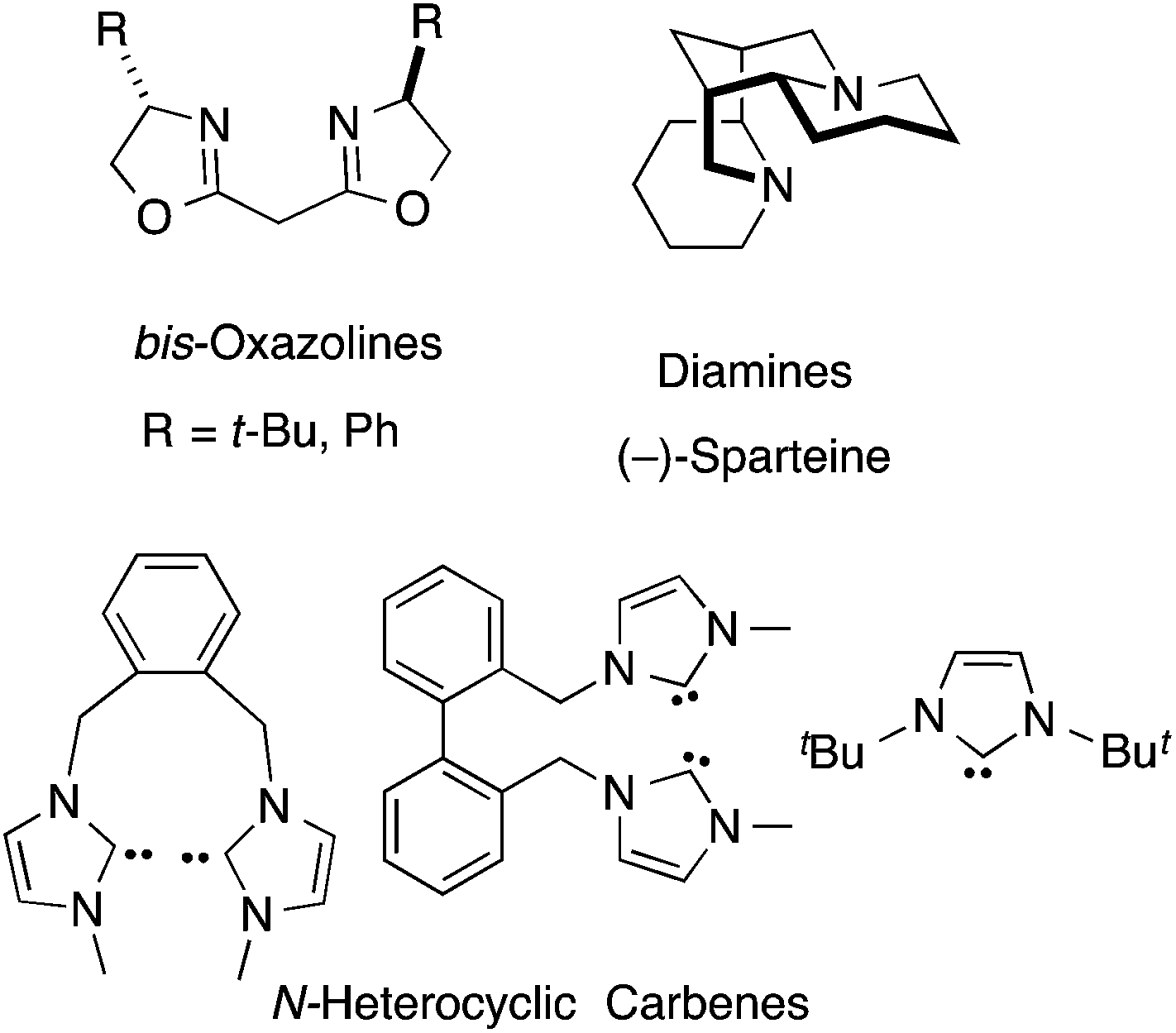
Assorted ligands found unsuitable for Co(ii)-catalysed hydrovinylation.

Having identified a chelating bis-phosphine and Me_3_Al as a viable ligand/promoter combination for an efficient cobalt-catalysed HV, we also examined three other popular classes of ligands, bis-oxazolines, chelating bis-amines and *N*-heterocyclic carbenes (Fig. 3) for this reaction. Cobalt(ii)-complexes of these ligands failed to yield any HV products in reactions of (*E*)-1,3-nonadiene under conditions similar to what has been prescribed for the bis-phosphines in eqn (7).

### Scope of 1,3-diene substrates

The full scope of the new protocol for the Co-catalysed hydrovinylation reaction was examined with the help of the substrates shown in Table 3. The general optimized procedure used for the reactions is shown in eqn (10). In this procedure, a round-bottomed flask with a side-arm is charged with the X_2_Co(P ∼ P) complex (5–10 mol%) in degassed methylene chloride at 0 °C under argon. When methylaluminoxane (MAO) is used as the promoter, it is charged to the reaction flask right after the addition of the cobalt-complex. If Me_3_Al is used, a solution of Me_3_Al in toluene is added to the Co-complex and the reaction vessel is carefully evacuated and refilled with ethylene from a balloon, which is kept in place until the reaction is quenched. It is subsequently cooled to the prescribed temperature and the substrate is added and the mixture stirred for the periods shown in the table. Progress of the reaction maybe followed by gas chromatography, where baseline separation of the various possible adducts is observed. Excess methanol is added to quench the reaction and the products are extracted with pentane/ether. The conversions generally are quantitative under conditions shown in the table and, the yield, as judged by estimated weight of the product and its purity (GC and NMR) is >95% in most cases. The lower yields of some of the products result from the volatility of the hydrocarbon products. Attempts to remove last traces of the solvent (especially on small scales) result in some loss of materials. Details of the characterization of the isomeric products obtained from prototypical substrates (eqn (7)) and the diagnostic peaks in the ^1^H NMR that characterize the various isomers are documented in the ESI.†

10

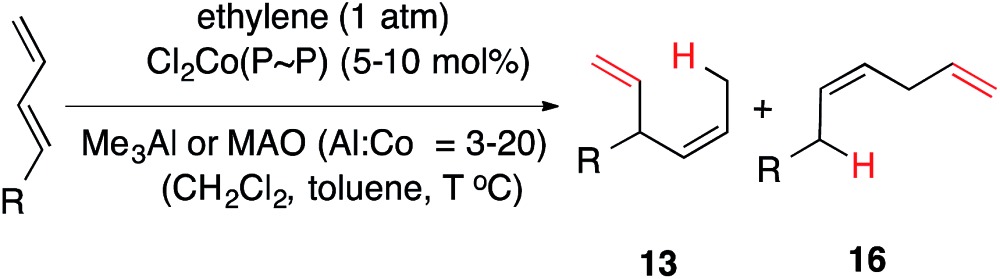




**Table 3:** Scope of substrates in the Co-catalysed hydrovinylation of linear 1,3-dienes^*a*^

Entry	Diene (**12**) R in **12**eqn (10)	Cl_2_Co(P ∼ P) (P ∼ P), mol%	Al/Co	Conditions temp. (°C)/time (h)	Yield	
					**13**	**16**
1	C_5_H_11_ (**12a**)	dppb (10)	5	0/0.5	>95	
2	C_6_H_13_ (**12b**)	dppb (5)	3	–10/6^*b*^	>95	
3	C_7_H_15_ (**12c**)	dppb (5)	3	–10/6^*b*^	>95	
4	C_8_H_17_ (**12d**)	dppb (5)	3	–10/6^*b*^	>95	
5	C_8_H_17_ (**12d**) (*E* : *Z* 54 : 46)	dppb (10)	20	–10/8^*c*^	82	
6	Cyclohexyl (**12e**) (*E* : *Z* 45 : 55)	dppb (10)	20	–10/8^*c*^	90	
7	CH_3_ (**12f**)	dppb (5)	3	–10/6^*b*^	>95	
8	Ph (**12g**)	dppp (10)	3	–20/4	0	>99 (86)
9		dppb (10)	3	–10/6	0	>99 (87)
10		dppm (10)	3	–20/7	0	0^*d*^
11	Ph (**12g**) (*E* : *Z* 40 : 60)	dppb (10)	20	–10/8^*c*^	0	>99
12	CH_2_CO_2_Et (**12h**)	dppp (10)	10	5/11	0	0
13		dppb (10)	10	5/11	0	0
14		dppm (10)	10	0/15	84	
15	CH_2_CH_2_OBn (**12i**)	dppb (10)	20	–15/14^*c*^	>97	<2
16	CH_2_CH_2_Ph (**12j**) (*E* : *Z* 53 : 47)	dppb (10)	20	–10/8^*c*^	84	
17	4-Me_2_N–C_6_H_4_ (**12k**)	dppb (10)	20	0/13^*c*^	0	79

**Other dienes**						
18	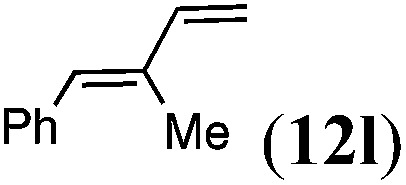	dppm (5)	3	–10/6^*b*^	0	0^*e*^
19		dppp	3	10/6	0	0^*f*^
20	β-myrcene (**12m**)	dppb (10)	3	0/6	0	>95^*g*^
21		dppp (10)	3	Rt/1	0	83^*g*^
22	Isoprene (**12n**)	dppp (10)	10	–20/4	0	>99

^*a*^See eqn (10) for procedure.

^*b*^Solvent CH_2_Cl_2_ : toluene 4 : 1.

^*c*^MAO as ‘Al–Me’ source.

^*d*^1,2-branched product **15g** (99%).

^*e*^1,2-branched product **15l**, 97%.

^*f*^Product **15l** 62%.

^*g*^Co : Al 1 : 3 (*Z*)-2-methyl-6-(3-propenyl)octa-2,6-diene (**16m**). See Fig. 4 for structures of **15g**, **15l**, **16g**, **16k**, **16m** and **16n**.

As shown in eqn (10), the cobalt-catalysed hydrovinylation of 1,3-dienes is most useful to prepare two types of adducts, 1,4-adducts **13** and **16** depending on the nature of the substituent R and the chelating bis-phosphine that is used. In general, alkyl- and substituted alkyl-1,3-dienes give excellent yields of the 1,4-(*Z*)-adduct **13** (entries 1–7) with the Cl_2_Co(dppb) complex with either Me_3_Al or MAO as the activating agent. The ester bearing substrate **12h** (entries 12–14) is uniquely different in its reactivity in that neither the commonly used dppp complex (entry 12) nor the dppb complex (entry 13) is able to effect the hydrovinylation of this substrate. Yet a cobalt complex with a bis-phosphine ligand with a narrow bite-angle, dppm gives an excellent yield of the product **13h** (entry 14). Recall that dppm-complex with the substrate with a simple alkyl substituent (**12a**) gave a 1,4 *E*-adduct **14a** and a 1,2 *E*-adduct **15a** (Table 1, entries 12 and 13).

**Fig. 4:**
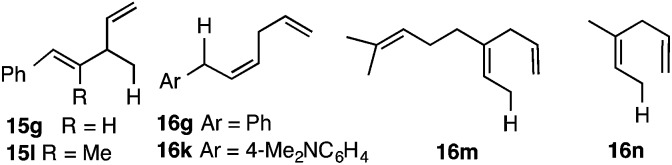
Major Products from **12g**, **12k**, **12l**, **12m**, **12n**.

1,3-Dienes with aromatic ring in conjugation with the diene belong to a class of substrates that behaves dramatically differently (**12g**: entries 8–11, **12k**: entry 17). These substrates give linear 1,4-adducts **16g** and **16k** (Fig. 4) with a (*Z*)-geometry in the HV reactions catalysed by Cl_2_Co(dppb) and Cl_2_Co(dppp) complexes (entries 8, 9, 17). The Cl_2_Co(dppm) complex, in sharp contrast, gives exclusively a 1,2-(*E*)-adduct, **15g** (entry 10, Fig. 4). Most notably, *there is no contamination from the isomeric impurities* in either of these reactions. Likewise (*E*)-2-methyl-1-phenyl-1,3-butadiene (**12l**) gives only a 1,2-HV product (**15l**, Fig. 4) with both the dppm and dppp complexes (entries 18 and 19).

3-Substituted 1,3-butadienes, β-myrcene (**12m**) and isoprene (2-methyl-1,3-butadiene, **12n**) gave very high yields of 1,4-linear adducts **16m** and **16n** (Fig. 4) in which the hydrogen adds to the less substituted double bond (entries 20–22).

#### Hydrovinylation of (*Z*)- *vs.* (*E*)-1,3-dienes

While the increased reactivity of 1,3-dienes which can readily assume *s-cis* conformation (for example *E*- *vs. Z*-1,3-pentadiene) has been noted in the Fe(iii)-catalysed hydrovinylation reactions,^1^ under cobalt-mediated reactions reported here there is much less discrimination between the (*E*)- and (*Z*)- isomers. Nearly quantitative yields of the expected products are formed upon reaction of (*E*/*Z*)-mixtures of dienes **12d**, **12e**, **12g** and **12j** (entries 5, 6, 11, 16) with ethylene using Cl_2_Co(dppb) complex and Me_3_Al or MAO. However, as will be described later, chiral ligands (*S*,*S*)-DIOP and to some extent (*S*,*S*)-BDPP, react much faster with the (*E*)-isomers of **12d** and **12e**, leaving behind most of the Z-isomer unreacted (*vide infra*).

#### Cobalt(ii)-catalysed heterodimerization between nona-1,3-diene and propylene

Upon reaction with [(allyl)NiBr]_2_ and Ph_3_P, propylene and various vinylarenes undergo efficient Ni-catalysed heterodimerization reactions.^82^ Various attempts to carry out this reaction under conditions that enabled the addition of ethylene to (*E*)-nona-1,3-diene using Cl_2_Co(P ∼ P)/Me_3_Al gave no conversion of the starting material (eqn (11)).
11

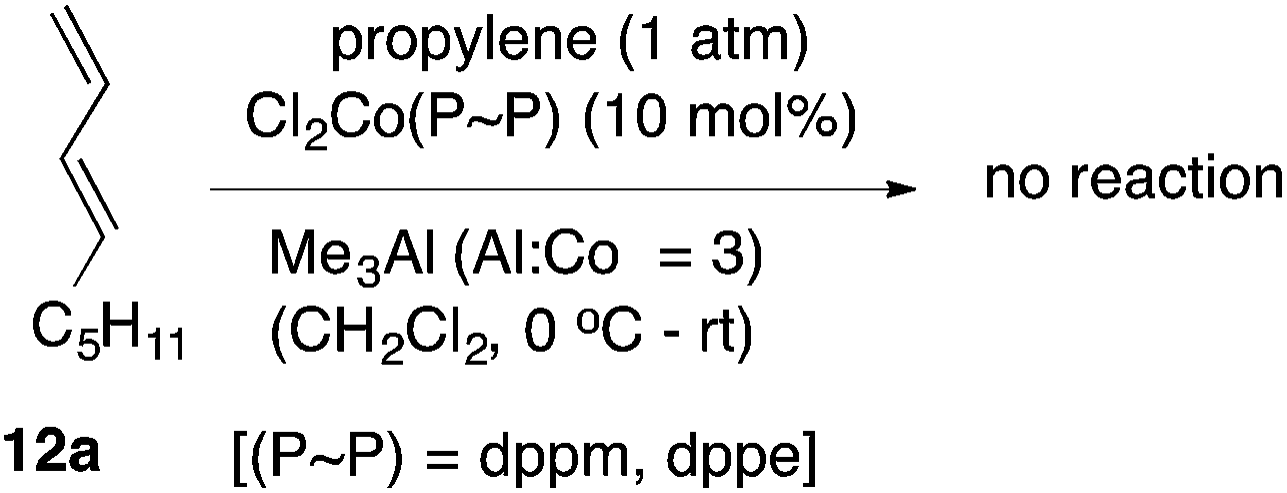




### Iron(iii)-catalysed codimerization of ethylene and (*E*)-nona-1,3-diene

Iron(iii)-catalysed codimerisation of ethylene and 1,3-butadiene (60 kg cm^–2^, 30–80 °C) in the presence of alkylaluminum reagents is among the earliest reported examples of these reactions.^1^,^2^ Other related reactions involving Fe-catalysis include codimerization of 1,3-pentadiene and ethylene,^3^ dimerization of α-olefins,^83^ and 1,4-addition of α-olefins to dienes.^84^ In a brief exploration to establish a direct comparison of reactivity of Cl_3_Fe(dppe) with Cl_2_Co(dppe) revealed that the former is much less reactive, with no particular advantages in terms of selectivity of products obtained, even though 1,4-*Z*-adduct **13a** is the major product in both cases. One of the moderately successful experiments is depicted in eqn (12).
12

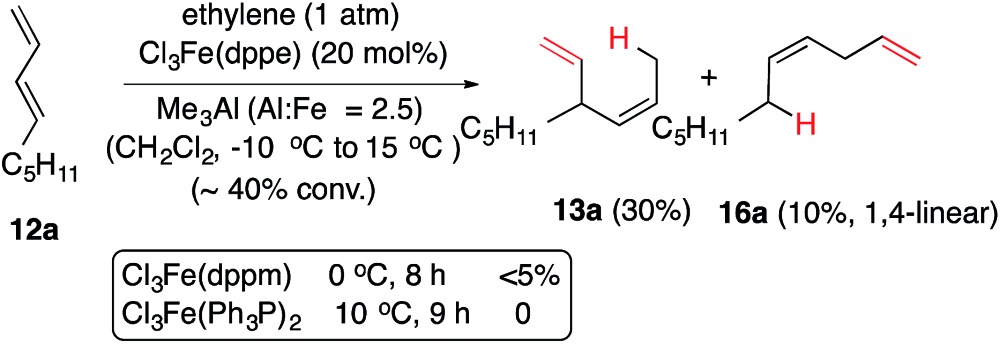




### Asymmetric hydrovinylation of 1,3-dienes

#### Effect of chiral ligands

**Fig. 5:**
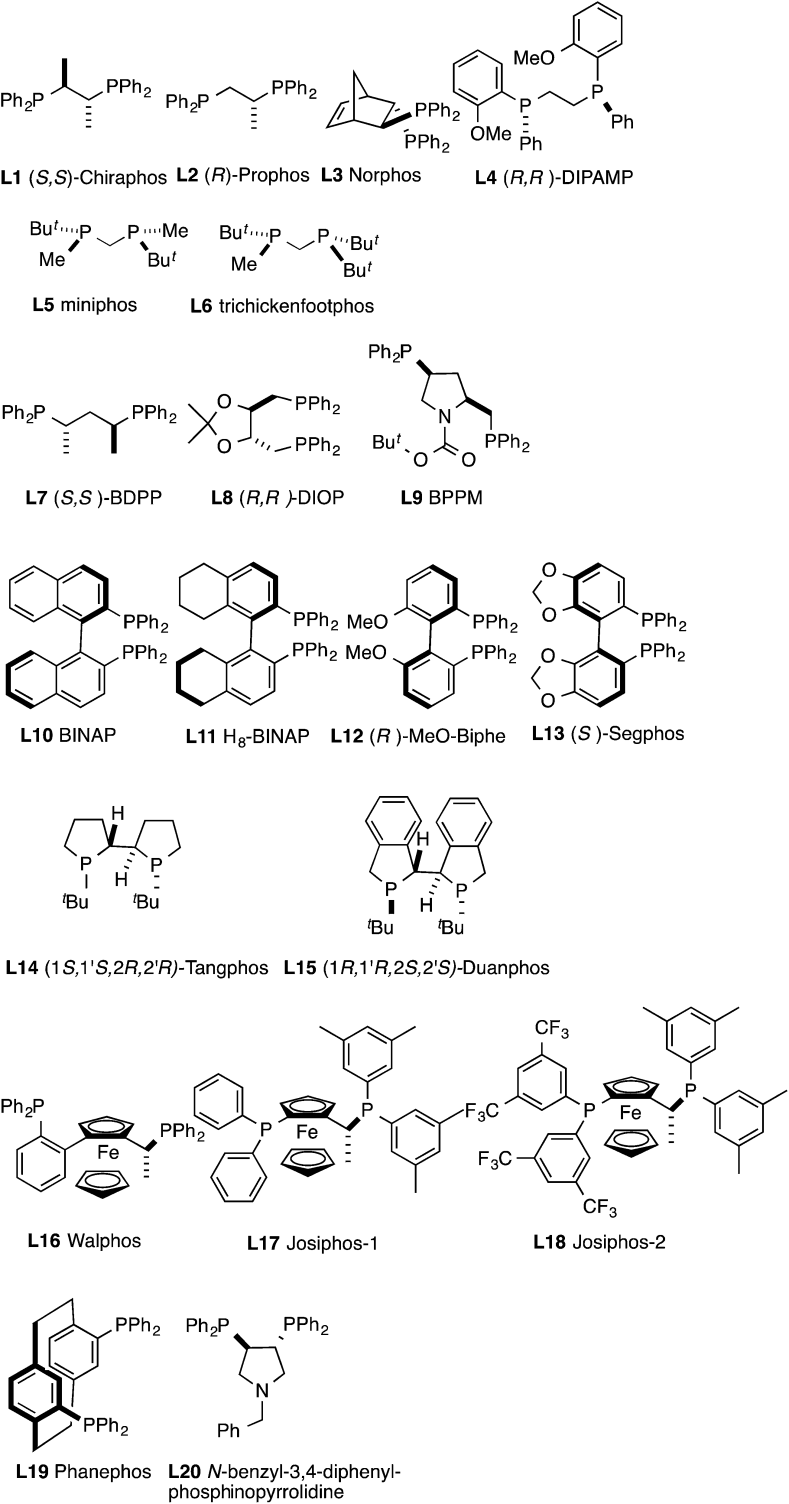
Ligands for cobalt(ii)-catalysed asymmetric hydrovinylation of 1,3-dienes.

The remarkable facility with which chelating bis-phosphine complexes of Co(ii) catalyse the chemo- and regioselective hydrovinylation of 1,3-dienes prompted further investigations into an asymmetric version of this reaction. Hydrovinylation of a prototypical substrate **12a** (R = C_5_H_11_) was examined under conditions shown in eqn (13) using Co(ii)-complexes of several commercially available enantiopure chiral ligands. The structures of these ligands are shown in Fig. 5. The distribution of products and the enantioselectivities of the chiral products obtained are listed in Table 4.

**Table 4:** Enantioselective HV of (*E*)-nona-1,3-diene. Effect of chiral ligands^*a*^

Entry	Ligand^*c*^	Conversion	Products (%ratio/%ee)^*b*^			
			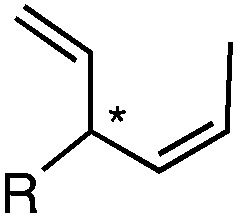 **13a**	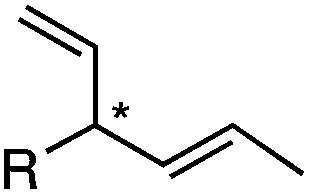 **14a**	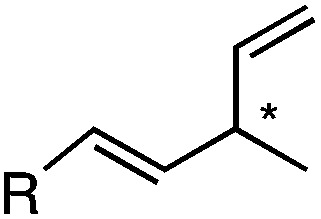 **15a**	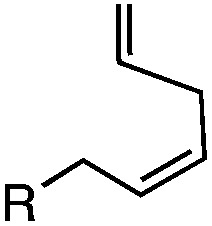 **16a**
1	**L1**	99	41/78(*S*)	4/—	55/16	0
2	**L2**	99	18/44	15/—	62/1	2
3	**L3**	99	90/8	0	0	9
4	**L4**	66	54/1	0	0	12
5	**L5**	40^*d*^	7/—	—	31/8	
6	**L6**	0^*d*^	—	—	—	—
7	**L7**	99	96/97(*R*)	0	0	<1
8	**L8**	99	95/95(*S*)	0	0	<1
9	**L9**	99	86/64(*S*)	11/—	2/—	0
10	**L10**	0	—	—	—	—
11	**L11**	0	—	—	—	—
12	**L12**	88	66/8	0	0	7
13	**L13**	95	63/14	0	11/31	4
14	**L14**	80	39/95(*S*)	0	0	40
15	**L15**	∼6^*e*^	6/—	0	0	0
16	**L16**	99	0	28/20	59/12	0
17	**L17**	87^*d*^	79/10	0	0	8
18	**L18**	95	95/87(*S*)	0	0	4
19	**L19**	0	—	—	—	—
20	**L20**	0	—	—	—	—

^*a*^See eqn (13) for typical procedure (R = C_5_H_11_).

^*b*^Determined by CSP GC.

^*c*^See Fig. 5 for structures of ligands.

^*d*^At –45 °C/8 h.

^*e*^Rest starting material.

The reactivities of the chelating chiral ligands roughly parallel those of the corresponding achiral ligands with similar bite angles. In general, bis-diarylphosphino-ligands with bite angles comparable to dppe, dppp and dppb are the most reactive (Table 4, entries 1–3 and 7–9, 17, 18; ligands **L1**, **L2**, **L3**, **L7**, **L8**, **L9**, **L17**, **L18**). As expected, ligands with narrow bite-angles, (*S*,*S*)-chiraphos (**L1**) and (*R*)-prophos (**L2**) give, as the major product the 1,2-HV product **15a**, along with minor amounts of **13a**, the 1,4-(*Z*)-adduct (entries 1 and 2). The enantioselectivities for these products are low, with the ee for **13a** almost always higher than that of the any other adducts in these reactions. Two other phosphines containing 2-carbon chains in the backbone, **L3** and **L4**, give **13a** as the major product with very low enantioselectivities (entries 3 and 4). Two chiral analogs of dppm, miniphos **L5** and trichickenfootphos **L6** are electron-rich phosphines,^85^ which are poor ligands for this reaction (entries 5 and 6).
13

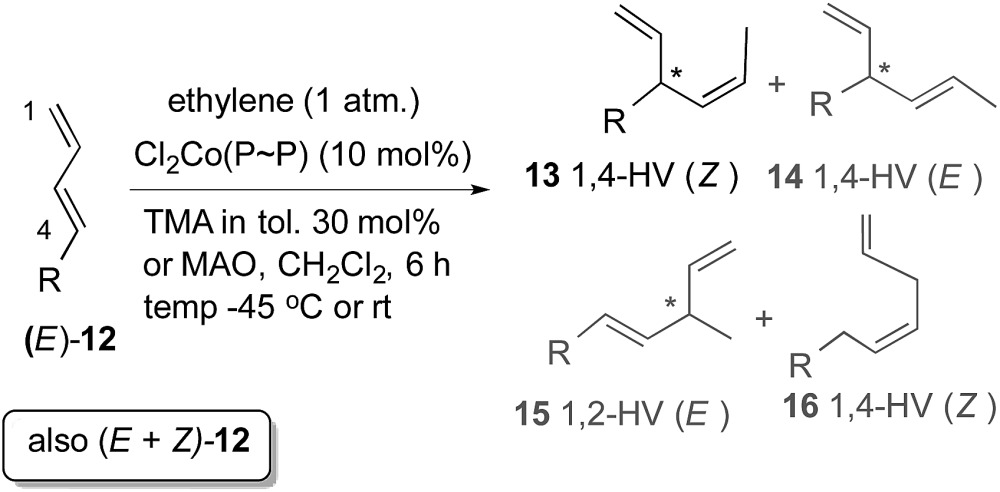




The dppp analog (*S*,*S*)-BDPP (**L7**) and dppb analog (*R*,*R*)-DIOP (**L8**) gave the best yields (99%) and highest enantioselectivities (97% and 95% respectively) for the hydrovinylation of 1,3-nonadiene (entries 7 and 8). In these cases less than 1% of the isomeric linear product (**16a**) is formed as the only contaminant. A constrained analog of dppb, **L9**, derived from proline also gives **13a** as the major product, albeit in relatively low ee (entry 9).

2,2′-Diphenylphosphino-1,1-biaryls are among the most widely studied ligands in asymmetric catalysis, and in the present case only the biaryl ligands that gave any products are (*R*)-MeO-Biphe (**L12**) and (*S*)-Segphos (**L13**). Only low ee's (entries 12 and 13) were observed in these cases. Co(ii)-complexes of BINAP (**L10**) and H_8_-BINAP (**L11**) failed to affect the reaction (entries 10 and 11).

Cobalt(ii) complexes of electron-rich phospholanes **L14** and **L15** are poor catalysts, with the latter giving <6% conversion (entries 14 and 15). It is not unreasonable to assume that in the cases of ligands **L6** (entry 6) and **L15** (entry 15) the reactions might be retarded by both electronic and steric properties of these ligands. Yet, Tangphos (**L14**), an electron-rich bis-phospholane is among the best ligands (95% ee) for the formation of the 1,4-*Z*-HV product **13a**, even though it gave up to 40% of the linear achiral product **16a** (entry 14).

The complex derived from ferrocene-based ligand Walphos (**L16**) is the only ligand among the many we tested that gave *none of the most commonly observed major product***13a** (entry 16). This complex gave the (*E*)-1,4 and (*E*)-1,2- adducts **14a** and **15a** as the major products, both in only modest selectivities (entry 16). Two other related ligands Josiphos-1 (**L17**) and Josiphos-2 (**L18**) are among the best ligands, the latter giving up to 87% ee for the branched product **13a**, with <4% of the linear adduct **16a** as a contaminant (entry 18). A remarkable electronic effect was observed in going from complex of **L17**, a ligand with diphenylphosphino-substituent, to one with a more electron-withdrawing di-(bis-1,3-CF_3_-phenylphosphino)-ligand **L18**. The enhancement of reactivity (yield: improvement from 87% to >95%) and selectivity (from 10% ee to 87% ee) observed in this ligand change is quite uncommon, and is reminiscent of similar effects seen in Ni-catalysed asymmetric hydrocyanation of vinylarenes.^86^ Electronic tuning of ligands might offer an attractive option to enhance enantioselectivity in the asymmetric hydrovinylation of 1,3-dienes. In addition the complex from ligand Josiphos 2 (**L18**) effects these reactions at 0.01 equivalent loading level (See Table 5, entries 4, 7, 9).

**Table 5:** Co-catalysed asymmetric HV of linear 1,3-diene^*a*^

Entry	Diene (**12**) R in **12**eqn (13)	(P ∼ P) in Cl_2_Co(P ∼ P)	Al/Co	Conditions temp. (°C)/time (h)	Yield (%)	**13** (%ee)^*b*^	**16** (%)
1	C_5_H_11_ (**12a**-*E*)	**L8** (*R*,*R*)-DIOP	3	–45/6	>95	95.0(*S*)	
2		**L7** (*S*,*S*)-BDPP	3	–45/6	97.1	97.1(*R*)	
3		**L14** Tangphos	3	–10/8	39	95.0(*S*)	40
4		**L18** Josiphos 2^*c*^	3	–20/14	>95	87.0(*S*)	<4
5	C_6_H_13_ (**12b**-*E*)	**L8** (*R*,*R*)-DIOP	3	–45/6	>95	95.3(*S*)	
6	C_7_H_15_ (**12c**-*E*)	**L8** (*R*,*R*)-DIOP	3	–45/6	>98	95.4(*S*)	
7		**L18** Josiphos 2^*c*^	3	–20/14	>95	87.0(*S*)	<3
8	C_8_H_17_ (**12d**-*E*)	**L8** (*R*,*R*)-DIOP	3	–45/6	95	96.1(*S*)	
9		**L18** Josiphos 2^*c*^	3	–20/14	88	86.0(*S*)	<3
10	C_8_H_17_ (**12d**) (*E* : *Z* = 54 : 46)	**L8** (*S*,*S*)-DIOP	5	–45/1	53^*d*^	74.0(*R*)	5
11	Cyclohexyl (**12e**) (*E* : *Z* = 47 : 53)	**L8** (*S*,*S*)-DIOP	3	–10/8	49^*e*^	84.0(*R*)	5
12	CH_3_ (**12f**-*E*)	**L8** (*R*,*R*)-DIOP	3	–45/6	>95^*f*^	90.1(*S*)	
13	Ph (**12g**-*E*)	**L8** (*S*,*S*)-DIOP	3	0/5	46	—^*g*^	55
14	CH_2_CO_2_Et (**12h**-*E*)	**L7** (*S*,*S*)-BDPP	10	0/15	84	92(*R*)	*x*
15		**L18** Josiphos 2^*h*^	3	10/8	0	0	0
16	CH_2_CH_2_OBn (**12i**-*E*)	**L8** (*R*,*R*)-DIOP	3	–20/6	40^*i*^	99.0(*S*)	0
17		**L8** (*S*,*S*)-DIOP^*j*^	3	–10/6	99	94.0(*R*)	6
18		**L7** (*S*,*S*)-BDPP^*j*^	3	–10/9	>99	92.0(*R*)	0

^*a*^See eqn (13) for procedure.

^*b*^Determined by CSP GC. Configurations are tentative and are based on the known product of HV of 1-methylbuta-1,3-diene (see Ref. 3). See ESI for details.

^*c*^1 mol% Co.

^*d*^Reaction stopped after most of the (*E*)-isomer was converted, recovered starting material (*E* : *Z* = 1 : 17).

^*e*^Reaction stopped after most of the (*E*)-isomer is converted, recovered starting material (*E* : *Z* = 1 : 49).

^*f*^Estimated by GC, volatile products.

^*g*^1,2 –Adduct, **15g** (43% yield; 52% ee), rest linear **16g**.

^*h*^5 mol%.

^*i*^Rest starting material.

^*j*^Using MAO.

Finally two other ligands, Phanephos (**L19**) and the bis-phosphinopyrrolidine **L20** are not competent for this reaction under the standard conditions (entries 19 and 20).

### Scope of substrates in the asymmetric hydrovinylation of 1,3-dienes

A close examination of the results presented in Table 4 suggests that under optimized conditions the (*Z*)-1,4-HV product **13a** can be prepared in synthetically useful yield and enantioselectivity by asymmetric hydrovinylation of (*E*)-1,3-dienes. The best ligands for this process have been identified as BDPP (**L7**), DIOP (**L8**) and Josiphos 2 (**L18**) and they appear to have much broader applications. A variety of simple and functionalized 1,3-dienes undergo the reaction giving excellent yields and enantioselectivities and the results are documented in Table 5. For simple 1,3-dienes such as **12a–g** (entries 1–13) DIOP and BDPP gave the best results. In most cases, under optimized conditions, nearly quantitative yield of the product (**13**) is obtained in >95% ee with very little if any of contamination by an isomeric product, which is usually the linear adduct **16**. Complexes of Josiphos 2 (**L18**, Fig. 5) are much more active as compared to BDPP (**L7**, Fig. 5) and DIOP (**L8**, Fig. 5), even though the ee's are slightly lower (1 mol% catalyst under otherwise identical conditions entry 6 *vs.* 7 and 8 *vs.* 9). Only in the case of Tangphos ligand **L14** the linear product **16** is formed in significant amount (entry 3). As expected from studies described earlier, this product is also the major one in the cases of 1,3-dienes in conjugation with an aromatic nucleus, *e.g.*, **12g** (entry 13). In this case, up to 55% of linear adduct **16g** is formed with the DIOP complexes. The major chiral product is the 1,2 *E*-adduct **15g**, which is formed in a modest ee of 52%.

The reaction is compatible with an ester group (**12h**) on the diene as shown in entries 14 and 15. Unexpectedly, cobalt complex of the Josiphos 2 ligand (**L18**) failed to affect the reaction of this substrate even with higher catalyst loading (entry 15). The diene containing a benzyl ether (**12i**) undergoes the reaction giving excellent yield and selectivity in the presence of CoCl_2_ complexes of DIOP and BDPP (entries 16–18). *The DIOP complex, especially at low temperature is excellent for this reaction (>99% ee).*

During these studies we also noticed significant difference in the rates of reactions of (*E*)- and (*Z*)-1,3-dienes.57*c* While this difference is hardly perceptible with the achiral Cl_2_Co(dppb) complex (entries 5, 6, 11 and 16 in Table 3), with the Cl_2_Co(DIOP) the (*E*)-isomers react significantly faster, leaving behind essentially unreacted (*Z*)-isomer near the end of the reaction (entries 10, 11, Table 5). In the case of substrate **12e** (entry 11, *E* : *Z* = 47 : 53), the unreacted starting material left behind at the end of the HV reaction (–10 °C, 8 h) using the complex Cl_2_Co(DIOP) is essentially pure (*Z*)-isomer (*E* : *Z* = 1 : 49). Similar behavior is also seen with substrate **12d** (entry 10). Note that while the isomerically pure (*E*)-**12d** leads to 96.1 %ee (entry 8), the *E*/*Z* mixture gives only 74% ee (entry 10), suggesting that the *E*- and *Z*-isomers of a given diene lead to different proportion of the enantiomers in the asymmetric HV.

## Discussion

There are several key features that differentiates this reaction from the more well-studied (P)Ni(allyl)Br-catalysed hydrovinylation of 1,3-dienes^87^ and the hydroalkenylation reaction of 1,3-dienes catalysed by Br_2_Co(P ∼ P)/Zn/ZnI_2_.^88^ Whereas the nickel-catalysed reaction is completely inhibited by chelating ligands,^67^,^89^ the cobalt-catalysed reaction works well with a wide range of chelating ligands, and, by appropriate tuning of these ligands it is possible to obtain synthetically useful yields and selectivities of specific isomers of these hydrovinylation products. For example, cobalt complexes of chelating bis-phosphine ligands with narrow bite angles such as dppm give predominantly the 1,2-adduct (**15**, eqn (7)) whereas complexes of almost all other ligands lead to 1,4-HV leading to the major product **13** in which the initial hydride addition takes place at the terminal carbon and the vinyl group at the C_4_-carbon. The major contamination in these reactions results from a 1,4-addition with the hydride adding to the C_4_-carbon and the vinyl group to C_1_, leading to linear adducts (**16**). When the reaction is carried out at low temperature, the configuration of the internal double bond is almost exclusively (*Z*). When the diene is conjugated with an aromatic nucleus this linear product is formed almost exclusively.

**Scheme 3:**
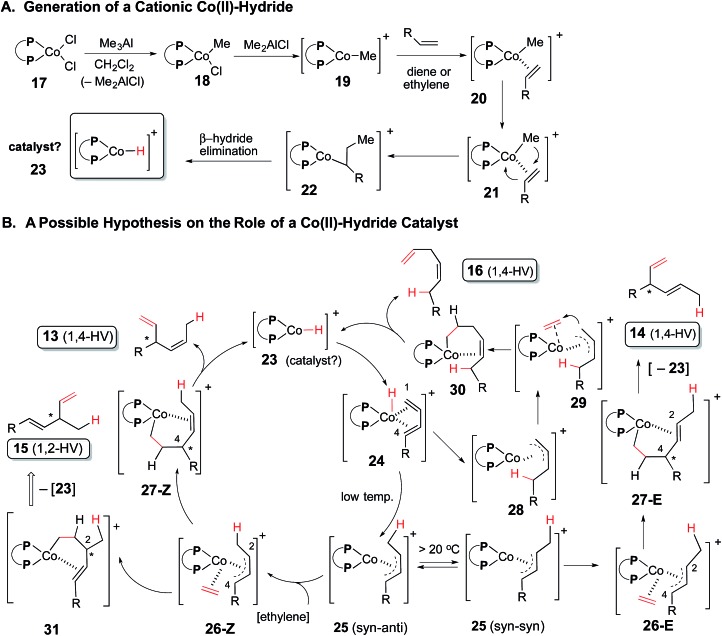
Possible mechanism of Co(ii)-catalysed hydrovinylation of 1,3-dienes.

The remarkable selectivity seen in the HV of 1,3-dienes catalysed by cobalt complexes of dppp- and dppb-analogs (Table 3) stands in stark contrast to the corresponding Ni-catalysed reaction, which leads to an intractable mixture of products. We ascribe the poor results in the Ni-catalysed HV to the inability of this metal (small, limited coordination possibilities) to control the conformational mobility (*s-cis*/*s-trans*) of the 1,3-dienes. Based on several anecdotal observations in the literature^5^,^20^,^56^,^71^,^80^ and analogy to our own previous work on the mechanism of Ni-catalysed hydrovinylation of vinylarenes,^68^ we propose a mechanism for this reaction which is shown in Scheme 3. In this mechanism we hypothesize that a cationic cobalt hydride **23** is the true catalyst in the reaction (panel **A**). This species is formed by a sequence of reactions which start with the reaction of Cl_2_Co(P ∼ P) (**17**) with Me_3_Al to form **18**. Abstraction of a halide by the *in situ* generated Lewis acid (Me_2_AlCl) would give the coordinately unsaturated complex **19**, which facilitates an alkene (ethylene or the 1,3-diene) insertion into the Co–Me bond to generate **22**. A β-hydride elimination from this species would produce the putative catalyst **23**.

The catalyst **23** upon coordination of a diene would form **24** (panel **B**). We believe it is this η^4^-complex that is responsible for the high selectivity of this reaction. Isoelectronic metal complexes containing two chelating phosphines and a hydride are known in the literature^71^ and this is not an unreasonable intermediate for this process. Such a structure would limit the conformation of the coordinated diene to the *s-cis* arrangement, a possibility that does not exist in any corresponding Ni(ii)-species. Two modes of addition of hydride to the intermediate **24** are possible. Addition of hydride to the terminal position (C_1_) would lead to an η^3^-allyl complex **25** (*syn*–*anti*), which after incorporation of ethylene at C_4_ leads to **27**, and from there, to **13** with regeneration of the catalyst **23** by β-hydride elimination. Instead, if the ethylene addition takes space at C_2_ of **26**, **31** will result, which after β-hydride elimination of **23** would lead to the 1,2-HV product **15**. In the second mode, hydride addition takes place at C_4_ of **24** to give **28**, which upon addition of ethylene at C_1_ would lead to the 1,4-linear adduct, **16**.

We expect the initially formed adduct **25** (*syn*–*anti*) to be configurationally stable at low temperature and this would account for the Z-geometry seen in the major product **13** and the *E*-geometry seen in **15**. However, if the *syn*–*anti* isomer of **25** undergoes isomerization to the more stable **25**(*syn*–*syn*), for example at higher temperature, the 1,4-adduct **14** with an *E*-configuration of the double bond will result.^66^,^90^,^91^ This indeed has been observed (compare entries 1–3, or entries 5, 6 in Table 1). Further support for the intermediacy of the η^4^-complex also comes from the enhanced reactivity of the *E*-isomer in a mixture of (*Z*)- and (*E*)-terminal 1,3-dienes (entries 10 and 11 in Table 5). For steric reasons, the formation of the η^4^-complex should be significantly favored for the (*E*)-isomer of the diene, which can adapt an *s-cis* conformation much more easily as compared to the corresponding (*Z*)-diene. This differential reactivity is especially striking in the case of a highly discriminating ligand such as DIOP.

The differences between the hydrovinylation reactions catalysed by Cl_2_Co(P ∼ P)/Me_3_Al and the corresponding reactions carried out under the reducing conditions carried out by Hilt *et al.* [Br_2_Co(P ∼ P)/Zn/ZnI_2_]^46^,^88^ are also noteworthy. The former reaction proceeds in nearly quantitative yields with a broad selection of chelating bis-phosphines from dppm (bite angle 72) to BISBI (bite angle 122) [Table 1]. The reaction under reducing conditions (Zn/ZnI_2_) is limited to cobalt complexes of dppm, dppe and dppp.^81^ We have confirmed these ligand effects in additions of ethylene to 1,3-nonadiene (See Table 2, entries 17, 19, 20). Further, while the more easily reduced complex Br_2_Co(dppp) is catalytically competent upon treatment with Zn/ZnI_2_, the corresponding Cl_2_Co(dppp) returned no products under identical conditions (entry 15 *vs.* entry 20 in Table 2). It is reasonable to assume that under the highly reducing conditions, the reactions probably involve Co(i)-intermediates, where as under our reaction conditions, *viz.*, using [Cl_2_Co(P ∼ P)/Me_3_Al], a cationic cobalt hydride is the active catalyst. All attempts to obtain discernable NMR spectra of any of the putative intermediates under our conditions have been unsuccessful, suggesting involvement of paramagnetic intermediates. On the other hand, (dppe)_2_CoH, an isolable diamagnetic complex (^1^H 16 ppm, ^31^P 69 ppm), described in literature^69^,^70^ is competent to effect the hydrovinylation of (*E*)-1,3-dodecadiene (**12d**), albeit in relatively low yield (10 mol%, CH_2_Cl_2_, 0 °C to rt, 7 h, 43% conversion to **13d** and **16d**). This experiment provides indirect support to the idea of a Co(i) hydride in some of the HV reactions. Other observations in the literature, for example, Et_2_AlCl is a better activator than Et_3_Al for HV reaction of 1,3-butadiene in the presence of isolated (dppe)_2_CoH,^92^ is not also inconsistent with the viability of a cobalt hydride in these reactions. Further studies to clarify the mechanisms of these reactions and to expand their scope will be reported in due course.

## Conclusions

In the presence of bidentate 1,*n*-bis-diphenylphos-phinoalkane-CoCl_2_ complexes {Cl_2_Co[P ∼ P]} and Me_3_Al or methylaluminoxane, acyclic (*E*)-1,3-dienes react with ethylene (1 atmosphere) to give excellent yields of hydrovinylation products. The regioselectivity (1,4- or 1,2-addition) and the configuration (*E*- or *Z*- internal alkene) of the product depend on the nature of the ligand and temperature at which the reaction is carried out. Cobalt(ii)-complexes of 1,1-dipheylphosphinomethane and similar ligands with narrow bite angles give mostly 1,2-addition, retaining the *E*-geometry of the original diene. Complexes of most other ligands at low temperature (–40 °C) give almost exclusively the branched product, (*Z*)-3-alkylhexa-1,4-diene, which arises from a 1,4-hydrovinylation reaction. A minor product is the linear adduct, 6-alkyl-hexa-1,4-diene, also arising from a 1,4-addition of ethylene. As the temperature is increased, a higher proportion of the major 1,4-adduct appears as the (*E*)-isomer. The unexpectedly high selectivity seen in the Co-catalysed reaction as compared to the corresponding Ni-catalysed reaction can be explained by invoking an η^4^-[(diene)[P ∼ P]CoH]^+^-complex and its subsequent reactions. The enhanced reactivity of terminal *E*-1,3-dienes over the corresponding *Z*-dienes can also be explained on the basis of the ease of formation of the η^4^-complex in the former case. The complete lack of reactivity of the X_2_Co(dppb) (X = Cl, Br) complexes in the presence of Zn/ZnI_2_ sets the Me_3_Al-mediated reaction apart from the previously reported hydroalkenylation of dienes. Electron-rich phospholanes, bis-oxazolines and *N*-heterocyclic carbenes appear to be poor ligands for the Co(ii)-catalysed hydrovinylation of 1,3-dienes. An extensive survey of chiral ligands reveals that complexes of DIOP, BDPP and Josiphos ligands are quite effective for these reactions even at –45 °C and enantioselectivities in the range of 90–99% ee can be realized for a variety of 1,3-dienes.

## Supplementary Material

Supplementary information
