# Supplementary material for: Cobalt-catalysed asymmetric hydrovinylation of 1,3-dienes
Source: Chem Sci. 2015 Apr 23;6(7):3994–4008. doi: 10.1039/c5sc00929d (PMC4587399; doi:10.1039/c5sc00929d)
Supplement: Supplementary file 1 [file SC-006-C5SC00929D-s001.pdf]

Electronic Supplementary Material (ESI) for Chemical Science.
This journal is © The Royal Society of Chemistry 2015

SUPPORTING INFORMATION

Cobalt-Catalyzed Asymmetric Hydrovinylation of 1,3-Dienes

Yam N. Timsina, Rakesh K. Sharma,+ T. V. RajanBabu*
Department of Chemistry and Biochemistry, The Ohio State University,
100 West 18th Avenue, Columbus, Ohio 43210, USA

General methods S4

Synthesis of complexes S5

Typical modified procedure for synthesis of cobalt complexes. Synthesis of [*(R,R)*-DIOP]$CoCl_2$
Synthesis of cobalt complexes of *N*-heterocyclic carbene ligand
Synthesis of *tert*-Butyl *bis*-oxazoline complex
Synthesis of [dppe]$FeCl_3$ Complex
Synthesis of methylaluminoxane

Synthesis of Substrates S7

Synthesis of (*E*)-6-benzyloxy-1,3-hexadiene
Synthesis of (*E*)-ethyl-2-methyl-penta-2,4-dienoate
Synthesis of (*E*)-ethyl-hexa-3,5-dienoate and (*E*)-*t*-Bu-hexa-3,5-dienoate
Synthesis of (*Z*/*E*)-1-cyclohexyl-1,3-butadiene and other related dienes

X_2Co(L)-Catalyzed Hydrovinylation of 1,3-Dienes S9

Hydrovinylation of (*E*)-nona-1,3-diene (**12a**) using [DPPB]$CoCl_2$ (Table 1, Entry 9, Typical procedure for ligand screening). S9
Identification of products of hydrovinylation of (*E*)-nona-1,3-diene (**12a**) S10
Procedure for Co-catalyzed hydrovinylation of (*E*)-nona-1,3-diene (**12a**) using [dppb]$CoBr_2$ (Table 1, Ligand Screening, Entry 10). S11
Hydrovinylation of (*E*)-nona-1,3-diene (**12a**) using [dppp]$CoCl_2$ at room temperature (Ligand screening, Table 1, Entry 7). S11
Hydrovinylation of (*E*)-nona-1,3-diene (**12a**) using [dppm]$CoCl_2$ at room temperature (Ligand screening, Table 1, Entry 12). S11
Hydrovinylation of (*E*)-nona-1,3-diene (**12a**) using[BISBI]$CoCl_2$ (Ligand Screening, Table 1, Entry 14). S12

Screening of activators in the Co-Catalyzed hydrovinylation of 1,3-dienes (Table 2) S12

Unsuccessful hydrovinylation of nona-1,3-diene using [dppe]$CoCl_2$ and Mn as promoter (Promoters screening, Table 2, Entry 9). S12
Hydrovinylation of (*E*)-dodeca-1,3-diene using [DPPP]$COBr_2$ and Zn/ZnI_2. (Promoters screening, Table 2, Entry 20) S13
Unsuccessful hydrovinylation of (*E*)-dodeca-1,3-diene using DPPB$CoCl_2$ and diethyl aluminum chloride (Et_2AlCl) as a promoter (Table 2, Entry 13). S13
Unsuccessful hydrovinylation of nona-1,3-diene using [DPPM]$CoCl_2$ and MeMgBr and AgOTf as promoters (Promoters screening, Table 2, Entry 12) S13
Iron-catalyzed hydrovinylation of (*E*)-nona-1,3-diene using[DPPE]$FeCl_3$ (Equation 11): S14

Unsuccessful hydrovinylation of substrate (*E*)-ethyl-2-methylpenta-2,4-dienoate using [dppm]$CoCl_2$ S14
Unsuccessful attempt to dimerize (*E*)-1,3-nonadiene and propylene using [dppe]$CoCl_2$ (Equation 10) S14

Cobalt-Catalyzed Hydrovinylation of 1,3-Dienes. Scope of Substrates (Tables 3 and 5) S16

(*E*)-nona-1,3-diene (12a) S16
Typical procedure for asymmetric Co-catalyzed hydrovinylation reactions of (*E*)-nona-1,3-diene (**12a**) for ligand screening (Table 4, Entries 1-20).
Asymmetric Co-catalyzed hydrovinylation reactions(*E*)-1,3-nonadiene (**12a**) by using (*S,S,R,R*)-Tangphos [(**L14**)$CoCl_2$] (Table 5, Entry 3).
Asymmetric Co-catalyzed hydrovinylation reaction (*E*)-1,3-nonadiene by using 1 mol % Josiphos 2 [(L**18**)$CoCl_2$] (Table 5, Entry 4).

(*E*)-Deca-1,3-diene (12b) S26
Hydrovinylation of (*E*)-deca-1,3-diene using (*R,R*)[DIOP]$CoCl_2$ (Table 5, Entry 5).

(*E*)-Undeca-1,3-diene (12c) S31
Hydrovinylation of (*E*)-undeca-1,3-diene using 1 mol % [**L18** Josiphos-2]$CoCl_2$ Loading (Table 5, Entry 7).

(*E*)-Dodeca-1,3-diene (12d) S39
Hydrovinylation of (*E/Z*)-dodeca-1,3-diene using[DPPB]$CoCl_2$ (Table 3, Entry 5).
Hydrovinylation of (*E*/*Z*)-dodeca-1,3-diene using (*S,S*)[DIOP]$CoCl_2$ (Table 5, Entry 10)

1-Cyclohexyl-1,3-butadiene (12e) S45
Hydrovinylation of (*E/Z*)-1-cyclohexyl-1,3-butadiene using[DPPB]$CoCl_2$ (Table 3, Entry 6):
Unsuccessful hydrovinylation of (only *Z*)-1-cyclohexyl-1,3-butadiene using (*S,S*)[DIOP]$CoCl_2$ and TMA (Table 3, Entry 6, but only *Z*-isomer).

(*E*)-1,3-pentadiene (12f) S55
Hydrovinylation of (*E*)-1,3-pentadiene using [DPPB]$CoCl_2$/Me_3Al at – 10 ^oC (Table 3, Entry 7).
Hydrovinylation of (*E*)-1,3-pentadiene using (*R,R*)-[DIOP]$CoCl_2$ /Me_3Al at – 45 ^oC (Table 5, Entry 12).

(*E*)-1-phenyl-1,3-butadiene (12g) S64
Hydrovinylation of (*E*)-1-phenyl-1,3-butadiene using[dppp]$CoCl_2$ (Table 3, Entry 8)
Hydrovinylation of (*E*)-1-phenyl-1,3-butadiene using [dppm]$CoCl_2$ (Table 3, Entry 10)
Hydrovinylation of (*E*)-1-phenyl-1,3-butadiene using (*SS*)[DIOP]$CoCl_2$ (Table 5, Entry 13)

(*E*)-ethyl hexa-3,5-dienoate (12h) S76

Hydrovinylation of (*E*)-ethyl hexa-3,5-dienoate using [DPPM]$CoCl_2$ (Table 3, Entry 14)

(*E*)-6-benzyloxy-1,3-hexadiene (12i) S82
Hydrovinylation of (*E*)-6-benzyloxy-1,3-hexadiene using [dppb]$CoCl_2$ (Table 3, Entry 15)
Asymmetric hydrovinylation of (*E*)-6-benzyloxy-1,3-hexadiene using (*S,S*)-[DIOP]$CoCl_2$ (Table 5, Entry 17)

(*E/Z*)-hexa-3,5-dienylbenzene (12j) S92
Hydrovinylation of (*E/Z*)-hexa-3,5-dienylbenzene using[dppb]$CoCl_2$ (Table 3, Entry 16)

(*E*)-4-(buta-1,3-dienyl)-*N,N*-dimethylaniline (12k) S97
Hydrovinylation of (*E*)-4-(buta-1,3-dienyl)-*N,N*-dimethylaniline using DPPB$CoCl_2$ (Table 3, Entry 17)

(*E*)-1-phenyl-2-methyl-1,3-butadiene (12l) S103
Hydrovinylation of (*E*)-1-phenyl-2-methyl-1,3-butadiene using [dppm]$CoCl_2$ (Table 3, Entry 18 and 19).

7-Methyl-3-methylene-octa-1,6-diene (*β*-myrcene) (12m) S108
Hydrovinylation of 7-methyl-3-methylene-octa-1,6-diene (*β*-myrcene) using [dppp]$CoCl_2$ (Table 3, Entry 21)

Isoprene (12n) S114
Hydrovinylation of Isoprene using [dppp]$CoCl_2$ (Table 3, Entry 22)

References S119

SUPPORTING INFORMATION

Cobalt-Catalyzed Hydrovinylation of 1,3-Dienes

Yam N. Timsina, Rakesh K. Sharma,[+] T. V. RajanBabu*
Department of Chemistry and Biochemistry, The Ohio State University, 100 West 18th Avenue, Columbus, Ohio 43210, USA
(e-mail: rajanbabu.1@osu.edu)

General methods. Reactions requiring air-sensitive manipulations were conducted under an inert atmosphere of nitrogen or argon by using Schlenk techniques or a Vacuum Atmospheres glovebox. Ethylene (99.5%) was purchased from Matheson Inc., and passed through a column of Drierite® before use. All chemicals obtained from commercial sources were used as received unless otherwise mentioned. All dienes, unless otherwise mentioned, were prepared via Wittig reaction of the corresponding aldehydes with the Wittig reagent generated from methyl triphenylphosphonium bromide and *n*-BuLi in THF. Tetrahydrofuran and diethyl ether were distilled under nitrogen over sodium/benzophenone. Dichloromethane and toluene were purified by distillation from calcium hydride, and subsequently storing over 4 Å molecular sieve. Analytical TLC was performed on E. Merck precoated (0.25 mm) silica gel 60 F254 plates. Flash column chromatography was carried out on silica gel 40 (Scientific Adsorbents Incorporated, Microns Flash). Absence of polymeric impurities was ascertained by NMR of the crude materials. Except for the volatile materials, the isolated yields of the products were not significantly different from the conversions. The percentage compositions for isomeric products was determined by uncalibrated analysis of the areas. This is possible since the response of volatile isomeric hydrocarbons are essentially same in flame ionization detection as determined by comparison of GC-derived compositions with that obtained from ^{1}H NMR in several cases. Gas chromatographic analyses were performed on Agilent gas chromatographs using a HP-1 Methylsilicone column [30 m x 0.250 mm, 0.25 μm film thickness, achiral stationary phase (or ASP) GC column] and an FID detector. Enantiomeric excesses of chiral compounds were determined by chiral stationary phase gas chromatographic (CSP GC) analyses, which were performed on a Hewlett-Packard 5890 with Cyclodex-B capillary GC column (60 m x 0.25 mm, 0.25 μm film thickness) or on an Agilent 7820A using a Cyclosil-B capillary GC column (25 m x 0.25 mm, 0.25 μm film thickness) using helium or hydrogen as a carrier gas. The columns containing Chiral Stationary Phase (CSP) materials were also used for analysis of some of the geometric isomers because they gave better separation. These are indicated under appropriate chromatograms. Where ever appropriate, %ee's were determined from chromatograms where base-line separation of the enantiomers was achieved for an authentic racemic mixture. Limits of detection of the minor enantiomer were established by analyzing mixtures of both enantiomers of known compositions. Optical rotations were recorded on a Rudolph Research Analytical AUTOPOL VI polarimeter in the solvents mentioned. Unless otherwise mentioned, the measurements were done on a filtered (45 micron filter) solution at the sodium line at room temperature. ^{1}H and ^{13}C NMR spectra were recorded either on a spectrometer operating at 500 MHz for ^{1}H and 125 MHz for ^{13}C, or a machine operating at 400 MHz for ^{1}H and 162.02 MHz for ^{31}P. Proton chemical shifts were internally referenced to the residual solvent proton resonance (e.g., $CHCl_3$ at δ 7.26). In several instances where there is a minor deviation, position of a reference peak is included in the data to facilitate assignments. Coupling constants are

reported in Hz. Phosphorous (^{31}P NMR) are reported as δ in units of parts per million (ppm) relative to external H_3PO_4 (δ 0.0).

Synthesis of Cobalt Complexes

Literature methods[1] were used for preparation of complexes Co(dppm)Cl_2 and Co(dppp)Cl_2. For the preparation of Co(dppb)Cl_2, [(*RR*)-(–)(DIOP)CoCl_2], [(*SS*)-(+)-(DIOP)CoCl_2] and [(*SS*)-(–)-(BDPP)CoCl_2] modified procedures (see below) were used. Solid-state structures of these two complexes have been reported in our previous publication.[2]

Typical modified procedure for synthesis of cobalt complexes: [*(R,R)*-DIOP]CoCl_2. A 25-mL dry round bottom flask loaded with anhydrous CoCl_2 (10.9 mg, 0.0839 mmol) was charged with THF (5 mL). On stirring at room temperature for 15 min, a clear deep blue solution formed. A solution of (*R,R*)-DIOP (41.8 mg, 0.0839 mmol) in dry and degassed ether (5 mL) was added dropwise to yield a blue turbid solution. After stirring at room temperature for 6 h, 20 mL deoxygenated hexane was added in one portion to get a blue solid. The resulting solid was washed with diethyl ether and hexane (1:1) mixture (3 X 5 mL) to remove any unreacted DIOP. Further purification was accomplished by crystallization of the Co(II)-complex from a saturated $CHCl_3$ solution by slow vapor diffusion of pentane at room temperature. The solid state structure determined by X-ray crystallography has been reported.[2]

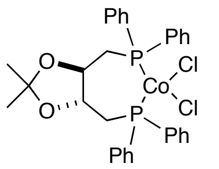

(4*R*,5*R*)-(-)-O-Isopropylidene-2,3-dihydroxy-1,4-bis(diphenylphosphino)-butane dichloro cobalt(II): (–)-[(*RR*)-(–)-(DIOP)CoCl_2]. Yield 94%. Elemental analysis calculated for $C_{31}H_{32}Cl_2CoO_2P_2$: C, 59.25; H, 5.13. Found: C, 56.60; H, 5.09. $[\alpha]^{25}_{405}$ ($CHCl_3$, *c* 1.25) = – 93.7

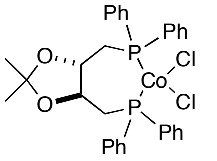

(4*S*,5*S*)-(+)-O-Isopropylidene-2,3-dihydroxy-1,4-bis(diphenylphosphino)-butane dichloro cobalt(II): (+)-[(*SS*)-(+)-(DIOP)CoCl_2]; Yield 92%. Elemental analysis calculated for $C_{31}H_{32}Cl_2CoO_2P_2$: C, 59.25; H, 5.13. Found C, 57.05, H, 5.12. $[\alpha]^{25}_{405}$ ($CHCl_3$, *c* 1.25) = + 88.8

[*(S,S)*-BDPP]CoCl_2. Cobalt chloride (50.5 mg, 0.390 mmol) was added to a previously flame-dried 50-mL round two-necked bottom flask fitted with a flow control gas inlet and magnetic stir-bar loaded in a glove box under nitrogen. Outside the box, the nitrogen atmosphere was removed and the flask purged with dry argon. Freshly distilled, degassed THF (5 mL) was added, and upon stirring at room temperature for 15 min, a clear deep blue solution formed. A solution of (*S,S*)-BDPP (181 mg, 0.410 mmol) in freshly distilled, degassed ether (5 mL) was added dropwise to yield a blue turbid solution. After stirring at room temperature for 12 h, 20 mL freshly distilled, degassed hexane was added in one portion to yield a blue precipitate. The resulting precipitate was filtered under argon atmosphere, and washed with diethyl ether and hexane (1:1) mixture (3 X 5 mL) to remove any unreacted (*SS*)-BDPP, resulting in quantitative yield of a light blue solid, which was used with no further purification. A sample was recrystallized from saturated $CHCl_3$ solution by slow vapor diffusion of pentane at room temperature. The solid-state structure determined by X-ray crystallography has been reported.[2]

(*2S,4S*)-(-)-2,4-Bis(diphenylphosphino)-pentane dichlorocobalt(II): (+)-[(*SS*)-(–)-(BDPP)CoCl_2]. Yield 96%. Elemental analysis calculated for $C_{29}H_{30}Cl_2CoP_2$: C, 61.07; H, 5.30. Found C, 60.69; H, 5.28.

$[\alpha]^{25}_{405}$ ($CHCl_3$, *c* 1.00) = + 271.0; $[\alpha]^{25}_{436}$ ($CHCl_3$, *c* 1.00) = + 132.0; $[\alpha]^{25}_{546}$ ($CHCl_3$, *c* 1.00) = + 164.0

Cobalt(II)-complexes of ligands listed in Table 1 and Figure 5 were similarly prepared and used for hydrovinylation under conditions listed in various tables.

Synthesis of cobalt complexes of *N*-heterocyclic carbene ligand (Figure 3). In a typical procedure the bromide salt of a carbene precursor (195 mg, 0.46 mmol, 1 equiv.) and potassium *tert*-butoxide (306 mg, 2.73 mmol, 6 equiv.) were added to a previously oven-dried 20-mL round bottom flask with magnetic stir-bar loaded in a glove box under nitrogen. Freshly distilled THF (5 mL) was added and stirred at room temperature for 36 hours under nitrogen in glove box. The reaction solution was passed through a pad of celite in dry box and the solvent was evaporated under vacuum to get free carbene. Anhydrous $CoCl_2$ (48 mg, 0.37 mmol, 0.95 equiv.) was added to a previously oven-dried 20-mL round bottom flask with sidearm fitted with a flow control gas inlet and magnetic stir-bar loaded in a glove box under nitrogen. The flask was removed from the drybox and the nitrogen atmosphere was removed and the flask purged with dry argon. Freshly distilled, degassed THF (4 mL) was added, and upon stirring at room temperature for 15 min, a clear deep blue solution formed. A solution of free carbene (104 mg, 0.39 mmol, 1 equiv.) in freshly distilled degassed THF (2 mL) was added dropwise to yield a blue solution. After stirring at room temperature for 32 h, 10 mL freshly distilled, degassed hexane was added in one portion to yield a blue precipitate. The resulting precipitate was filtered under argon atmosphere, and washed with diethyl ether and hexane (1:1) mixture (3 x 10 mL) to remove any unreacted carbene, resulting in quantitative yield of a light blue solid, which was used in hydrovinylation with no further purification.

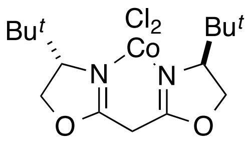

Synthesis of $CoCl_2$[*tert*-butyl bis-oxazoline] complex. Similar procedure was used for synthesis of cobalt complexes from dinitrogen based chelating ligands (–)-sparteine and the bis-oxazoline complexes (Figure 3). As a typical procedure, anhydrous $CoCl_2$ (23 mg, 0.18 mmol, 1 equiv.) was added to a previously oven-dried 20-mL round bottom flask with sidearm fitted with a flow control gas inlet under nitrogen. The nitrogen atmosphere was removed and the flask purged with dry argon. Freshly distilled, degassed THF (3 mL) was added, and upon stirring at room temperature for 20 min, a clear deep blue solution formed. A solution of the *t*-butyl bis-oxazoline ligand (51 mg, 0.19 mmol, 1.05 equiv.) in freshly distilled degassed THF (3 mL) was added dropwise to yield a blue solution. After stirring at room temperature for 36 h, 15 mL freshly distilled, degassed hexane was added in one portion to yield a blue precipitate. The resulting precipitate was filtered under argon atmosphere, and washed with diethyl ether and hexane (1:1) mixture (3 x 15 mL) to remove any unreacted ligand, resulting in quantitative yield of a light blue solid, which was used for hydrovinylation of 1,3-dienes with no further purification.

Synthesis of [dppe]$FeCl_3$ complex. As a typical procedure, anhydrous $FeCl_3$ (45 mg, 0.28 mmol, 1 equiv.) was added to a previously oven-dried 20-mL round bottom flask with sidearm fitted with a flow control gas inlet under nitrogen. Freshly distilled, degassed THF (2 mL) was added, and upon stirring at room temperature for 20 min, a black-brown solution formed. A solution of DPPE (47 mg, 0.29 mmol, 1.05 equiv.) in freshly distilled degassed THF (3 mL) was added dropwise to yield a light pink solution. After stirring at room temperature for 24 h, 15 mL

freshly distilled, degassed hexane was added in one portion to yield a blue precipitate. The resulting precipitate was filtered under argon atmosphere, and washed with diethyl ether and hexane (1:1) mixture (3 x 15 mL) to remove any unreacted dppe, resulting in good yield of a light blue solid (130 mg, 84%), which was used for hydrovinylation of 1,3-dienes with no further purification.

Synthesis of Methylaluminoxane.[3] A flame-dried 250 mL three-necked round-bottom flask equipped with a septum, flow control gas inlet, temperature probe, and magnetic stir-bar was purged with argon and charged with 3.85 g $Al_2(SO_4)_3 16\ H_2O$, 15 mL dry, distilled toluene and chilled to –10 °C using a salt/ice bath. To this slurry was added 20 mL 2 M trimethylaluminum solution in toluene stored at 0 °C dropwise via syringe with vigorous stirring. The ice bath was removed and the solution allowed to warm to room temperature slowly, and then heated to 30 °C for 4 hrs. After the first heating period, the mixture was then slowly brought to 50 °C and stirred for 8 hrs overnight. A Schlenk filter (12" column fitted with two male ground glass joints and a microporous fret in the center) and a 250 mL single-necked round-bottom were flamed dried and attached to the 250 mL three-necked flask. The solution was filtered to remove the solids and the resulting toluene filtrate was distilled to remove the toluene to give a white glassy solid that was harvested in an inert atmosphere glovebox to get the product as a white solid 0.610 g (26% yield)

Synthesis of Substrates

Synthesis of (*E*)-6-benzyloxy-1,3-hexadiene (12i). A 250 mL flask with sidearm equipped with a magnetic stirring bar, purged with nitrogen, was charged with methyltriphenylphosphonium bromide (6.31 g, 17.66 mmol, 1.1 equiv.) and anhydrous THF (25.0 mL). *n*-BuLi (2.5 M in hexane, 7.1 mL, 17.66 mmol, 1.1 equiv.) was added dropwise via syringe at 0 ^{0}C and the reaction mixture was allowed to stir at 0 ^{0}C for 2 h. A solution of (*E*)-5-(benzyloxy)pent-2-enal (3.0 g, 15.77 mmol) dissolved in anhydrous THF was added dropwise and the reaction mixture was allowed to stir at ambient temperature for 12 h. The reaction mixture was diluted with pentane, filtered over Celite® and the filtrate was concentrated and purified via flash column chromatography (pentane-ether, 20:1) to yield the 1,3 diene (2.04 g, 69%) as a *E*-1,3-diene. ^{1}H NMR and ^{13}C NMR agreed with published data.[2] ^{1}H NMR ($CDCl_3$, 400 MHz): 2.48 (q, J = 4 Hz, 2 H), 3.59 (t, J = 8 Hz, 2 H), 4.58 (s, 2 H), 5.05 (d, J = 8 Hz, 1 H), 5.17 (d, J = 16 Hz, 1 H), 5.75 – 5.82 (m, 1 H), 6.15 – 6. 22 (m, 1 H), 6.33 – 6.43 (m, 1 H), 7.31 – 7.43 (m, 5 H, aromatic). Gas Chromatography: CSP GC (Cyclosil, OT 150 ^{0}C/ isothermal) R_T for diene = 12.357 min. Methyl silicone SP GC (OT 150 ^{0}C/ isothermal) R_T for diene product = 10.492 min.

All the (*E*)-1,3-dienes as well as like **12a**, **12b, 12c, 12d,** and **12e** were prepared by the literature methods of Wittig olefination using the corresponding aldehyde. Dienes like β-myrcene (**12m**), isoprene (**12n**) and (*E*)-1,3-pentadiene (**12f**) were purchased directly and used after proper distillation.

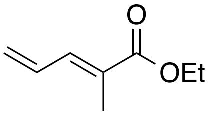

Synthesis of (*E*)-ethyl-2-methyl-penta-2,4-dienoate. A 250 mL flask with sidearm equipped with a magnetic stirring bar, purged with nitrogen, was charged with (1-ethoxycarbonylethyl)triphenylphosphonium bromide (5.0 g, 11.28 mmol, 1.0 equiv.) and anhydrous CH_2Cl_2 (20.0 mL). Potassium

hydroxide (KOH) (0.8 gm, 13.54 mmol, 1.2 equiv.) as aqueous solution was added dropwise and stirred for 3 hrs at room temperature. A solution of acrolein (0.8 g, 13.54 mmol) dissolved in anhydrous CH_2Cl_2 was added dropwise and the reaction mixture was refluxed for 3 hrs and stirred 24 hrs at room temperature. The reaction mixture was diluted with petroleum ether, filtered over Celite® and the filtrate was concentrated and purified via flash column chromatography (Hex-EtOAc, 7:1) to yield the 1,3 diene (0.6 g, 38%) as a *E*-1,3-diene. ^{1}H NMR and ^{13}C NMR agreed with published data.[5-7] ^{1}H NMR ($CDCl_3$, 400 MHz): 1.29 (t, J = 7.2 Hz, 3 H), 1.93 (s, 3 H), 4.20 (q, J = 7.2 Hz, 2 H), 5.42 (d, J = 8 Hz, 1 H), 5.53 (d, J = 16 Hz, 1 H), 6.59 – 6.68 (m, 1 H), 7.13 (d, J = 12 Hz, 1 H). CSP GC (OT 80 ^{0}C/ isothermal, H_2 carrier gas) Conditions: R_t for diene product = 14.28 min.

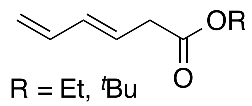

R = Et, ^{t}Bu

Synthesis of (*E*)-ethyl-hexa-3,5-dienoate and (*E*)-*t*-Bu-hexa-3,5-dienoate. A 250 mL flask with condenser equipped with a magnetic stirring bar, purged with nitrogen, was charged with hexa-2,4-dienoic acid (12.0 g, 107 mmol, 1.0 equiv.) and 5 drops of DMF and 3 boiling chips were added. Thionyl chloride (19 g, 161 mmol, 1.5 mmol) was added from the top of the condenser dropwise and refluxed 30 minutes. HCl and SO_2 gas were trapped by dil. NaOH solution through out the reflux period and the reaction was stopped when there was no more evolution of HCl and SO_2 gas through the NaOH solution. Hexa-2,4-dienoyl chloride (10 g, 71%) was distilled out from the same round bottom flask and directly used for the next set up reaction for diene synthesis. Freshly distilled ethyl alcohol (5.6 g, 123 mmol, 2 equiv.) and freshly distilled triethyl amine (12.4 g, 122.54 mmol, 2 equiv.) were mixed in 120 mL CH_2Cl_2 and chilled under dry ice/acetone. Hexa-2,4-dienoyl chloride (8 g, 61.27 mmol, 1 equiv.) was added dropwise via syringe. After 1 hr, a thick white precipitate appeared and the reaction was allowed to warm to room temperature and left overnight. Around 200 mL water was added and partitioned between water and DCM. The organic layer was washed (2 X 10% H_2SO_4, 1 X saturated $NaHCO_3$, 1 x H_2O, 1 X brine) and dried with $MgSO_4$ and solvent was evaporated to get crude diene. A few crystals of BHT were added to the crude product which was then distilled to afford ethyl-3,5-hexadienoate (5.1 g, 59%). ^{1}H NMR and ^{13}C NMR agreed with published data.[8-9]

Same procedure was applied to synthesize (*E*)-*t*-Bu-3,5-hexadienoate by using *t*-butyl alcohol instead of ethyl alcohol.

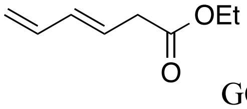

^{1}H NMR ($CDCl_3$, 400 MHz): 1.21 (t, J = 7.2 Hz, 3 H), 3.07 (d, J = 6.4 Hz, 2 H), 4.12 (q, J = 4 Hz, 2 H), 5.02 (d, J = 10.4 Hz, 1 H), 5.12 (d, J = 16.4 Hz, 1 H), 5.71 – 5.78 (m, 1 H), 6.06 – 6.13 (m,1 H), 6.25 – 6.34 (m, 1 H). CSP GC (OT 90 ^{0}C/ isothermal, cyclodex-B, He carrier gas) R_T for diene product = 38.27 min.

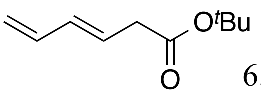

^{1}H NMR ($CDCl_3$, 400 MHz): 1.59 (s, 9 H), 3.16 (d, J = 8 Hz, 2 H), 5.18 (d, J = 10.4 Hz, 1 H), 5.28 (d, J = 17.2 Hz, 1 H), 5.87 – 5.94 (m, 1 H), 6.22 – 6.28 (m, 1 H), 6.42 – 6.51 (m,1 H). ^{13}C NMR ($CDCl_3$, 125.02 MHz): 28.30, 39.45, 80.99, 116.76, 126.63, 134.16, 136.79, 171.04. CSP GC (OT 100 ^{0}C/ isothermal, cyclosil, H_2 carrier gas) R_T for diene product = 12.09 min.

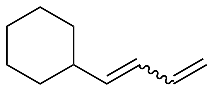

(*E* + Z 47:53)

Synthesis of (*Z*/*E*)-1-cyclohexyl-1,3-butadiene and other related dienes. As a typical procedure to make (*E*/*Z*)-mixture of 1-cyclohexyl-1,3-butadiene, a 250 mL flask with sidearm equipped with a magnetic stirring bar, purged with nitrogen, was charged with allyltriphenylphosphonium bromide (7.0 g, 18.0 mmol, 1.5 equiv.) and anhydrous THF (30.0 mL). Base *n*-BuLi (2.5 M in hexane, 6.5 mL, 16.2 mmol, 0.9 equiv.) was added dropwise via syringe at 0 ^{0}C and the reaction mixture was allowed to stir at 0 ^{o}C for 1 h. A solution of cyclohexanecarboxaldehyde (1.82 g, 16.2 mmol) dissolved in anhydrous THF was added dropwise and the reaction mixture was allowed to stir at ambient temperature for 12 h. The reaction mixture was diluted with pentane, filtered over Celite® and the filtrate was concentrated and purified via flash column chromatography (pentane-ether, 20:1) to yield the 1,3 diene (1.30 g, 59%) as a mixture of *Z*- and *E*-isomers (53:47, by GC). ^{1}H NMR and ^{13}C NMR agreed with published data.[10-11] ^{1}H NMR ($CDCl_3$, 400 MHz): δ 1.05-1.30 (m, 11 H, both *E* and *Z*) 1.61-1.72 (m, 9 H, both *E* and *Z*), 1.96 – 1.98 (m, 1 H, *E* only), 2.41 – 2.44 (m, 1 H, *Z* only), 4.93 (d, J = 9 Hz, 1 H, *E* only), 5.03 – 5.17 (m, 3 H, overlapped *E* and *Z*), 5.29 (t, J = 10 Hz, 1 H, *Z* only), 5.64 (dd, J = 5, 16 Hz, 1 H, *E* only), 5.86 (t, J = 11 Hz, 1 H, *Z* only), 5.96 – 6.02 (m, 1 H, *E* only), 6.23 – 6.33 (m, 1 H, *E* only), 6.58 – 6.68 (m, 1 H, *Z* only). ^{13}C NMR ($CDCl_3$, 125.72 MHz): 26.07, 26.22, 26.38, 32.97, 33.45, 37.07, 40.84, (both *E* and *Z* overlapped), 114.90 (*E*), 116.88 (*Z*), 127.47 (*Z*), 128.53 (*E*), 132.81 (*Z*), 137.88 (*E*), 139.15 (*Z*), 141.51 (*E*).
Gas Chromatography: (Cyclosil, temp. 80 ^{o}C / isothermal, H_2 carrier gas) starting material R_T = 18.63 min for *Z*-diene and 26.243 min for *E* diene.

Similar procedure was used for the synthesis of (*E*/*Z*)-mixture of 1,3-dienes like (*E*/*Z*) dodeca-1,3-diene (**12d**), (*E*/*Z*)1-phenyl-1,3-butadiene (**12g**) and (*E*/*Z*) hexa-3,5-dinylbenzene (**12j**) using corresponding aldehyde and allyltriphenylphosphonium bromide.

X_2Co(L)-Catalyzed Hydrovinylation of 1,3-Dienes

Hydrovinylation of (*E*)-nona-1,3-diene using[DPPB]$CoCl_2$ (Table 1, Entry 9, Typical procedure for ligand screening). To an oven dried round bottom flask with a sidearm, was added [DPPB]$CoCl_2$ (35 mg, 0.063 mmol) dissolved in dichloromethane (1 mL) at room temperature. Trimethylaluminium as a 2M solution in toluene (0.17 mL, 0.32 mmol) was added dropwise and the color of the solution changed from deep blue to red brown with the formation of white fumes over the solution. After 2 to 3 minutes, the reaction vessel was carefully evacuated and then refilled with ethylene from a balloon. The reaction vessel was kept at 0 ^{o}C temperature and (*E*)-nona-1,3-diene (78 mg, 0.63 mmol) in dichloromethane (0.5 mL) solution added under ethylene and the mixture was stirred for 0.5 h. The ethylene balloon was removed and 0.1 mL of methanol was added to the flask. The reaction solution was diluted with pentane (3 mL) and subsequently passed through a silica plug. The plug was washed with pentane (3 x 5 mL). Removal of solvent yielded the product as colorless oil (96% 1,4-*Z* (**13a**) and 1,4-linear (**16a**) by GC and NMR analysis.

All the reactions in Table 1 were performed in similar fashion using the conditions described in the Table 1 using Co(II)-complexes prepared as described before. Some typical procedures are described below after details of the identification of the isomeric products from **12a**.

Identification of Products of Hydrovinylation of (*E*)-nona-1,3-diene (12a)

[The following data extracted from ref. 2, which also contains details of gas chromatographic analysis including chiral stationary phase separations of various isomers. The reported ee's were determined from these gas chromatograms.]

Hydrovinylation of (*E*)-nona-1,3-diene (13a) using [dppb]$CoCl_2$/Me_3Al at -10 ºC (Entry 9, Table 1), or using [DIOP]$CoCl_2$ at - 45 ºC (Entry 8, Table 4)

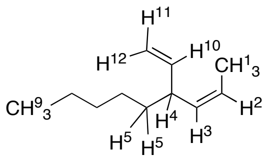

(*Z*)-4-Vinylnon-2-ene (13a): ^{1}H NMR (500 MHz, $CDCl_3$): δ 0.869 (t, J = 7 Hz, 3 H, H^9), 1.21-1.35 (m, 7 H), 1.38-1.45 (m, 1 H) 1.21-1.45 total 8 H, 1.622 (dd, J = 6.5 Hz, 2 Hz, 3 H, H^1), 3.011 (m, 1 H, H^4), 4.929 (ddd, J = 10 Hz, 1.5 Hz, 1.5 Hz, 1 H, H^{11}), 4.982 (ddd, J = 17.5 Hz, 1.5 Hz, 1.5 Hz, 1 H, H^{12}), 5.216 (ddq, 10 Hz, 10 Hz, 1.5 Hz, H^3), 5.495 (dq, 10.5 Hz, 6.5 Hz, 1 Hz, H^2), 5.709 (ddd, 17 Hz, 10 Hz, 7 Hz, 1H, H^{10}). Assignments and coupling confirmed by COSY and NOESY (CH^1_3–> H^4).
^{13}C NMR ($CDCl_3$): 13.02, 14.07 22.63, 26.79, 31.90, 35.24, 41.26, 112.87, 123.78, 133.23, 141.87. The peak at δ 14.07 (due to the vinyl methyl carbon is at a higher field compared to the corresponding peak in the corresponding (*E*)-isomer , which appears at δ 17.96, see below).
IR (neat cm^{-1}): 3079 (w), 3111 (m), 2959 (s), 2927 (s), 2858 (s), 1636 (m), 992 (w), 909 (m), 724 (m)- 724 cm^{-1} is characteristic of a (*Z*)-alkene. Absence of peaks ~ 960-970 cm^{-1} indicates absence of the (*E*)-isomer, **14a**.
ESI-MS; m/z 191.1 [M+K]; mass calculated for $C_{11}H_{20}K$, 191.1.
Product from (*RR*)-(-)-DIOP $[\alpha]^{25}_D$ = – 20.9 (hexane, *c* 1.15)

Gas Chromatography: (Polymethyldisiloxane): 80 ºC R_T **13a**: 21.169 min.; **14a**: 21.93min.; **15a** 22.77 min.; CSP GC (Cyclodex B) 60 ºC **13a:** R_T = 26.07 min. (*S*), 27.25 (*R*); **16a** 44.79 min.

Isomeric hydrovinylation products obtained from (*E*)-nona-1,3-diene (13a) using [dppm]$CoCl_2$/Me_3Al (Entries 12 and 13, Table 1)

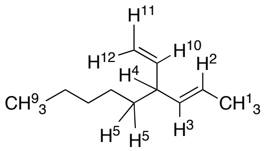

(*E*)-4-vinylnon-2-ene (14a): ^{1}H NMR ($CDCl_3$): δ 0.85-0.91 (m, 3 H, H^9), 1.21-1.41 (m, H^5, H^6, H^7, H^8), 1.669 (dd, J = 6 Hz, 0.5 Hz, 3 H, H^1), 2.619 (pent, J = 7.5 Hz, 1 H, H^4), 4.931 (dm, J = 10 Hz, 1 H, H^{11}), 4.977 (dm, J = 17.5 Hz, 1 H, H^{12}), 5.29-5.45 (m, 2H, H^2, H^3), 5.706 (ddd, J = 17.5, 10, 7.5 Hz, 1 H, H^{10}). Chemical shifts and coupling constants assigned by COSY and double irradiation experiments.
^{13}C NMR ($CDCl_3$): δ 14.07, 17.96 (vinyl Me), 22.63, 26.84, 31.88, 34.86, 46.88, 113.24, 124.53, 134.17, 142.35. The peak at δ17.96 (due to vinyl methyl carbon is at a lower field compared to the corresponding peak in the (*Z*)-isomer **13a**, which appears at δ 17.07, see under **13a**, the previous compound.)

Gas chromatography: (polydimethylsiloxane column): see previous experiment.

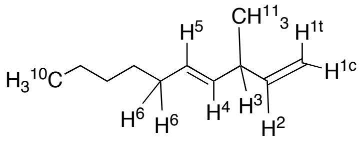

(*E*)-3-methyldeca-1,4-diene (15a). ^{1}H NMR ($CDCl_3$): 0.85-0.91 (m, 3 H, H^{10}), 1.075 (d. J = 6.5 Hz, 1 H, H^{11}), 1.20-1.40 (m, H^7,

H^8, H^9), 1.990 (ddd, J = 6.5, 6.5, 6.5 Hz, 2 H, H^6), 2.807 (ddq, J = 6.0, 6.0, 6.0 Hz, H^3), 4.931 (dm, J = 10 Hz, 1H, H^{1c}), 4.977 (dm, J = 17.5 Hz, 1 H, H^{1t}), 5.352 (dd, J = 15.5, 6.5 Hz, 1 H, H^4), 5.416 (dt, J = 15.5, 6.5, 1 H, H^5), 5.791 (ddd, J = 17, 10.5, 6.5 Hz, 1 H, H^2). Chemical shifts and coupling constants assigned by COSY and double irradiation experiments.
^{13}C NMR ($CDCl_3$): 14.04, 20.00, 22.54, 29.23, 31.41, 32.55, 40.31, 112.37, 129.44, 133.86, 143.40.

Gas chromatography: (polydimethylsiloxane column) : see under **13a.**

Procedure for Co-catalyzed hydrovinylation of (*E*)-nona-1,3-diene using [dppb]$CoBr_2$ (Table 1, Ligand Screening, Entry 10). To an oven dried round-bottom-flask with a sidearm, was added [dppb]$CoBr_2$ (16 mg, 0.025 mmol) and under argon it was dissolved in dichloromethane (1 mL) at room temperature. Trimethylaluminium as a 2M solution in toluene (0.12 mL, 0.24 mmol) was added dropwise and the color of the solution changed from blue to red brown with the formation of white fumes over the solution. After 2 to 3 minutes, the reaction vessel was carefully evacuated and then refilled with ethylene from a balloon. The reaction vessel was cooled to -20 ^oC and (*E*)-nona-1,3-diene (30 mg, 0.24 mmol) in a dichloromethane (1 mL) added under ethylene and the mixture was stirred for 20 h. The progress of the reaction was monitored via gas chromatography until completion. The ethylene balloon was removed and 0.1 mL methanol was added to the flask. The solution was diluted with pentane (5 mL) and warmed to room temperature and subsequently passed through a silica plug. The plug was washed with pentane (3 x 5 mL). Removal of solvent yielded the product as colorless oil as 94% (Z)-1,4 HV product (**13a**) and 5% (*Z*)-1,4-linear dimerized HV product (**16a**).

Hydrovinylation of (*E*)-nona-1,3-diene using[dppp]$CoCl_2$ at room temperature (Ligand screening, Table 1, Entry 7). To an oven-dried round-bottom flask with a sidearm, was added [dppp]$CoCl_2$ (11 mg, 0.020 mmol) under argon and it was dissolved in dichloromethane (1 mL) at room temperature. Trimethylaluminum solution (2M) in toluene (0.03 mL, 0.060 mmol) was added dropwise as color of the solution quickly changed from deep blue to brown with the formation of white fumes over the solution. When all the fumes disappeared in about 5 minutes, the reaction vessel was carefully evacuated and then refilled with ethylene from a balloon, a vigorous reaction with evolution of fumes was observed. When this evolution stopped, (*E*)-nona-1,3-diene (25 mg, 0.20 mmol) was added under ethylene at room temperature and the mixture was stirred for 1 h until the completion of reaction monitored via gas chromatography. The ethylene balloon was removed and 0.1 mL of methanol was introduced into the flask and stirring was continued for 5 minutes. The solution was subsequently passed through a silica plug. The plug was washed with pentane (3 x 10 mL). Concentration and removal of solvent yielded the product as a colorless oil and isomeric compositions were determined by gas chromatography and NMR spectroscopy. The ratios of products are indicated in the table.

Hydrovinylation of (*E*)-nona-1,3-diene using[dppm]$CoCl_2$ at room temperature (Ligand screening, Table 1, Entry 12). To an oven-dried round-bottom flask with a sidearm, was added [dppm]$CoCl_2$ (42 mg, 0.082 mmol) under argon and it was dissolved in dichloromethane (1.5 mL) at room temperature. Trimethylaluminum solution (2M) in toluene (0.13 mL, 0.25 mmol) was added dropwise as color of the solution quickly changed from bluish brown to red brown with the formation of white fumes over the solution. When all the fumes disappeared in around

5 minutes, the reaction vessel was carefully evacuated and then refilled with ethylene from a balloon, a vigorous reaction with evolution of fumes was observed. When this evolution stopped, (*E*)-nona-1,3-diene (102 mg, 0.82 mmol) in dichloromethane (1 mL) was added under ethylene at room temperature and the mixture was stirred for 2 h. The progress of the reaction was monitored via gas chromatography intermittently. After 2 hours, the ethylene balloon was removed and 0.1 mL of methanol was introduced into the flask and stirring was continued for 5 minutes. The solution was subsequently passed through a silica plug. The plug was washed with pentane (3 x 10 mL). Concentration and removal of last traces of solvent yielded the product as a colorless oil and isomeric compositions were determined by gas chromatography and NMR spectroscopy (Table 1, entry 12).

Hydrovinylation of (*E*)-nona-1,3-diene using[BISBI]$CoCl_2$ (Ligand Screening, Table 1, Entry 14). To an oven-dried round-bottom flask with a sidearm, was added [BISBI]$CoCl_2$ (20 mg, 0.029 mmol) under argon and it was dissolved in dichloromethane (1 mL) at room temperature. Trimethylaluminum solution (2M) in toluene (0.15 mL, 0.29 mmol) was added dropwise as color of the solution quickly changed from blue to red brown with the formation of white fumes over the solution. When all the fumes disappeared in around 5 minutes, the reaction vessel was carefully evacuated and then refilled with ethylene from a balloon, a vigorous reaction with evolution of fumes was observed. When this evolution stopped, (*E*)-nona-1,3-diene (36 mg, 0.29 mmol) in dichloromethane (1 mL) was added under ethylene at -12 ^oC temperature and the mixture was stirred for 6 h. The progress of the reaction was monitored via gas chromatography intermittently. After 6 hours, the ethylene balloon was removed and 0.1 mL of methanol was introduced into the flask and stirring was continued for 5 minutes. The solution was subsequently passed through a silica plug. The plug was washed with pentane (3 x 10 mL). Concentration and removal of solvent yielded the product as a colorless oil and isomeric compositions [**13a**, 1,4-Z HV (65%) and **15a**, 1,2-E HV (34%]) were determined by gas chromatography and NMR spectroscopy.

Screening of activators in the Co-Catalyzed hydrovinylation of 1,3-dienes (Table 2)

The typical procedure described above (Hydrovinylation of (*E*)-nona-1,3-diene using[DPPB]$CoCl_2$ (Table 1, Entry 9) was used in screening various promoters for the reaction (Table 2).

The solid activators like Mn, InI_3, and Zn/ZnI_2 were added at the same time with cobalt complexes in the round bottom flask with side-arm in the nitrogen atmosphere glove box. Liquid activators like Me_3B, Et_3B, Ph_3B, *i*-BuAlH, $LiEt_3BH$ (in THF), Et_2AlOEt, Et_2AlCl, $EtAlCl_2$, PhMgBr, were added in the same way as trimethylaluminium (TMA solution in toluene) to the solution of cobalt complexes via syringe.

Typical experiments are described below:

Unsuccessful hydrovinylation of nona-1,3-diene using [dppe]$CoCl_2$ and Mn as promoter (Promoters screening, Table 2, Entry 9). As a typical procedure, to an oven dried round-bottom-flask with a sidearm, was added DPPE$CoCl_2$ (21 mg, 0.040 mmol) and Mn (22 mg, 0.40 mmol) under argon and was dissolved in dichloromethane (1 mL) at room temperature. After 30 minutes, the reaction vessel was carefully evacuated and then refilled with ethylene from a balloon. The reaction vessel was kept in room temperature and the nona-1,3-diene (50 mg, 0.40

mmol) in dichloromethane (1 mL) added under ethylene and the mixture was stirred for 14 h at room temperature intermittently monitoring the progress of reaction by gas chromatography. The ethylene balloon was removed and 0.1 mL methanol was added to the flask. The solution was diluted with pentane (5 mL) and subsequently passed through a silica plug. The plug was washed with pentane (3 X 6 mL). Removal of solvent recovered the starting material back. GC and NMR analysis showed that the product was not formed at all.

Hydrovinylation of (*E*)-dodeca-1,3-diene using [DPPP]COBr$_2$ and Zn/ZnI$_2$. (Promoters screening, Table 2, Entry 20) To an oven dried round-bottom-flask with a sidearm, was added [DPPP]CoBr$_2$ (20 mg, 0.032 mmol), Zn dust (4.2 mg, 0.064 mmol) and ZnI$_2$ (20 mg, 0.064 mmol) under argon and was dissolved in dichloromethane (2 mL) at room temperature. The solution was stirred for at least 30 minutes at room temperature, and then refilled with ethylene from a balloon. The reaction vessel was cooled to 0 ^oC and (*E*)-dodeca-1,3-diene (53 mg, 0.32 mmol) was added under ethylene. After 1 hour, first aliquot of the reaction mixture was taken out via syringe and flush through a small pad of silica and was analyzed by GC. Reaction was monitored by GC analysis until completion. Finally after stirring the reaction mixture for 4 h at ambient temperature, the ethylene balloon was removed and 0.1 mL methanol was added to the flask. The solution was diluted with pentane (3 mL) and subsequently passed through a silica plug. The plug was washed with pentane (3 x 5 mL). Removal of solvent yielded the product as a mixture of hydrovinylation product (**13d**,1,4-*Z*, 79%) and linear hydrovinylation product(**16d**, 1,4-Z-linear, 21%) from GC and NMR analysis. All the reactions in Table 2 using Zn and ZnI$_2$ were performed exactly the same way using the conditions described therein.

Unsuccessful hydrovinylation of (*E*)-dodeca-1,3-diene using DPPBCoCl$_2$ and diethyl aluminum chloride (Et$_2$AlCl) as a promoter (Table 2, Entry 13). To an oven dried round-bottom-flask with a sidearm, was added DPPBCoCl$_2$ (35 mg, 0.063 mmol) and under argon it was dissolved in dichloromethane (1 mL) at room temperature. Diethylaluminium chloride (Et$_2$AlCl) as a 2M solution in toluene (0.16 mL, 0.32 mmol) was added dropwise and the color of the solution remain unchanged from its blue color. After 2 to 3 minutes, the reaction vessel was carefully evacuated and then refilled with ethylene from a balloon. The reaction vessel was cooled to -10 ^oC and (*E*)-dodeca-1,3-diene (105 mg, 0.63 mmol) in a dichloromethane (1 mL) added under ethylene and the mixture was stirred for 5 h. The ethylene balloon was removed and 0.1 mL methanol was added to the flask. The solution was diluted with pentane (5 mL) and warmed to room temperature and subsequently passed through a silica plug. The plug was washed with pentane (3 x 10 mL). Removal of solvent and analysis by GC and NMR gave none of the expected hydrovinylation products.

Unsuccessful hydrovinylation of nona-1,3-diene using [DPPM]CoCl$_2$ and MeMgBr and AgOTf as promoters (Promoters screening, Table 2, Entry 12). As a typical procedure, to an oven dried round-bottom-flask with a sidearm, was added [DPPM]CoCl$_2$ (19 mg, 0.037 mmol) under argon and was dissolved in dichloromethane (1 mL) at room temperature. Methylmagnesium bromide (MeMgBr) as a 3M solution in ether (0.01 mL, 0.037 mmol) was added via syringe and the color of the solution changed from deep blue to black with the formation of white fumes over the solution. Subsequently, AgOTf (9.3 mg, 0.037 mmol) was added to the solution. After 3 minutes, the reaction vessel was carefully evacuated and then refilled with ethylene from a balloon. The reaction vessel was cooled to 0 ^0C and the nona-1,3-

diene (46 mg, 0.37 mmol) in dichloromethane (1 mL) added under ethylene and the mixture was stirred for 4 h at 0 ^{0}C to rt. The ethylene balloon was removed and 0.1 mL methanol was added to the flask. The solution was diluted with pentane (3 mL) and warmed to room temperature and subsequently passed through a silica plug. The plug was washed with pentane (3 X 6 mL). Removal of solvent recovered the starting material back. GC and NMR analysis showed that the product was not formed at all.

Iron-catalyzed hydrovinylation of (*E*)-nona-1,3-diene using[DPPE]FeCl$_3$ (Equation 11): To an oven dried round-bottom-flask with a sidearm, was added [DPPE]FeCl$_3$ (56 mg, 0.099 mmol) under argon and was dissolved in dichloromethane (1 mL) at room temperature. Trimethylaluminium as a 2M solution in toluene (0.24 mL, 0.49 mmol) was added dropwise and the color of the solution changed from light pink to red brown with the formation of white fumes over the solution. After 2 to 3 minutes, the reaction vessel was carefully evacuated and then refilled with ethylene from a balloon. The reaction vessel was cooled to -10 ^{o}C and (*E*)-nona-1,3-diene (61 mg, 0.49 mmol) as a dichloromethane solution (0.5 mL) was added under ethylene and the mixture was stirred for 3 h at ambient temperature. The ethylene balloon was removed and 0.1 mL methanol was added to the flask. The solution was diluted with pentane (5 mL) and warmed to room temperature and subsequently passed through a silica plug. The plug was washed with pentane (3 X 7 mL). Removal of solvent yielded the product as a colorless oil as a mixture of (*Z*)-1,4-product (30%), 1,4-linear dimerized product (10%) and starting material (60%) by GC and NMR analysis.

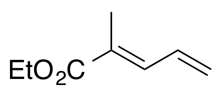

Unsuccessful hydrovinylation of substrate (*E*)-ethyl-2-methylpenta-2,4-dienoate using [dppm]CoCl$_2$. To an oven dried round-bottom-flask with a sidearm, was added [dppm]CoCl$_2$ (30 mg, 0.058 mmol) under argon and was dissolved in dichloromethane (1 mL) at room temperature. Trimethylaluminium as a 2M solution in toluene (0.6 ml, 1.16 mmol) was added dropwise and the color of the solution changed from deep blue to red brown with the formation of white fumes over the solution. After 2 to 3 minutes, the reaction vessel was carefully evacuated and then refilled with ethylene from a balloon. The reaction vessel was cooled to 0 ^{o}C and (*E*)-ethyl-2-methylpenta-2,4-dienoate (81 mg, 0.58 mmol) as a dichloromethane solution (0.5 mL) added under ethylene and the mixture was stirred for 15 h. The ethylene balloon was removed and 0.1 mL methanol was added to the flask. The solution was diluted with pentane (2 mL) and warmed to room temperature and subsequently passed through a silica plug. The plug was washed with pentane (3 X 5 mL). Removal of solvent recovered the starting material back. GC and NMR analysis showed that the product was not formed at all.

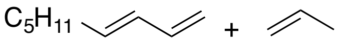

Unsuccessful attempt to dimerize (*E*)-1,3-nonadiene and propylene using [dppe]CoCl$_2$ (Equation 10). To an oven dried round-bottom-flask with a sidearm, was added (dppe)CoCl$_2$ (30 mg, 0.056 mmol) under argon and was dissolved in dichloromethane (1 mL) at room temperature. Trimethylaluminium as a 2M solution in toluene (0.08 mL, 0.17 mmol) was added dropwise and the color of the solution changed from deep blue to red brown with the formation of white fumes over the solution. After 2 to 3 minutes, the reaction vessel was carefully evacuated and then refilled with propylene from a balloon. The reaction vessel was cooled to -20 ^{o}C and (*E*)-1,3-nonadiene (70 mg, 0.56 mmol) as a dichloromethane solution (0.5 mL) was added under propylene and the mixture was stirred

for 14 h. The propylene balloon was removed and 0.1 mL of methanol was added to the flask. The solution was diluted with pentane (4 mL) and warmed to room temperature and subsequently passed through a silica plug. The plug was washed with pentane (3 x 7 mL). Removal of solvent showed no codimerization reaction as all the (*E*)-1,3-nonadiene starting material was recovered as judged by GC and ^{1}H NMR.

Cobalt-Catalyzed Hydrovinylation of 1,3-Dienes. Scope of Substrates (Tables 3 and 5)

Hydrovinylation of different dienes and the major products obtained from them are listed below:

Hydrovinylation of (*E*)-nona-1,3-diene (12a)

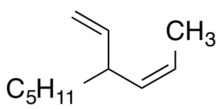

The exploratory studies of effects of ligands and additives were conducted using this substrate. Rigorous identifications of all products from this substrate were described in our original communication.[2] For hydrovinylation procedure for (*E*)-nona-1,3-diene using[DPPB]$CoCl_2$ (Table 1, Entry 9, see under **Ligand Screening**. Details of the identification of various products were also listed earlier).

Typical procedure for asymmetric Co-catalyzed hydrovinylation reactions of (*E*)-nona-1,3-diene for ligand screening (Table 4, Entry 1-20). To an oven-dried round-bottom flask with a sidearm, was added (*RR*)-[DIOP]$CoCl_2$ (12.6 mg, 0.0201 mmol) under argon and it was dissolved in a mixture of degassed dichloromethane (2.0 mL) and toluene (0.5 mL), at 0 °C. Trimethylaluminum solution (2M) in toluene (4.3 mg, 30 μl, 0.0604 mmol) was added dropwise as color of the solution changed from deep blue to red-brown with the formation of white fumes over the solution. When all the fumes disappeared (typically in 5-10 min depending on complex), the reaction vessel was carefully evacuated and then refilled with ethylene from a balloon. The filled balloon was used to maintain the ethylene atmosphere, while a vigorous reaction with evolution of fumes was observed. This evolution stopped in typically 3-5 min. The reaction vessel was cooled to – 45 °C (or whatever is the prescribed temperature with other ligands and substrates) and (*E*)-nona-1,3-diene (50.0 mg, 0.403 mmol) was added under ethylene and the mixture was stirred for 6 h (color of the reaction solution turned blue again at the end of the reaction). The ethylene balloon was removed and 0.1 mL of methanol was introduced into the flask and stirring was continued for 5 minutes. The solution was warmed to room temperature and was subsequently passed through a silica plug. The plug was washed with pentane (3 x 10 mL). Concentration and removal of last traces of solvent yielded the product as a colorless oil (58.2 mg, 95%). Analysis by GC and NMR showed that the product was essentially pure. Isomeric compositions were determined by gas chromatography and NMR spectroscopy.

Details of the identification of the individual components of the mixture and chromatographic separation of these compounds including those of the enantiomers have been reported before.[2]

The enantioselectivities obtained with various other chiral ligands under different conditions are listed in Table 4. Typical examples are described below:

Asymmetric Co-catalyzed Hydrovinylation Reactions (*E*)-1,3-nonadiene by using (*S,S,R,R*)-Tangphos [(L14)$CoCl_2$] (Table 5, Entry 3). To an oven-dried round-bottom flask with a sidearm, was added (*S,S,R,R*)-[Tangphos]$CoCl_2$ (19 mg, 0.046 mmol) under argon and it was dissolved in a degassed dichloromethane (1.0 mL) at room temperature. Trimethylaluminum solution (2M) in toluene (0.07 mL, 0.14 mmol) was added dropwise as color of the solution changed from deep blue to red-brown with the formation of white fumes over the solution.

When all the fumes disappeared (typically in 5 min), the reaction vessel was carefully evacuated and then refilled with ethylene from a balloon. The filled balloon was used to maintain the ethylene atmosphere, while a vigorous reaction with evolution of fumes was observed. This evolution stopped in typically 3 minute. The reaction vessel was cooled to – 10 °C and (*E*)-nona-1,3-diene (57 mg, 0.46 mmol) in dichloromethane (1 mL) was added under ethylene and the mixture was stirred for 8 h. The ethylene balloon was removed and 0.1 mL of methanol was introduced into the flask and stirring was continued for 5 minutes. The solution was warmed to room temperature and was subsequently passed through a silica plug. The plug was washed with pentane (3 x 10 mL). Concentration and removal of last traces of solvent yielded the product as a colorless oil. Analysis by GC and NMR showed that the product was essentially pure with two major isomers (**13a** product with 39% yield and 95% ee and the linear product **16a** product in 40% yield). Isomeric compositions were determined by gas chromatography and NMR spectroscopy (See attached chromatograms and Spectra).

Asymmetric Co-catalyzed hydrovinylation reaction (*E*)-1,3-nonadiene by using 1 mol % Josiphos 2 [(18)$CoCl_2$] (Table 5, Entry 4). To an oven-dried round-bottom flask with a sidearm, was added [Josiphos]$CoCl_2$ (4 mg, 0.0038 mmol) under argon and it was dissolved in a degassed dichloromethane (1.0 mL) at room temperature. Trimethylaluminum solution (2M) in toluene (0.0057 mL, 0.0114 mmol) was added dropwise as color of the solution changed from deep blue to red-brown with the formation of white fumes over the solution. When all the fumes disappeared (typically in 5 min), the reaction vessel was carefully evacuated and then refilled with ethylene from a balloon. The filled balloon was used to maintain the ethylene atmosphere, while a vigorous reaction with evolution of fumes was observed. This evolution stopped in typically 3 minute. The reaction vessel was cooled to – 20 °C and (*E*)-nona-1,3-diene (47 mg, 0.38 mmol) in dichloromethane (1 mL) was added under ethylene and the mixture was stirred for 14 h until the completion of reaction monitored by gas chromatography. The ethylene balloon was removed and 0.1 mL of methanol was introduced into the flask and stirring was continued for 5 minutes. The solution was warmed to room temperature and was subsequently passed through a silica plug. The plug was washed with pentane (3 x 10 mL). Concentration and removal of last traces of solvent yielded the product as a colorless oil. Analysis by GC and NMR showed that the product was essentially pure with major isomer (**13a** product with 95% yield and 87% ee). Isomeric compositions were determined by gas chromatography and NMR spectroscopy.

Highest ee for this substrate was obtained using (*R,R)*-DIOP (>95% ee, >95% yield) or (*S,S*)-BDPP (>97% ee, >95% yield) at -45 °C.[2]

13a using Cl2Co(dppb Entry 1, Table 3)

methylsilicone 80 degree , isotherm

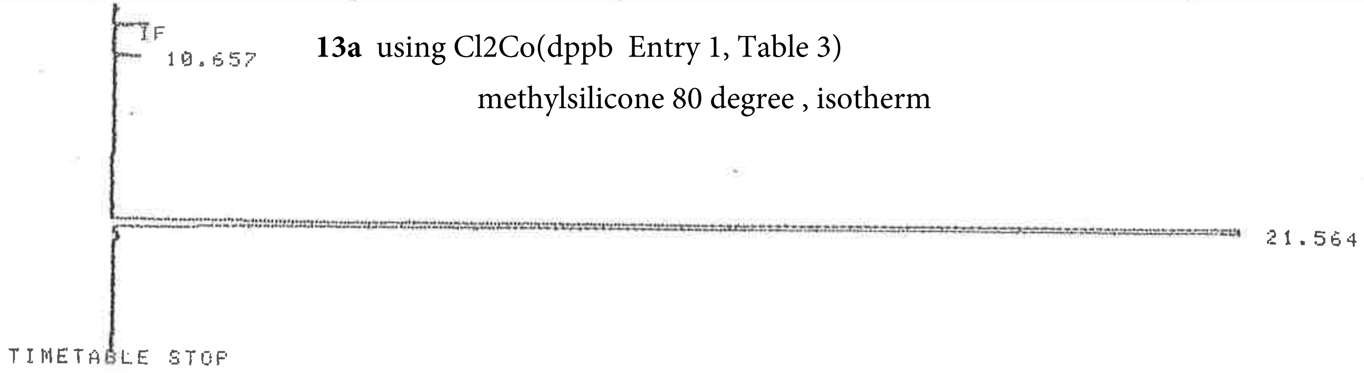

Error storing signal to M:SIGNAL .RAW
ATTEMPTED WRITE PAST END OF FILE

RUN# 948 SEP 11, 1901 14:14:13

AREA%

RT	AREA	TYPE	WIDTH	AREA%
10.657	16423	VP	.086	.22850
21.564	7170784	PB	.174	99.77152

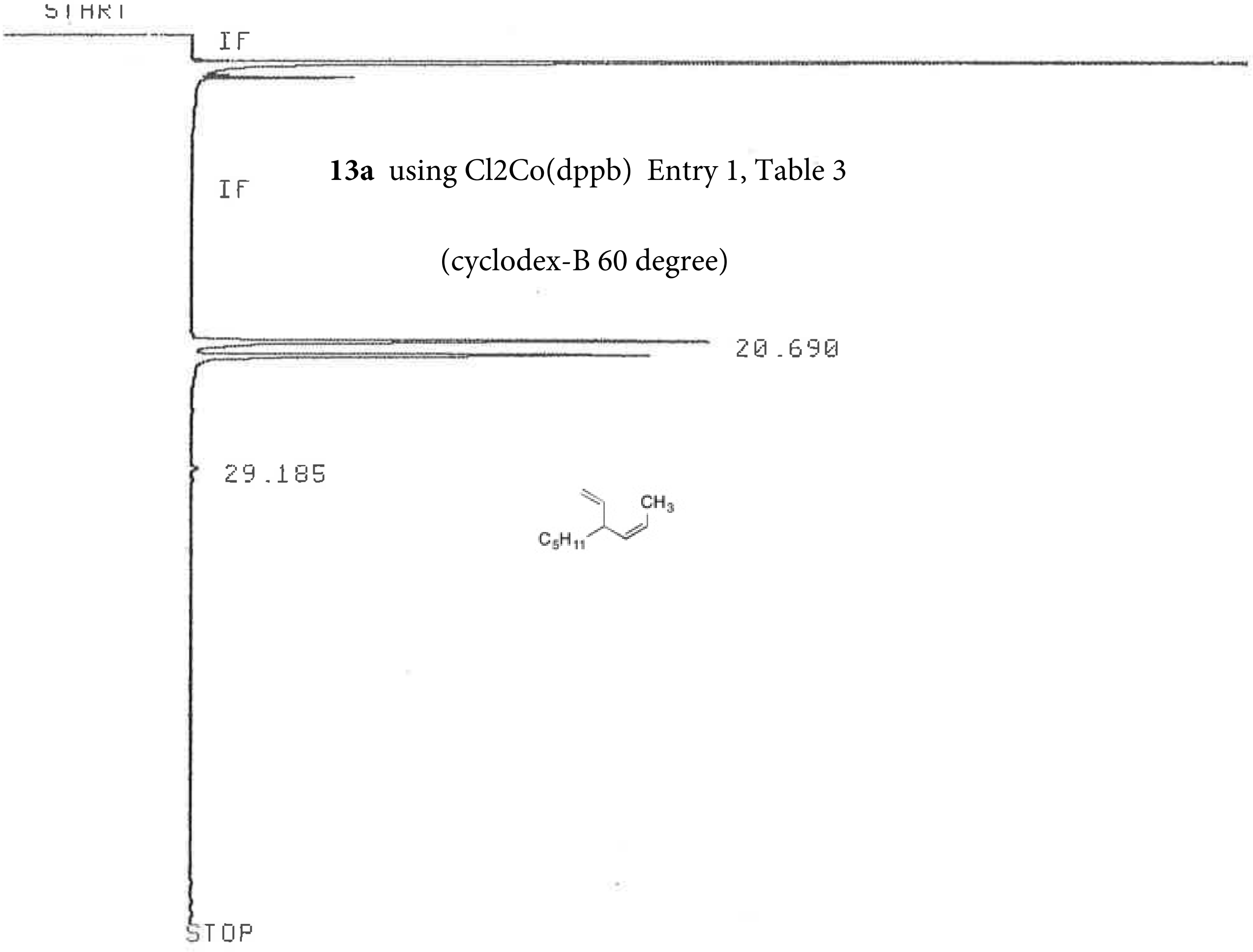

13a using Cl2Co(dppb) Entry 1, Table 3

(cyclodex-B 60 degree)

Closing signal file M:SIGNAL .BNC

RUN# 3884 MAY 6, 2009 01:42:26

SIGNAL FILE: M:SIGNAL.BNC
AREA%

RT	AREA	TYPE	WIDTH	AREA%
20.690	48959	VV	.219	53.51470
21.644	42528	VV	.215	46.48530

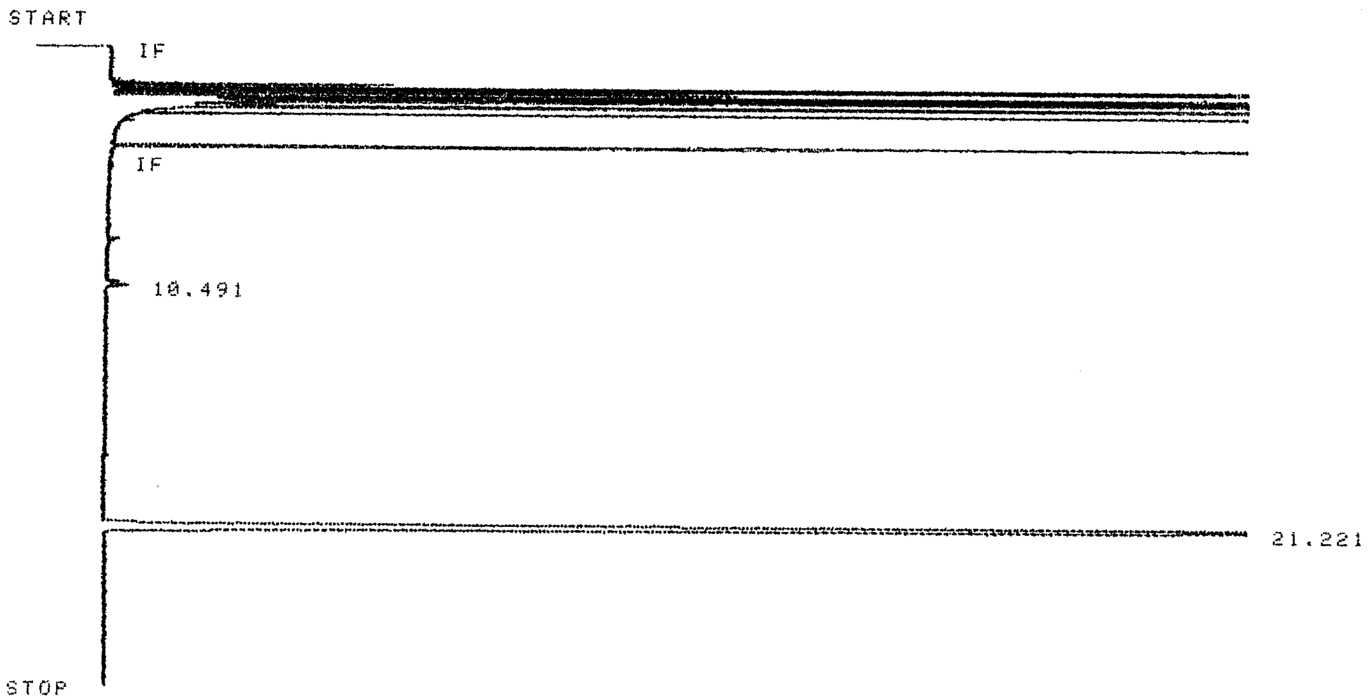

Error storing signal to M:SIGNAL .RAW
ATTEMPTED WRITE PAST END OF FILE

RUN# 835 JUN 24, 1901 11:20:05

AREA%

RT	AREA	TYPE	WIDTH	AREA%
21.221	1083970	PB	.168	100.00000

TOTAL AREA=1083970
MUL FACTOR=1.0000E+00

*
*
*

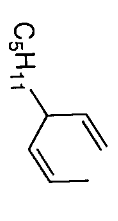

(*RR*)-(−)[DIOP]$CoCl_2$/−45 °C

(Polydimethylsiloxane, 80 °C)

(Entry1, Table 5)

Overtemp =80 C / [illegible]
RB-2-152

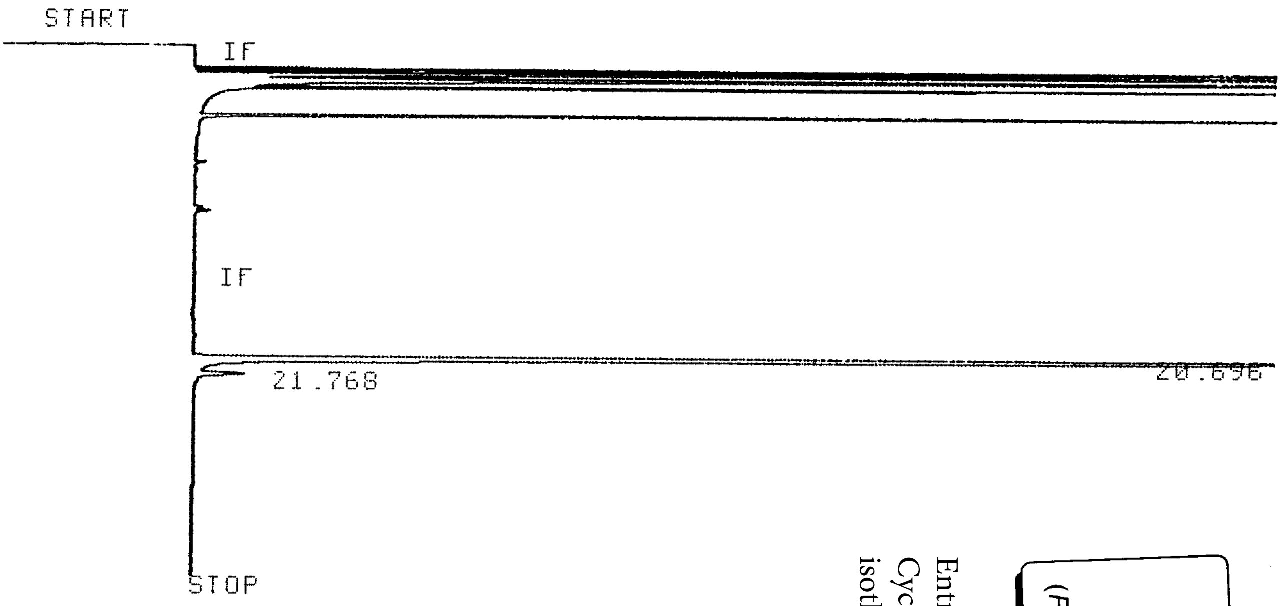

Closing signal file M:SIGNAL .BNC

RUN# 3919 MAY 16, 2009 00:35:45

SIGNAL FILE: M:SIGNAL.BNC
AREA%

RT	AREA	TYPE	WIDTH	AREA%
20.696	75830	BB	.203	98.01462
21.768	1536	BP	.179	1.98537

TOTAL AREA= 77366
MUL FACTOR=1.0000E+00

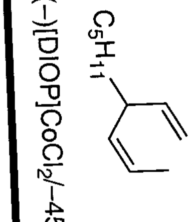

(*RR*)-(−)[DIOP]$CoCl_2$/−45 °C
(Cyclodex-B, 60 °C)

Entry1, Tble 5
Cyclodex-B, 60 degree isotherm)

RXL.2.152 / 5% (−)DIOPCoCl2 / isotherm 60°C
(−45°C)

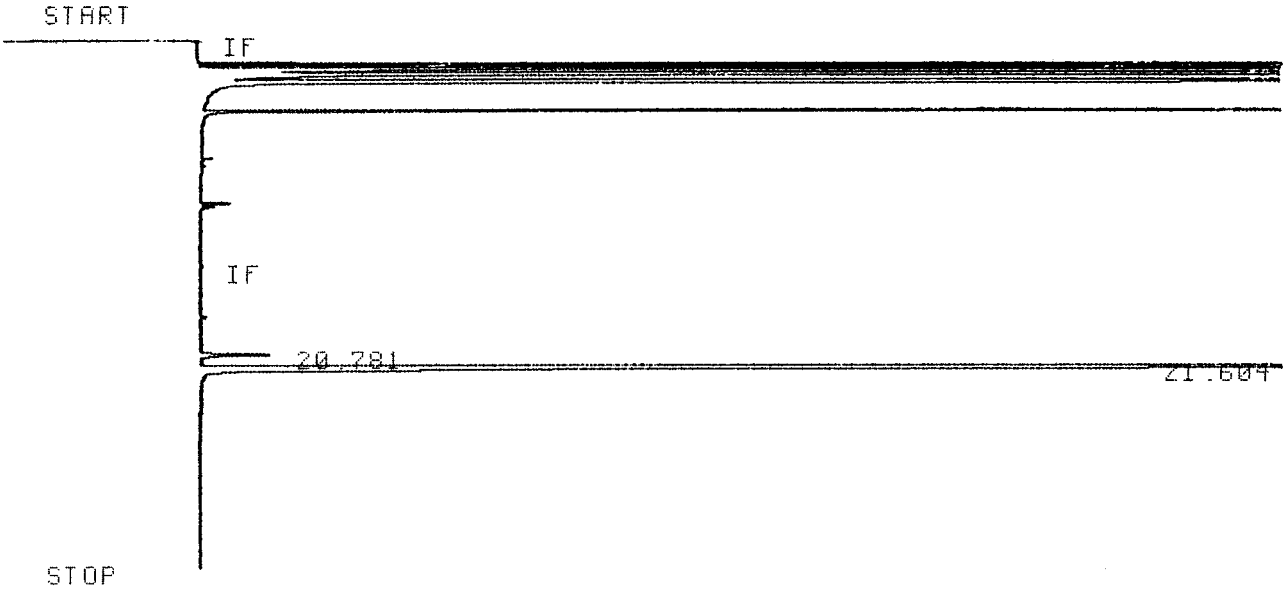

Closing signal file M:SIGNAL .BNC

RUN# 3910 MAY 13, 2009 02:51:50

SIGNAL FILE: M:SIGNAL.BNC
AREA%

RT	AREA	TYPE	WIDTH	AREA%
20.781	2449	VV	.167	3.31879
21.604	71343	PV	.206	96.68122

TOTAL AREA= 73792
MUL FACTOR=1.0000E+00

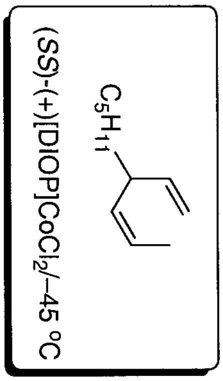

(Cyclodex-B, 60 °C)

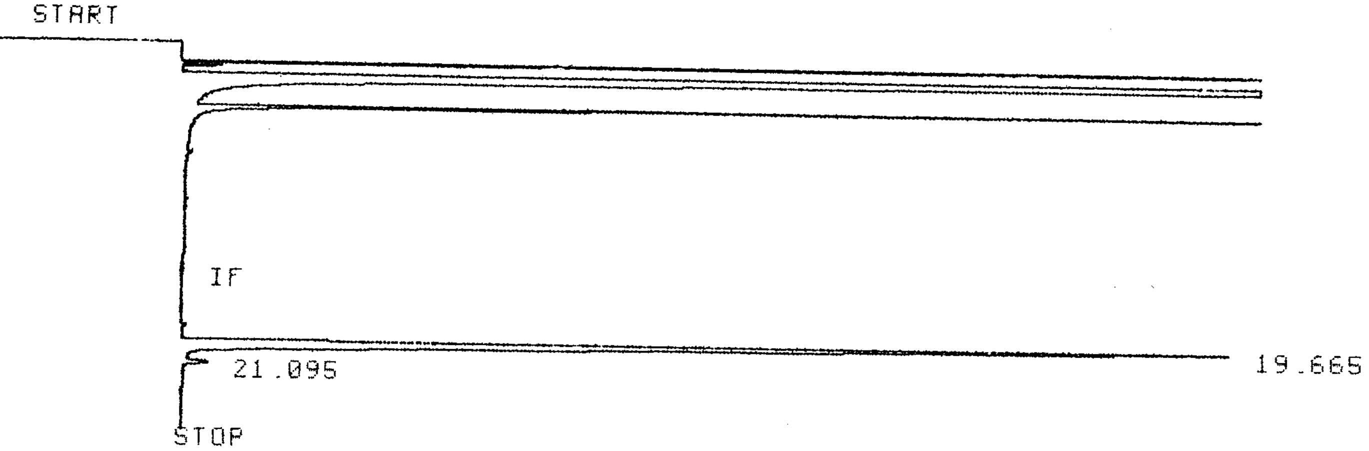

Closing signal file M:SIGNAL .BNC

RUN# 4007 AUG 3, 2009 00:35:24

SIGNAL FILE: M:SIGNAL.BNC
AREA%

RT	AREA	TYPE	WIDTH	AREA%
19.665	592287	BB	.335	98.56893
21.095	8599	BB	.219	1.43105

TOTAL AREA= 600886
MUL FACTOR=1.0000E+00

13a using (S,S)-(BDPP) Entry 2, Table 5)

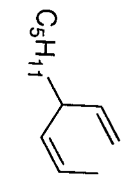

(SS)-(–)[BDPP]$CoCl_2$/–45 °C
(Cyclodex-B, 60 °C)

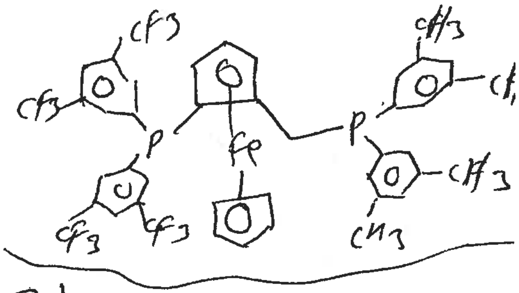

YT-03-281
OT 70°C, cyclosil, Isothermal.
(1 mol% catalyst)

RUN # 4772   FEB 17, 2012 20:24:37
ART

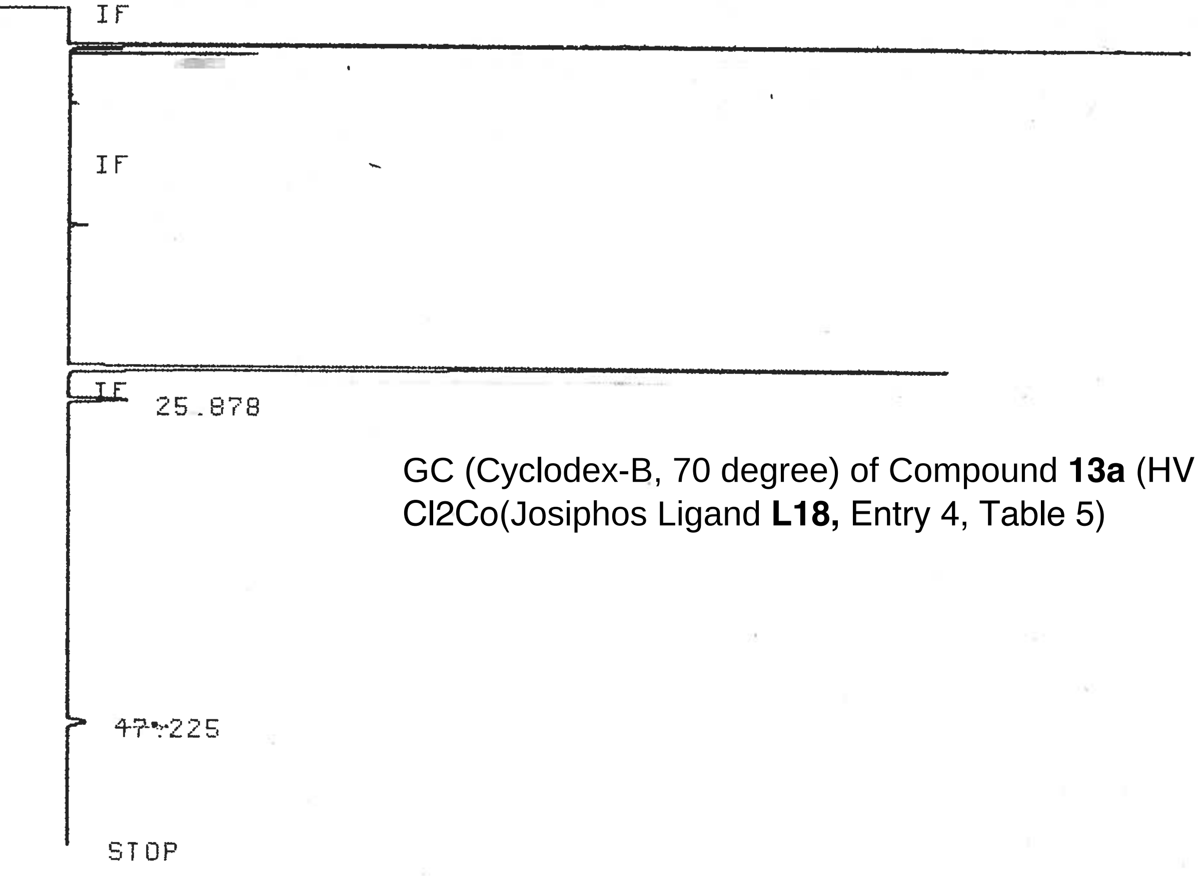

GC (Cyclodex-B, 70 degree) of Compound **13a** (HV using Cl2Co(Josiphos Ligand **L18,** Entry 4, Table 5)

osing signal file M:SIGNAL .BNC

N# 4772   FEB 17, 2012 20:24:37

GNAL FILE: M:SIGNAL.BNC
EA%

RT	AREA	TYPE	WIDTH	AREA%
14.375	2058	BP	.113	1.20836
23.720	151804	BB	.204	89.13184
25.878	10506	BB	.206	6.16860
47.225	5946	BB	.349	3.49120

TAL AREA= 170314
L FACTOR=1.0000E+00

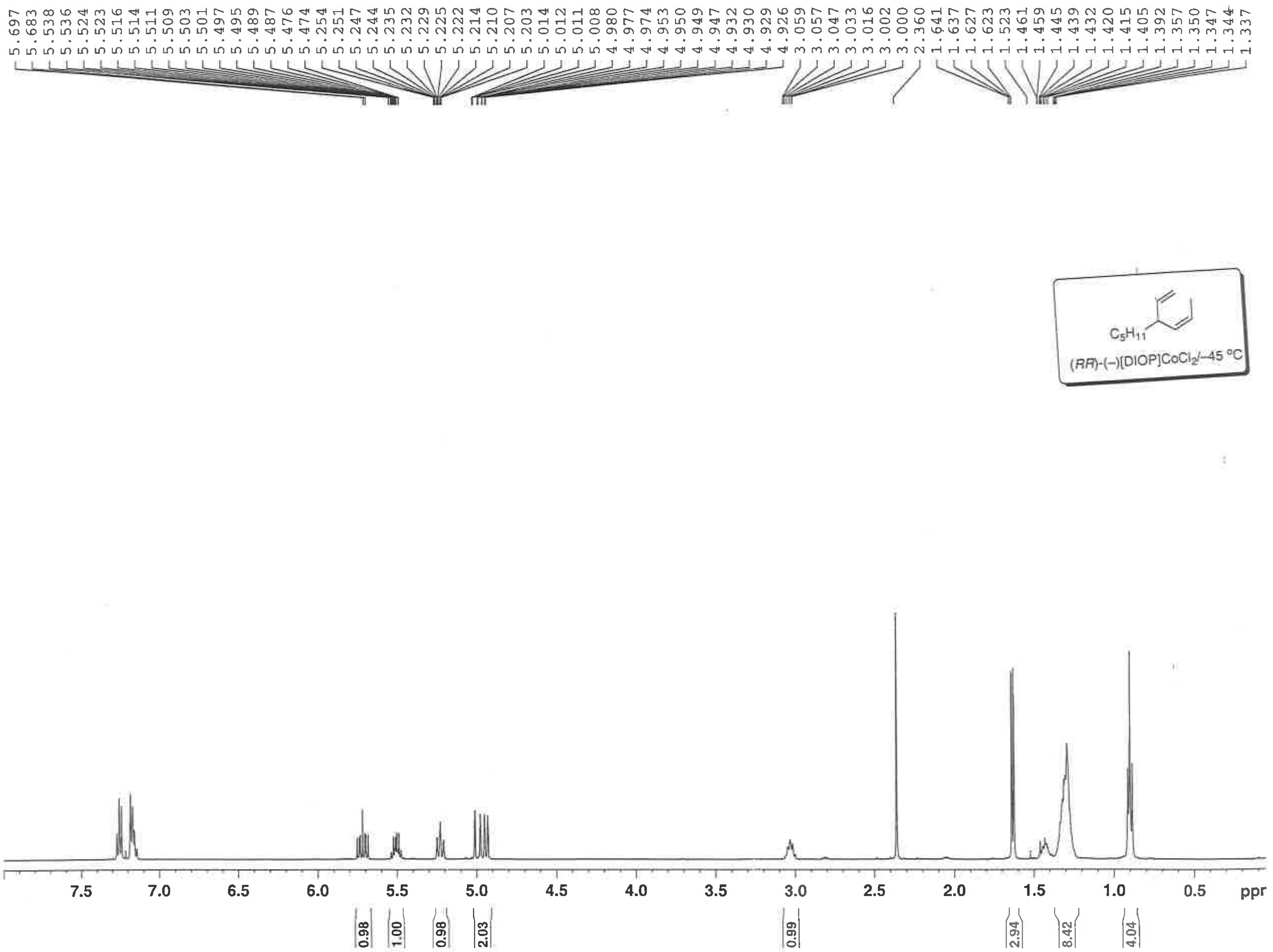
5.697
5.683
5.538
5.536
5.524
5.523
5.516
5.514
5.511
5.509
5.503
5.501
5.497
5.495
5.489
5.487
5.476
5.474
5.254
5.251
5.247
5.244
5.235
5.232
5.229
5.225
5.222
5.214
5.210
5.207
5.203
5.014
5.012
5.011
5.008
4.980
4.977
4.974
4.953
4.950
4.949
4.947
4.932
4.930
4.929
4.926
3.059
3.057
3.047
3.033
3.016
3.002
3.000
2.360
1.641
1.637
1.627
1.623
1.523
1.461
1.459
1.445
1.439
1.432
1.420
1.415
1.405
1.392
1.357
1.350
1.347
1.344
1.337
C_5H_{11}
(RR)-(–)[DIOP]$CoCl_2$/–45 °C
7.5
7.0
6.5
6.0
5.5
5.0
4.5
4.0
3.5
3.0
2.5
2.0
1.5
1.0
0.5
ppm
0.98
1.00
0.98
2.03
0.99
2.94
8.42
4.04

(54)

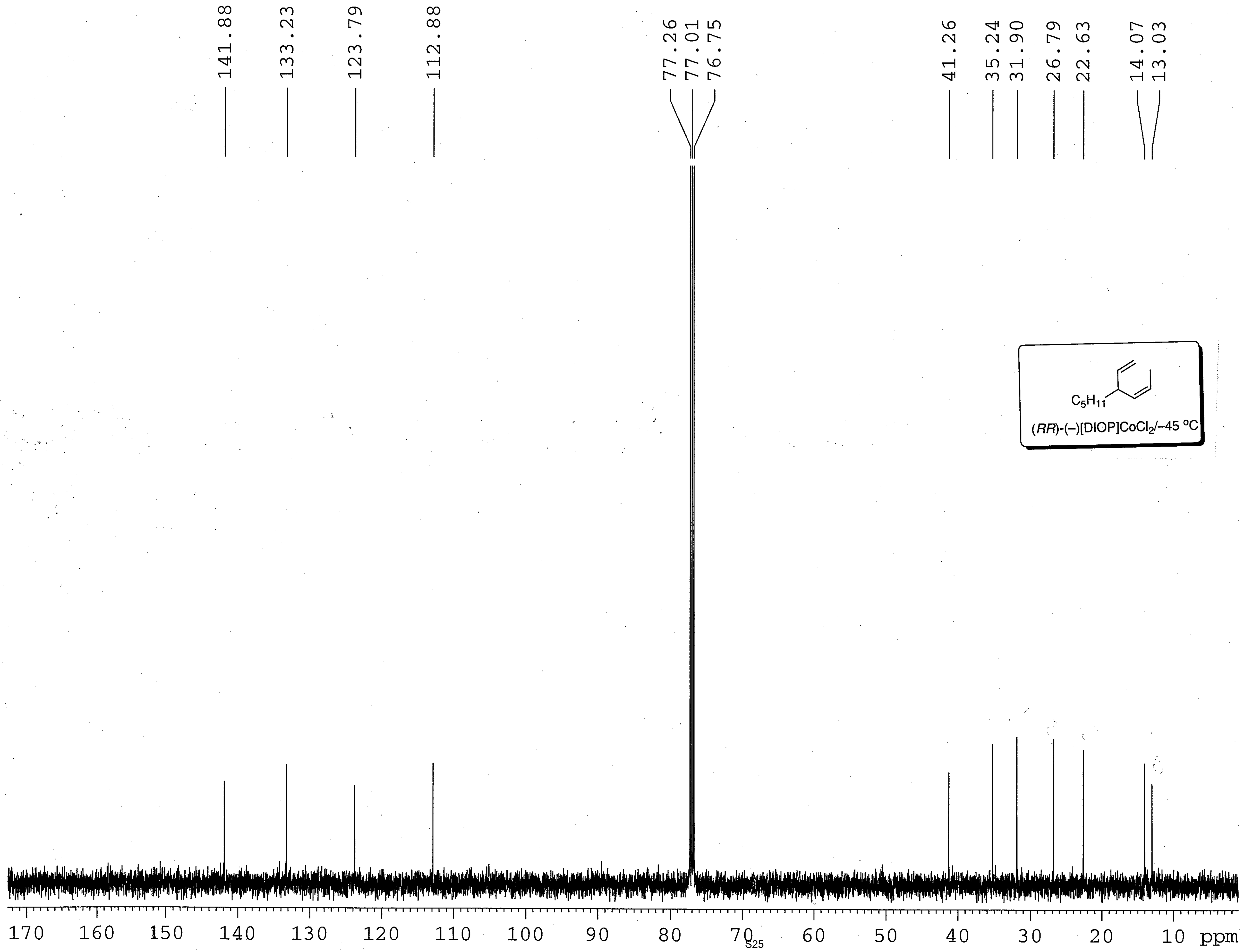

(*RR*)-(–)[DIOP]$CoCl_2$/–45 °C

Current Data Parameters
NAME RKS-2-152 again carbon
EXPNO 383
PROCNO 1

F2 - Acquisition Parameters
Date_ 20091224
Time 13.29
INSTRUM spect
PROBHD 5 mm Multinucl
PULPROG zgpg30
TD 65536
SOLVENT CDCl3
NS 50
DS 4
SWH 30030.029 Hz
FIDRES 0.458222 Hz
AQ 1.0912244 sec
RG 3251
DW 16.650 usec
DE 12.00 usec
TE 300.2 K
D1 2.00000000 sec
d11 0.03000000 sec
DELTA 1.89999998 sec
MCREST 0.00000000 sec
MCWRK 0.01500000 sec

======== CHANNEL f1 ========
NUC1 13C
P1 12.00 usec
PL1 3.00 dB
SFO1 125.7427020 MHz

======== CHANNEL f2 ========
CPDPRG2 waltz16
NUC2 1H
PCPD2 100.00 usec
PL2 -1.00 dB
PL12 15.59 dB
PL13 22.50 dB
SFO2 500.0220001 MHz

F2 - Processing parameters
SI 32768
SF 125.7301290 MHz
WDW EM
SSB 0
LB 1.00 Hz
GB 0
PC 1.00

Hydrovinylation of (*E*)-deca-1,3-diene (12b)

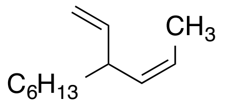

Hydrovinylation of (*E*)-deca-1,3-diene using (*R,R*)[DIOP]$CoCl_2$ (Table 5, Entry 5). To an oven dried round bottom flask with a sidearm, was added (*RR*)[DIOP]$CoCl_2$ (3 mg, 0.0028 mmol) and dichloromethane (0.5 mL) was added. Trimethylaluminium solution (2 M) (0.05 mL,0.008 mmol) was added to the solution and the color changed from deep blue to red brown with the formation of white fumes. After 2 to 3 minutes, the reaction vessel was carefully evacuated and then refilled with ethylene balloon. The reaction vessel was kept at -45 ^{o}C and (*E*)-deca-1,3-diene (43 mg, 0.28 mmol), was added via syringe under ethylene and the mixture was stirred for 6 h. The ethylene balloon was removed and 0.1 mL methanol was added into the flask. The reaction solution was diluted with pentane (5 mL) and warmed to room temperature and subsequently passed through a silica plug. The plug was washed with pentane (3 x 6 mL). Removal of the solvent yielded the product as colorless oil. GC showed 97% (*Z*)-1,4-product and 3% 1,4-linear dimerized product.

An authentic sample of the racemic compound was prepared by similar procedure (Table 3, Entry 2) using corresponding achiral complex [(dppb)$CoCl_2$] at the prescribed temperature.

^{1}H NMR ($CDCl_3$): δ 0.891 (t, J = 7 Hz, 3 H, H^{10}), 1.23-1.40 (m, 9 H), 1.38-1.47 (m, 1 H), 1.23-1.47 total 10 H, 1.628 (dd, J = 7, 2 Hz, 3 H, H^{1}), 3.027 (m, 1 H, H^{4}), 4.935 (dm, J = 10 Hz, H^{12}), 4.987 (ddd, J = 17.5 Hz, m, 1 H, H^{13}), 5.222 (app t m, 10.5 Hz, H^{3}), 5.502 (dqm, 11.5 Hz, 6.5 Hz, 1 Hz, H^{2}), 5.714 (ddd, 17 Hz, 10 Hz, 7 Hz, 1H, H^{11}). [toluene @ 2.364]. Also seen: (t, J = 6.5 Hz) 0.221 up-field from C_{sp3}-C*H* (3.027) ~ 2% (bis-allylic C*H*$_2$).
^{13}C ($CDCl_3$) 13.01, 14.07, 22.66, 27.10, 29.34. 31.87, 35.30, 41.26, 112.86, 123.77, 133.23, 141.86.
Mass spectrum: m/z = 167.1 (calculated for $[M+H]^+$, M= $C_{12}H_{23}$ 167.1)

Product from (*RR*)-(–)-DIOP $[\alpha]^{25}_{D}$ = –18.6 (hexane, *c* 1.25)

Gas chromatography: (Polydimethylsiloxane) conditions: 90 ^{o}C isotherm, retention time (min): R_T = 27.40. CSP GC (Cyclodex) conditions: 60 ^{o}C isotherm, R_T = 35.94 min. (*S*, 97.7%), 38.07 (*R*, 2.3%).

* RUN # 893 AUG 5, 1901 13:19:05

START

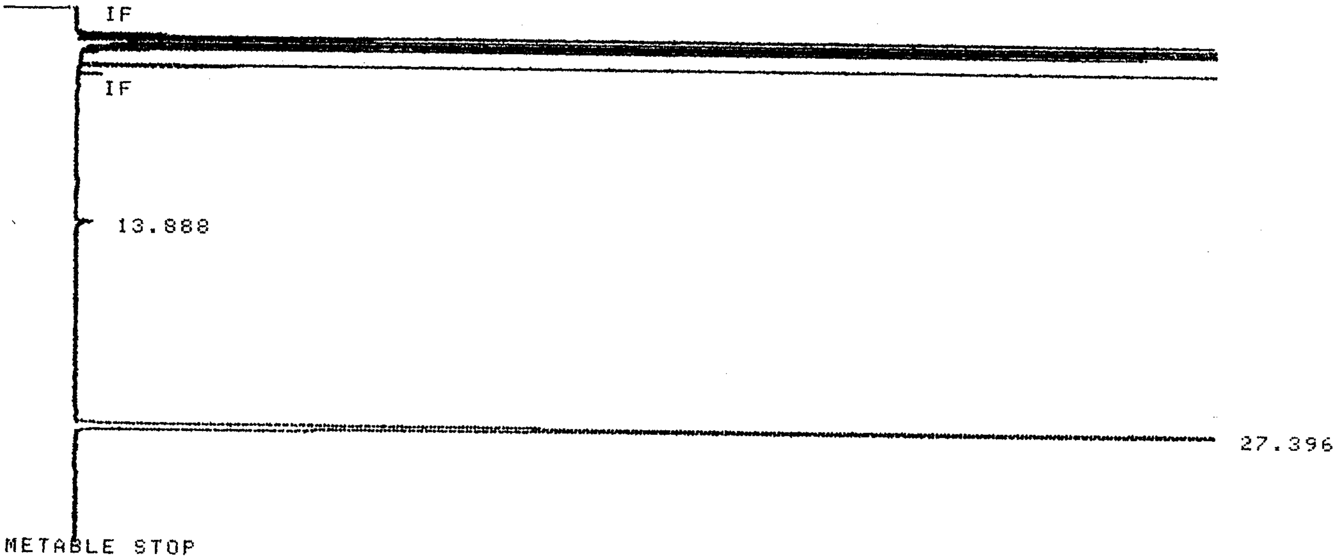

TIMETABLE STOP

Error storing signal to M:SIGNAL .RAW

ATTEMPTED WRITE PAST END OF FILE

RUN# 893 AUG 5, 1901 13:19:05

AREA%

RT	AREA	TYPE	WIDTH	AREA%
13.888	11686	PV	.116	.69185
27.396	1677406	PP	.214	99.30813

TOTAL AREA=1689092

MUL FACTOR=1.0000E+00

13b using (RR)-DIOP -45 degree
methylsilicone column
90 degree isotherm, Entry 5, Table 5

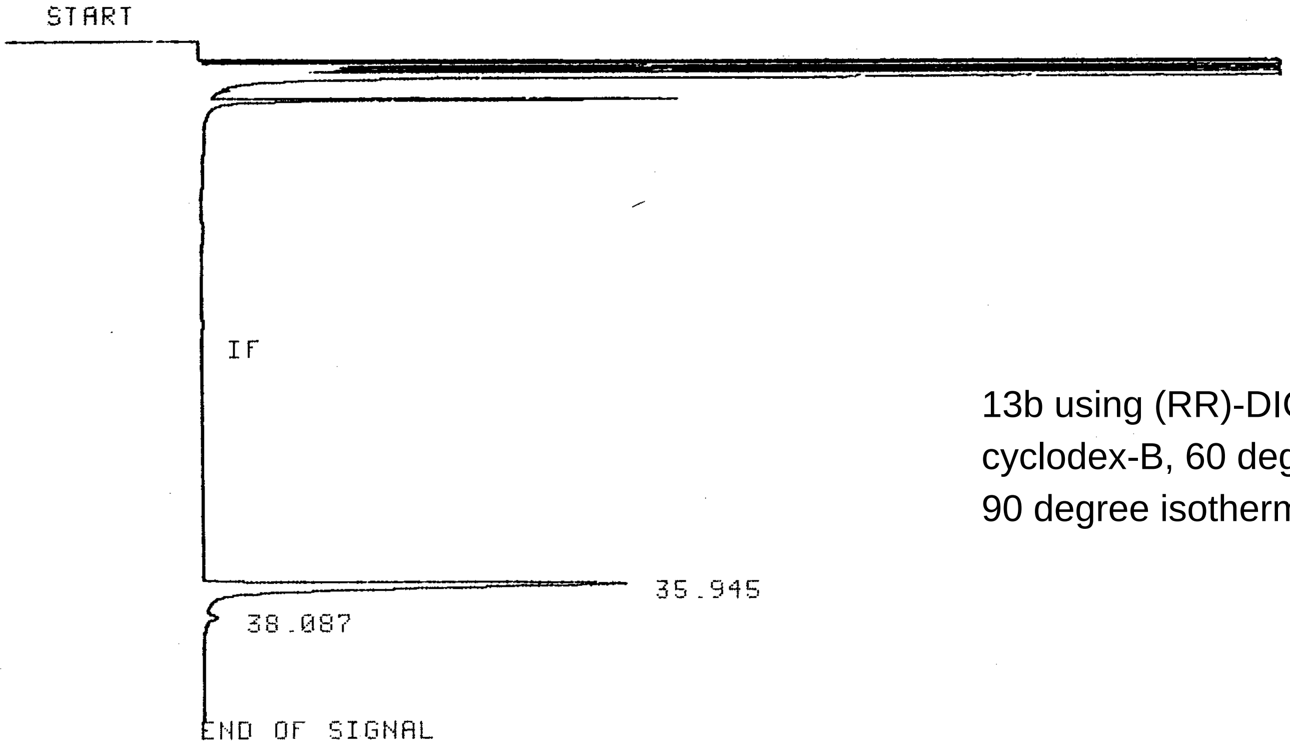

13b using (RR)-DIOP -45 degree cyclodex-B, 60 degree isoterm, column 90 degree isotherm, Entry 5, Table 5

Closing signal file M:SIGNAL .BNA

RUN# 3963 JUN 27, 2009 22:45:12

SIGNAL FILE: M:SIGNAL.BNA
AREA%

RT	AREA	TYPE	WIDTH	AREA%
35.945	167914	BV	.472	97.69712
38.087	3958	BV	.402	2.30288

TOTAL AREA= 171872
MUL FACTOR=1.0000E+00

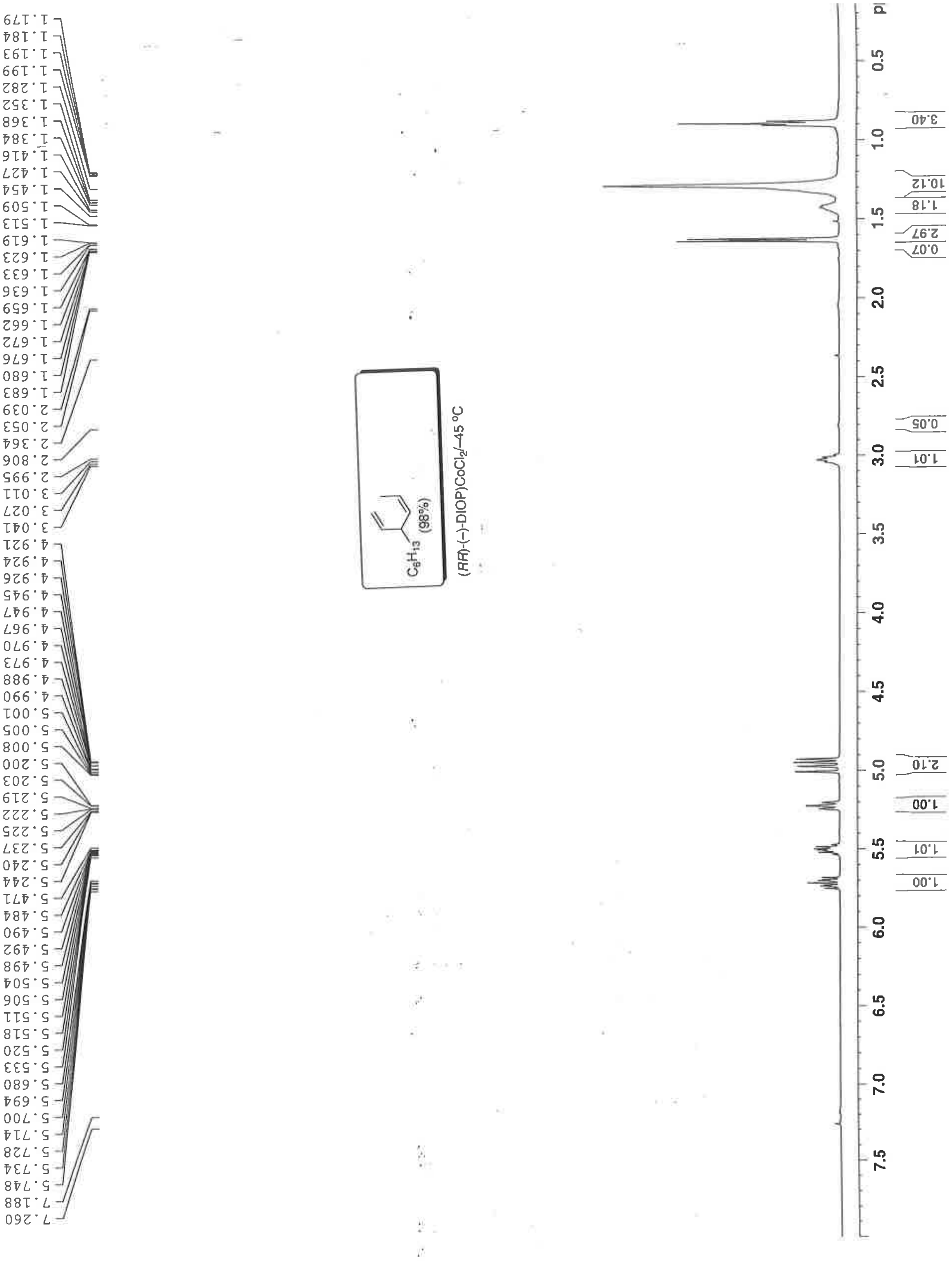
1.179
1.184
1.193
1.199
1.282
1.352
1.368
1.384
1.416
1.427
1.454
1.509
1.513
1.619
1.623
1.633
1.636
1.659
1.662
1.672
1.676
1.680
1.683
2.039
2.053
2.364
2.806
2.995
3.011
3.027
3.041
4.921
4.924
4.926
4.945
4.947
4.967
4.970
4.973
4.988
4.990
5.001
5.005
5.008
5.200
5.203
5.219
5.222
5.225
5.237
5.240
5.244
5.471
5.484
5.490
5.492
5.498
5.504
5.506
5.511
5.518
5.520
5.533
5.680
5.694
5.700
5.714
5.728
5.734
5.748
7.188
7.260
C_6H_{13}
(98%)
(RR)-(−)-DIOP)CoCl2/−45 °C
3.40
10.12
1.18
2.97
0.07
0.05
1.01
2.10
1.00
1.01
1.00
0.5
1.0
1.5
2.0
2.5
3.0
3.5
4.0
4.5
5.0
5.5
6.0
6.5
7.0
7.5

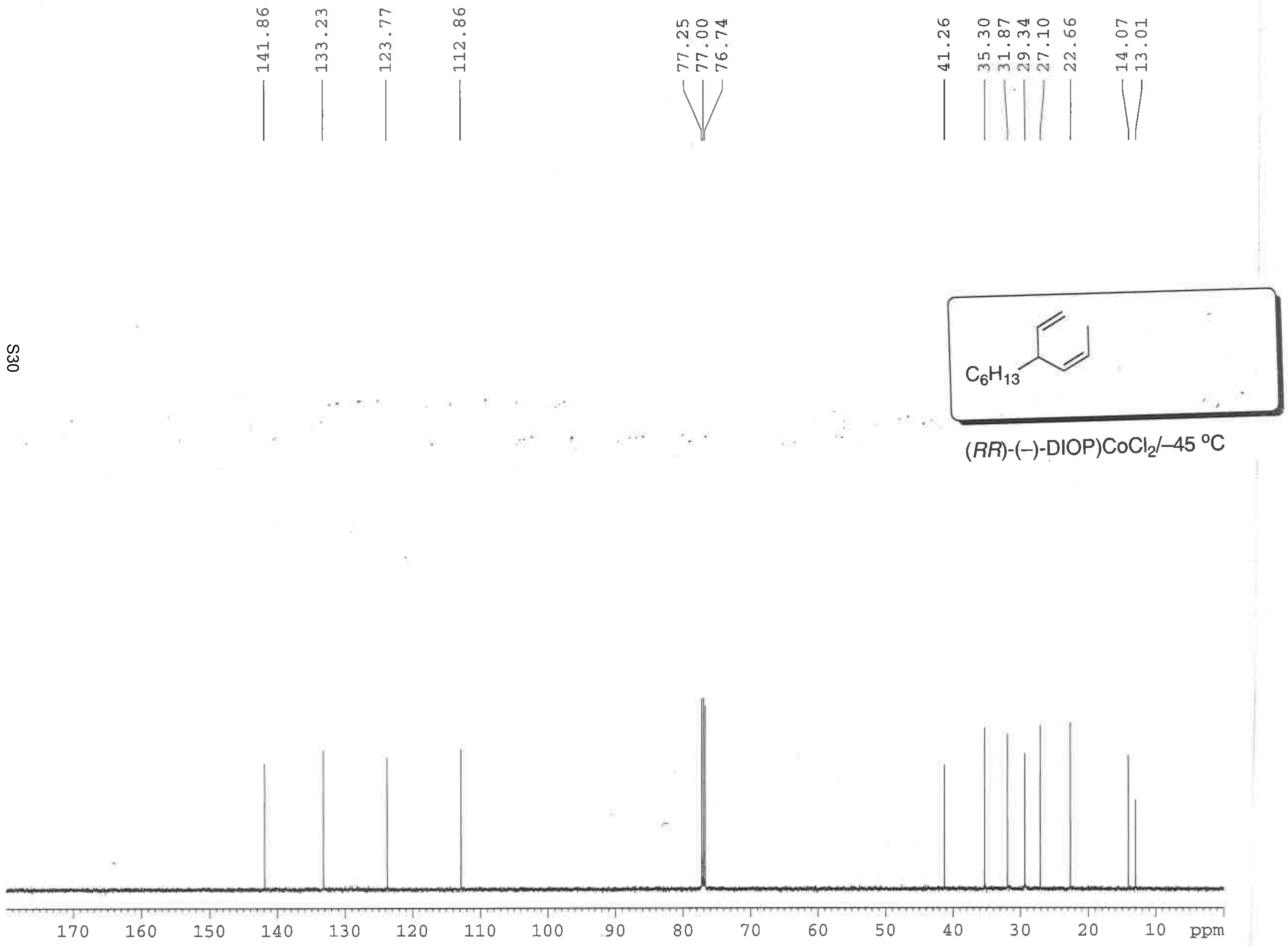

(*RR*)-(–)-DIOP)$CoCl_2$/–45 °C

Hydrovinylation of (*E*)-undeca-1,3-diene (12c)

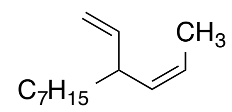

Hydrovinylation of (*E*)-undeca-1,3-diene using 1 mol % [L18 Josiphos-2]$CoCl_2$ Loading (Table 5, Entry 7). To an oven dried round bottom flask with a sidearm, was added [(**L18**)$CoCl_2$] (3 mg, 0.0028 mmol) and dichloromethane (0.5 mL) was added. Trimethylaluminium solution (2 M) (0.05 mL,0.008 mmol) was added to the solution and the color changed from deep blue to red brown with the formation of white fumes. After 2 to 3 minutes, the reaction vessel was carefully evacuated and then refilled with ethylene balloon. The reaction vessel was kept at -20 ^{o}C and (*E*)-undeca-1,3-diene (43 mg, 0.28 mmol), was added via syringe under ethylene and the mixture was stirred for 7 h. The ethylene balloon was removed and 0.1 mL methanol was added into the flask. The reaction solution was diluted with pentane (5 mL) and warmed to room temperature and subsequently passed through a silica plug. The plug was washed with pentane (3 x 6 mL). Removal of the solvent yielded the product as colorless oil. GC showed 97% (*Z*)-1,4-product and 3% 1,4-linear dimerized product.
Gas Chromatography (**13c**): CSP GC (Cyclodex-B, OT 100 ^{o}C/ isothermal, H_2 carrier gas). R_T for product = 50.0 min and 51.2 min (enantioselectivity 87 %ee).

[(*R,R*)-DIOP]$CoCl_2$ at -45 ^{o}C (6 h) gave the highest selectivity for this substrate (>95 %ee).[2]

13c using (dppb)CoCl2 (Table 3, entry 3)
methylsilicone column Oven temp 110 degrees

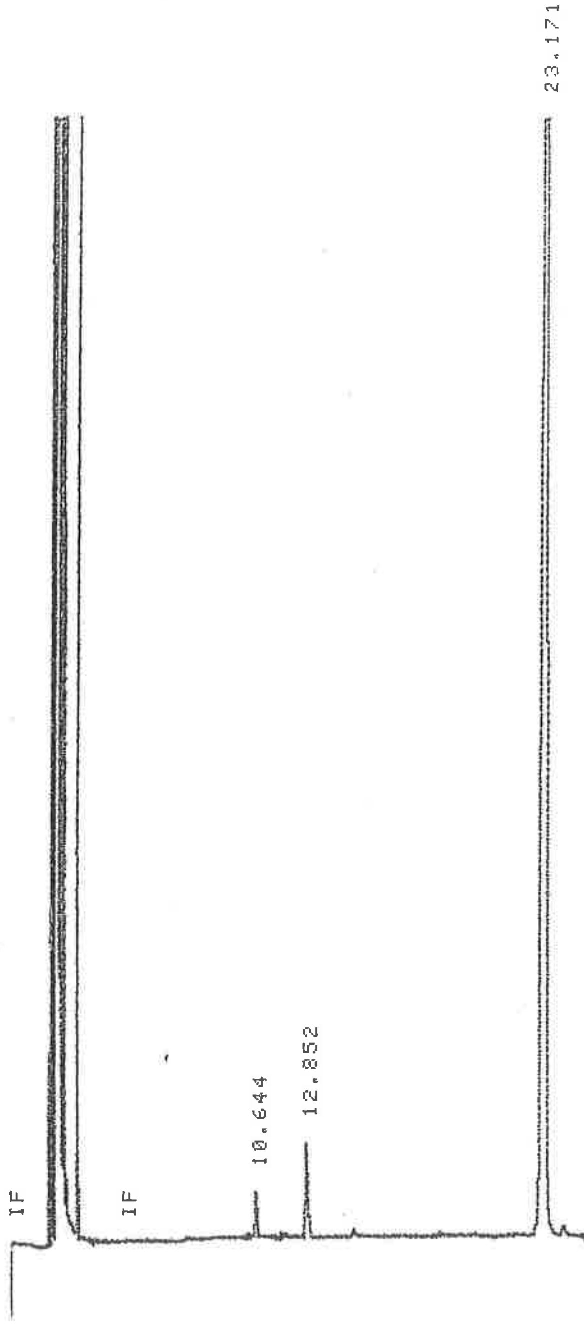

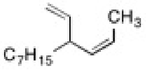

TIMETABLE STOP

Error storing signal to M:SIGNAL .RAW
ATTEMPTED WRITE PAST END OF FILE

RUN# 870 JUL 11, 1901 14:02:51

AREA%

RT	AREA	TYPE	WIDTH	AREA%
10.644	14868	PP	.092	.57381
12.852	30605	PV	.095	1.18116
23.171	2545624	BV	.179	98.24502

TOTAL AREA=2591098
MUL FACTOR=1.0000E+00

Racemic 13c using (dppb)CoCl2 (Table 3, entry 3)
cyclodex-B column oven temp 70 degrees/30 min,
increase to 85 degree at 0.7 degree per minute

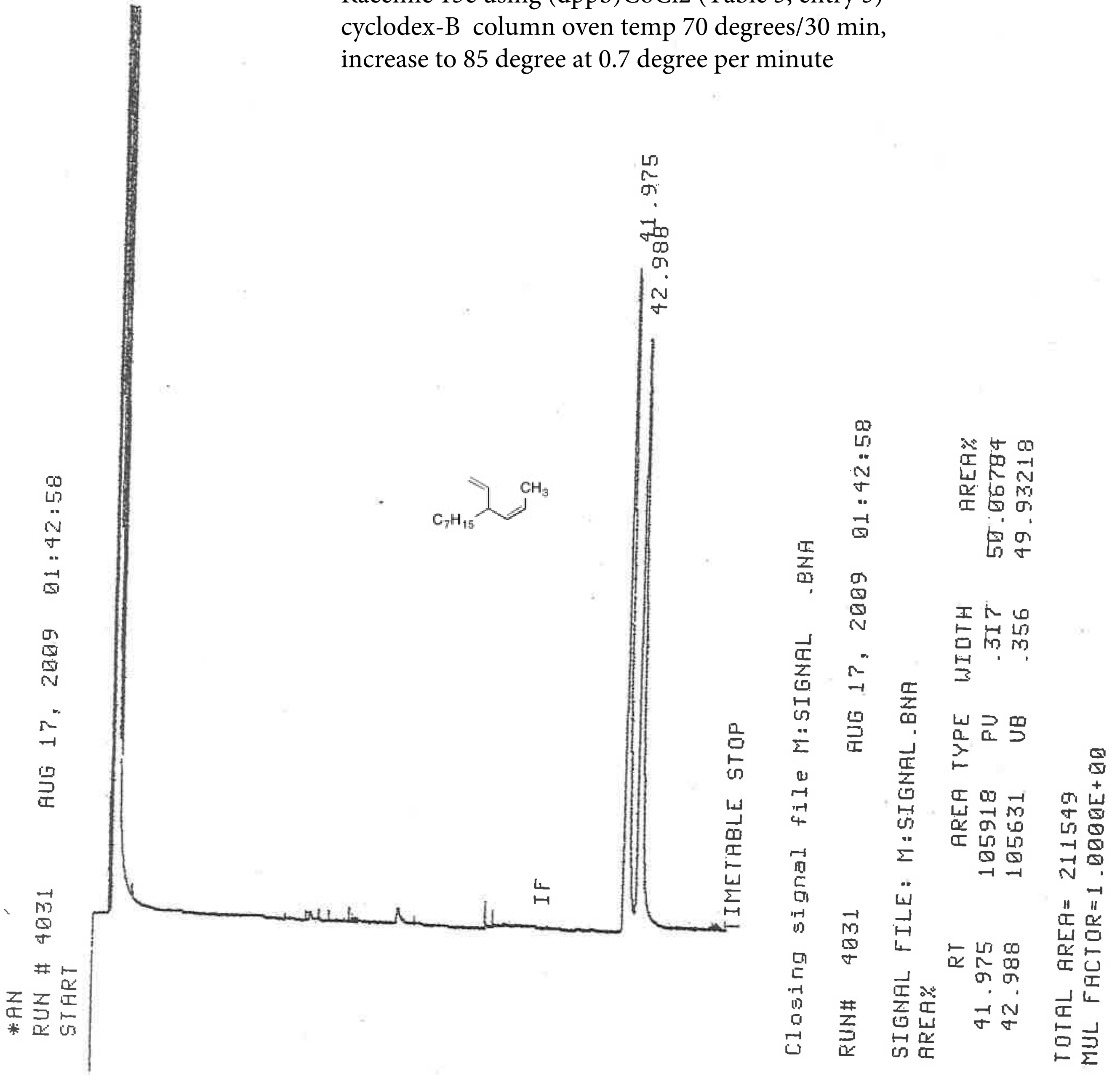

* RUN # 3956 JUN 26, 2009 23:38:42

START

13c using (RR)-DIOPCoCl2 at -45 degree (entry 6, Table 5) cyclodex-B column oven temp 70 degrees/30 min, increase to 85 degree at 0.7 degree per minute

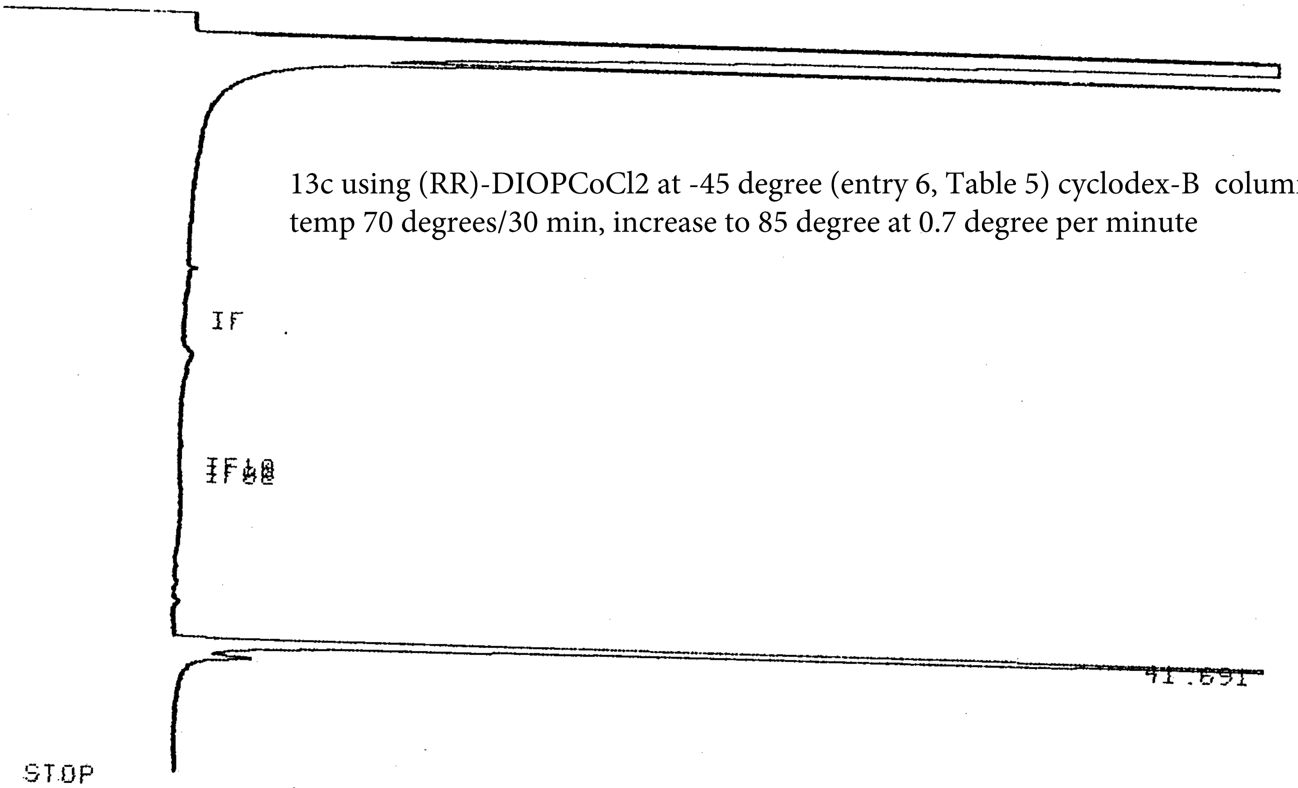

STOP

Closing signal file M:SIGNAL .BNC

RUN# 3956 JUN 26, 2009 23:38:42

SIGNAL FILE: M:SIGNAL.BNC

AREA%

RT	AREA	TYPE	WIDTH	AREA%
41.691	53419	BB	.303	97.72960
42.766	1241	PP	.255	2.27040

95.45% ee

TOTAL AREA= 54660

MUL FACTOR=1.0000E+00

GC (Cyclodex-B, 100 degree) of Compound **13c** (HV using Josiphos ligand **L18)** (entry 7, Table 5) 87% ee

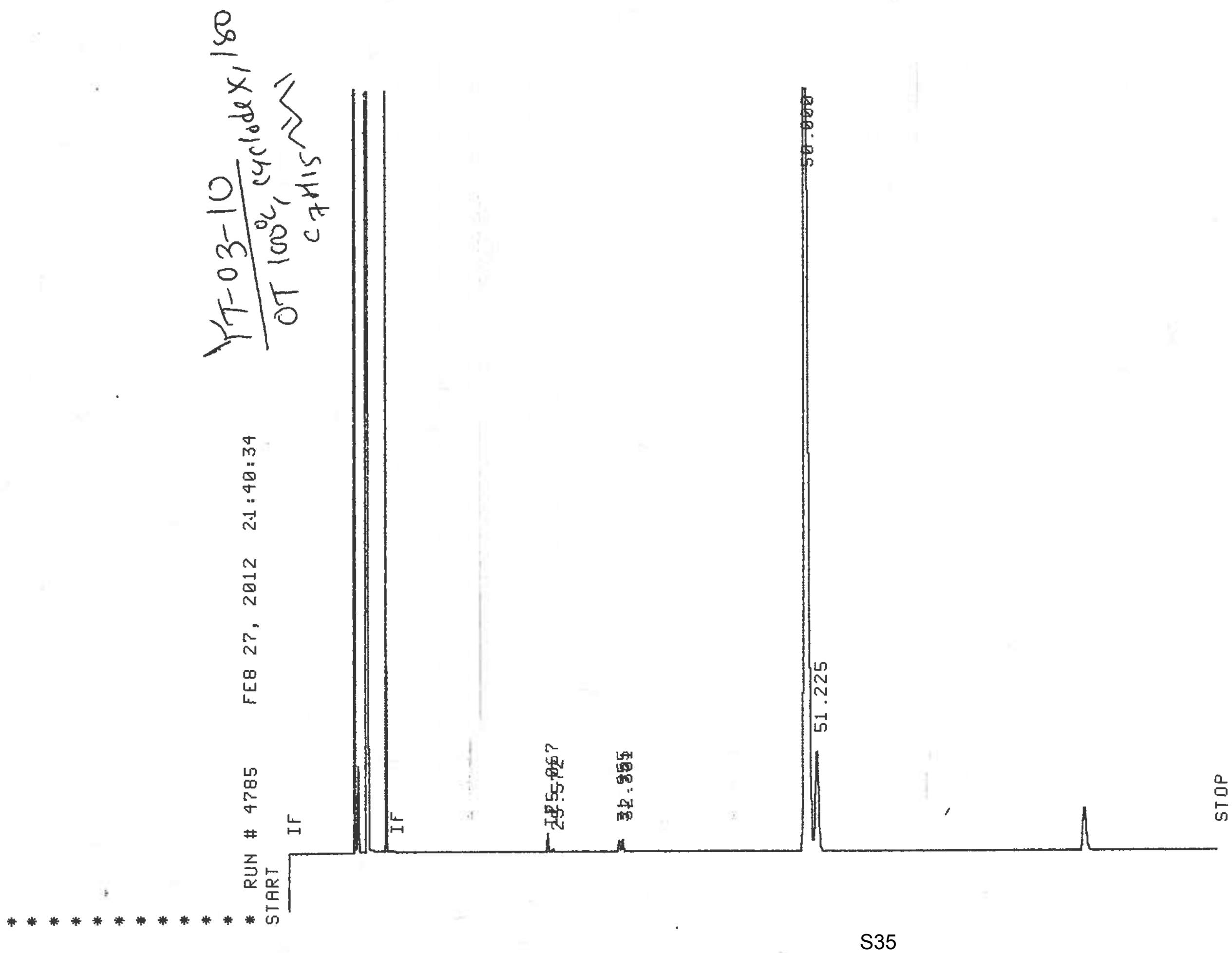

Closing signal file M:SIGNAL .BNC

RUN# 4785 FEB 27, 2012 21:40:34

SIGNAL FILE: M:SIGNAL.BNC
AREA%

RT	AREA	TYPE	WIDTH	AREA%
25.067	6762	VP	.133	.45712
25.572	1646	PB	.136	.11127
31.955	5030	BV	.164	.34004
32.301	5982	VB	.173	.40439
50.000	1321737	PV	.383	89.35142
51.225	93350	VB	.370	6.31060
77.168	44750	BB	.408	3.02517

TOTAL AREA=1479257
MUL FACTOR=1.0000E+00

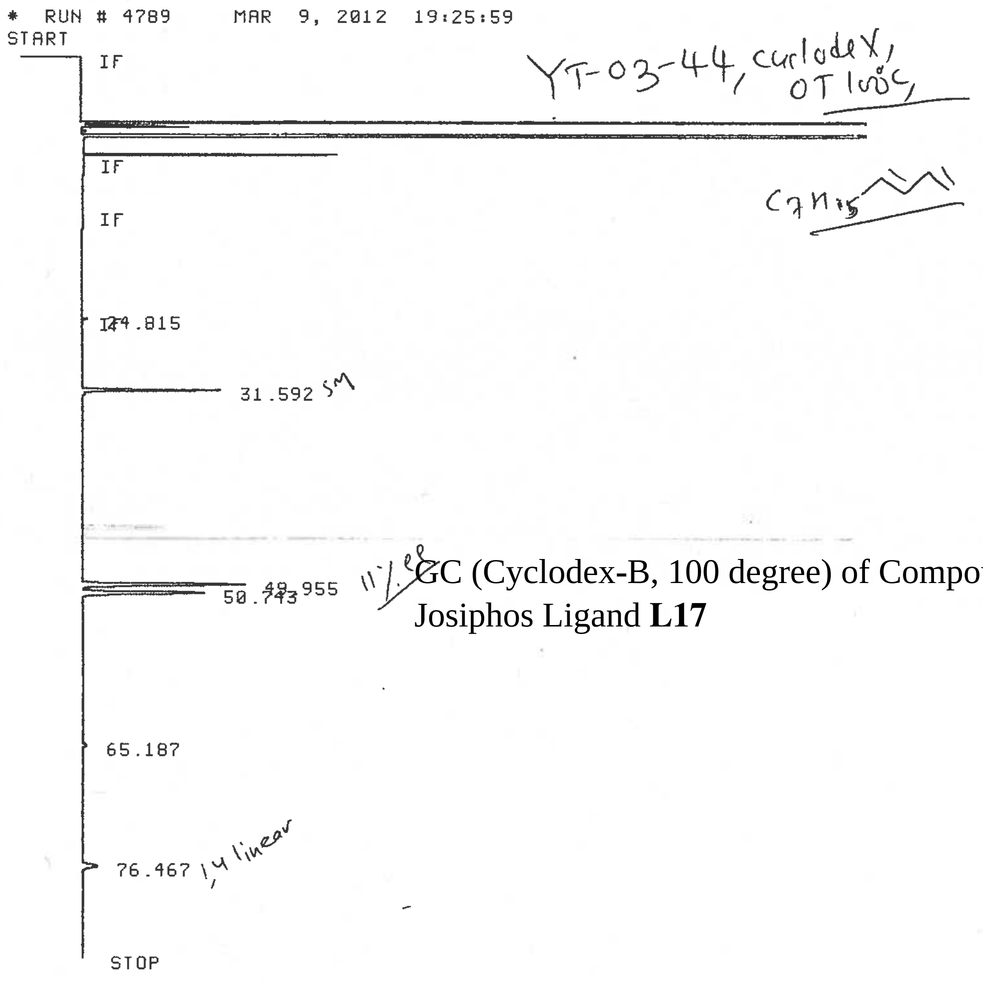

GC (Cyclodex-B, 100 degree) of Compound **13c** Using Josiphos Ligand **L17**

Closing signal file M:SIGNAL .BNC

RUN# 4789 MAR 9, 2012 19:25:59

SIGNAL FILE: M:SIGNAL.BNC
AREA%

RT	AREA	TYPE	WIDTH	AREA%
24.815	2064	PB	.128	.77326
31.592	57986	VV	.168	21.72403
49.955	103251	VV	.257	38.68224
50.743	82991	VB	.275	31.09197
65.187	4500	BB	.330	1.68589
76.467	16129	PB	.395	6.04261

TOTAL AREA= 266921
MUL FACTOR=1.0000E+00

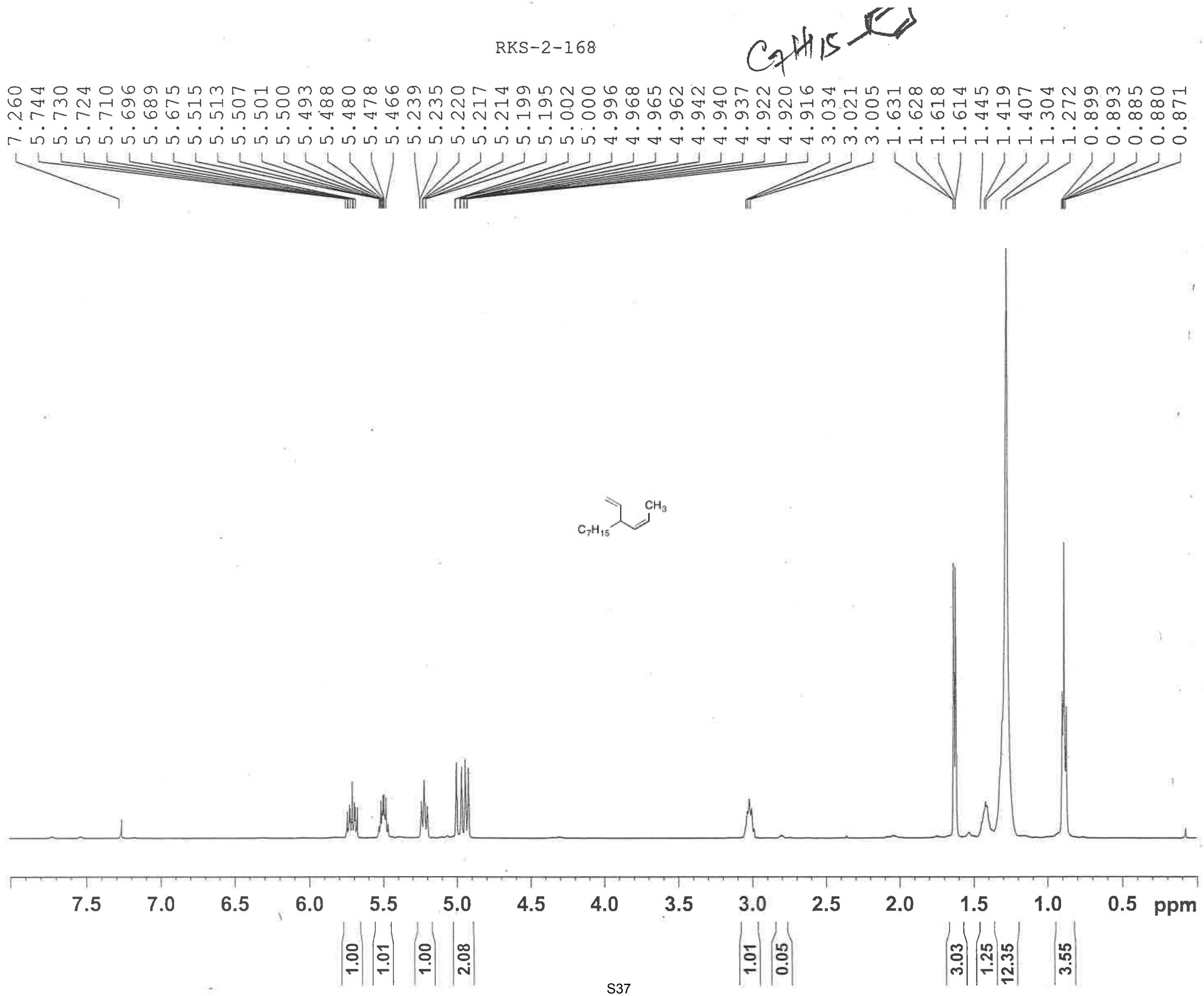
RKS-2-168
C7H15
7.260 5.744 5.730 5.724 5.710 5.696 5.689 5.675 5.515 5.513 5.507 5.501 5.500 5.493 5.488 5.480 5.478 5.466 5.239 5.235 5.220 5.217 5.214 5.199 5.195 5.002 5.000 4.996 4.968 4.965 4.962 4.942 4.940 4.937 4.922 4.920 4.916 3.034 3.021 3.005 1.631 1.628 1.618 1.614 1.445 1.419 1.407 1.304 1.272 0.899 0.893 0.885 0.880 0.871
CH3
C7H15
7.5 7.0 6.5 6.0 5.5 5.0 4.5 4.0 3.5 3.0 2.5 2.0 1.5 1.0 0.5 ppm
1.00 1.01 1.00 2.08 1.01 0.05 3.03 1.25 12.35 3.55

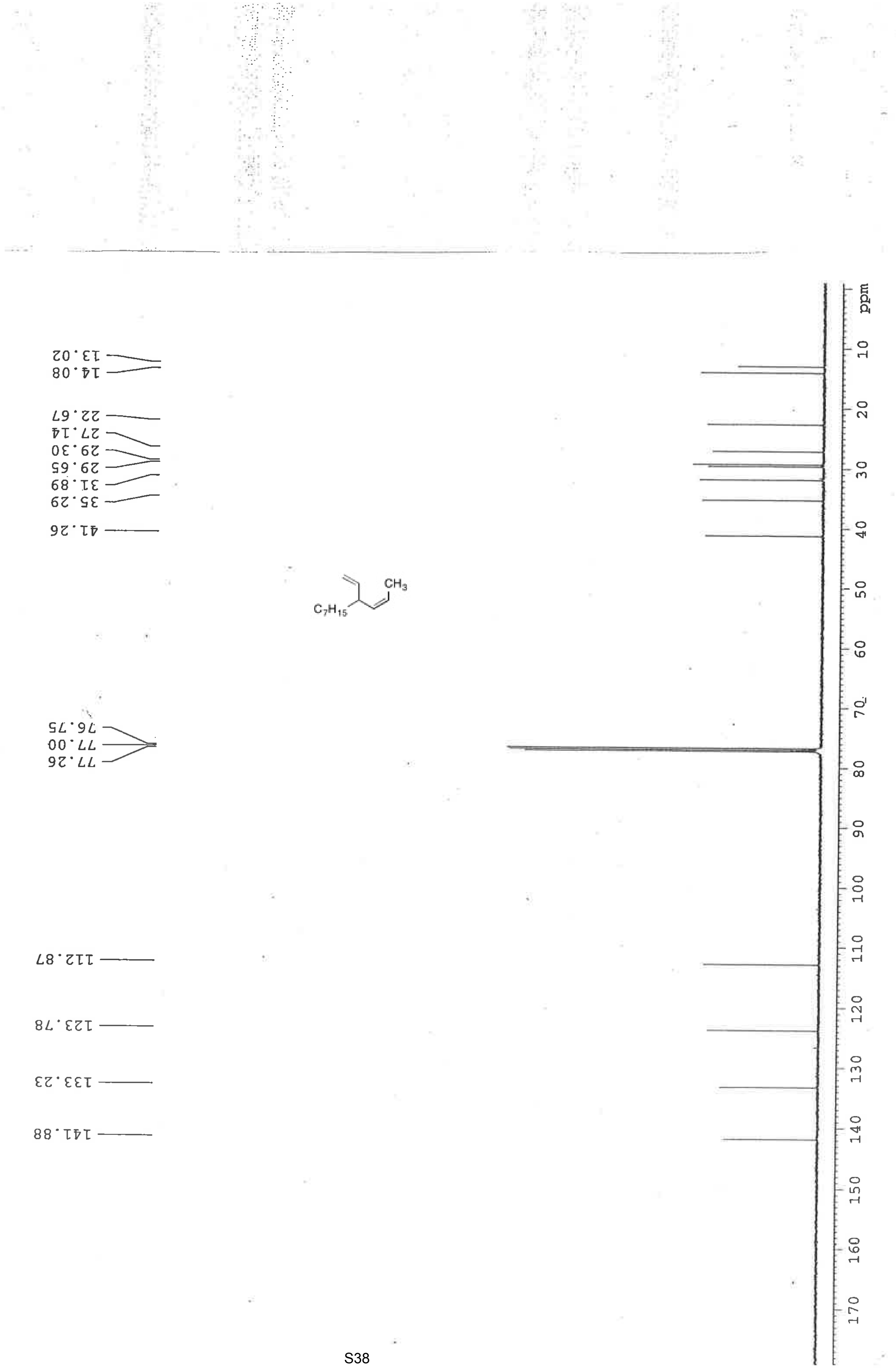
13.02
14.08
22.67
27.14
29.30
29.65
31.89
35.29
41.26
76.75
77.00
77.26
112.87
123.78
133.23
141.88
CH3
C7H15
ppm
10
20
30
40
50
60
70
80
90
100
110
120
130
140
150
160
170

Hydrovinylation of (*E/Z*)-dodeca-1,3-diene (12d)

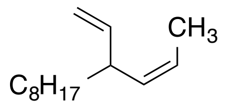

Hydrovinylation of (*E/Z*)-dodeca-1,3-diene using[DPPB]CoCl$_2$ (Table 3, Entry 5). To an oven dried round bottom flask with a sidearm, was added DPPBCoCl$_2$ (21 mg, 0.038 mmol) and methylaluminoxane (44 mg, 0.76 mmol) under argon. The color of the solution changed from deep blue to red brown with the formation of white fumes over the addition of dichloromethane solvent (1 mL). After 2 to 3 minutes, the reaction vessel was carefully evacuated and then refilled with ethylene balloon. The reaction vessel was kept at -10 ^oC and (*E/Z*)-dodeca-1,3-diene (63 mg, 0.38 mmol), mixture of *E*:*Z* in the ratio of 54:46 via GC, was added via syringe under ethylene and the mixture was stirred for 8 h. The ethylene balloon was removed and 0.1 mL methanol was added into the flask. The reaction solution was diluted with pentane (2 mL) and warmed to room temperature and subsequently passed through a silica plug. The plug was washed with pentane (3 x 5 mL). Removal of the solvent yielded the product as colorless oil (61 mg, 82%). GC and NMR analysis showed that the product was essentially pure. ^1H NMR (CDCl$_3$, 400 MHz): 0.836 (t, J = 7 Hz, 3 H), 1.25 – 1.43 (m, 14 H), 1.60 (dd, J = 7, 2 Hz, 3 H), 2.96 – 3.03 (m, 1 H), 4.89 - 4.99 (m, 2 H), 5.16 – 5.23 (m, 1 H), 5.43 - 5.51 (m, 1 H), 5.69 (ddd, J = 17, 10, 7 Hz, 1 H). ^{13}C NMR (CDCL$_3$, 125.02 MHz): 13.26, 14.33, 22.91, 27.37, 29.56, 29.83, 29.92, 32.14, 35.52, 41.49, 113.10, 124.01, 133.46, 142.10.
Gas Chromatography (**13d**): CSP GC (Cyclosil, OT 90 ^0C/ isothermal, H_2 carrier gas): starting material R_T = 31.19 min. for *E*-diene and 31.81 for *Z* diene. R_T for product = 51.74 min and 54.82 min (racemic). Cyclodex-B 65 ^oC/30 min increase 2 ^oC per min to 80 ^oC (He): R_T for product = 96.65 min and 99.14 min (racemic).

Hydrovinylation of (*E/Z*)-dodeca-1,3-diene using (*S,S*)[DIOP]CoCl$_2$ (Table 5, Entry 10): To an oven-dried round-bottom flask with a sidearm, was added (*SS*)-[DIOP]CoCl$_2$ (20 mg, 0.032 mmol) under argon and it was dissolved in a degassed dichloromethane (1 mL) at room temperature. Trimethylaluminum solution (2M) in toluene (0.08 mL, 0.16 mmol) was added dropwise as color of the solution changed from deep blue to red-brown with the formation of white fumes over the solution. When all the fumes disappeared, the reaction vessel was carefully evacuated and then refilled with ethylene from a balloon, a vigorous reaction with evolution of fumes was observed. This evolution stopped in typically within 4 minutes. The reaction vessel was cooled to – 45 ^oC and (*E/Z*)-dodeca-1,3-diene (53 mg, 0.32 mmol, *E*:*Z* = 54:46) was added under ethylene and the mixture was stirred for 1 h until the completion of the reaction monitored by gas chromatography. The ethylene balloon was removed and 0.1 mL of methanol was introduced into the flask and stirring was continued for 5 minutes. The solution was warmed to room temperature and was subsequently passed through a silica plug. The plug was washed with pentane (3 X 10 mL). Pentane was removed and product was separated as a colorless oil as a major product 1,4-Z hydrovinylation product, **13d** (53% yield) with 74% ee. Isomeric compositions were determined by gas chromatography and NMR spectroscopy. ^1H NMR (CDCl$_3$, 400 MHz): 0.836 (t, J = 7 Hz, 3 H), 1.25 – 1.43 (m, 14 H), 1.60 (dd, J = 7, 2 Hz, 3 H), 2.96 – 3.03 (m, 1 H), 4.89 - 4.99 (m, 2 H), 5.16 – 5.23 (m, 1 H), 5.43 - 5.51 (m, 1 H), 5.69 (ddd, J = 17, 10, 7 Hz, 1 H). ^{13}C NMR (CDCL$_3$, 125.02 MHz): 13.26, 14.33, 22.91, 27.37, 29.56, 29.83, 29.92, 32.14, 35.52, 41.49, 113.10, 124.01, 133.46, 142.10.
Hydrovinylation of (*E*)-dodeca-1,3-diene using(*S,S*)[DIOP]CoCl$_2$ at -45 ^oC (6 h) gave the highest ee for this substrate (>96 %ee, Table 5, Entry 8).[2]

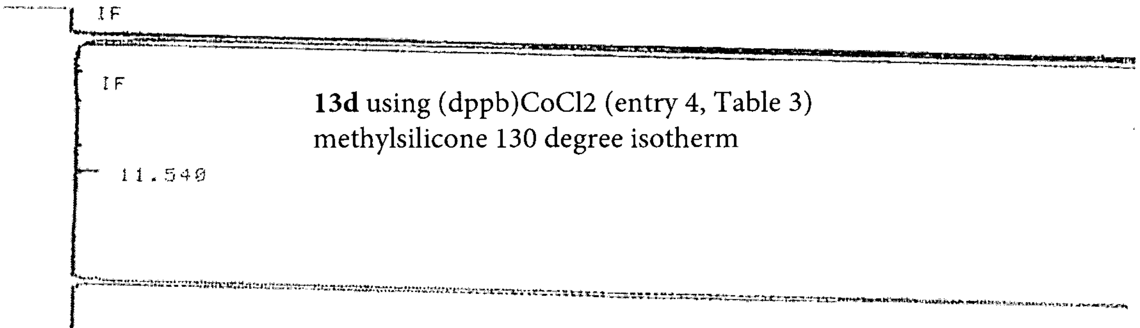

TIMETABLE STOP

Error storing signal to M:SIGNAL .RAW
ATTEMPTED WRITE PAST END OF FILE

RUN# 861 JUL 9, 1901 16:15:36

AREA%

RT	AREA	TYPE	WIDTH	AREA%
19.265	1327140	VV	.144	100.00000

TOTAL AREA=1327140
MUL FACTOR=1.0000E+00

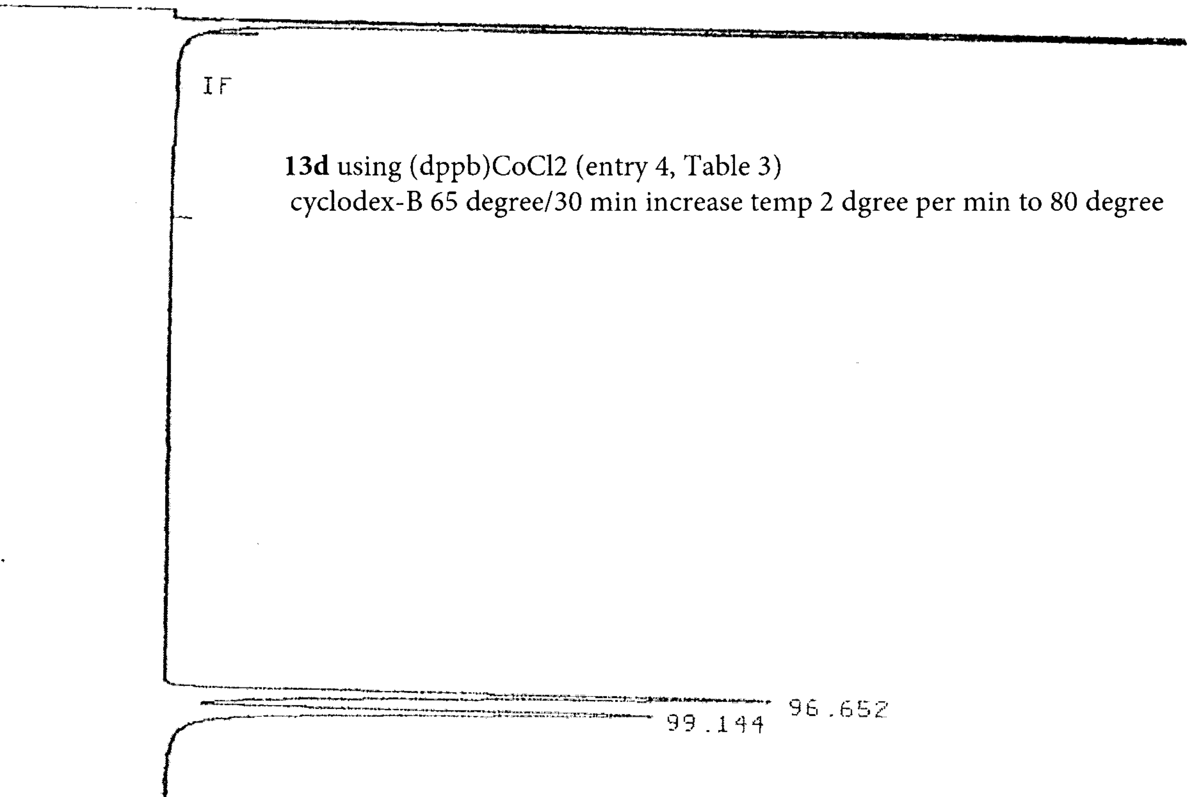

END OF SIGNAL

Closing signal file M:SIGNAL .BNA

RUN# 4040 AUG 20, 2009 23:25:01

SIGNAL FILE: M:SIGNAL.BNA
AREA%

RT	AREA	TYPE	WIDTH	AREA%
96.652	116697	BV	.896	51.77582
99.144	108692	VB	1.034	48.22418

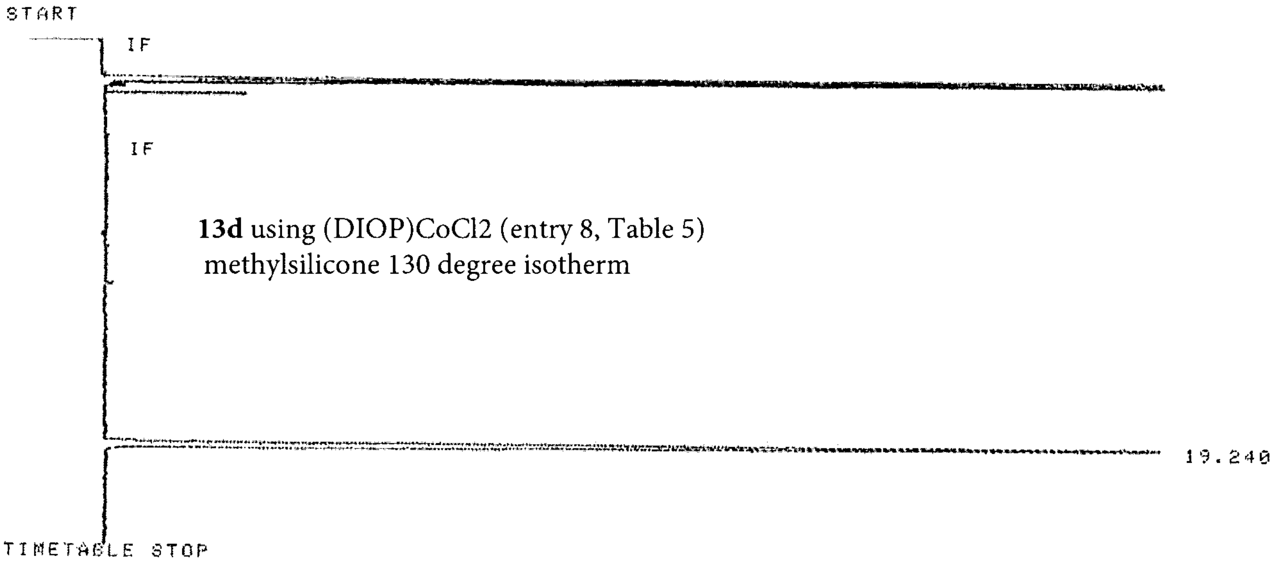

13d using (DIOP)CoCl2 (entry 8, Table 5)
methylsilicone 130 degree isotherm

Error storing signal to M:SIGNAL .RAW
ATTEMPTED WRITE PAST END OF FILE

RUN# 863 JUL 10, 1901 12:19:24

AREA%

RT	AREA	TYPE	WIDTH	AREA%
19.240	824759	PP	.145	100.00000

TOTAL AREA= 824759
MUL FACTOR=1.0000E+00

*
*
*
*
*
*
*
*
*

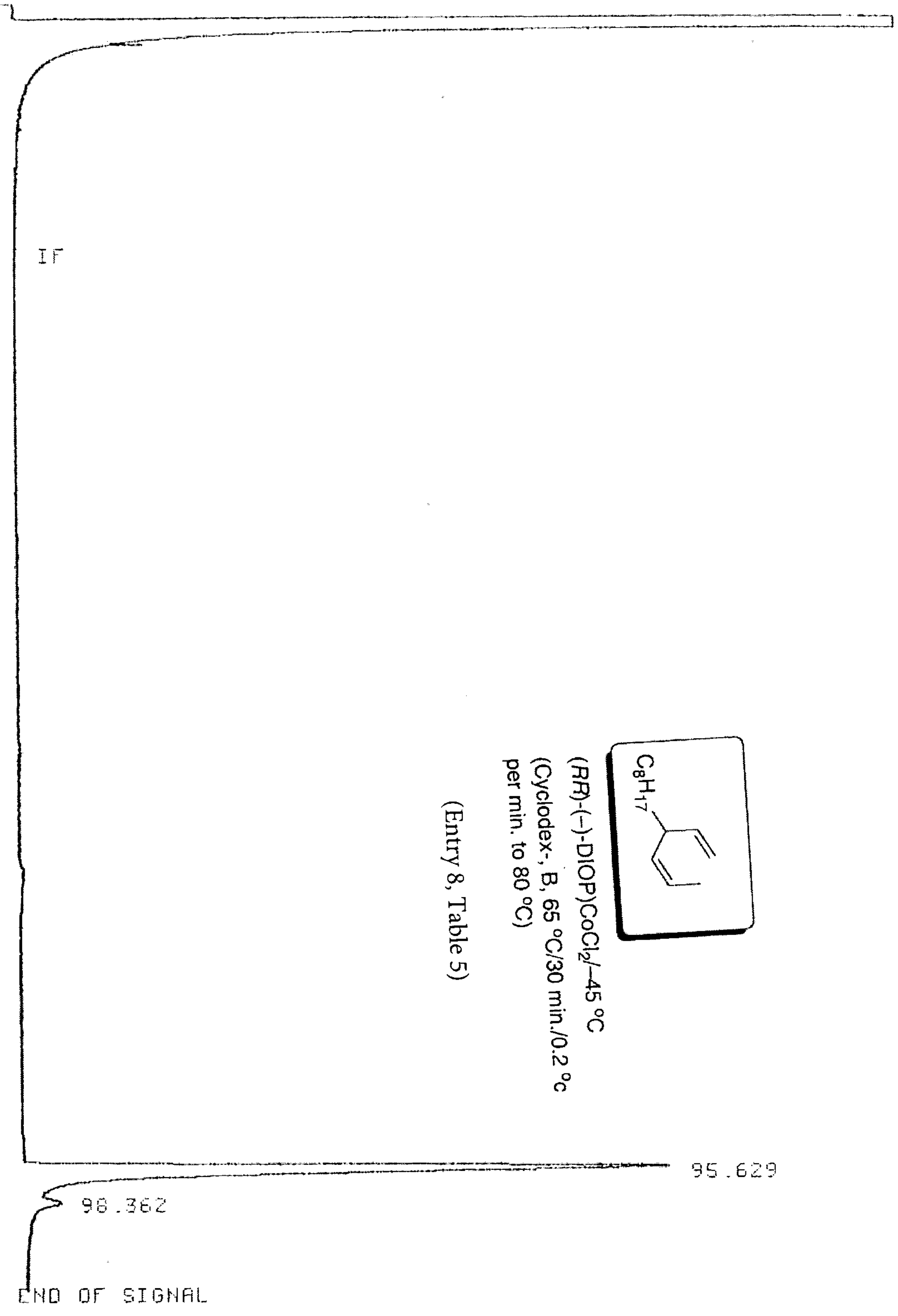

Closing signal file M:SIGNAL .BNA

RUN# 3973 JUN 30, 2009 02:46:41

SIGNAL FILE: M:SIGNAL.BNA
AREA%

RT	AREA	TYPE	WIDTH	AREA%
95.629	132930	PV	.794	99.11424
98.362	1188	VV	.189	.88579

TOTAL AREA= 134118
MUL FACTOR=1.0000E+00

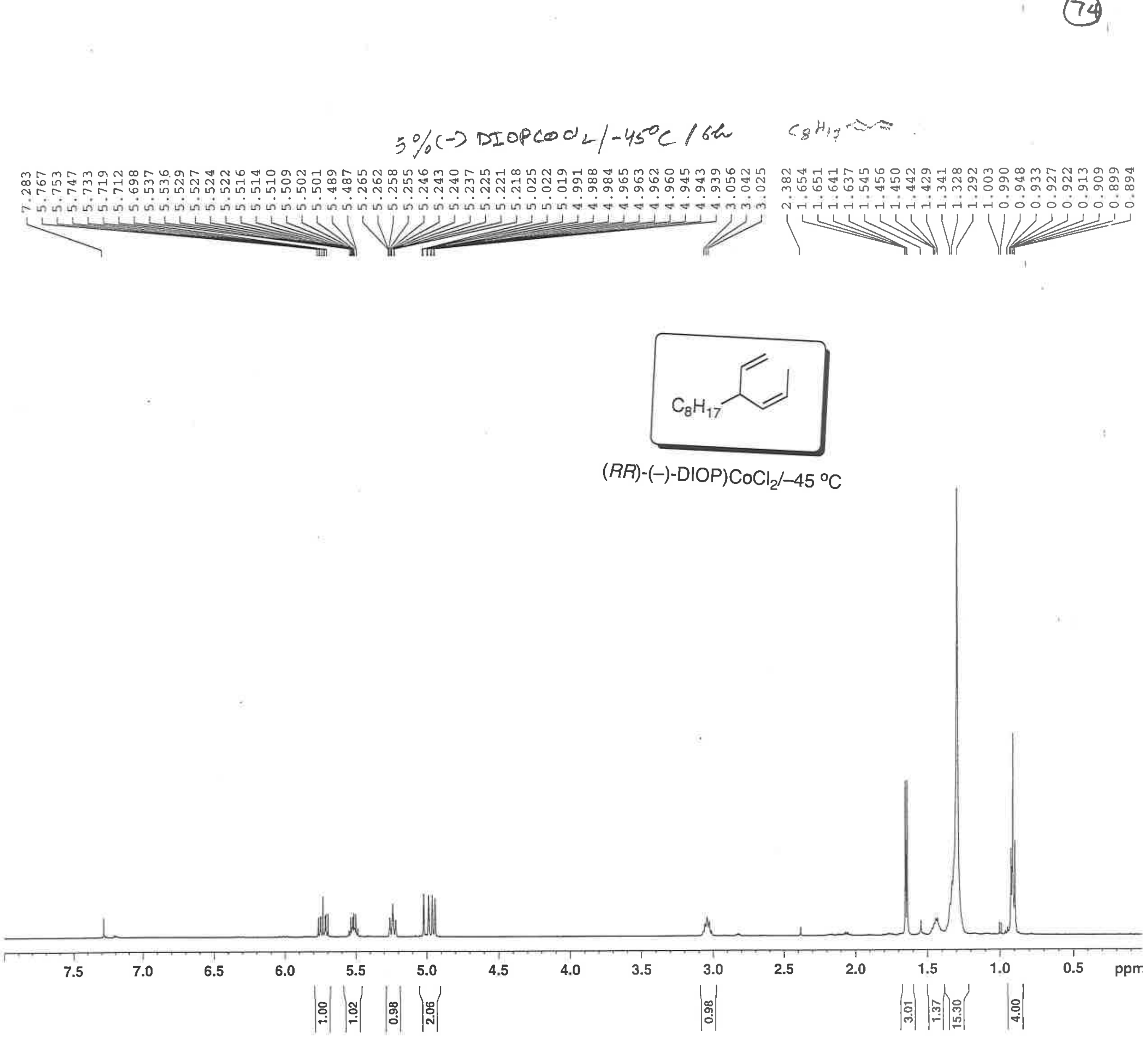
(74)
5%(-) DIOPCoCl2/-45°C / 6h
C8H17
7.283 5.767 5.753 5.747 5.733 5.719 5.712 5.698 5.537 5.536 5.529 5.527 5.524 5.522 5.516 5.514 5.510 5.509 5.502 5.501 5.489 5.487 5.265 5.262 5.258 5.255 5.246 5.243 5.240 5.237 5.225 5.221 5.218 5.025 5.022 5.019 4.991 4.988 4.984 4.965 4.963 4.962 4.960 4.945 4.943 4.939 3.056 3.042 3.025 2.382 1.654 1.651 1.641 1.637 1.545 1.456 1.450 1.442 1.429 1.341 1.328 1.292 1.003 0.990 0.948 0.933 0.927 0.922 0.913 0.909 0.899 0.894
C8H17
(RR)-(–)-DIOP)CoCl2/–45 °C
7.5 7.0 6.5 6.0 5.5 5.0 4.5 4.0 3.5 3.0 2.5 2.0 1.5 1.0 0.5 ppm
1.00 1.02 0.98 2.06 0.98 3.01 1.37 15.30 4.00

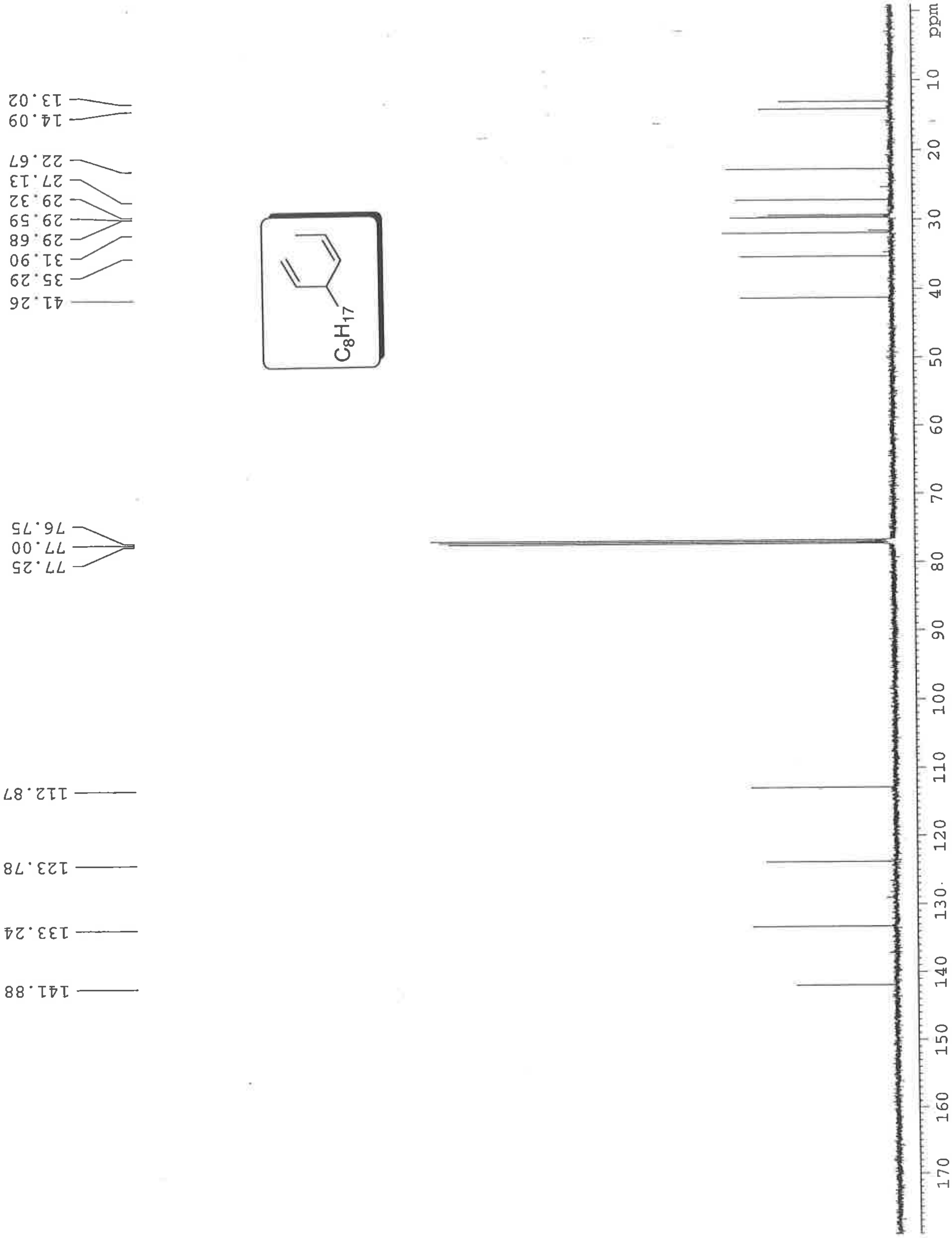

13.02
14.09
22.67
27.13
29.32
29.59
29.68
31.90
35.29
41.26
76.75
77.00
77.25
112.87
123.78
133.24
141.88
C_8H_{17}
ppm

Hydrovinylation of (*E/Z*)-1-cyclohexyl-1,3-butadiene

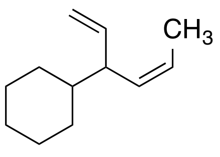

Hydrovinylation of (*E/Z*)-1-cyclohexyl-1,3-butadiene using[DPPB]$CoCl_2$ (Table 3, Entry 6): To an oven dried round bottom flask with a sidearm, was added DPPB$CoCl_2$ (30 mg, 0.053 mmol) and methylaluminoxane (62 mg, 1.06 mmol) under argon. The color of the solution changed from deep blue to red brown with the formation of white fumes over the addition of dichloromethane solvent (1 mL). After 2 to 3 minutes, the reaction vessel was carefully evacuated and then refilled with ethylene balloon. The reaction vessel was kept at -10 ^{o}C and (*E/Z*)-1-cyclohexyl-1,3-butadiene (72 mg, 0.53mmol), mixture of *E*:*Z* in the ratio of 45:55, was added via syringe under ethylene and the mixture was stirred for 8 h. The ethylene balloon was removed and 0.1 mL methanol was added into the flask. The reaction solution was diluted with pentane (2 mL) and warmed to room temperature and subsequently passed through a silica plug. The plug was washed with pentane (3 x 5 mL). Removal of the solvent yielded the product as colorless oil (78 mg, 90%). GC and NMR analysis showed that the product (**13e**) was essentially pure. ^{1}H NMR ($CDCl_3$, 400 MHz): 1.07 - 1.26 (m, 6 H), 1.58 (dd, J = 1.6, 7 Hz, 3 H), 1.62 - 1.73 (m, 5 H), 2.79 (q, J = 8 Hz, 1 H), 4.91 - 4.91 (m, 1 H), 4.94 – 4.96 (m, 1 H), 5.24 - 5.30 (m, 1 H), 5.45 - 5.33 (m, 1 H), 5.62 – 5.71 (m, 1 H). ^{13}C NMR ($CDCL_3$, 125.02 MHz): 13.11, 26.48, 26.54, 26.64, 30.29, 30.95, 42.16, 47.75, 113.82, 124.02, 131.75, 140.55. Gas Chromatography: Methyl silicone SP GC (OT 90 ^{o}C/ isothermal) R_t for product = 17.05 min. CSP GC (Cyclodex, OT 100 ^{o}C/ isothermal, He carrier gas): starting material R_T= 25.28 min. for Z-diene and 29.84 for E diene. R_T for product = 45.19 min and 46.35 min (enantiomers, racemic). CSP GC (Cyclosil, OT 80 ^{o}C/ isothermal) R_T for product = 34.52 min and 37.04 min (enantiomers, racemic).

An enantioselectivity of 84 %ee was obtained with (*S,S*)-[DIOP]$CoCl_2$ (at -10 ^{o} C, 8 h) with only the *E*-diene undergoing the conversion. With this ligand, when both *E* and *Z* are converted, maximum enantioselectivity is only 15%. With (*R,R*)-[BDPP]$CoCl_2$ and *E* and *Z* full conversion, maximum enantioselectivity is 35% [determined by CSP GC).

The following table shows the detail of HV reactions using (*E*/*Z*)-mixture of 1-cyclohexyl-1,3-diene using various ligands.

Entry	Ligand	Temp (^{o}C) /Time (h)	Activator	Conv.of isomer shown (%)	Product
1.	[DPPM]	-28/7	MAO	49 (*E* & *Z*)	Mixture of 1,2 and 1,4-HV
2.	(*S,S*)-[DIOP]	-15/14	MAO	98% (only *E*)	1,4- (*Z*)- HV, 84% ee.
3.	(*S,S*)-[DIOP]	-10/8	MAO	100% (*E* & *Z*)	1,4- (*Z*)- HV, 15% ee
4.	Josiphos 1 **L17**	-10/12	MAO	100% (only *E*)	1,4- (*Z*)- HV, 34% ee
5.	Josiphos 2 **L18**	-10/8	TMA	80% (only *E*)	1,4- (*Z*)- HV, 64% ee
6.	(*R,R*)-[BDPP]	-10/11	MAO	100% (*E* & *Z*)	1,4- (*Z*)- HV(78%), 35% ee, linear(21%)

Unsuccessful hydrovinylation of (only *Z*)-1-cyclohexyl-1,3-butadiene using (*S,S*)[DIOP]CoCl$_2$ and TMA (Table 5, Entry 11, but only *Z*-isomer). To an oven dried round bottom flask with a side-arm, was added (*S,S*)[DIOP]CoCl$_2$ (15 mg, 0.024 mmol) dissolved in dichloromethane (1 mL) at room temperature. Trimethylaluminium as a 2M solution in toluene (0.03 mL, 0.072 mmol) was added dropwise and the color of the solution changed from deep blue to red brown with the formation of white fumes over the solution. After 2 to 3 minutes, the reaction vessel was carefully evacuated and then refilled with ethylene from balloon. The reaction vessel was cooled to -12 ^{o}C and (*Z*)-1-cyclohexyl-1,3-butadiene (32 mg, 0.23 mmol) added under ethylene and the mixture was stirred for 12 h at -12 ^{o}C. The ethylene balloon was removed and 0.1 mL methanol was added into the flask. The reaction solution was diluted with pentane (5 mL) and warmed to room temperature and subsequently passed through a silica plug very quickly. The plug was washed with pentane (3 x 6 mL). Removal of solvent was difficult so GC and NMR analysis showed that the product was complex mixture of many unidentified different compounds along with starting material.

*
*
*
*
*
* RUN # 348 APR 12, 1901 01:23:22
START
IF

GC (HP-5, methyl silicone, 90 degree) of Compound **13e (HV** using Cl2Co(DPPB) (entry 6, Table 3)

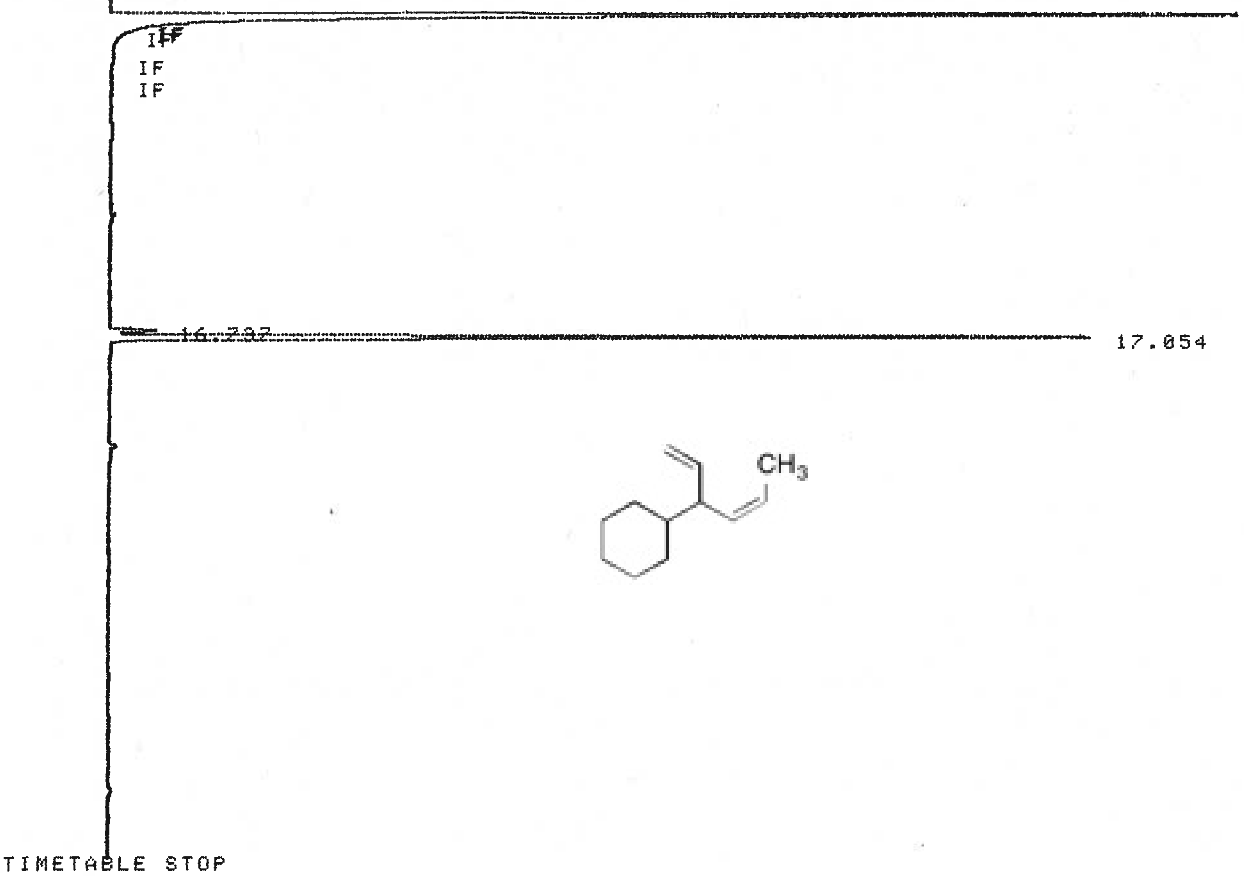

TIMETABLE STOP

RUN# 348 APR 12, 1901 01:23:22

AREA%

RT	AREA	TYPE	WIDTH	AREA%
16.737	22651	PV	.140	4.48270
17.054	482647	VB	.145	95.51731

TOTAL AREA= 505298
MUL FACTOR=1.0000E+00

*

Data File C:\CHEM32\1\DATA\YAM\CC-CHIRAL 2013-02-22 11-30-24\YT-04-20RE.D
Sample Name: YT-04-20RE.D

===

Acq. Operator : YAM   Seq. Line : 1
Acq. Instrument : Babu   Location : Vial 201
Injection Date : 2/22/2013 11:31:29 AM   Inj : 1
Inj Volume : 1 µl
Different Inj Volume from Sequence !   Actual Inj Volume : 3 µl
Acq. Method : C:\CHEM32\1\DATA\YAM\CC-CHIRAL 2013-02-22 11-30-24\YT_85_C_CHIRAL.M
Last changed : 2/22/2013 10:09:50 AM by YAM
Analysis Method : C:\CHEM32\1\METHODS\YT_85_C_CHIRAL.M
Last changed : 2/22/2013 12:23:56 PM by YAM
(modified after loading)

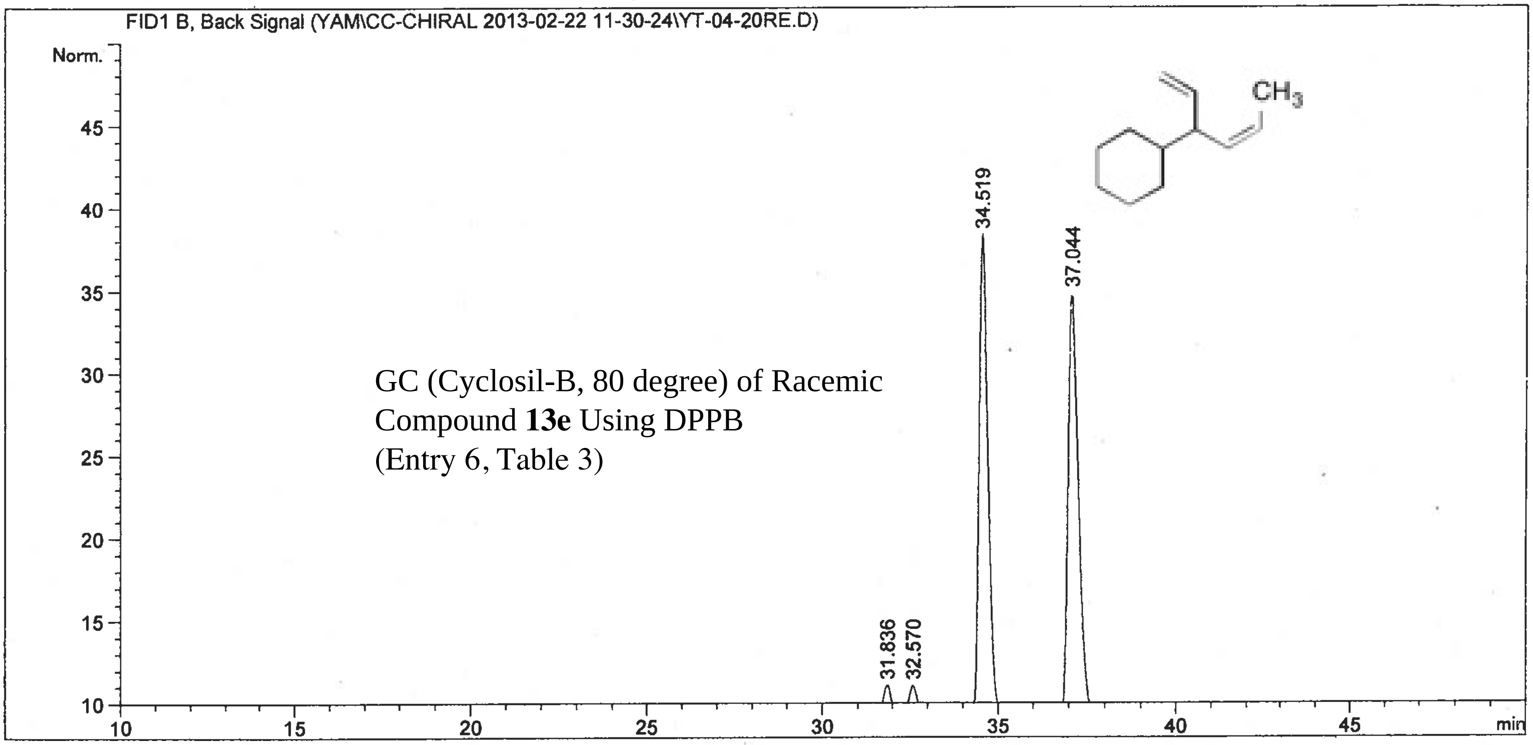

===

Area Percent Report

===

Sorted By : Signal
Multiplier: : 1.0000
Dilution: : 1.0000
Use Multiplier & Dilution Factor with ISTDs

Signal 1: FID1 B, Back Signal

Peak #	RetTime [min]	Type	Width [min]	Area [pA*s]	Height [pA]	Area %
1	31.836	BB	0.2275	24.61501	1.67404	2.32915
2	32.570	BB	0.2370	25.28156	1.65907	2.39223
3	34.519	BB	0.2568	502.73248	28.98533	47.57022
4	37.044	BB	0.2822	504.19278	25.46459	47.70840

*
*
*
*
* RUN # 350 APR 12, 1901 04:39:12
START

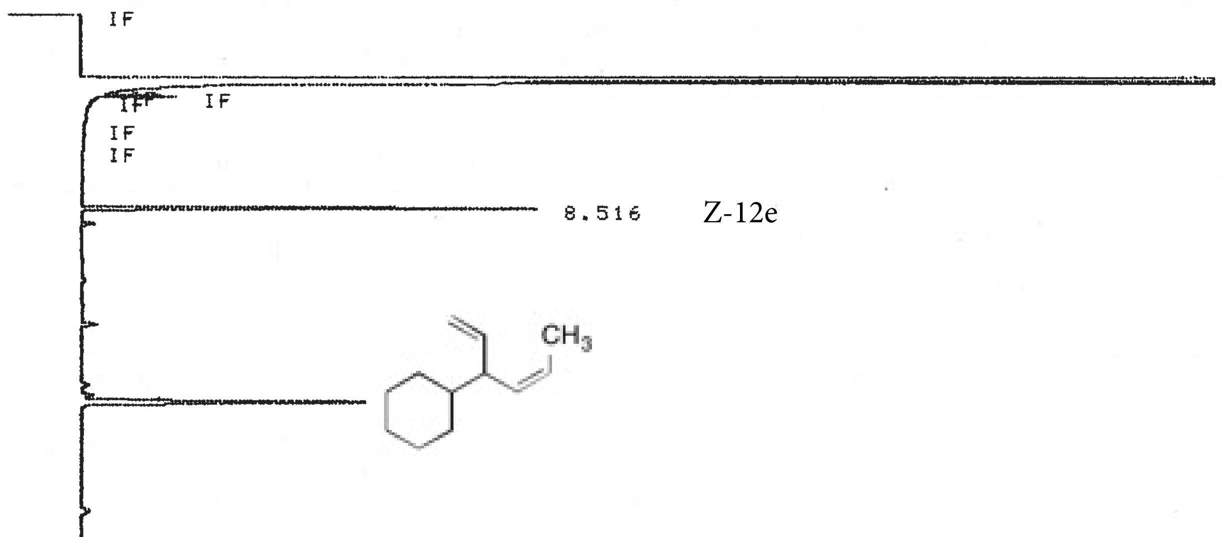

GC (HP-5, methyl silicone, 90 degree) of mixture of **13e** and Z-**12e** (Entry 11, Table 5)

TIMETABLE STOP

RUN# 350 APR 12, 1901 04:39:12

AREA%

RT	AREA	TYPE	WIDTH	AREA%
8.516	117127	PB	.075	45.38170
17.052	140966	VB	.145	54.61829

TOTAL AREA= 258093
MUL FACTOR=1.0000E+00

Data File C:\CHEM32\1\DATA\YAM\CC-CHIRAL 2013-02-22 14-46-19\YT-04-14RE.D
Sample Name: YT-04-14RE.D

===

Acq. Operator : YAM   Seq. Line : 1
Acq. Instrument : Babu   Location : Vial 201
Injection Date : 2/22/2013 2:47:24 PM   Inj : 1
                                                     Inj Volume : 1 µl
Different Inj Volume from Sequence ! Actual Inj Volume : 3 µl
Acq. Method : C:\CHEM32\1\DATA\YAM\CC-CHIRAL 2013-02-22 14-46-19\YT_85_C_CHIRAL.M
Last changed : 2/22/2013 2:46:17 PM by YAM
Analysis Method : C:\CHEM32\1\METHODS\YT_85_C_CHIRAL.M
Last changed : 2/22/2013 2:46:17 PM by YAM

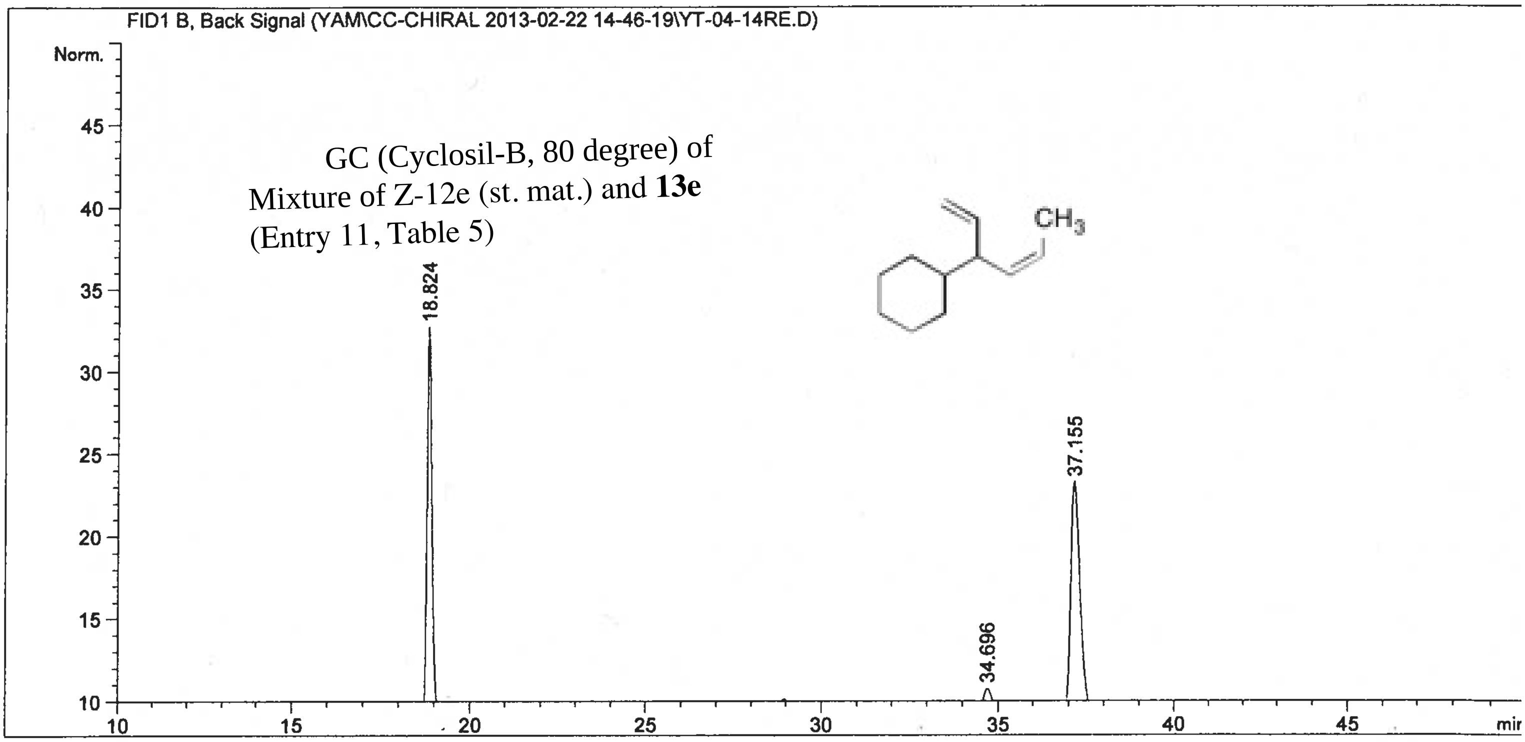

===

Area Percent Report

===

Sorted By : Signal
Multiplier: : 1.0000
Dilution: : 1.0000
Use Multiplier & Dilution Factor with ISTDs

Signal 1: FID1 B, Back Signal

Peak #	RetTime [min]	Type	Width [min]	Area [pA*s]	Height [pA]	Area %
1	18.824	BB	0.1434	222.28169	23.27076	44.91272
2	34.696	BB	0.2530	21.26893	1.31316	4.29745
3	37.155	BB	0.2727	251.36865	13.84104	50.78983

Totals : 494.91927 38.42496

Proton NMR (400 MHz, D-chloroform) spectrum of Compound **13e** (HV using DPPB) (entry 6, Table 3)

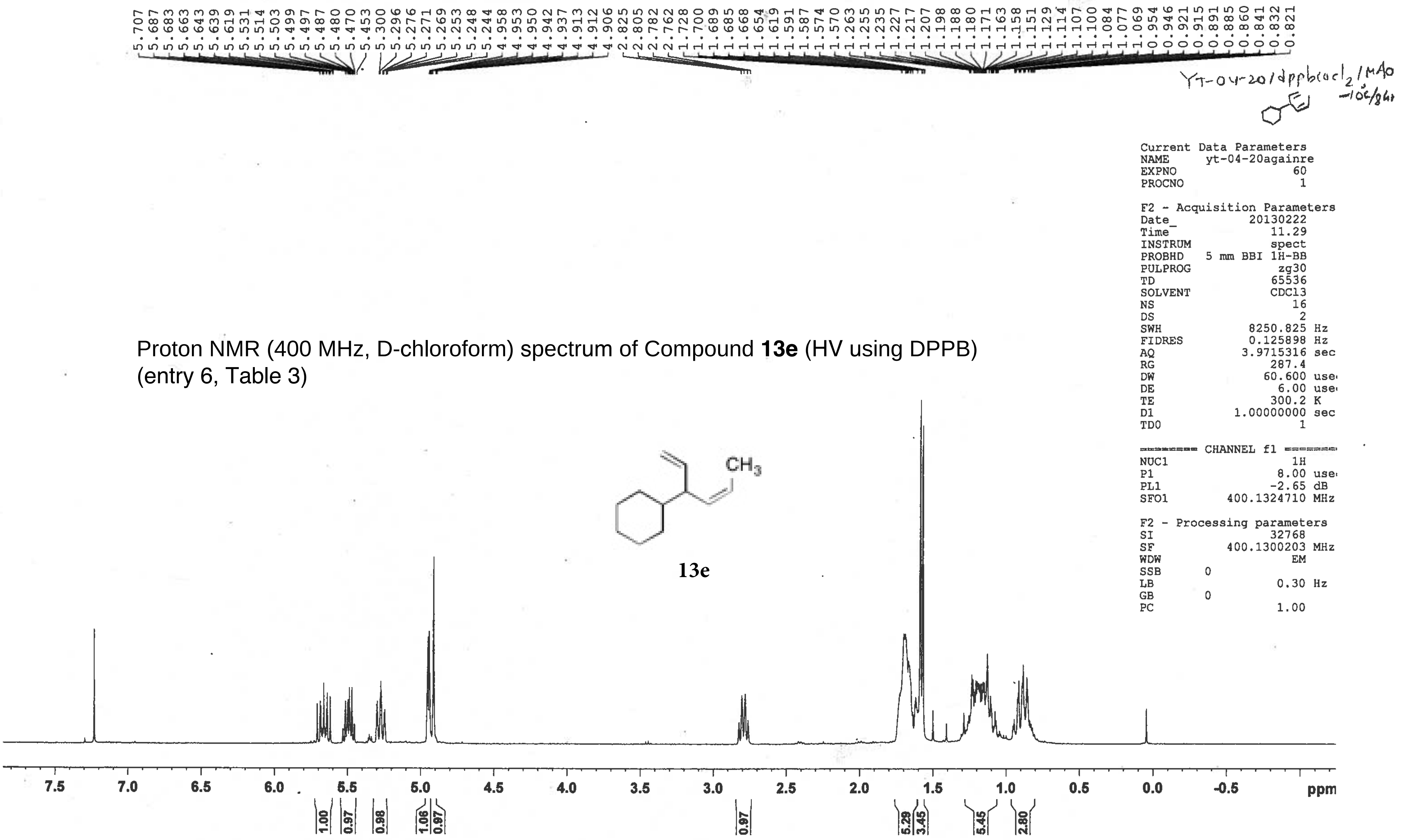

13e

Proton NMR (400 MHz, D-chloroform) spectrum of compound **13e** (HV using DPPB, Alkene Region Expansion)

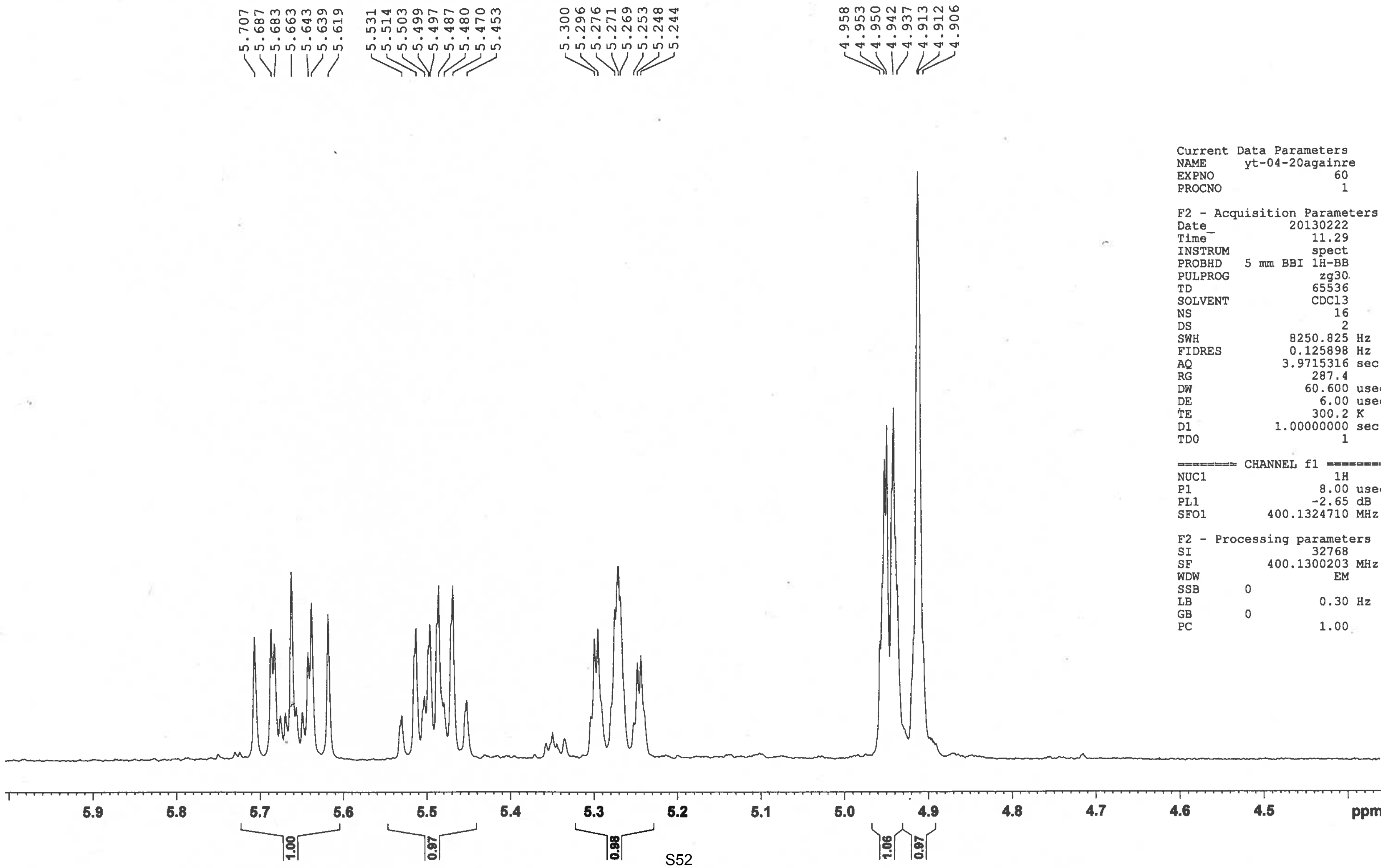

Carbon NMR (100 MHz, D-chloroform) spectrum of Compound **13e**

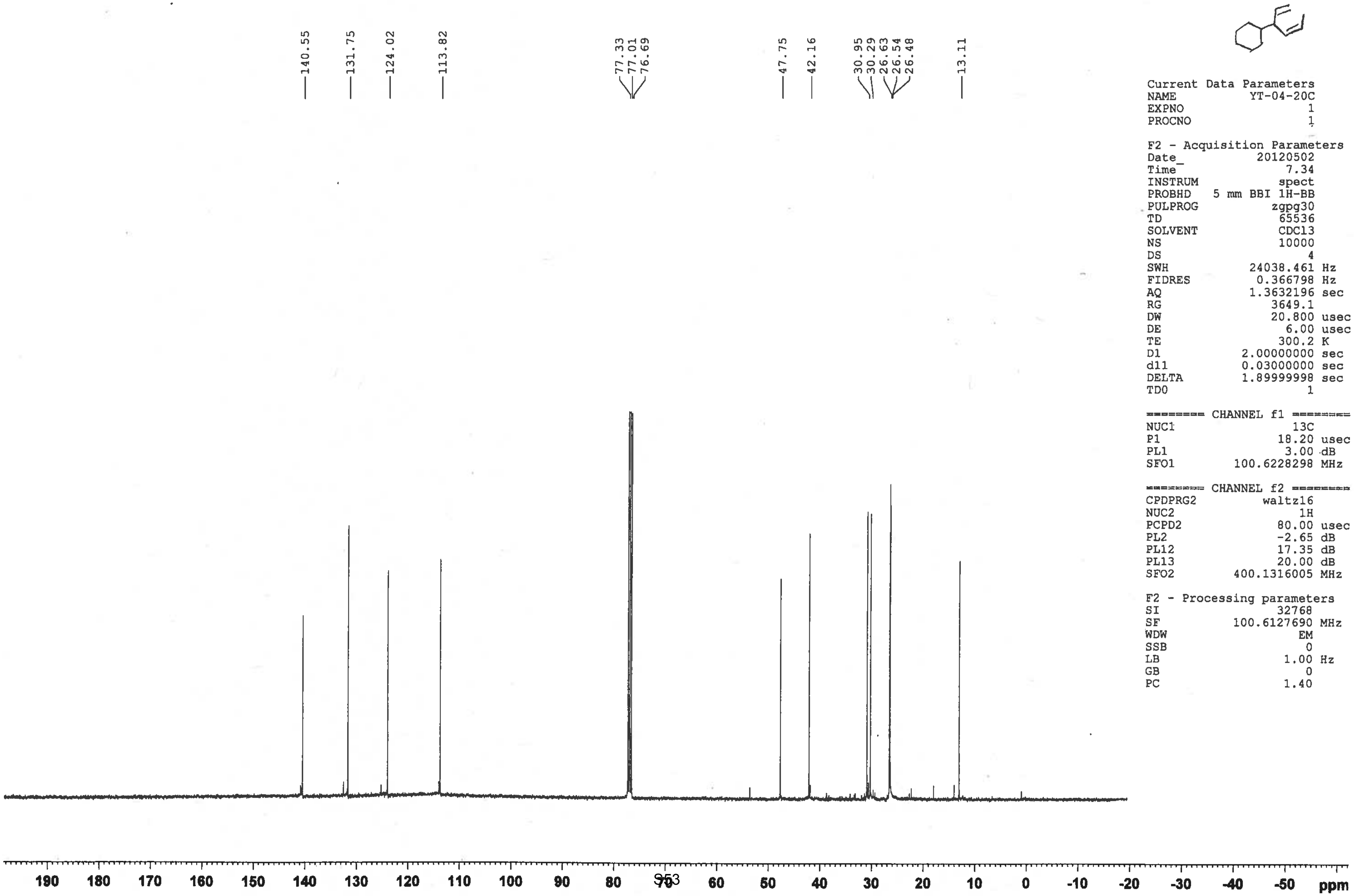

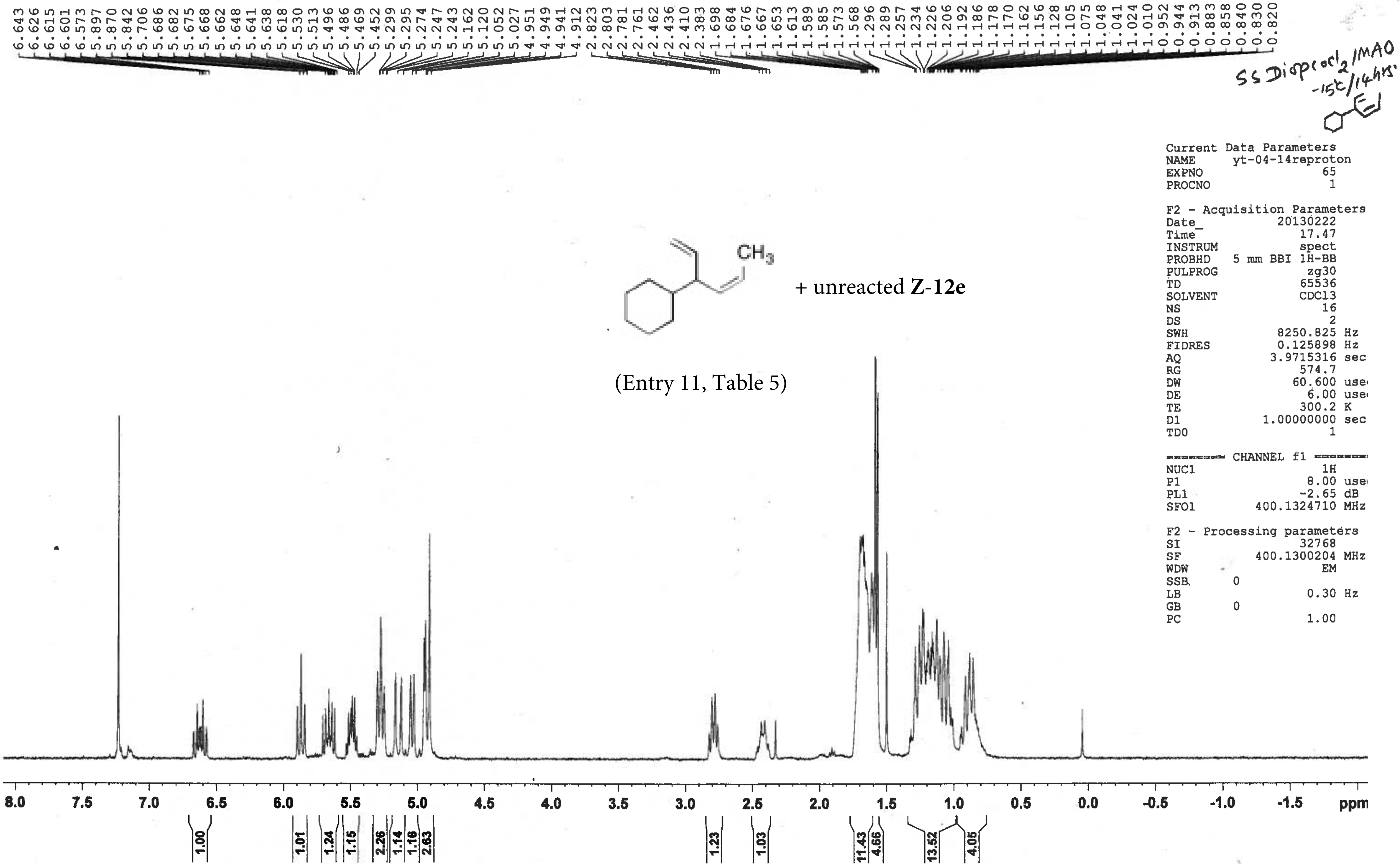

Proton NMR (400 MHz, D-Chloroform) Spectra of Mixture of 119 and Z-118 Using SS-DIOP

Hydrovinylation of (*E*)-1,3-pentadiene (12f)[2]

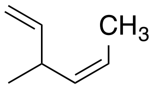

Hydrovinylation of (*E*)-1,3-pentadiene using [DPPB]$CoCl_2$/Me_3Al at – 10 °C (Table 3, Entry 7). [Extracted from ref. 2] To an oven-dried round-bottom flask with a sidearm, was added [dppb]$CoCl_2$ (20.4 mg, 0.037 mmol) under argon and it was dissolved in a mixture of dichloromethane and toluene (1.0 mL) at room temperature. Trimethylaluminum solution (2M) in toluene (0.06 mL 0.11 mmol) was added dropwise as color of the solution changed from deep blue to brown with the formation of white fumes over the solution. When all the fumes disappeared, the reaction vessel was carefully evacuated and then refilled with ethylene from a balloon and a vigorous reaction with evolution of fumes was observed. This evolution stopped in typically within 5 min and the reaction vessel was cooled to – 10 °C and (*E*)-1,3-pentadiene (50.0 mg, 0.74 mmol) was added under ethylene and the mixture was stirred for 6 h. The ethylene balloon was removed and 0.1 mL of water was introduced into the flask and stirring was continued for 5 minutes. The solution was warmed to room temperature and was subsequently passed through a silica plug. The plug was washed with pentane (3 X 10 mL) and pentane was removed under vacuum in acetonitrile bath (-20 °C). Concentration yielded the product as a colorless oil (product + toluene; 501 mg). Isomeric compositions were determined by gas chromatography and NMR spectroscopy (see attached chromatograms and spectra).

^{1}H NMR (500 MHz, $CDCl_3$): 1.072 (d, 7 Hz, 3 H), 1.570 (dd, 6.5, 1.5, Hz, 1 H), 3.167 (m, 1 H), 4.975 (ddd, 10, 1.5, 1.5, 1 H), 5.055 (ddd, 17, 1.5, 1.5, 1 H), 5.301 (ddq, 10, 10, 1.5 Hz, 1 H), 5.451 (ddq, 10, 1, 6, 1 H), 5.783 (ddd, 17.5, 11.0, 6.5, 1 H), [toluene @ 2.183]
In addition, the following minor peaks are seen in the ^{1}H NMR: a doublet at 0.009 ppm up-field from the C_{sp3}-C*H$_3$*; a triplet of multiplet at 0.417 upfield from the bis-allylic C*H* at 3.167. These minor peak might correspond to isomeric byproduct(s).
^{13}C NMR ($CDCl_3$): 12.85, 20.42, 35.20, 112.14, 123.18, 134.30, 142.89.
Isomeric purity by GC (Polydimethylsiloxane): (R_T = 6.289 min.)
CSP GC (Cyclodex-B, 28 °C): R_T = 7.36 min (*R*), 7.47 min (*S*). (*E)*-1,3-pentadiene R_T = 3.20 min., (*Z*)-1,3-pentadiene R_T = 3.47 min.
Asymmetric hydrovinylation of (*E*)-penta-1,3-diene using(*R,R*)[DIOP]$CoCl_2$ at -45 °C (6 h) gave the highest ee for this substrate (>90 %ee, Table 5, Entry 12).[2]

Hydrovinylation of (*E*)-1,3-pentadiene using (*R,R*)-[DIOP]$CoCl_2$ /Me_3Al at – 45 °C (Table 5, Entry 12). To an oven-dried round-bottom flask with a sidearm, was added (*RR*)-[DIOP]$CoCl_2$ (138 mg, 0.220 mmol) under argon and it was dissolved in a mixture of degassed dichloromethane (6 mL) and toluene (1.5 mL), at 0 °C. Trimethylaluminum solution (2M) in toluene (47.6 mg, 330 µl, 0.6604 mmol) was added dropwise as color of the solution changed from deep blue to red-brown with the formation of white fumes over the solution. When all the fumes disappeared, the reaction vessel was carefully evacuated and then refilled with ethylene from a balloon, a vigorous reaction with evolution of fumes was observed. This evolution stopped in typically within 3-5 min. The reaction vessel was cooled to – 45 °C and (*E*)-1,3-pentadiene (300 mg, 440 µl, 4.404 mmol, *E*:*Z* = 99:1) was added under ethylene and the mixture was stirred for 6 h. The ethylene balloon was removed and 0.2 mL of water was introduced into the flask and stirring was continued for 5 minutes. The solution was warmed to room temperature and was subsequently passed through a silica plug. The plug was washed with pentane (3 x 20 mL). Pentane was removed by evacuation from a cold bath (-20 °C). Product

was distilled bulb to bulb at room temperature as a colorless oil (product + toluene; 668 mg). Isomeric compositions were determined by gas chromatography and NMR spectroscopy (see attached chromatograms and spectra).

The product from (dppb)$CoCl_2$ (Entry 7, Table 3) reaction has, the following signals in addition to the ones due to the major product: a CH_3 (?)-doublet (J = 7 Hz) δ 0.008 down-field from the C_{sp3}-CH_3; a CH_3 doublet of multiplet (J = 5 Hz, 3 H) at δ 0.035 down-field from the major C_{sp2}-CH_3 signal; a CH or CH_2 a multiplet δ 0.384 up-field from the bis-allylic C*H* proton. These peaks correspond to isomeric byproduct as determined by GC (see GC traces: isomeric purity of major product determined by GC 94.5%)

Product **13f** from hydrovinylation using [(*R,R*)-(-)-DIOP]$CoCl_2$ at –45 °C: $[\alpha]^{24}_D$ = – 25.6 (toluene, *c* 11.4); lit.[4] $[\alpha]^{20}_D$ (CH_2Cl_2, *c* 10); – 34.8 for **13f** of %ee 37% (*S)*; Because of the volatility of the product, there is some uncertainty in these optical rotation measurements. However, the enantioselectivity measured by CSP GC (Cyclodex-B, 28 °C) is unambiguous (see the attached chromatograms). Enantioselectivity determined by CSP GC: 90.1 % ee (*S)*
Product **13f** from hydrovinylation using [(*S,S*)-(+)-DIOP]$CoCl_2$ at – 45 °C: $[\alpha]^{24}_D$ = + 29.1 (toluene, *c* 0.39). Enantioselectivity determined by CSP GC (Cyclodex-B, 28 °C): 89.1 % ee (*R*).

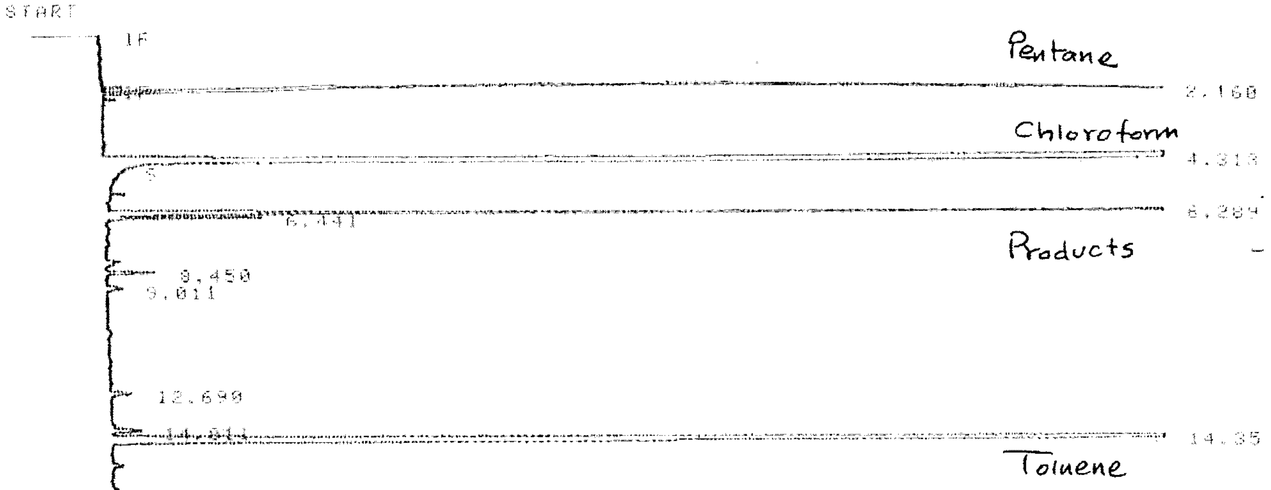

13f from (dppb)CoCl2
(Entry 7, Table 3)

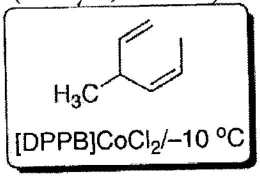

(with pentane and toluene)

13f

Polydimethylsiloxane
(35°C)

TIMETABLE STOP

Closing signal file M:SIGNAL .BNC

RUN# 1198 JAN 7, 1902 15:54:26

SIGNAL FILE: M:SIGNAL.BNC
AREA%

RT	AREA	TYPE	WIDTH	AREA%
2.160	782402	PV	.044	1.11399
4.313	67521920	SPB	.145	96.13789
6.289	620577	BV	.063	.88358
6.441	35326	I VB	.063	.05030
8.450	14459	PV	.079	.02059
14.011	11865	PP	.107	.01689
14.354	1247962	PV	.114	1.77685

TOTAL AREA=7.0234E+07
MUL FACTOR=1.0000E+00

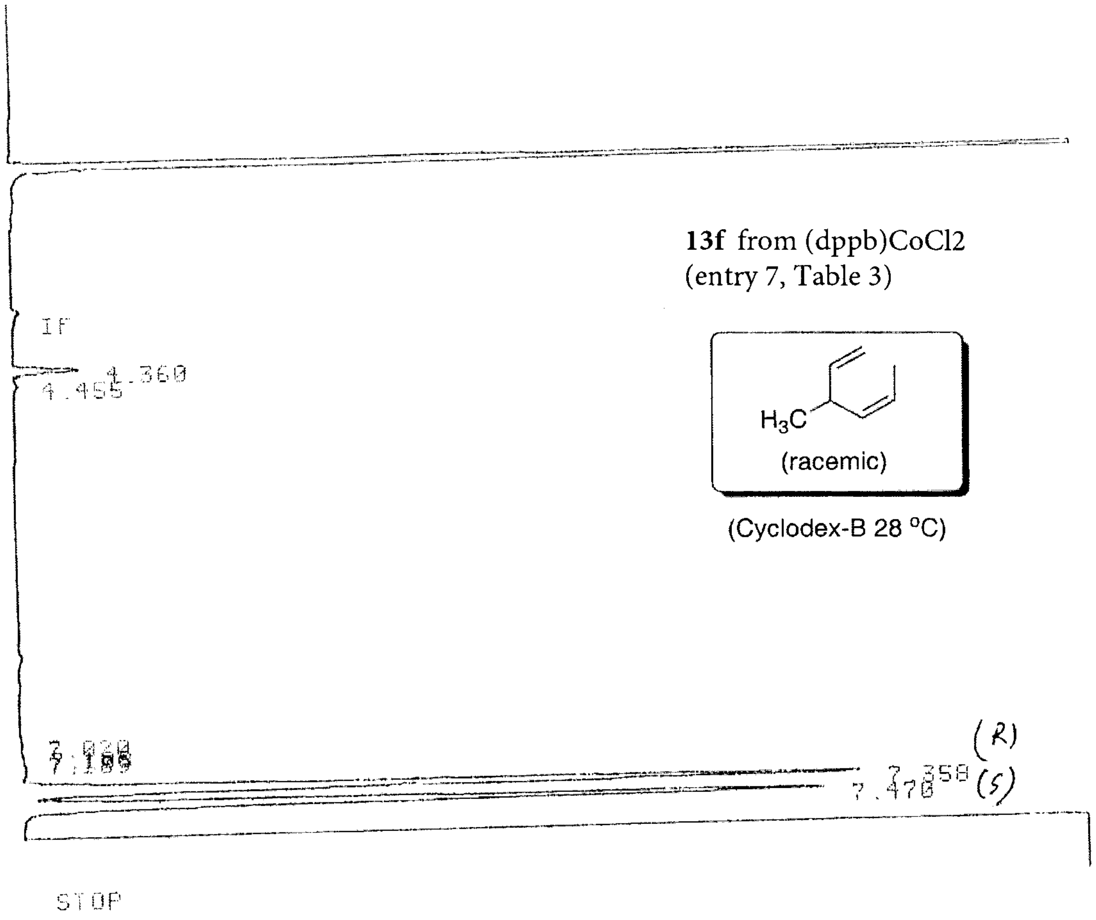

13f from (dppb)CoCl2
(entry 7, Table 3)

(Cyclodex-B 28 °C)

Closing signal file M:SIGNAL .BNA

RUN# 4164 NOV 30, 2009 02:34:51

SIGNAL FILE: M:SIGNAL.BNA
AREA%

RT	AREA	TYPE	WIDTH	AREA%
4.360	1181	PV	.039	3.43724
7.358	17371	VV	.046	50.55734
7.470	15807	VB	.044	46.00542

TOTAL AREA= 34359
MUL FACTOR=1.0000E+00

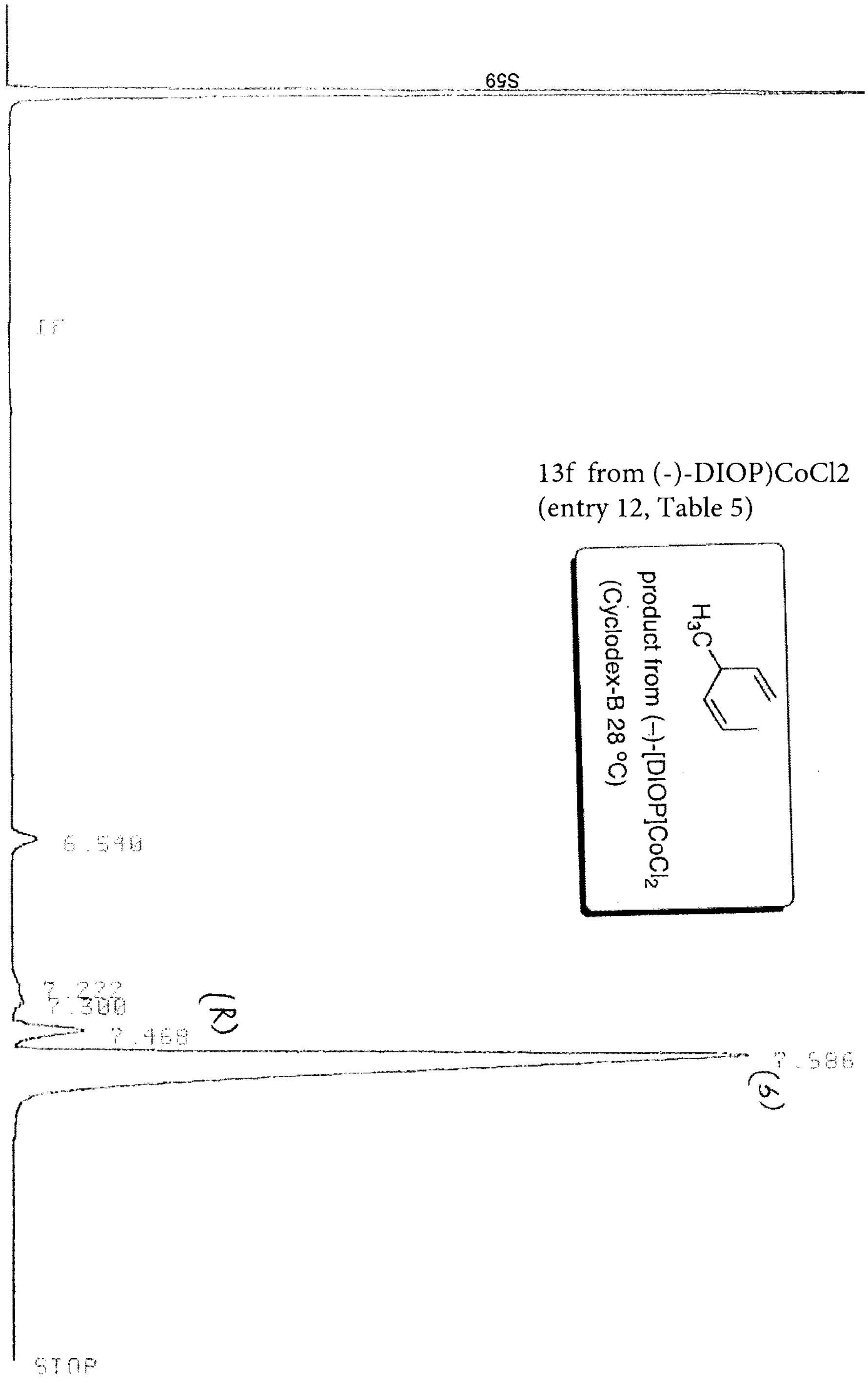

13f from (-)-DIOP)CoCl2
(entry 12, Table 5)

Closing signal file M:SIGNAL .BNA

RUN# 4168 NOV 30, 2009 22:16:01

SIGNAL FILE: M:SIGNAL BNA
AREA%

RT	AREA	TYPE	WIDTH	AREA%
6.540	2895	VP	.059	1.83796
7.222	781	PV	.067	.49584
7.300	1545	VV	.077	.98080
7.468	7483	VV	.056	4.75075
7.586	144808	VB	.106	91.93459

TOTAL AREA= 157512
MUL FACTOR=1.0000E+00

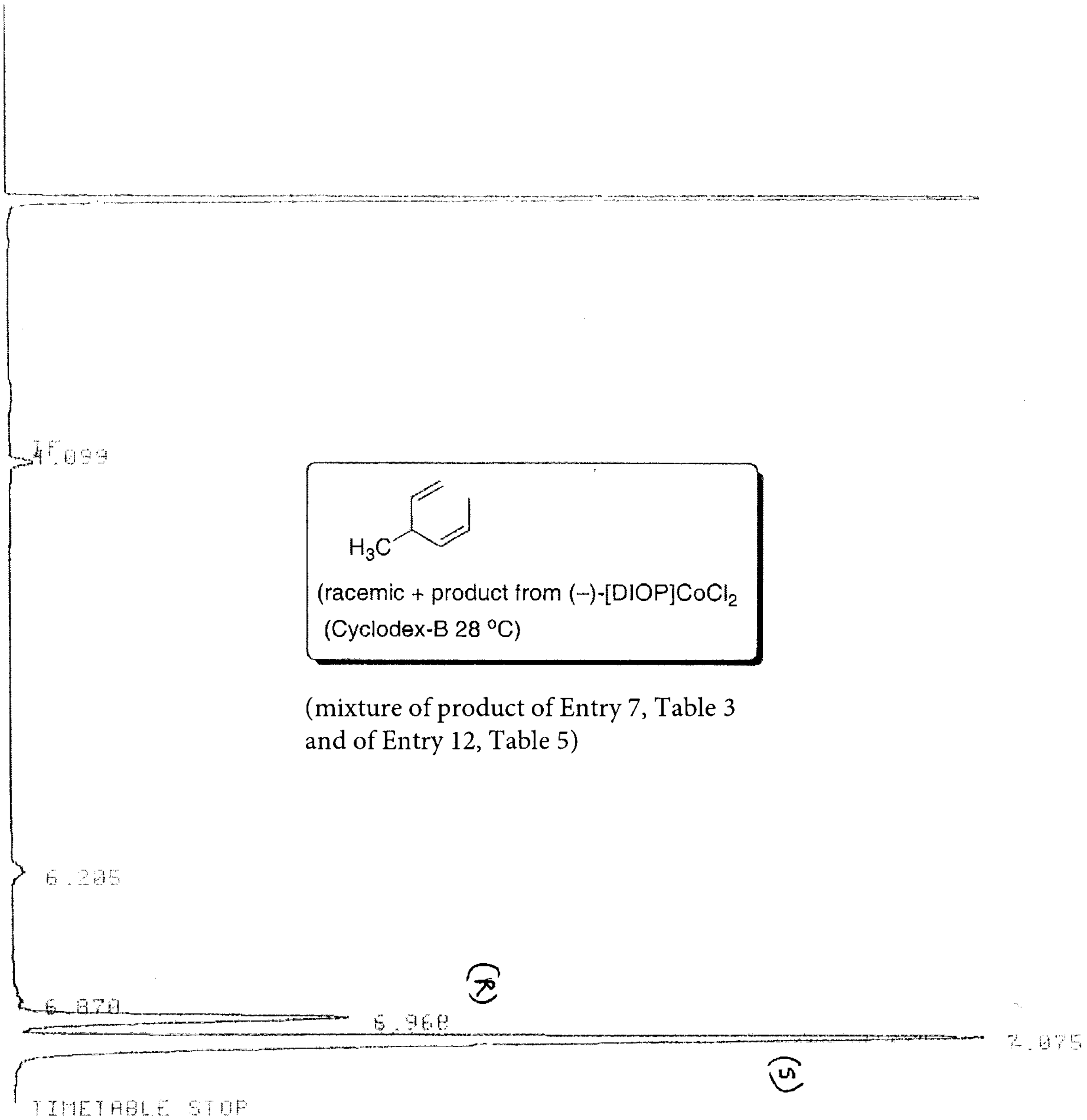

(mixture of product of Entry 7, Table 3 and of Entry 12, Table 5)

Closing signal file M:SIGNAL .BNA

RUN# 4169 DEC 1, 2009 01:16:06

SIGNAL FILE: M:SIGNAL.BNA
AREA%

RT	AREA	TYPE	WIDTH	AREA%
4.099	901	VV	.036	1.28185
6.968	15622	VV	.047	22.22538
7.075	53766	VB	.056	76.49277

TOTAL AREA= 70289
MUL FACTOR=1.0000E+00

(78)

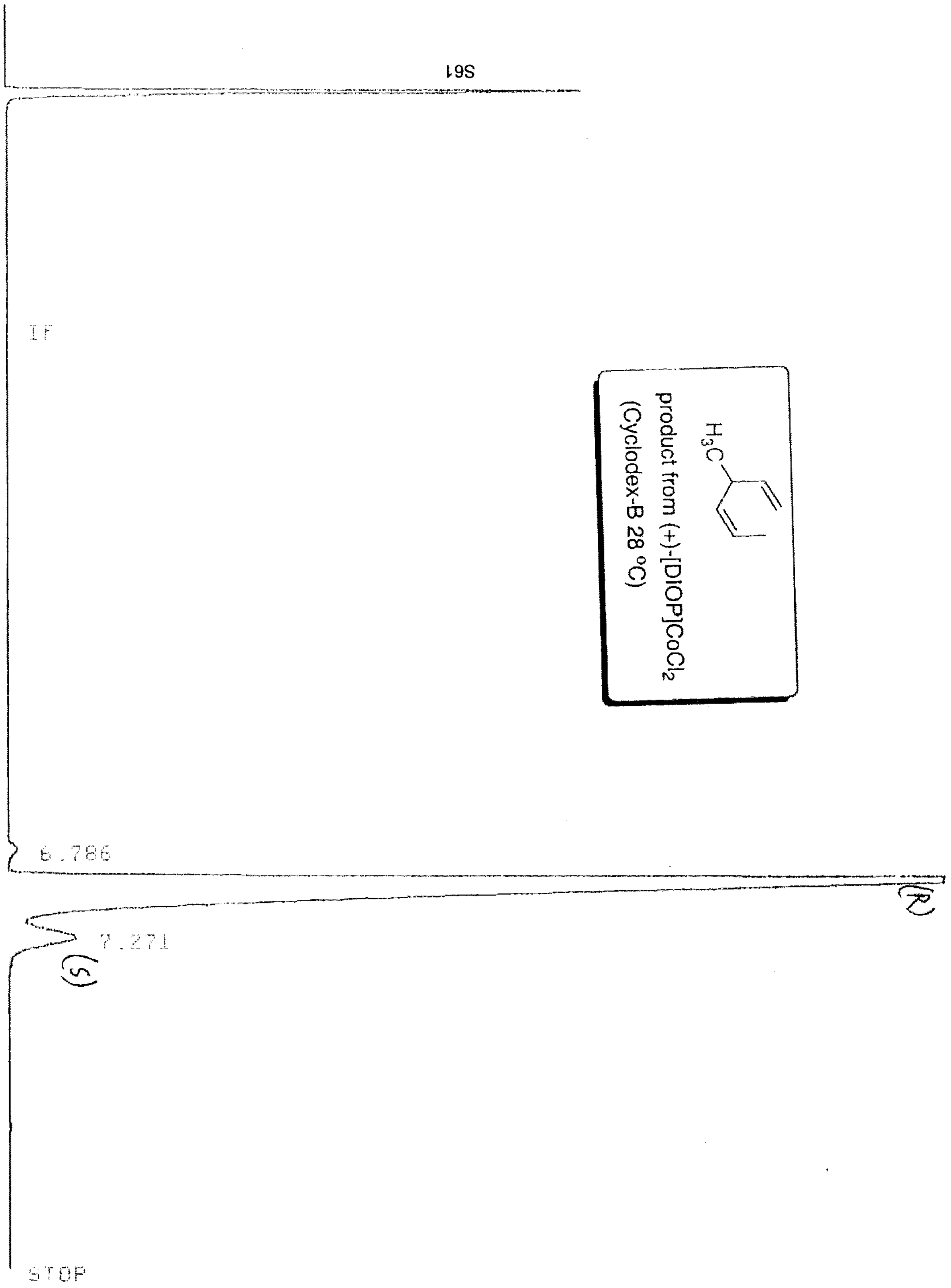

Closing signal file M:SIGNAL .BNA

RUN# 4170   DEC 2, 2009 01:00:00

SIGNAL FILE: M:SIGNAL.BNA
AREA%

RT	AREA	TYPE	WIDTH	AREA%
6.786	1305	PV	.059	.48171
6.943	254946	VV	.115	94.10726
7.271	14659	VB	.106	5.41102

(B2)

TOTAL AREA= 270910
MUL FACTOR=1.0000E+00

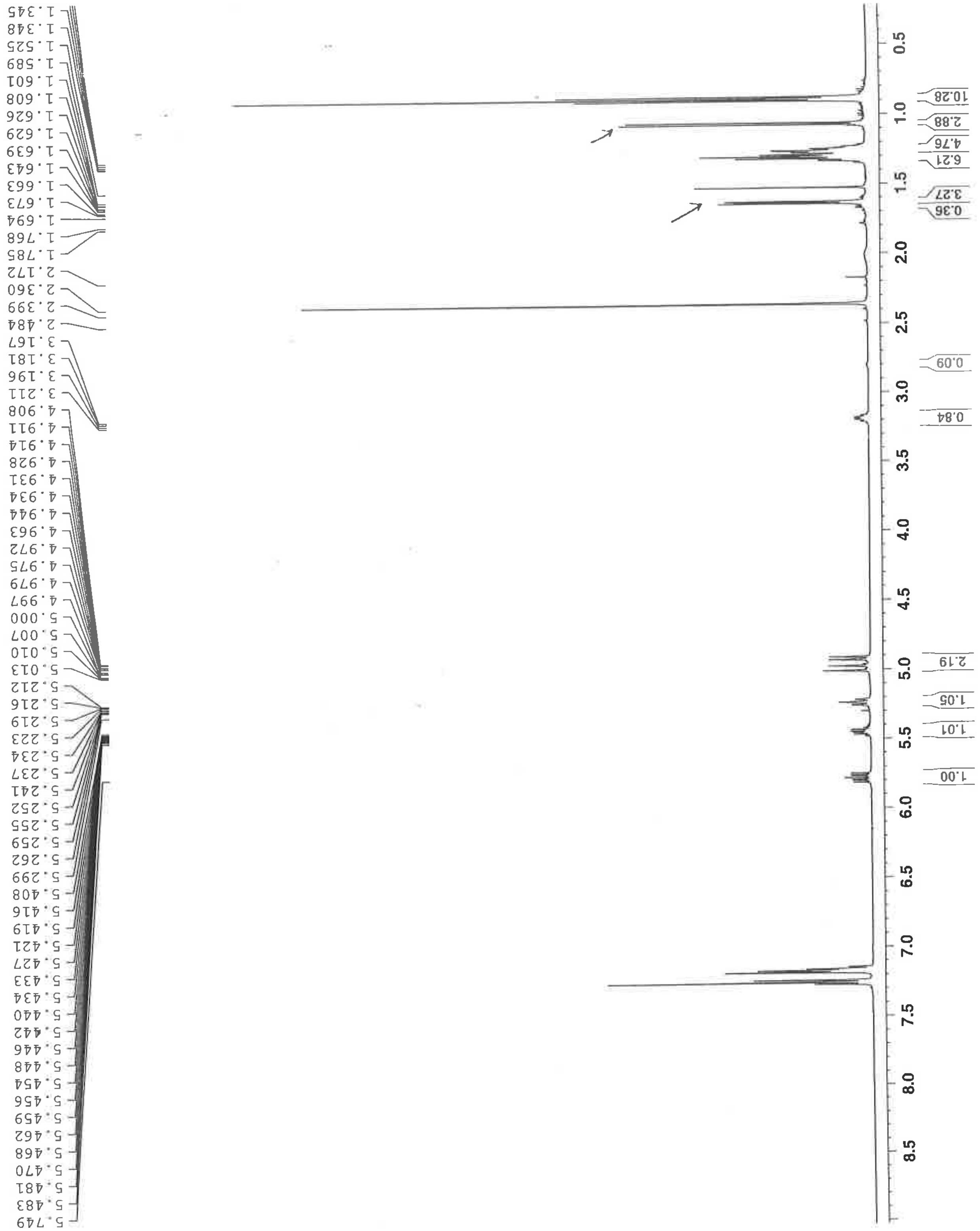
1.345
1.348
1.525
1.589
1.601
1.608
1.626
1.629
1.639
1.643
1.663
1.673
1.694
1.768
1.785
2.172
2.360
2.399
2.484
3.167
3.181
3.196
3.211
4.908
4.911
4.914
4.928
4.931
4.934
4.944
4.963
4.972
4.975
4.979
4.997
5.000
5.007
5.010
5.013
5.212
5.216
5.219
5.223
5.234
5.237
5.241
5.252
5.255
5.259
5.262
5.299
5.408
5.416
5.419
5.421
5.427
5.433
5.434
5.440
5.442
5.446
5.448
5.454
5.456
5.459
5.462
5.468
5.470
5.481
5.483
5.749
10.28
2.88
4.76
6.21
3.27
0.36
0.09
0.84
2.19
1.05
1.01
1.00
0.5
1.0
1.5
2.0
2.5
3.0
3.5
4.0
4.5
5.0
5.5
6.0
6.5
7.0
7.5
8.0
8.5

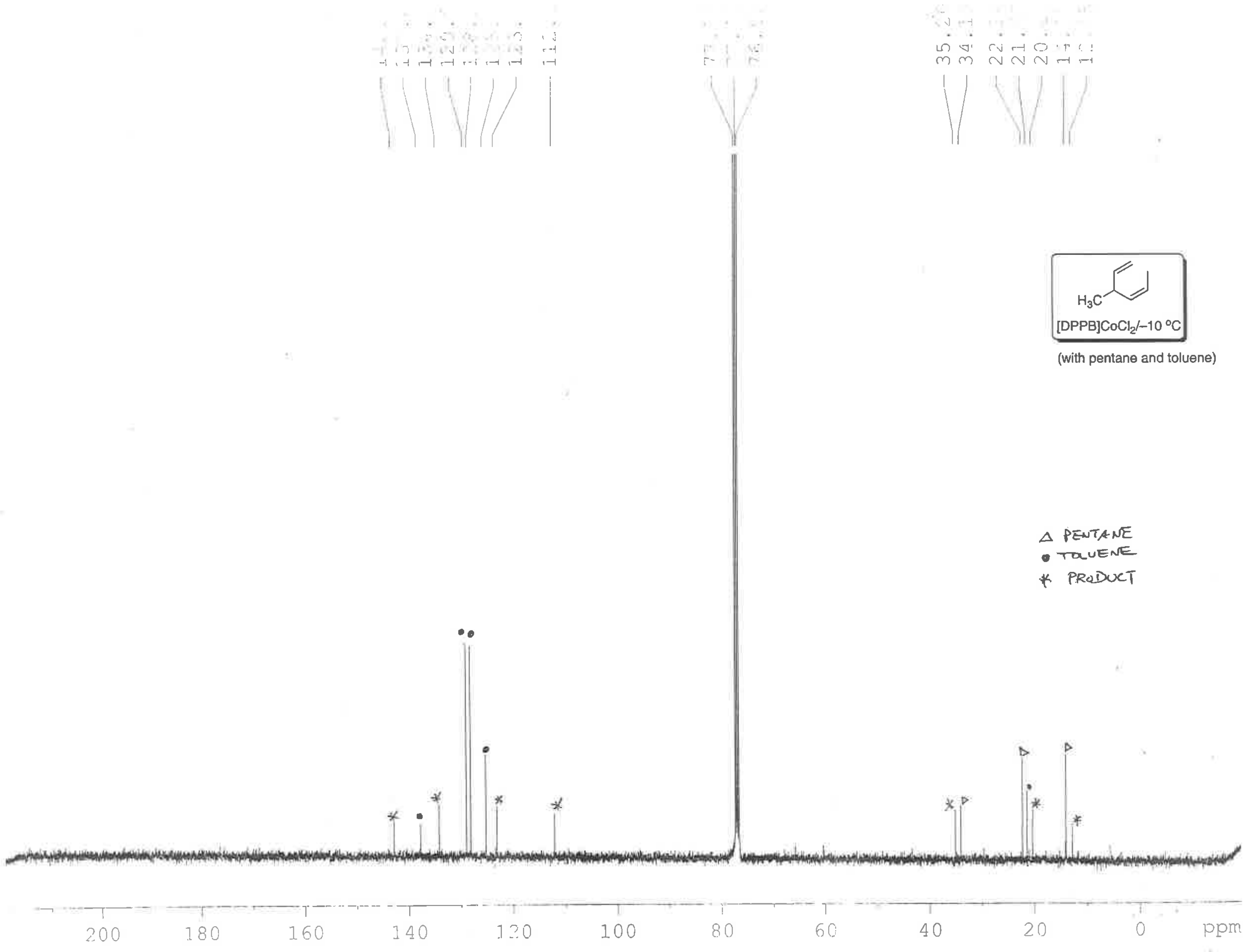

H_3C
[DPPB]CoCl$_2$/–10 °C
(with pentane and toluene)
△ PENTANE
● TOLUENE
✱ PRODUCT
200 180 160 140 120 100 80 60 40 20 0 ppm

Hydrovinylation of (*E*)-1-phenyl-1,3-butadiene

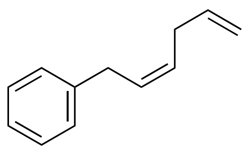

Hydrovinylation of (*E*)-1-phenyl-1,3-butadiene using[dppp]$CoCl_2$ (Table 3, Entry 8): To an oven dried round-bottom-flask with a sidearm, was added DPPP$CoCl_2$ (45 mg, 0.083 mmol) under argon and was dissolved in dichloromethane (1 mL) at room temperature. Trimethylaluminium as a 2M solution in toluene (0.13 mL, 0.25 mmol) was added dropwise and the color of the solution changed from deep blue to red brown with the formation of white fumes over the solution. After 2 to 3 minutes, the reaction vessel was carefully evacuated and then refilled with ethylene from a balloon. The reaction vessel was cooled to -20 ^oC and (*E*)-1-phenyl-1,3-butadiene (108 mg, 0.83 mmol) as a dichloromethane solution (0.5 mL) was added under ethylene and the mixture was stirred for 4 h. The ethylene balloon was removed and 0.1 mL methanol was added to the flask. The solution was diluted with pentane (2 mL) and warmed to room temperature and subsequently passed through a silica plug. The plug was washed with pentane (3 x 5 mL). Removal of solvent yielded the product as a colorless oil (113 mg, 86%). GC and NMR analysis showed that the product was essentially pure linear adduct (**16g**). ^1H NMR ($CDCl_3$, 500 MHz): 2.95-2.98 (m, 2 H), 3.45 (d, J = 7.5 Hz, 2 H), 5.06 (dq, J = 10, 1.5 Hz, 1 H), 5.13 (dq, J= 17, 1.5 Hz, 1 H), 5.56 - 5.62 (m, 1 H), 5.67 - 5.72 (m, 1 H), 5.89 (ddd, J = 6, 10, 17 Hz, 1 H), 7.21 - 7.23 (m, 3 H, aromatic), 7.31 - 7.34 (m, 2 H, aromatic).^{13}C NMR ($CDCl_3$, 125.02 MHz): 31.74, 33.66, 115.14, 126.12, 127.86, 128.58, 128.64, 129.53, 136.91, 141.07. Gas Chromatography: CSP GC (Cyclosil, OT 100 ^oC/ isothermal, H_2 carrier gas). Starting material R_t = 17.68 min. R_T for achiral linear product = 32.58 min.

This following table shows the additional experiments conducted with this diene, 1-phenyl-1,3-butadiene, employing 10 mol % catalyst (see Table 3 in the paper for details).

Entry	Catalyst	Tem(^{o}C) /Time(h)	Activator	Conv.(%)	Product
1.	DPPM$CoCl_2$	-20/7	TMA	100	1,2-HV
2.	DPPE$CoCl_2$	-10/3.5	TMA	89	1,4-linear
3.	DPPP$CoCl_2$	-20/4	TMA	97	1,4-linear
4.	DPPB$CoCl_2$	-10/6	TMA	87	1,4-linear
5.	(*S,S*)-[DIOP]$CoCl_2$	0-rt/5	MAO	97	1,4-linear (54%)+ 1,2-HV (43%, 52% ee)
6.	[DIOP]$CoCl_2$	-40/8	TMA	45	1,4-linear (40%) +1,2-HV(4%)
7.	(*SS*)-[BDPP]$CoCl_2$	-20/11	TMA	95(70+16)	1,2-HV+1,4 linear (ee not determined)
8.	DIPAMP	-12/11	TMA	6	1,4-linear
9.	DPPP$CoCl_2$	-40/8	TMA	8	1,4-linear

Data File C:\CHEM32\1\DATA\YAM\YT-1-CHIRAL 2012-10-17 11-51-48\YT-03-227,229,235TWO.D
Sample Name: YT-03-227,229,235two.D

===

Acq. Operator : yam   Seq. Line : 1
Acq. Instrument : Babu   Location : Vial 201
Injection Date : 10/17/2012 11:52:47 AM   Inj : 1
                                                          Inj Volume : 1 µl
Different Inj Volume from Sequence ! Actual Inj Volume : 5 µl
Sequence File : C:\Chem32\1\DATA\YAM\YT-1-CHIRAL 2012-10-17 11-51-48\YT-1-CHIRAL.S
Method : C:\CHEM32\1\DATA\YAM\YT-1-CHIRAL 2012-10-17 11-51-48\YT_85_C_CHIRAL.M (Sequence Method)
Last changed : 10/17/2012 11:00:05 AM by yam

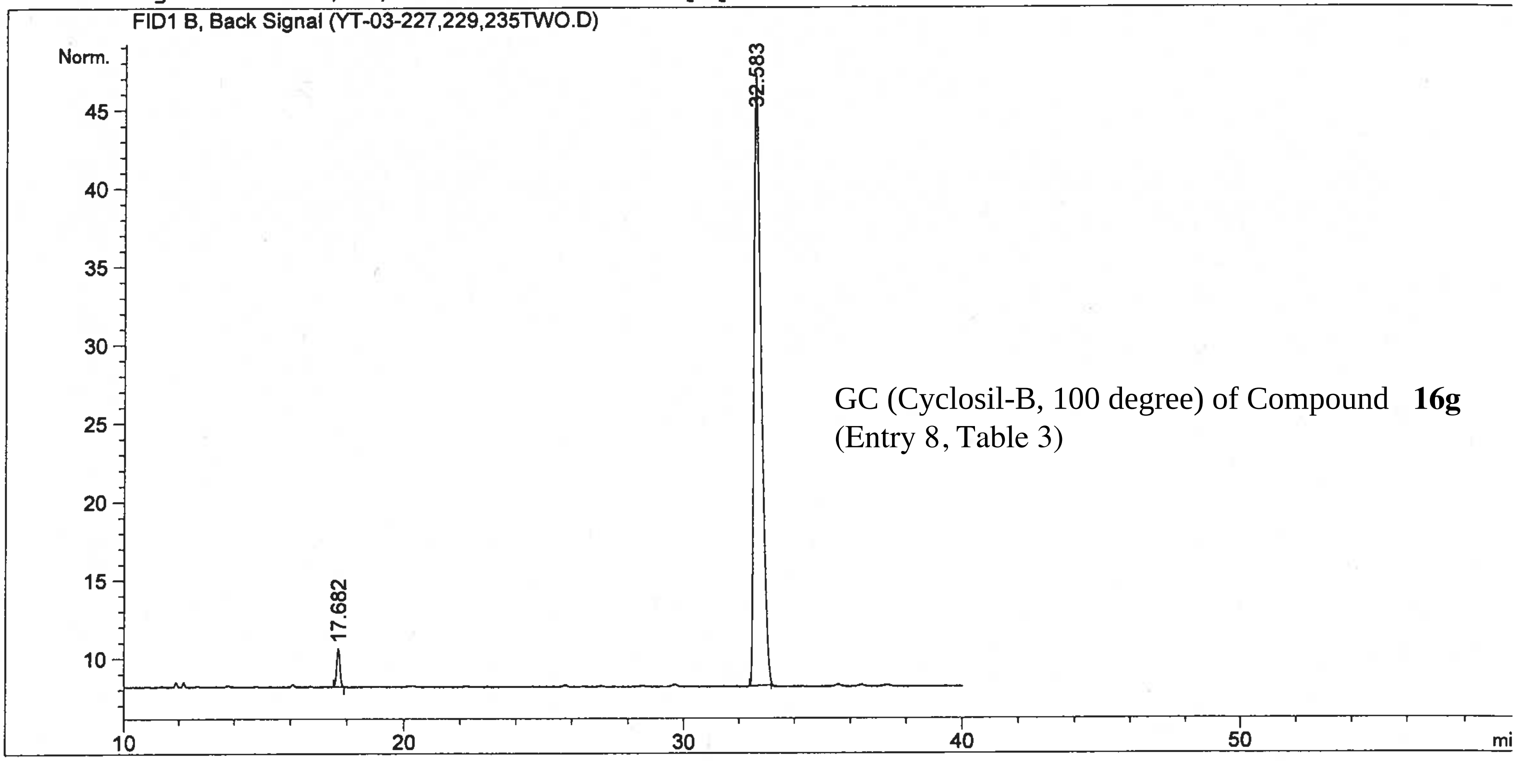

GC (Cyclosil-B, 100 degree) of Compound **16g** (Entry 8, Table 3)

===

Area Percent Report

===

Sorted By : Signal
Multiplier: : 1.0000
Dilution: : 1.0000
Use Multiplier & Dilution Factor with ISTDs

Signal 1: FID1 B, Back Signal

Peak #	RetTime [min]	Type	Width [min]	Area [pA*s]	Height [pA]	Area %
1	6.254	BB	0.0479	1.72123	5.71427e-1	0.23561
2	17.682	BB	0.1231	19.35352	2.43581	2.64916
3	32.583	BB	0.2413	709.47870	38.95836	97.11524

Totals : 730.55345 41.96559

===

*** End of Report ***

Proton NMR (500 MHz, D-Chloroform) spectrum of compound **16g** (HV using Cl2Co(DPPP)] (Entry 8, Table 3)

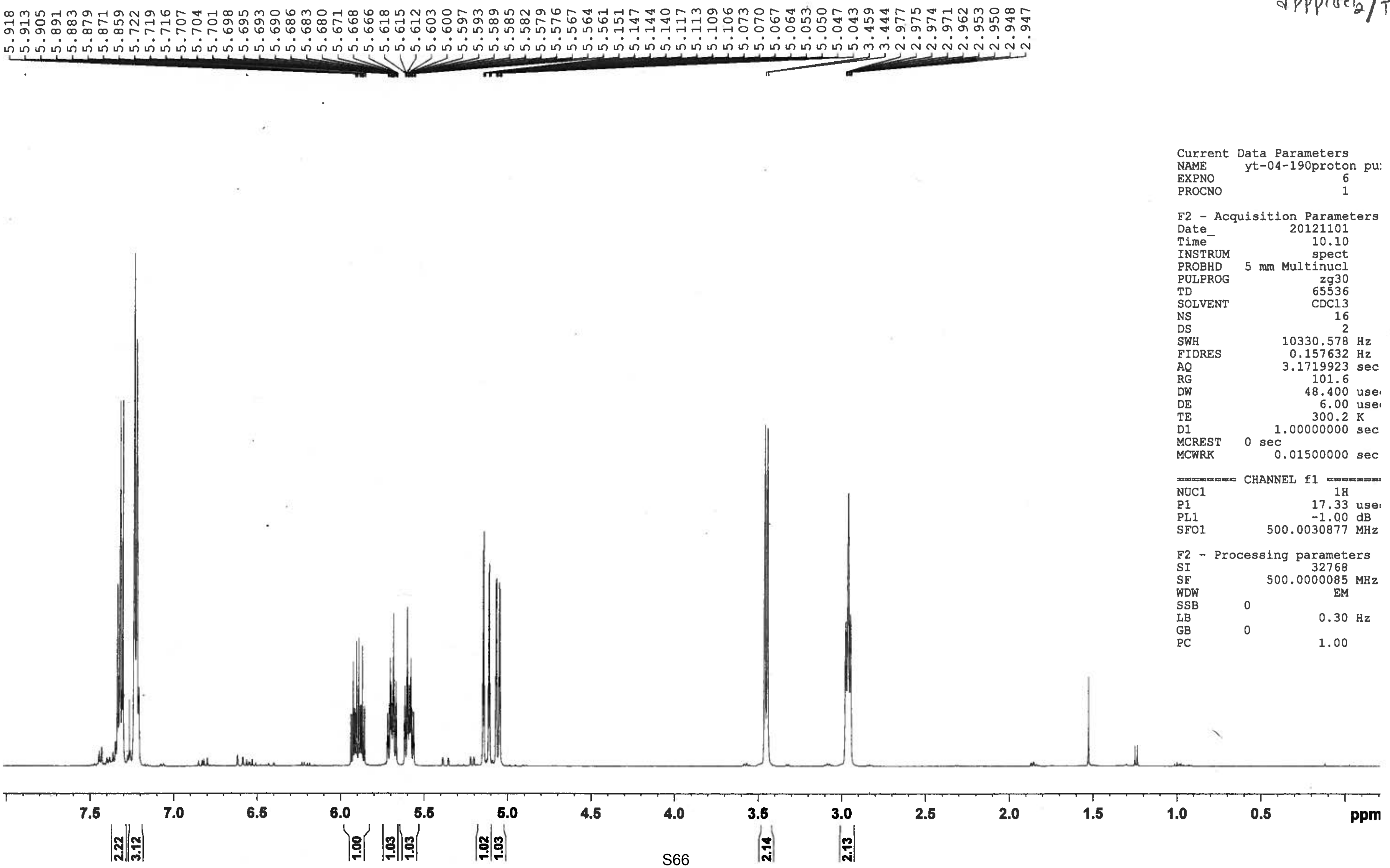

Proton NMR (500 MHz, D-Chloroform) spectrum of compound **16g** (HV using Cl2Co(DPPP))](Alkene Expansion)

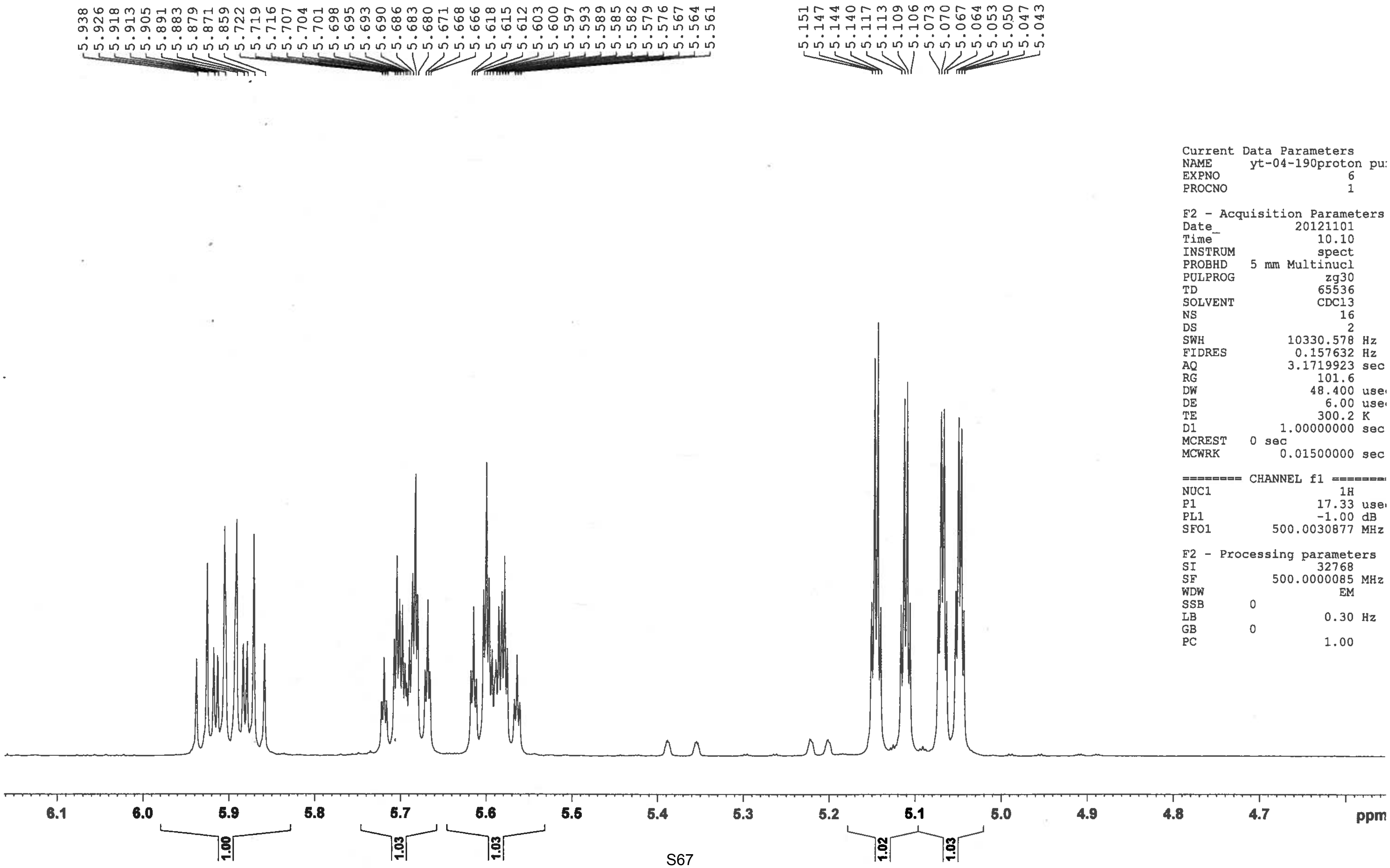

Current Data Parameters	
NAME	yt-04-190proton pu:
EXPNO	6
PROCNO	1
F2 - Acquisition Parameters	
Date_	20121101
Time	10.10
INSTRUM	spect
PROBHD	5 mm Multinucl
PULPROG	zg30
TD	65536
SOLVENT	CDCl3
NS	16
DS	2
SWH	10330.578 Hz
FIDRES	0.157632 Hz
AQ	3.1719923 sec
RG	101.6
DW	48.400 use
DE	6.00 use
TE	300.2 K
D1	1.00000000 sec
MCREST	0 sec
MCWRK	0.01500000 sec
======== CHANNEL f1 ========	
NUC1	1H
P1	17.33 use
PL1	-1.00 dB
SFO1	500.0030877 MHz
F2 - Processing parameters	
SI	32768
SF	500.0000085 MHz
WDW	EM
SSB	0
LB	0.30 Hz
GB	0
PC	1.00

Carbon NMR (500 MHz, D-Chloroform) spectrum of compound **16g** (HV using Cl2Co(DPPP)]

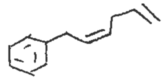

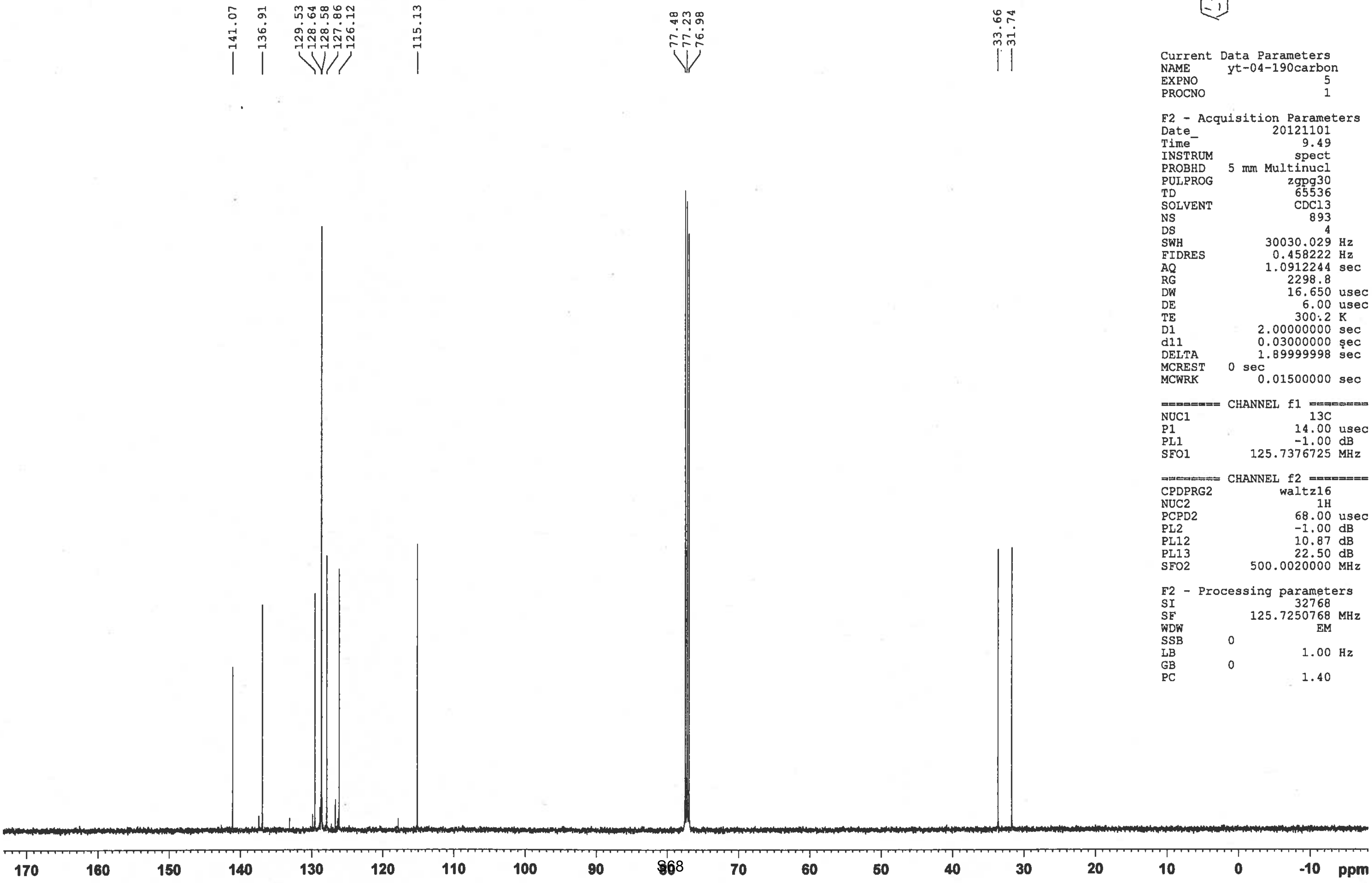


```
Current Data Parameters
NAME      yt-04-190carbon
EXPNO                   5
PROCNO                  1

F2 - Acquisition Parameters
Date_            20121101
Time                 9.49
INSTRUM             spect
PROBHD   5 mm Multinucl
PULPROG           zgpg30
TD                 65536
SOLVENT            CDCl3
NS                   893
DS                     4
SWH           30030.029 Hz
FIDRES         0.458222 Hz
AQ            1.0912244 sec
RG                2298.8
DW                16.650 usec
DE                  6.00 usec
TE                 300.2 K
D1            2.00000000 sec
d11           0.03000000 sec
DELTA         1.89999998 sec
MCREST   0 sec
MCWRK         0.01500000 sec

======== CHANNEL f1 ========
NUC1                 13C
P1                 14.00 usec
PL1                -1.00 dB
SFO1         125.7376725 MHz

======== CHANNEL f2 ========
CPDPRG2          waltz16
NUC2                  1H
PCPD2              68.00 usec
PL2                -1.00 dB
PL12               10.87 dB
PL13               22.50 dB
SFO2         500.0020000 MHz

F2 - Processing parameters
SI                 32768
SF           125.7250768 MHz
WDW                   EM
SSB      0
LB                  1.00 Hz
GB       0
PC                  1.40
```

Hydrovinylation of (*E*)-1-phenyl-1,3-butadiene using [dppm]$CoCl_2$ (Table 3, Entry 10): To an oven dried round-bottom-flask with a sidearm, was added DPPM$CoCl_2$ (34 mg, 0.066 mmol) and under argon it was dissolved in dichloromethane (1 mL) at room temperature. Trimethylaluminium as a 2M solution in toluene (0.1 ml, 0.20 mmol) was added dropwise and the color of the solution changed from bluish brown to red brown with the formation of white fumes over the solution. After 2 to 3 minutes, the reaction vessel was carefully evacuated and then refilled with ethylene from a balloon. The reaction vessel was cooled to -20 ^{o}C and (*E*)-1-phenyl-1,3-butadiene (86 mg, 0.66 mmol) in dichloromethane (1 mL) added under ethylene and the mixture was stirred for 7 h. The ethylene balloon was removed and 0.1 mL methanol was added to the flask. The solution was diluted with pentane (2 mL) and warmed to room temperature and subsequently passed through a silica plug. The plug was washed with pentane (3 X 5 mL). Removal of solvent yielded the product as colorless oil (83 mg, 80%). GC and NMR analysis showed that the product was essentially pure 1,2 hydrovinylation product (**15g**). ^{1}H NMR ($CDCl_3$, 500 MHz): 1.19 (d, J = 7 Hz, 3 H), 2.99 – 3.05 (m, 1 H), 4.99 – 5.08 (m, 2 H), 5.87 (ddd, J = 6.5, 10, 17 Hz, 1 H), 6.17 (dd, J = 7, 16 Hz, 1 H), 6.37 (d, J = 16 Hz, 1 H), 7.18 – 7. 36 (m, aromatic, 5 H). ^{13}C NMR ($CDCl_3$, 125.02 MHz): 20.00, 40.85, 113.54, 126.28, 127.20, 128.69, 128.86, 134.47, 137.86, 142.64. Gas Chromatography: CSP GC (Cyclosil, OT 120 ^{o}C/ isothermal, H_2 carrier gas) R_T for chiral 1,2 product = 14.34 and 14.58 min (enantiomers, racemic), (*SS*)-DIOP gave 52% ee with 43% yield. Methylsilicone SP GC (OT 120 ^{o}C/ isothermal, He carrier gas) R_t for chiral 1,2 product = 10.56 min.

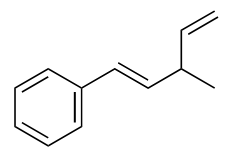

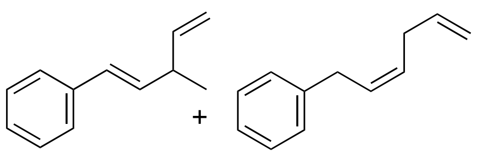

Hydrovinylation of (*E*)-1-phenyl-1,3-butadiene using (*S,S*)[DIOP]$CoCl_2$ (Table 5, Entry 13): To an oven dried round bottom flask with a sidearm, was added (*S,S*)[DIOP]$CoCl_2$ (24 mg, 0.038 mmol) and methylaluminoxane (44 mg, 0.76 mmol) under argon. The color of the solution changed from deep blue to red brown with the formation of white fumes over the addition of dichloromethane solvent (1.5 mL) at room temperature. After 2 to 3 minutes when fumes stopped, the reaction vessel was carefully evacuated and then refilled with ethylene balloon. The reaction vessel was cooled to 0 ^{o}C and (*E*)-1-phenyl-1,3-butadiene (49 mg, 0.38 mmol) in dichloromethane (1 mL) was added via syringe under ethylene and the mixture was stirred for 5 h in ambient temperature until the completion of reaction monitored via gas chromatography. The ethylene balloon was removed and 0.1 mL methanol was added into the flask. The reaction solution was diluted with pentane (5 mL) and warmed to room temperature and subsequently passed through a silica plug. The plug was washed with pentane (3 X 10 mL). Removal of the solvent yielded the product as colorless oil as a mixture of two major isomers **15g** (43% with 54% ee) and **16g** (55%) (Table 5, Entry 13). GC and NMR analysis showed that the products were essentially pure and the spectra and chromatogram are attached.

dPPMCoCl2/TMA
YT-04-220RR
OT 120°C/ 180/achiral

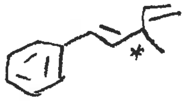

*
*
*
*
*
*
*
*
*
*
*
*
*
* RUN # 236 MAR 20, 1901 01:38:14
START

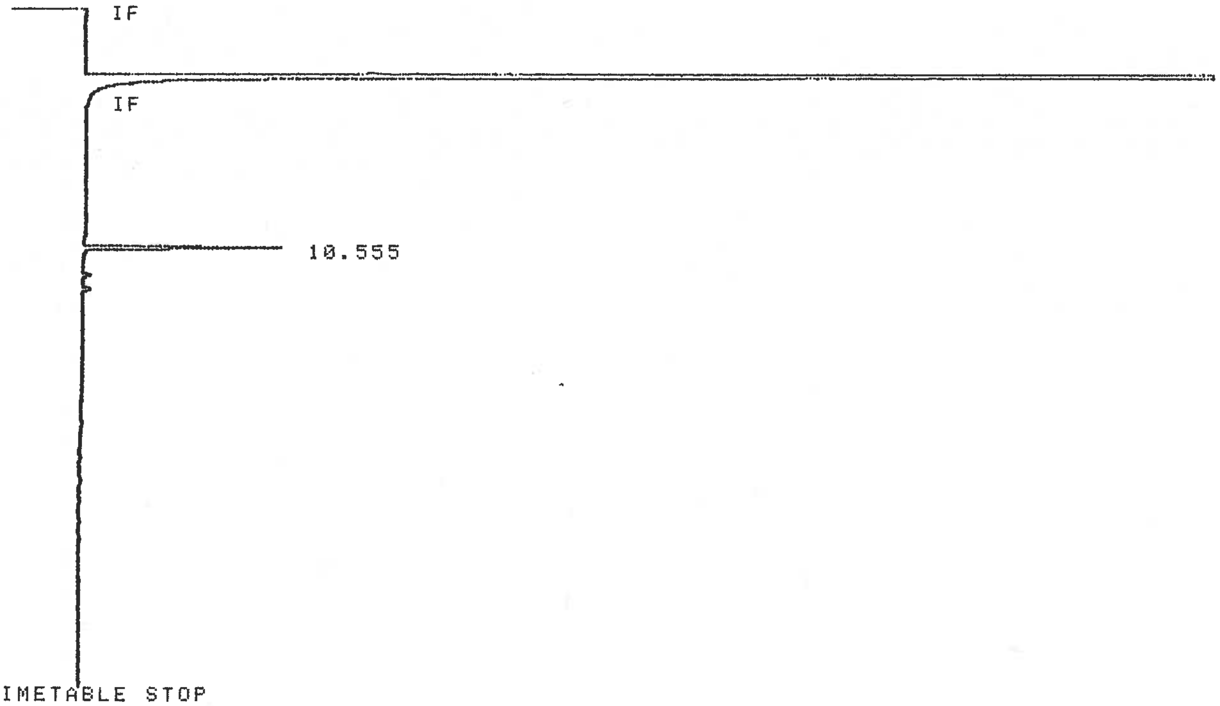

TIMETABLE STOP

RUN# 236 MAR 20, 1901 01:38:14

AREA%

RT	AREA	TYPE	WIDTH	AREA%
10.555	65242	BB	.096	100.00000

TOTAL AREA= 65242
MUL FACTOR=1.0000E+00

GC (HP-5 Methyl silicone, 120 degree) of racemic Compound **15g** (HV Using Cl2Co(DPPM) (Entry 10, Table 3)

*
*
*
*
*
*

Data File C:\CHEM32\1\DATA\YAM\YT-1-CHIRAL 2013-01-30 09-48-53\YT-04-220RR.D
Sample Name: YT-04-220RR.D

```
=====================================================================
Acq. Operator   : YAM                                Seq. Line :   1
Acq. Instrument : Babu                                Location : Vial 201
Injection Date  : 1/30/2013 9:50:07 AM                     Inj :   1
                                                    Inj Volume : 1 µl
Different Inj Volume from Sequence !         Actual Inj Volume : 3 µl
Acq. Method     : C:\CHEM32\1\DATA\YAM\YT-1-CHIRAL 2013-01-30 09-48-53\YT_85_C_CHIRAL.M
Last changed    : 1/30/2013 9:48:53 AM by YAM
Analysis Method : C:\CHEM32\1\METHODS\YT_85_C_CHIRAL.M
Last changed    : 1/30/2013 10:37:42 AM by YAM
                  (modified after loading)
```

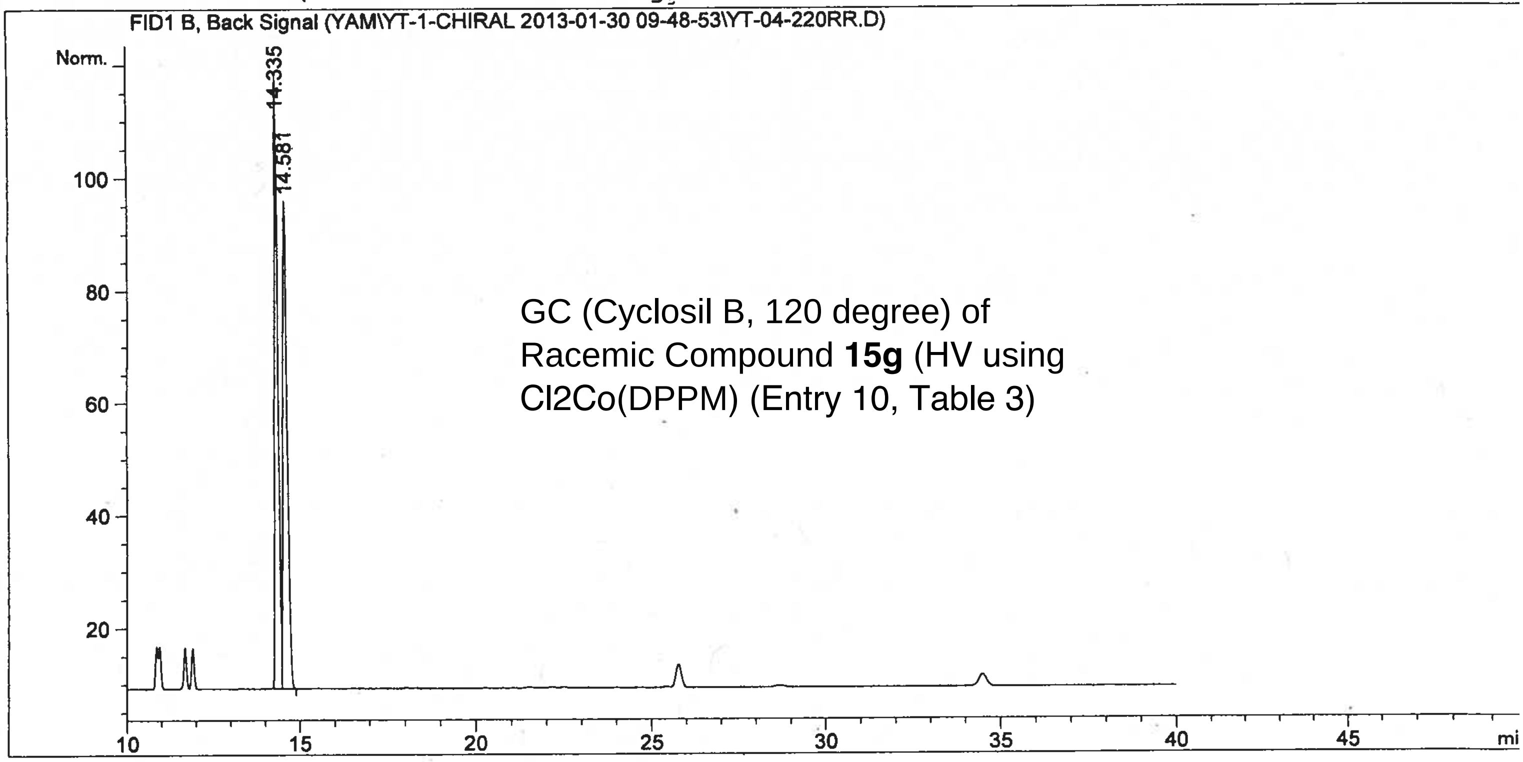


```
=====================================================================
                         Area Percent Report
=====================================================================

Sorted By             :      Signal
Multiplier:                   :      1.0000
Dilution:                     :      1.0000
Use Multiplier & Dilution Factor with ISTDs


Signal 1: FID1 B, Back Signal
```

Peak #	RetTime [min]	Type	Width [min]	Area [pA*s]	Height [pA]	Area %
1	14.335	BV	0.1027	747.93854	108.62716	49.76770
2	14.581	VB	0.1288	754.92090	86.67773	50.23230

Totals :				1502.85944	195.30489	

```
=====================================================================
                         *** End of Report ***
```

Data File C:\CHEM32\1\DATA\YAM\YT-1-CHIRAL 2012-10-12 16-55-52\YT-04-171RR.D
Sample Name: YT-04-171RR.D

===

Acq. Operator : YAM Seq. Line : 1
Acq. Instrument : Babu Location : Vial 201
Injection Date : 10/12/2012 4:56:56 PM Inj : 1
Inj Volume : 1 µl
Different Inj Volume from Sequence ! Actual Inj Volume : 5 µl
Sequence File : C:\Chem32\1\DATA\YAM\YT-1-CHIRAL 2012-10-12 16-55-52\YT-1-CHIRAL.S
Method : C:\CHEM32\1\DATA\YAM\YT-1-CHIRAL 2012-10-12 16-55-52\YT_85_C_CHIRAL.M (Sequence Method)
Last changed : 10/12/2012 3:42:07 PM by YAM

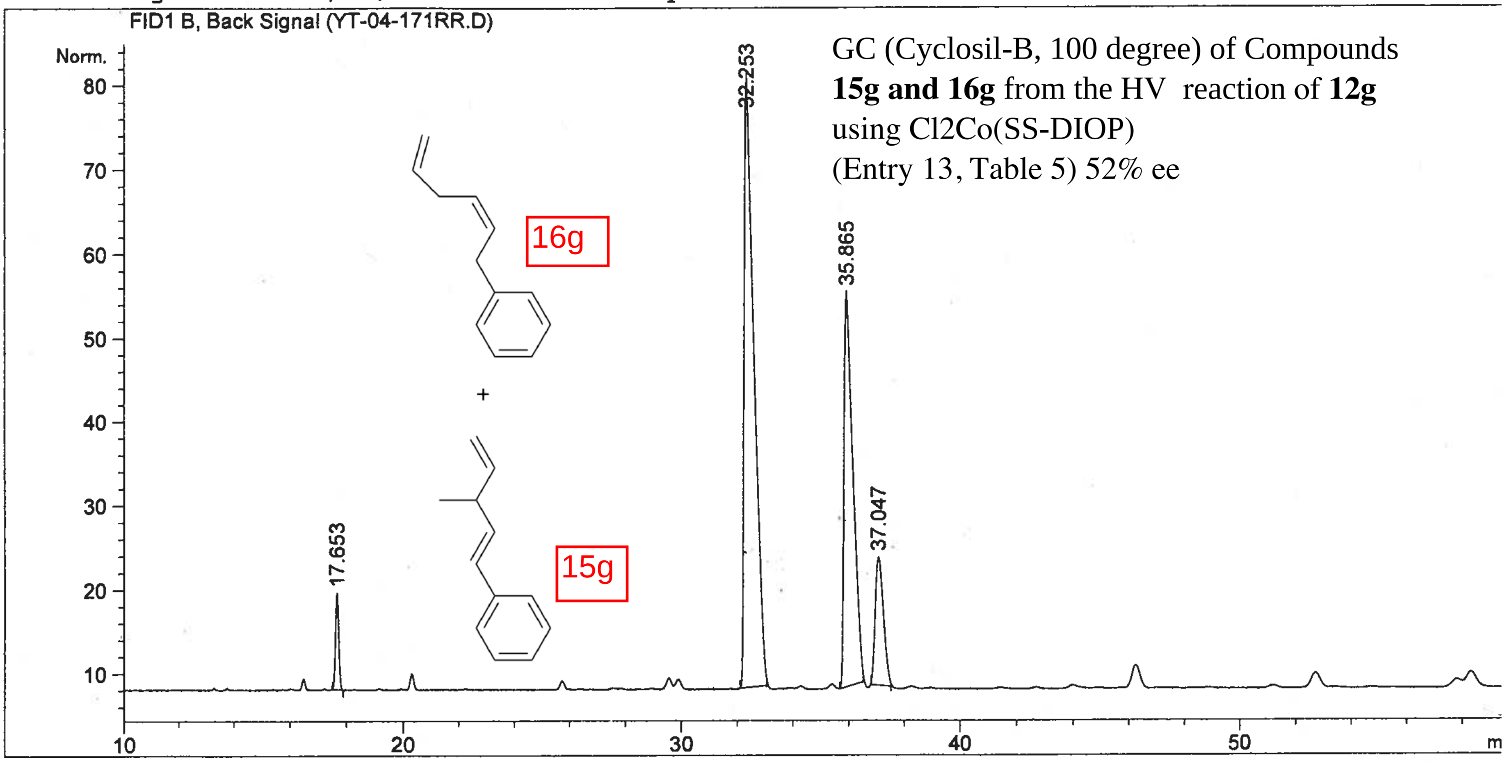

===

Area Percent Report

===

Sorted By : Signal
Multiplier: : 1.0000
Dilution: : 1.0000
Use Multiplier & Dilution Factor with ISTDs

Signal 1: FID1 B, Back Signal

Peak #	RetTime [min]	Type	Width [min]	Area [pA*s]	Height [pA]	Area %
1	17.653	BB	0.1264	91.76698	11.54052	2.85662
2	32.253	BB	0.3078	1760.57617	72.79673	54.80507
3	35.865	BB	0.2973	1046.50647	47.14975	32.57676
4	37.047	BB	0.2925	313.58331	15.26240	9.76155

Totals : 3212.43293 146.74941

===

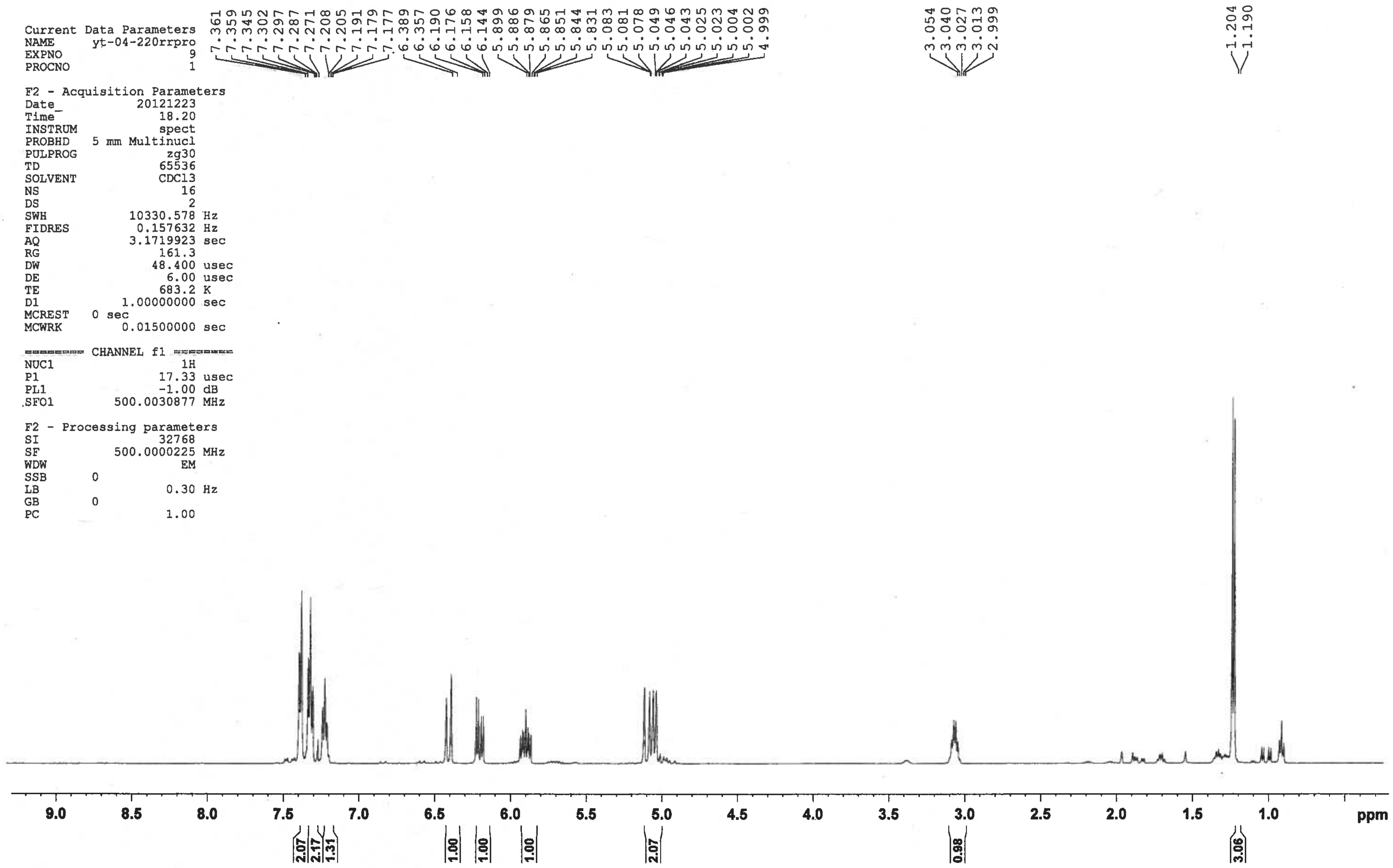

Proton NMR (500 MHz, Chloroform) Spectra of Compound **15g** Using DPPM

Current Data Parameters
NAME yt-04-220rrpro
EXPNO 9
PROCNO 1

F2 - Acquisition Parameters
Date_ 20121223
Time 18.20
INSTRUM spect
PROBHD 5 mm Multinucl
PULPROG zg30
TD 65536
SOLVENT CDCl3
NS 16
DS 2
SWH 10330.578 Hz
FIDRES 0.157632 Hz
AQ 3.1719923 sec
RG 161.3
DW 48.400 usec
DE 6.00 usec
TE 683.2 K
D1 1.00000000 sec
MCREST 0 sec
MCWRK 0.01500000 sec

======== CHANNEL f1 ========
NUC1 1H
P1 17.33 usec
PL1 -1.00 dB
SFO1 500.0030877 MHz

F2 - Processing parameters
SI 32768
SF 500.0000225 MHz
WDW EM
SSB 0
LB 0.30 Hz
GB 0
PC 1.00

Proton NMR (500 MHz, Chloroform) Spectra of Compound **15g**

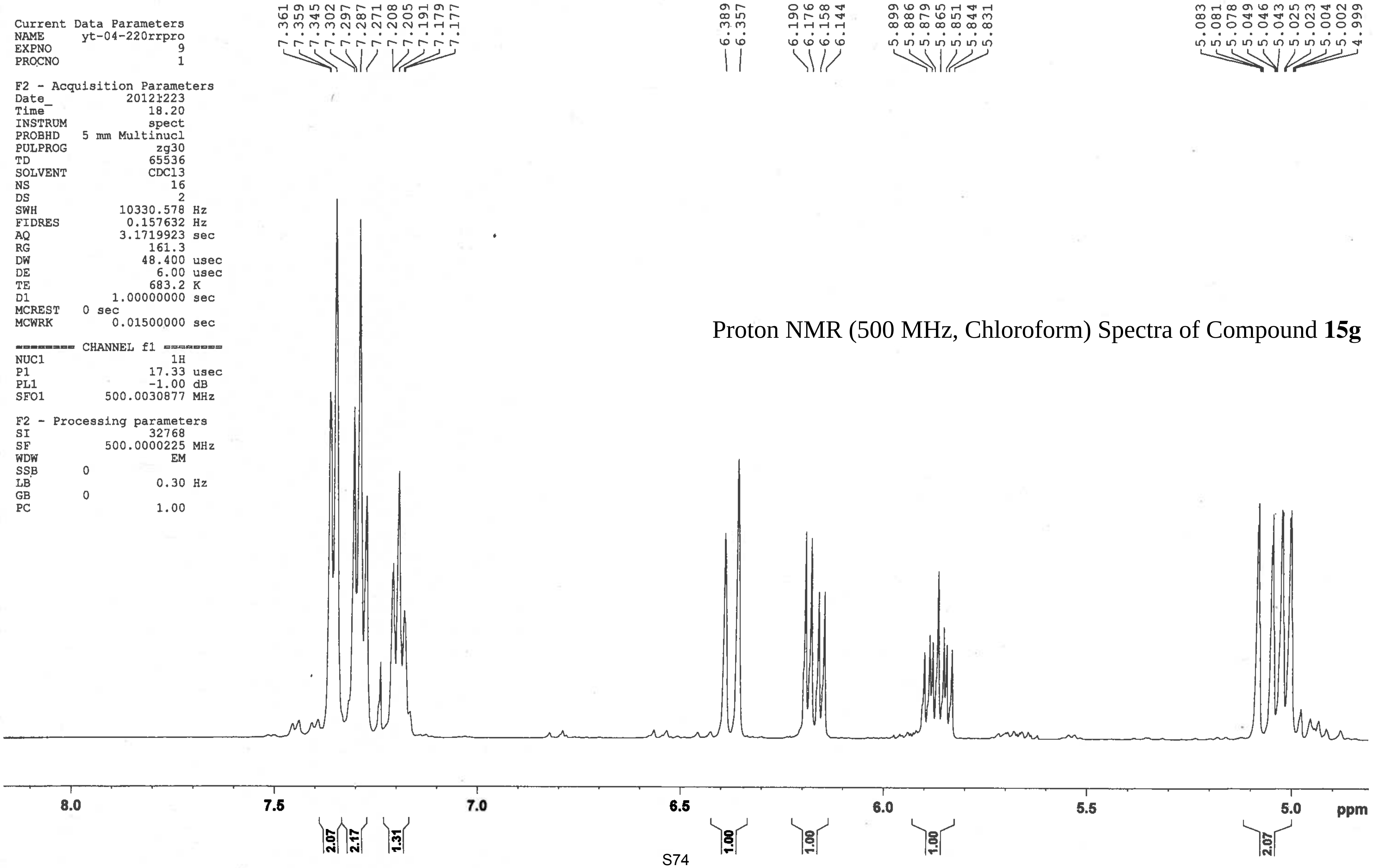

Carbon NMR (125 MHz, Chloroform) Spectra of Compound **15g**

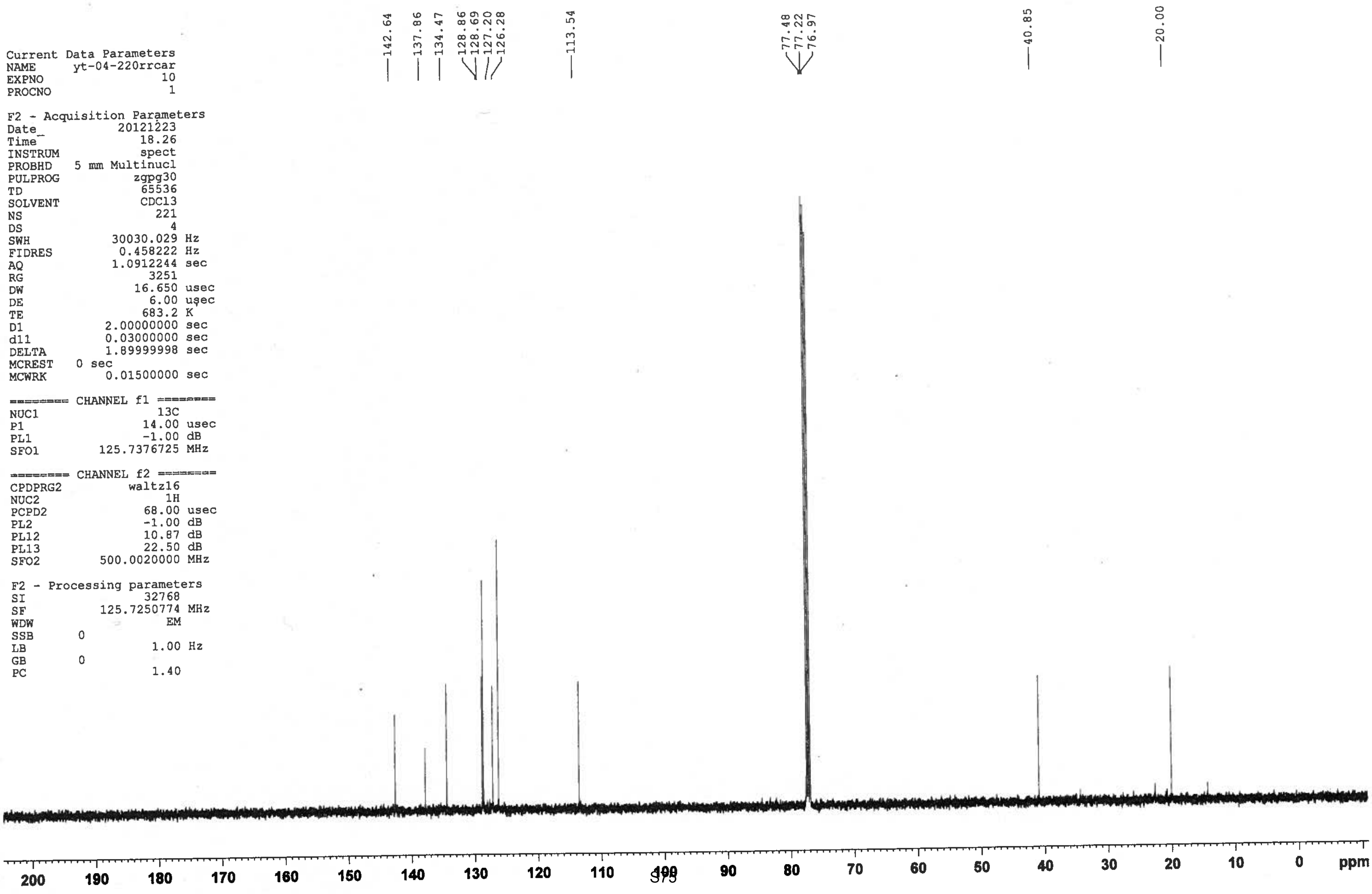

Hydrovinylation of (*E*)-ethyl hexa-3,5-dienoate (12h)

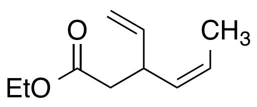

Hydrovinylation of (*E*)-ethyl hexa-3,5-dienoate using [DPPM]CoCl$_2$ (Table 3, Entry 14): To an oven dried round-bottom-flask with a sidearm, was added [DPPM]CoCl$_2$ (31 mg, 0.060 mmol) under argon and was dissolved in dichloromethane (1 mL) at room temperature. Trimethylaluminium as a 2M solution in toluene (0.3 mL, 0.60 mmol) was added dropwise and the color of the solution changed from bluish brown to red brown with the formation of white fumes over the solution. After 2 to 3 minutes, the reaction vessel was carefully evacuated and then refilled with ethylene from a balloon. The reaction vessel was cooled to 0 ^{o}C and (*E*)-ethyl hexa-3,5-dienoate (84 mg, 0.60 mmol) in dichloromethane (1 mL) added under ethylene and the mixture was stirred for 15 h at ambient temperature. The ethylene balloon was removed and 0.1 mL methanol was added into the flask. The reaction solution was diluted with pentane (2 mL) and diethyl ether (2 mL) and warmed to room temperature and subsequently passed through a silica plug. The plug was washed with diethyl ether (3 x 5 mL). Due to low boiling nature of product, toluene solvent was first removed with flash chromatography with neat *n*-pentane as an eluent and the compound was flushed through diethyl ether to collect the product. Removal of solvent yielded the product as slight yellowish oil (84 mg, 84%). GC and NMR analysis showed that the product **(13h)** was essentially pure. ^{1}H NMR (CDCl$_3$, 400 MHz): 1.22 (t, J = 7.2 Hz, 3 H), 1.62 (d, J = 7 Hz, 3 H), 2.30 (dd, J = 8, 15 Hz, 1 H), 2.42 (dd, J = 7, 15 Hz, 1 H), 3.52 - 3.58 (m, 1 H), 4.09 (q, J = 7 Hz, 2 H), 5.01 (dd, J = 17, 24 Hz, 2 H), 5.20 (t, J = 10 Hz, 1 H), 5.47 - 5.55 (m, 1 H), 5.72 (ddd, J = 6.4, 10.4, 17.2 Hz, 1 H). ^{13}C NMR (CDCl$_3$, 125.02 MHz): 13.41, 14.69, 38.23, 40.48, 60.73, 114.54, 125.84, 131.21, 140.02, 172.45. ESI-MS; m/z 191.1061 [M + Na]; mass calculated for $C_{10}H_{16}NaO_2$, 191.1043.

CSP GC (Cyclosil, OT 80 ^{o}C/ isothermal, H_2 carrier gas): Starting material R_T = 19.14 min. R_T for product = 22.84 min and 23.24 min (enantiomers). Enantioselectivity is 92 % with (*S,S)*-[BDPP]CoCl$_2$ (Table 5, entry 14). (Josiphos 2)CoCl$_2$ (5 mol% catalyst) gave no conversion.

This following table shows the additional experiments conducted by using diene (*E*)-ethyl hexa-3,5-dienoate employing 10 mol % catalyst (see also Table 3 in the paper for details).

Entry	Catalyst	Temp/Time	Activator	Conversion	Product (%ee)
1.	DPPMCoCl$_2$	0-16°C/15hr	TMA	100%	(*Z*)-1,4 HV
2.	DPPECoCl$_2$	5°C/9hr	TMA	No reaction	SM
3.	DPPPCoCl$_2$	5°C/7hr	TMA	No reaction	SM
4.	DPPBCoCl$_2$	5°C/11hr	TMA	No reaction	SM
5.	(*S,S*)-BDPPCoCl$_2$	0°C-rt/15hr	TMA	100%	(*Z*)-1,4HV (92% ee)
6.	(*S,S*)-DIOPCoCl$_2$	10°C/12hr	TMA	No reaction	SM
7.	JoshiphosCoCl$_2$	8°C/8hr	TMA	No reaction	SM

Data File C:\CHEM32\1\DATA\YAM\YT-1-CHIRAL 2012-08-20 15-58-05\YT-04-118.D
Sample Name: YT-04-118.D

===

```
Acq. Operator   : yam                          Seq. Line :   1
Acq. Instrument : Babu                          Location : Vial 201
Injection Date  : 8/20/2012 3:59:07 PM               Inj :   1
                                              Inj Volume : 1 µl
Different Inj Volume from Sequence !   Actual Inj Volume : 4 µl
Acq. Method     : C:\CHEM32\1\DATA\YAM\YT-1-CHIRAL 2012-08-20 15-58-05\YT_80_C_CHIRAL.M
Last changed    : 8/20/2012 3:58:05 PM by yam
Analysis Method : C:\CHEM32\1\METHODS\BAKE.M
Last changed    : 11/28/2012 11:00:38 AM by Kendra Dewese
                  (modified after loading)
```

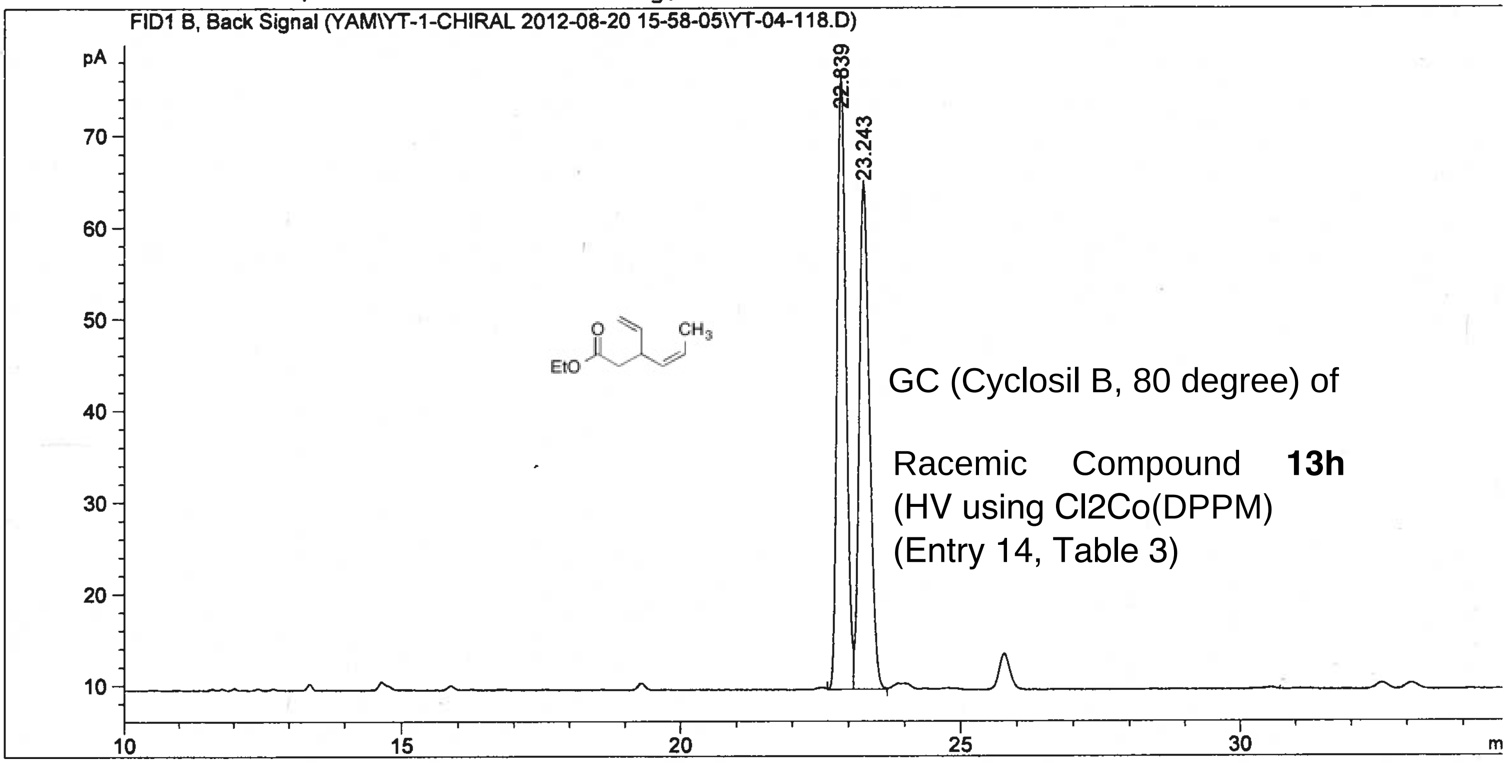

===

Area Percent Report

===

```
Sorted By             :      Signal
Multiplier:                  :      1.0000
Dilution:                    :      1.0000
Use Multiplier & Dilution Factor with ISTDs
```

Signal 1: FID1 B, Back Signal

Peak #	RetTime [min]	Type	Width [min]	Area [pA*s]	Height [pA]	Area %
1	22.839	VV	0.1757	744.05518	66.31979	51.50566
2	23.243	VV	0.1938	700.55347	54.85747	48.49434

Totals : 1444.60864 121.17726

===

*** End of Report ***

Data File C:\CHEM32\1\DATA\YAM\YT-1-CHIRAL 2013-10-10 15-01-23\YT-05-192-4,5.D
Sample Name: YT-05-192-4,5.D

===

Acq. Operator : YAM Seq. Line : 1
Acq. Instrument : Babu Location : Vial 201
Injection Date : 10/10/2013 3:02:38 PM Inj : 1
Inj Volume : 1 µl
Different Inj Volume from Sequence ! Actual Inj Volume : 3 µl
Acq. Method : C:\CHEM32\1\DATA\YAM\YT-1-CHIRAL 2013-10-10 15-01-23\YT_85_C_CHIRAL.M
Last changed : 10/9/2013 4:51:45 PM by YAM
Analysis Method : C:\CHEM32\1\METHODS\YT_85_C_CHIRAL.M
Last changed : 10/10/2013 4:47:26 PM by YAM
(modified after loading)

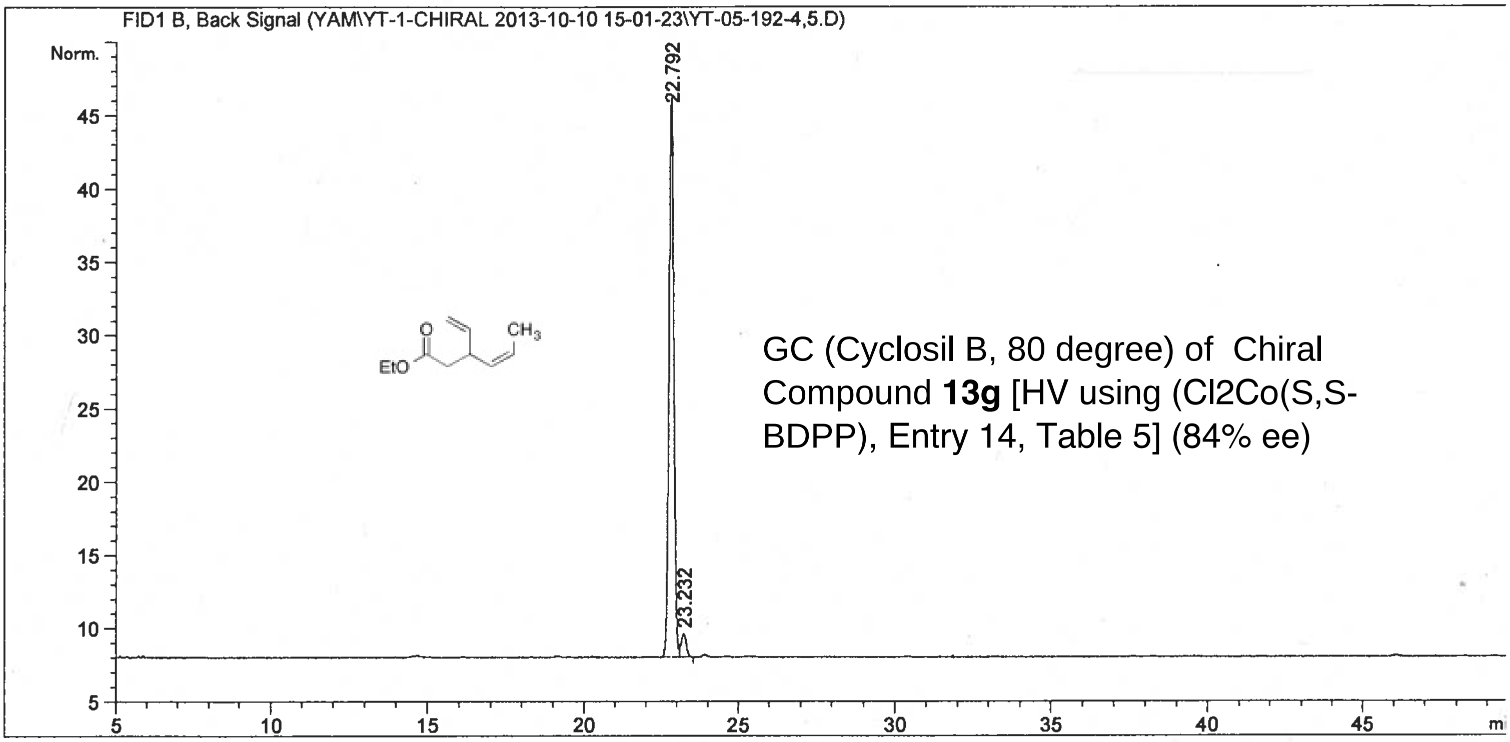

===

Area Percent Report

===

Sorted By : Signal
Multiplier: : 1.0000
Dilution: : 1.0000
Use Multiplier & Dilution Factor with ISTDs

Signal 1: FID1 B, Back Signal

Peak #	RetTime [min]	Type	Width [min]	Area [pA*s]	Height [pA]	Area %
1	22.792	BV	0.1668	415.19519	38.21350	95.71836
2	23.232	VB	0.1779	18.57237	1.60893	4.28164

Totals : 433.76756 39.82243

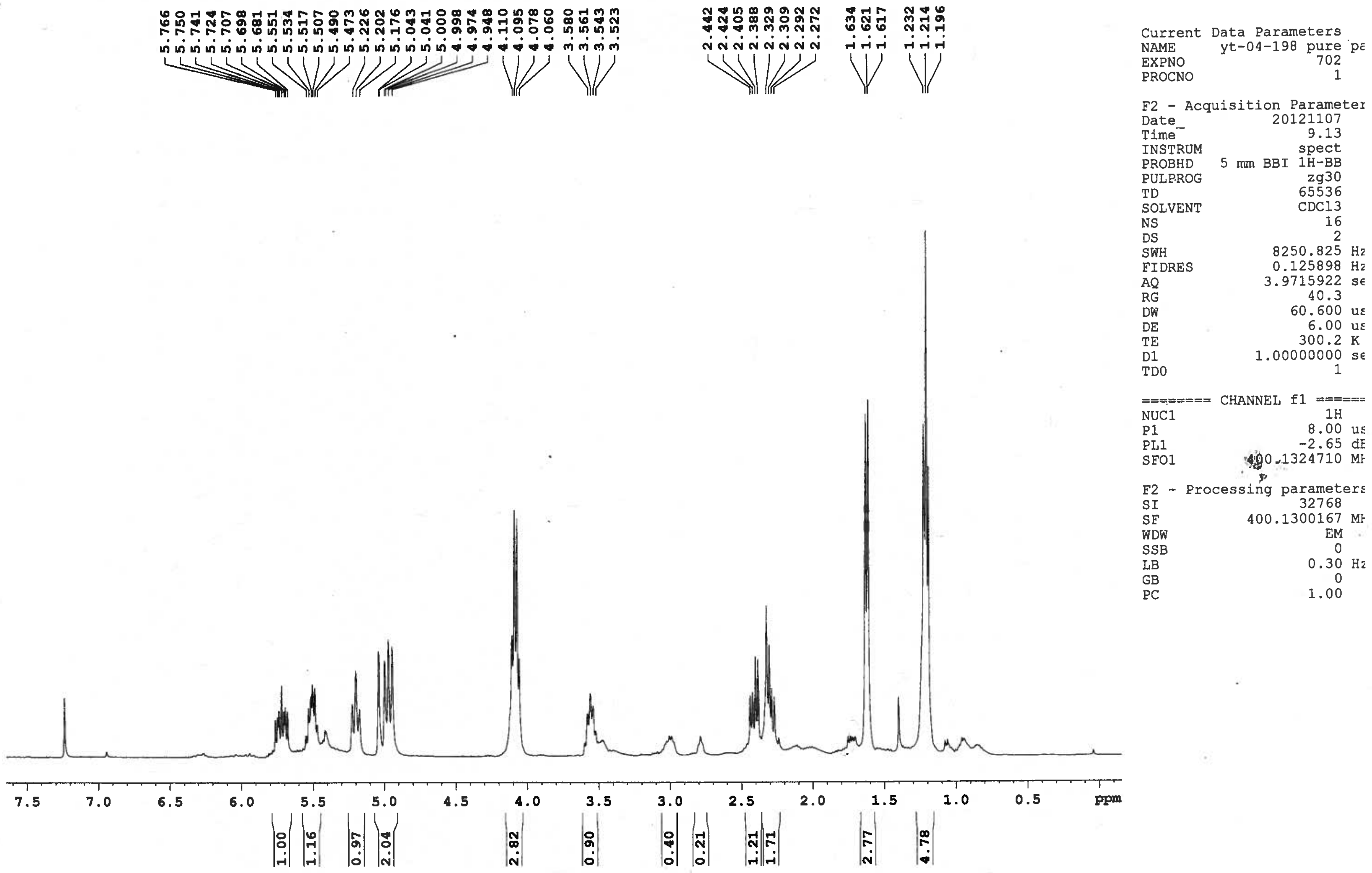

Proton NMR (400 MHz, chloroform) spectrum of compound **13h** (HV using Cl2Co(DPPM)

Proton NMR (400 MHz, Chloroform) Spectra of Compound **13h** Using DPPM (Expanded Alkene Region)

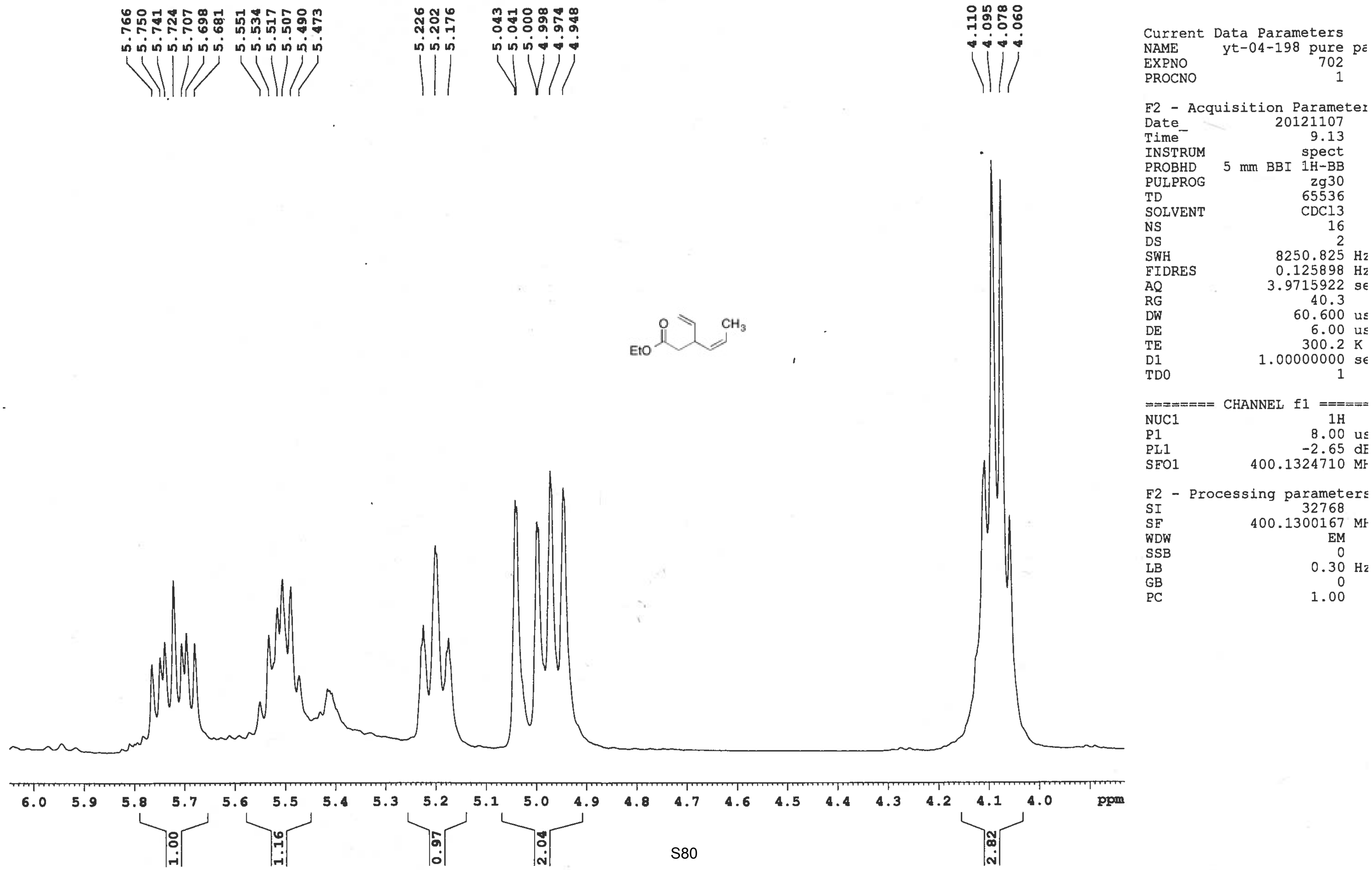

Carbon NMR (125 MHz, Chloroform) Spectra of Compound **13h** Using DPPM

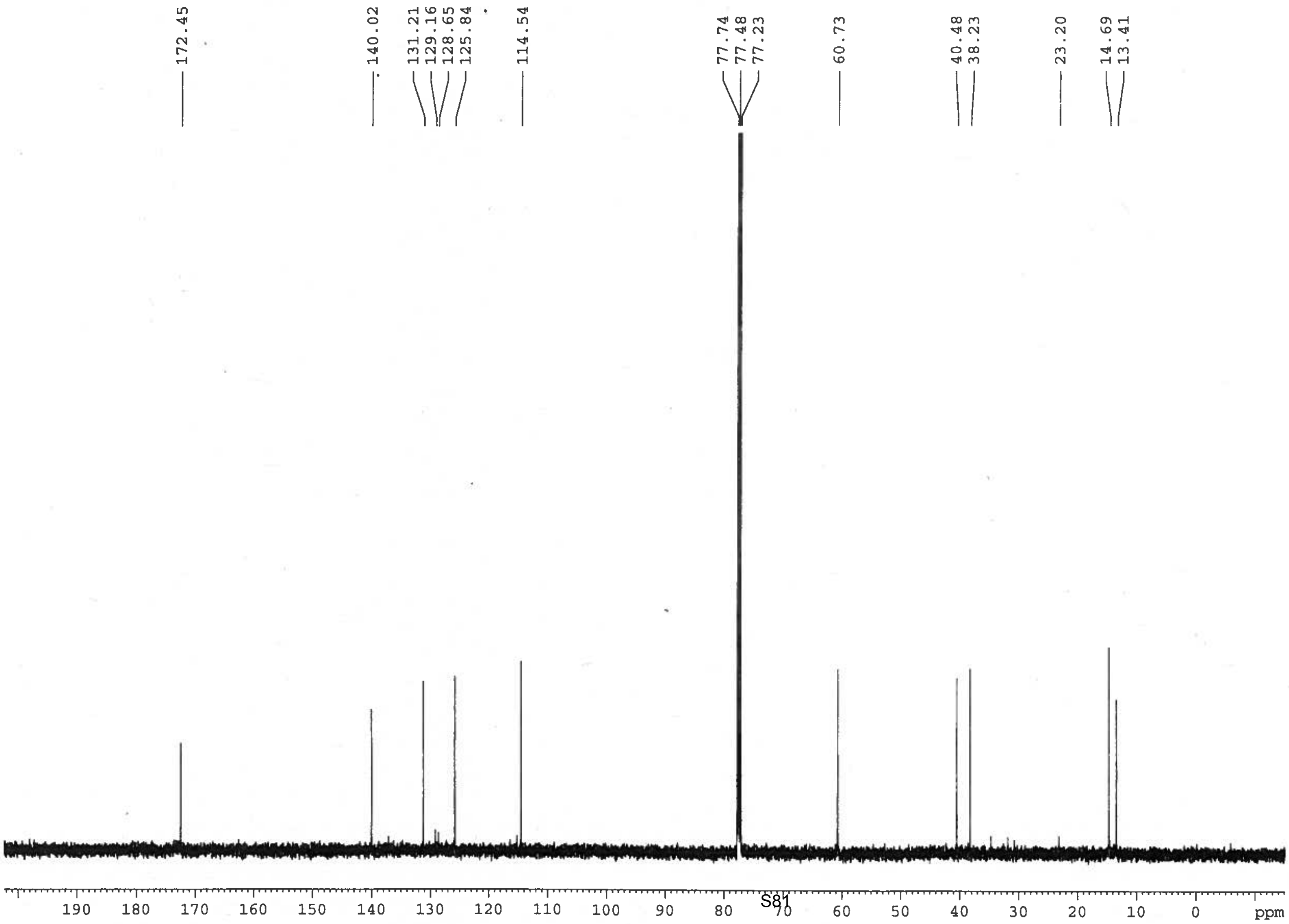


```
Current Data Parameters
NAME      yt-04-198carbon
EXPNO                   3
PROCNO                  1

F2 - Acquisition Parameters
Date_           20121107
Time               18.54
INSTRUM            spect
PROBHD   5 mm Multinucl
PULPROG           zgpg30
TD                 65536
SOLVENT            CDCl3
NS                   882
DS                     4
SWH           30030.029 Hz
FIDRES         0.458222 Hz
AQ            1.0912244 sec
RG                  6502
DW               16.650 usec
DE                 6.00 usec
TE                300.2 K
D1           2.00000000 sec
d11          0.03000000 sec
DELTA        1.89999998 sec
MCREST       0.00000000 sec
MCWRK        0.01500000 sec

======== CHANNEL f1 ========
NUC1                 13C
P1                 14.00 usec
PL1                -1.00 dB
SFO1         125.7376725 MHz

======== CHANNEL f2 ========
CPDPRG2          waltz16
NUC2                  1H
PCPD2              68.00 usec
PL2                -1.00 dB
PL12               10.87 dB
PL13               22.50 dB
SFO2         500.0020000 MHz

F2 - Processing parameters
SI                 32768
SF           125.7250430 MHz
WDW                   EM
SSB                    0
LB                  1.00 Hz
GB                     0
PC                  1.40
```

Hydrovinylation of (*E*)-6-benzyloxy-1,3-hexadiene (12i)

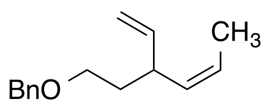

Hydrovinylation of (*E*)-6-benzyloxy-1,3-hexadiene using [dppb]$CoCl_2$ (Table 3, Entry 15). To an oven dried round bottom flask with a sidearm, was added [dppb]$CoCl_2$ (26 mg, 0.047 mmol) and methylaluminoxane (55 mg, 0.94 mmol) under argon and dissolved in dichloromethane (1 mL) at room temperature. After addition of dichloromethane, color of the solution changed from deep blue to red brown with the formation of white fumes over the solution. After 2 to 3 minutes, the reaction vessel was carefully evacuated and then refilled with ethylene balloon. The reaction vessel was cooled to -15 ^{o}C and (*E*)-6-benzyloxy-1,3-hexadiene (88 mg, 0.47 mmol) added under ethylene and the mixture was stirred for 14 h. The ethylene balloon was removed and 0.1 mL methanol was added into the flask. The reaction solution was diluted with pentane (4 mL) and diethyl ether (4 mL) and warmed to room temperature and subsequently passed through a silica plug. The plug was washed with diethyl ether (3 x 5 mL). Flash chromatography (5 % EtOAc in hexane) and removal of solvent yielded the product as slight yellow oil (98 mg, 97%). GC and NMR analysis showed that the product (**13i**) was essentially pure.
^{1}H NMR ($CDCl_3$, 400 MHz): 1.54 - 1.59 (m, 1 H), 1.61 (dd, J = 1.6, 6.8 Hz, 3 H), 1.74 - 1.82 (m, 1 H), 3.21 - 3.29 (m, 1 H), 3.40 - 3.51 (m, 2 H), 4.47 (s, 2 H), 4.94 (dd, J = 1.2, 10 Hz, 1H), 4.99 (dt, J = 1.6, 17.2 Hz, 1 H), 5.19 (td, J = 1.6, 10.8 Hz, 1 H), 5.47 – 5.55 (m, 1 H), 5.71 (ddd, J = 6.8, 10.4, 17.2 Hz), 7.25 - 7.33 (m, aromatic, 5H). ^{13}C NMR ($CDCl_3$, 125.02 MHz): 13.23, 35.17, 38.08, 68.43, 73.21, 113.61, 124.87, 127.69, 127.85, 128.54, 132.49, 138.85, 141.37. ESI-MS; m/z 239.1396 [M + Na]; mass calculated for $C_{15}H_{20}ONa$, 239.1406.

Gas Chromatography: Methylsilicone SP GC (OT 150 ^{o}C/isothermal, He carrier gas) starting material R_T = 10.49 min. R_T for product = 14.37 min. CSP GC (Cyclosil, OT 150 ^{0}C/isothermal, H_2 carrier gas). Starting material R_T = 12.36 min. R_T for product = 15.08 min. and 15.33 min (enantiomers, racemic from DPPB$CoCl_2$). (*S,S*)-DIOP$CoCl_2$ gave 94% ee. Reaction carried out with [(*S,S*)-DIOP]$CoCl_2$ at -20 ^{o}C gave 99% ee, albeit in lower (40%) conversion).

Asymmetric hydrovinylation of (*E*)-6-benzyloxy-1,3-hexadiene using (*S,S*)-[DIOP]$CoCl_2$ (Table 5, Entry 17): To an oven dried round bottom flask with a sidearm, was added (*SS*)-[DIOP]$CoCl_2$ (10 mg, 0.016 mmol) and methylaluminoxane (18 mg, 0.32 mmol) under argon and dissolved in dichloromethane (0.5 mL) at room temperature. After addition of dichloromethane, color of the solution changed from deep blue to red brown with the formation of white fumes over the solution. After 2 to 3 minutes, the reaction vessel was carefully evacuated and then refilled with ethylene balloon. The reaction vessel was cooled to -15 ^{o}C and (*E*)-6-benzyloxy-1,3-hexadiene (30 mg, 0.16 mmol) added under ethylene and the mixture was stirred for 14 h. The ethylene balloon was removed and 0.1 mL methanol was added into the flask. The reaction solution was diluted with pentane (2 mL) and diethyl ether (2 mL) and warmed to room temperature and subsequently passed through a silica plug. The plug was washed with diethyl ether (3 x 5 mL). Flash chromatography (5 % EtOAc in hexane) and removal of solvent yielded the product as slight yellow oil (28 mg, 88%). GC and NMR analysis showed that the product was essentially pure. ^{1}H NMR ($CDCl_3$, 400 MHz): 1.54 - 1.59 (m, 1 H), 1.61 (dd, J = 1.6, 6.8 Hz, 3 H), 1.74 - 1.82 (m, 1 H), 3.21 - 3.29 (m, 1 H), 3.40 - 3.51 (m, 2 H), 4.47 (s, 2 H), 4.94 (dd, J = 1.2, 10 Hz, 1H), 4.99 (dt, J = 1.6, 17.2 Hz, 1 H), 5.19 (td, J = 1.6, 10.8 Hz, 1 H), 5.47 – 5.55 (m, 1 H), 5.71 (ddd, J = 6.8, 10.4, 17.2 Hz), 7.25 - 7.33 (m, aromatic,

5H). ^{13}C NMR ($CDCl_3$, 125.02 MHz): 13.23, 35.17, 38.08, 68.43, 73.21, 113.61, 124.87, 127.69, 127.85, 128.54, 132.49, 138.85, 141.37. ESI-MS; m/z 239.1396 [M + Na]; mass calculated for $C_{15}H_{20}ONa$, 239.1406.

[(*S,S*)-DIOP]$CoCl_2$ gave 94% ee at full conversion.

This following table shows the percent conversion and percent ee's obtained using other catalysts.

Catalyst	% ee	% conversion
(*S,S*)-[BDPP]$CoCl_2$	92	100
(*S,S*)-[DIOP]$CoCl_2$	99[a]	40
**L18** Joshiphos 2	71	92

[a] at -10 °C/6 h, 100%, 94% ee.

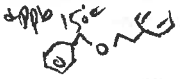

YT-04-09/dppbcocl2/MAO

*
*
*
*
*
*
* RUN # 360 APR 15, 1901 01:41:48

START

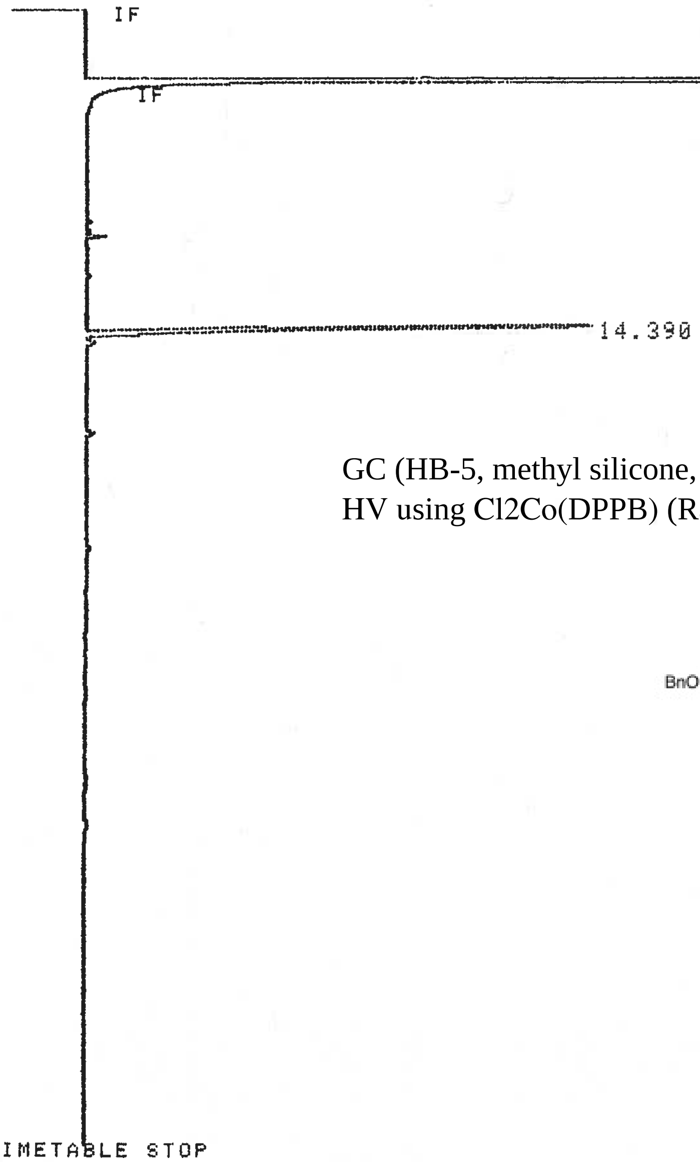

TIMETABLE STOP

GC (HB-5, methyl silicone, 150 degree) of Compound **13i** HV using Cl2Co(DPPB) (Racemic) Entry 15, Table 3)

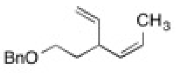

13i

RUN# 360 APR 15, 1901 01:41:48

AREA%

RT	AREA	TYPE	WIDTH	AREA%
14.390	215340	BP	.125	100.00000

TOTAL AREA= 215340
MUL FACTOR=1.0000E+00

*
*

Data File C:\CHEM32\1\DATA\YAM\YT-1-CHIRAL 2013-02-25 11-50-11\YT-04-09DPPBRR.D
Sample Name: YT-04-09DPPBRR.D

===

Acq. Operator : YAM Seq. Line : 1
Acq. Instrument : Babu Location : Vial 201
Injection Date : 2/25/2013 11:51:19 AM Inj : 1
Inj Volume : 1 µl
Different Inj Volume from Sequence ! Actual Inj Volume : 3 µl
Acq. Method : C:\CHEM32\1\DATA\YAM\YT-1-CHIRAL 2013-02-25 11-50-11\YT_85_C_CHIRAL.M
Last changed : 2/25/2013 10:26:04 AM by YAM
Analysis Method : C:\CHEM32\1\METHODS\35_CYCLODEX-B.M
Last changed : 9/9/2013 11:17:53 AM by Kendra
(modified after loading)

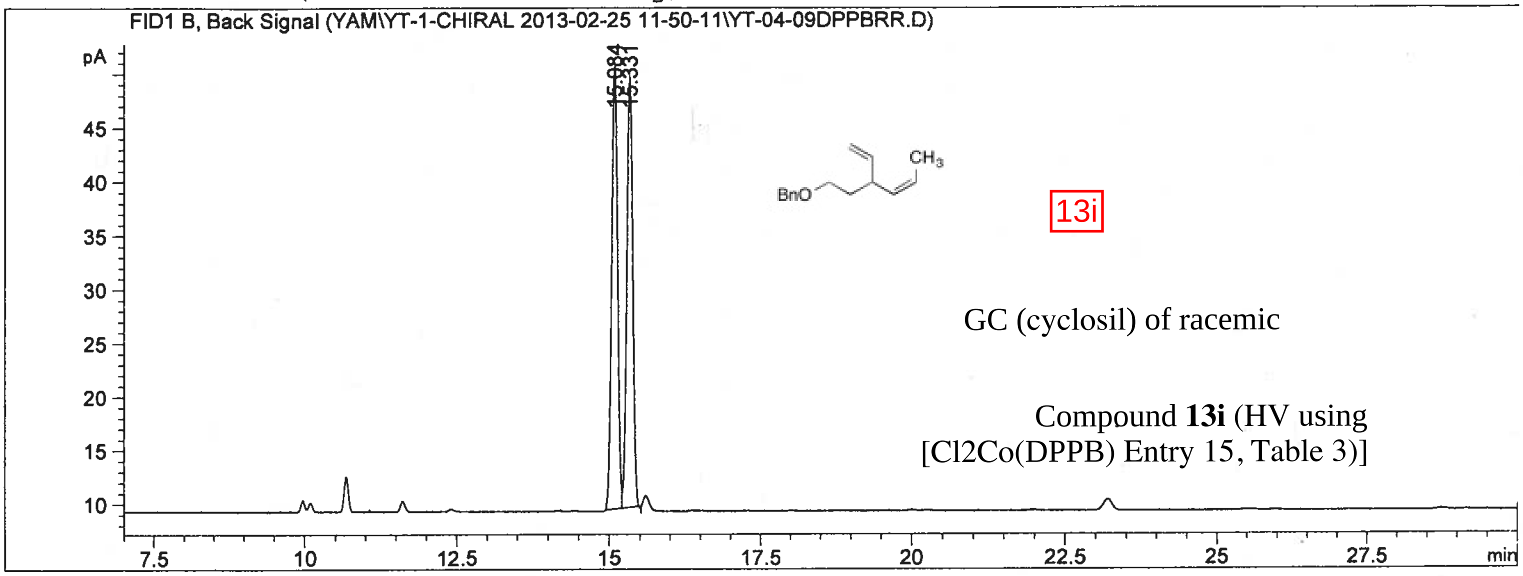

13i

GC (cyclosil) of racemic

Compound **13i** (HV using [Cl2Co(DPPB) Entry 15, Table 3)]

===
Area Percent Report
===

Sorted By : Signal
Multiplier: : 1.0000
Dilution: : 1.0000
Use Multiplier & Dilution Factor with ISTDs

Signal 1: FID1 B, Back Signal

Peak #	RetTime [min]	Type	Width [min]	Area [pA*s]	Height [pA]	Area %
1	15.084	BV	0.1036	269.52896	41.15466	50.19640
2	15.331	VB	0.1052	267.41983	39.99708	49.80360

Totals : 536.94879 81.15174

===
*** End of Report ***

ata File C:\CHEM32\1\DATA\YAM\YT-1-CHIRAL 2012-08-22 15-36-29\YT-04-138SMR.D
ample Name: YT-04-138SMR.D

===

Acq. Operator : YAM   Seq. Line : 1
Acq. Instrument : Babu   Location : Vial 201
Injection Date : 8/22/2012 3:37:31 PM   Inj : 1
                                                       Inj Volume : 1 µl
Different Inj Volume from Sequence ! Actual Inj Volume : 5 µl
Acq. Method : C:\CHEM32\1\DATA\YAM\YT-1-CHIRAL 2012-08-22 15-36-29\YT_80_C_CHIRAL.M
Last changed : 8/22/2012 3:36:29 PM by YAM
Analysis Method : C:\CHEM32\1\METHODS\JP_NONADIENE.M
Last changed : 2/25/2013 10:41:53 AM by CHANDRANI
(modified after loading)

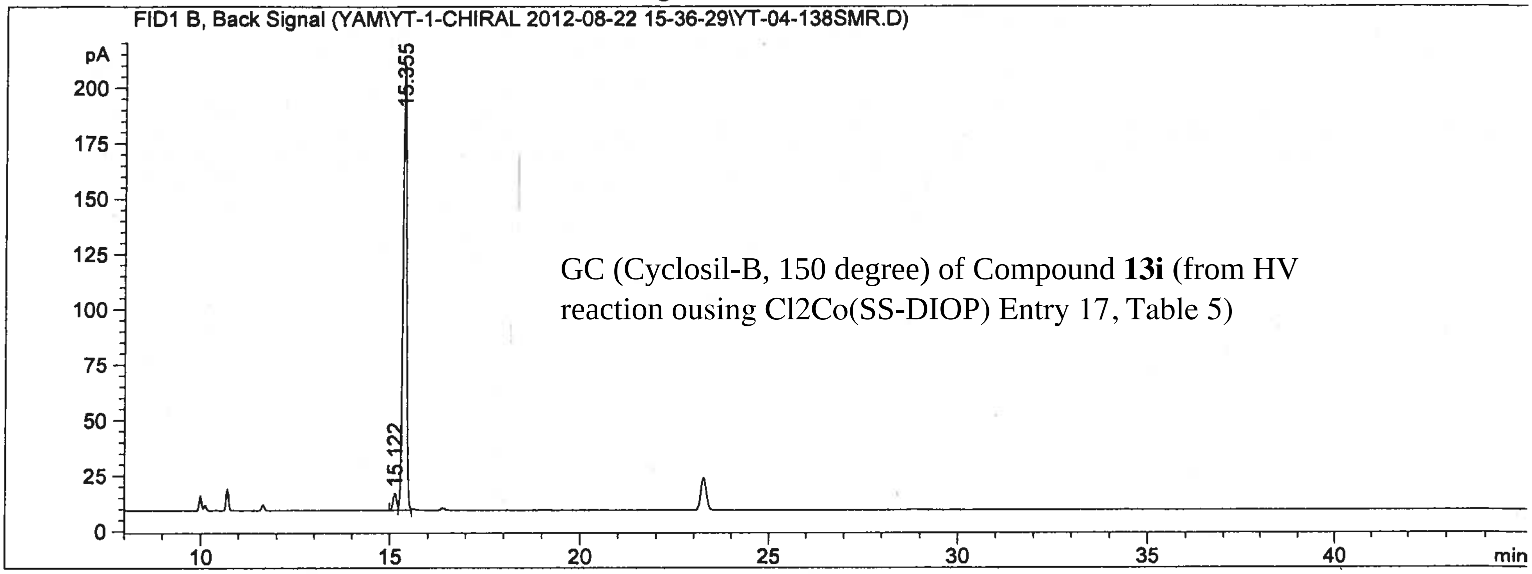

===

Area Percent Report

===

Sorted By : Signal
Multiplier: : 1.0000
Dilution: : 1.0000
Use Multiplier & Dilution Factor with ISTDs

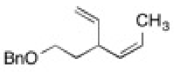

13i

Signal 1: FID1 B, Back Signal

Peak #	RetTime [min]	Type	Width [min]	Area [pA*s]	Height [pA]	Area %
1	15.122	BV	0.1015	49.09645	7.70513	3.49769
2	15.355	VV	0.1059	1354.58533	200.70699	96.50231

Totals : 1403.68178 208.41211

===

*** End of Report ***

YT-03-58/SS BDPP

```
*   TIME 50   STOP
*
*
*   RUN #   358      APR 14, 1901   23:02:58
START
```

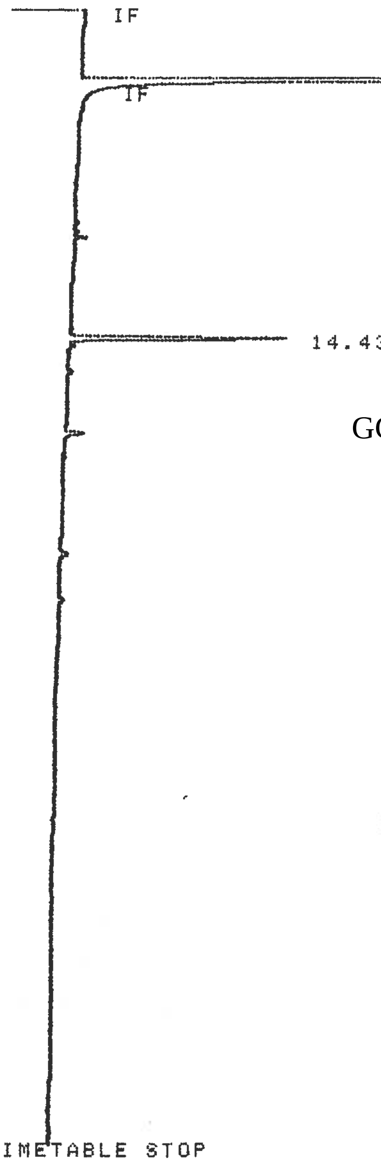

GC (methyl silicone, 150 degree) of Compound **13i** Using SS-BDPP (92%ee)

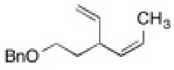

13i

```
TIMETABLE STOP


RUN#    358           APR 14, 1901   23:02:58

AREA%
       RT        AREA TYPE   WIDTH        AREA%
  14.439      97878    PV    .131   100.00000

TOTAL AREA=   97878
MUL FACTOR=1.0000E+00
```

ata File C:\CHEM32\1\DATA\YAM\YT-1-CHIRAL 2013-02-25 09-12-23\YT-03-58RR.D
ample Name: YT-03-58RR.D

```
=====================================================================
Acq. Operator   : YAM                              Seq. Line :   1
Acq. Instrument : Babu                              Location : Vial 201
Injection Date  : 2/25/2013 9:13:37 AM                   Inj :   1
                                                  Inj Volume : 1 µl
Different Inj Volume from Sequence !       Actual Inj Volume : 3 µl
Acq. Method     : C:\CHEM32\1\DATA\YAM\YT-1-CHIRAL 2013-02-25 09-12-23\YT_85_C_CHIRAL.M
Last changed    : 2/25/2013 9:12:23 AM by YAM
Analysis Method : C:\CHEM32\1\METHODS\YT_85_C_CHIRAL.M
Last changed    : 2/25/2013 10:22:47 AM by YAM
                  (modified after loading)
```

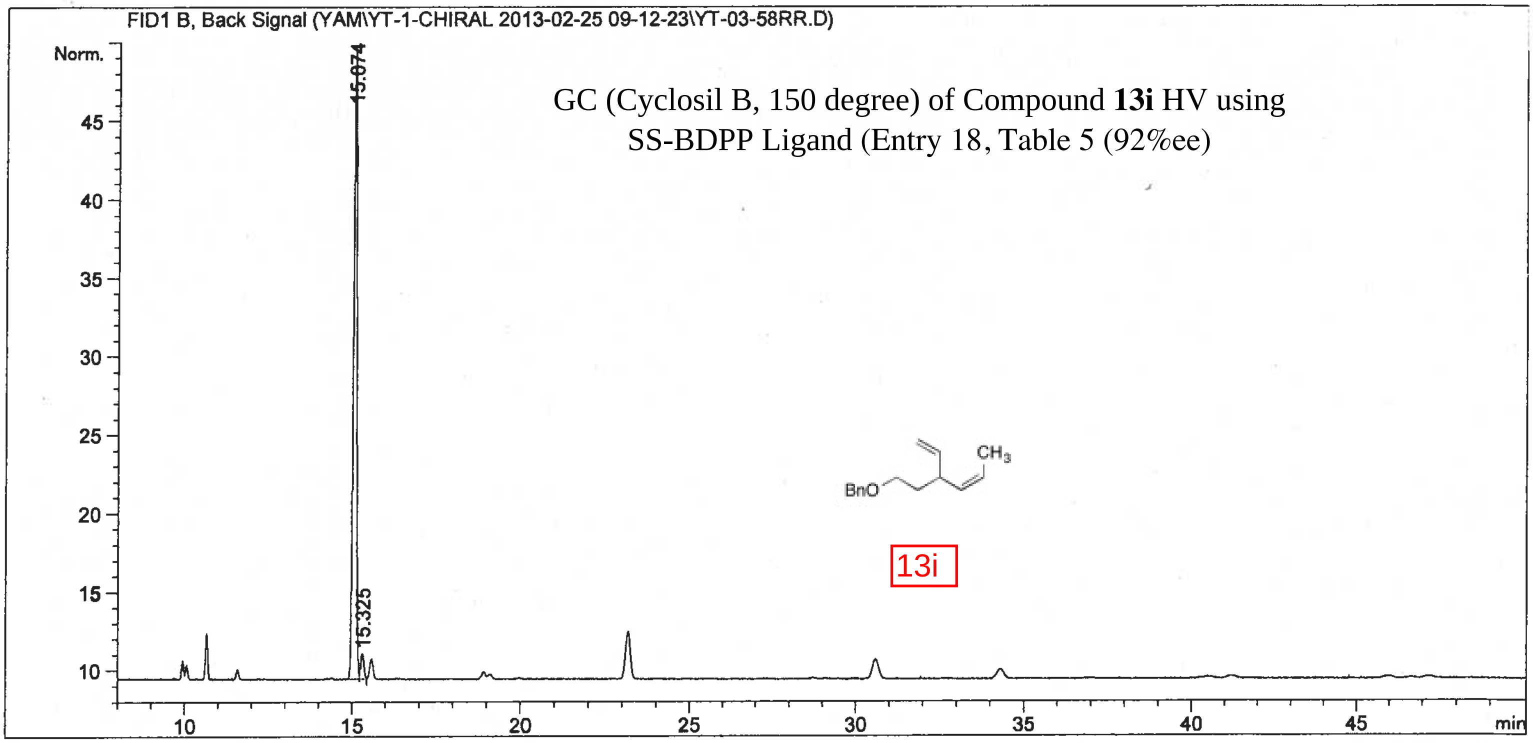

===
Area Percent Report
===

Sorted By : Signal
Multiplier: : 1.0000
Dilution: : 1.0000
Use Multiplier & Dilution Factor with ISTDs

Signal 1: FID1 B, Back Signal

Peak #	RetTime [min]	Type	Width [min]	Area [pA*s]	Height [pA]	Area %
1	15.074	BV	0.1042	265.28748	40.19469	96.03125
2	15.325	VV	0.1073	10.96372	1.62892	3.96875

Totals : 276.25119 41.82362

Proton NMR (400 MHz, D-chloroform) Spectra of Compound **13i**

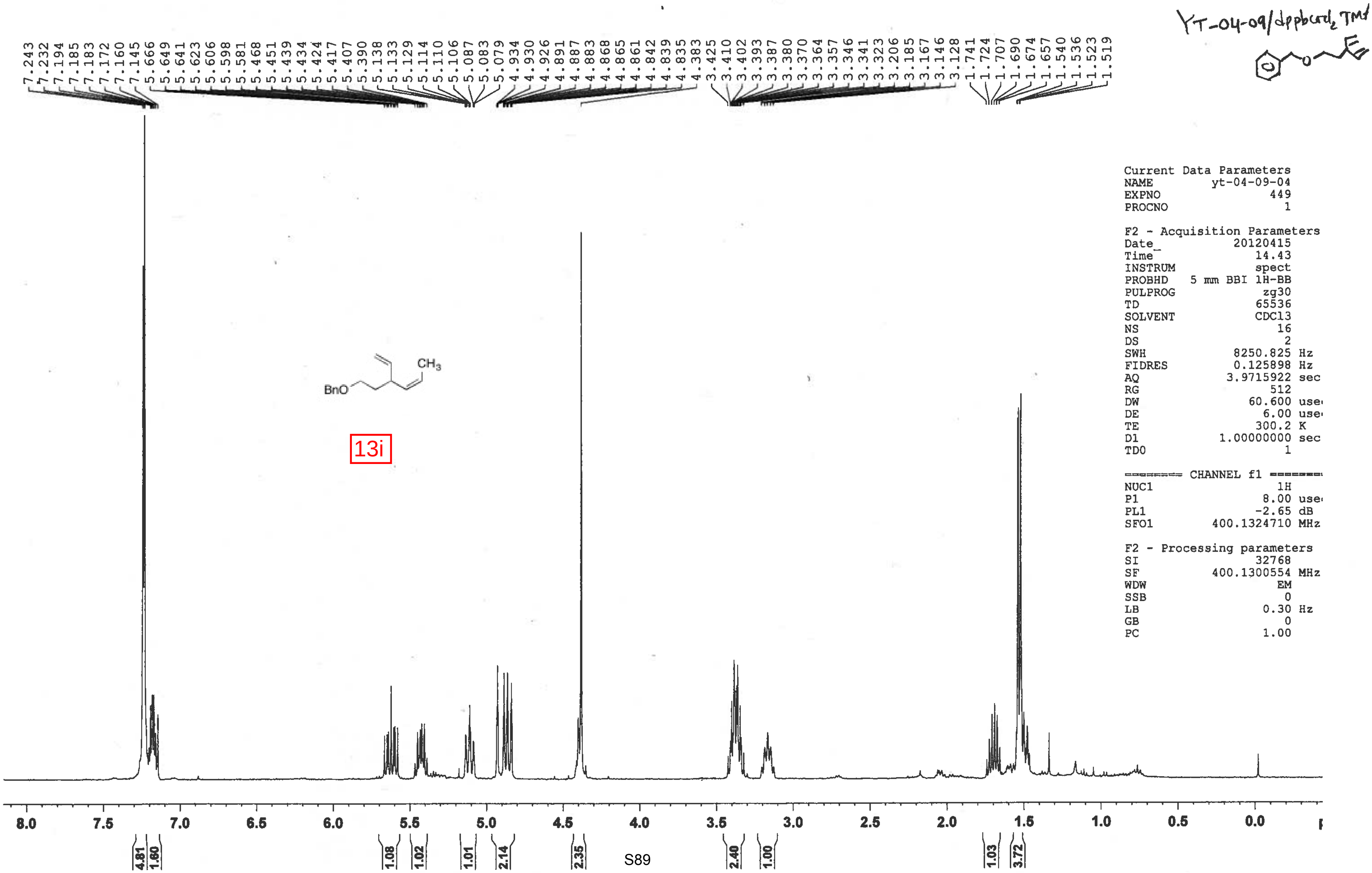

Proton NMR (400 MHz, D-chloroform) spectrum of Compound **13i** (HV Using SS-DIOP) (Alkene Region Expansion)

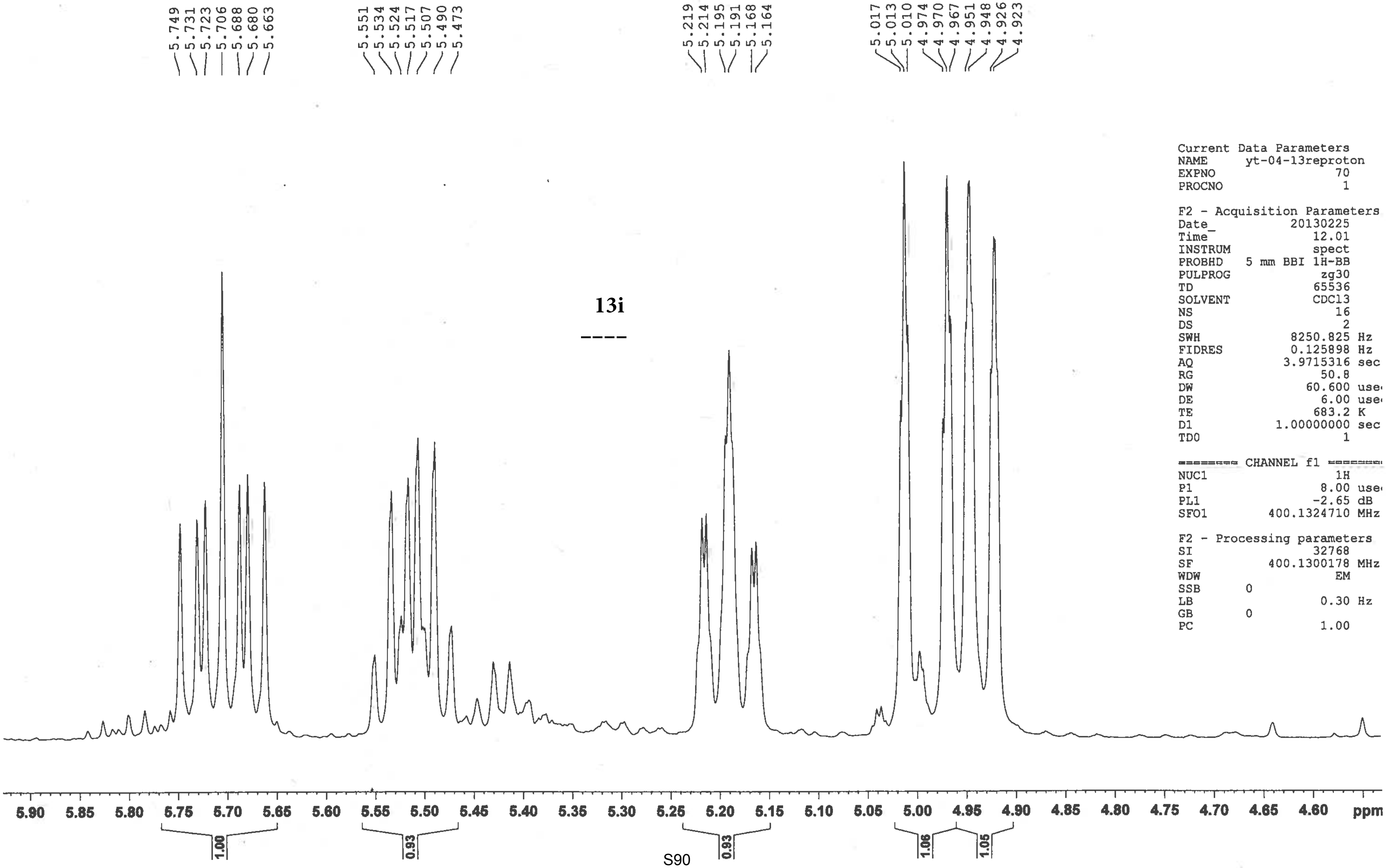

Carbon NMR (400 MHz, D-chloroform) spectrum of Compound **13i** (HV Using SS-DIOP)

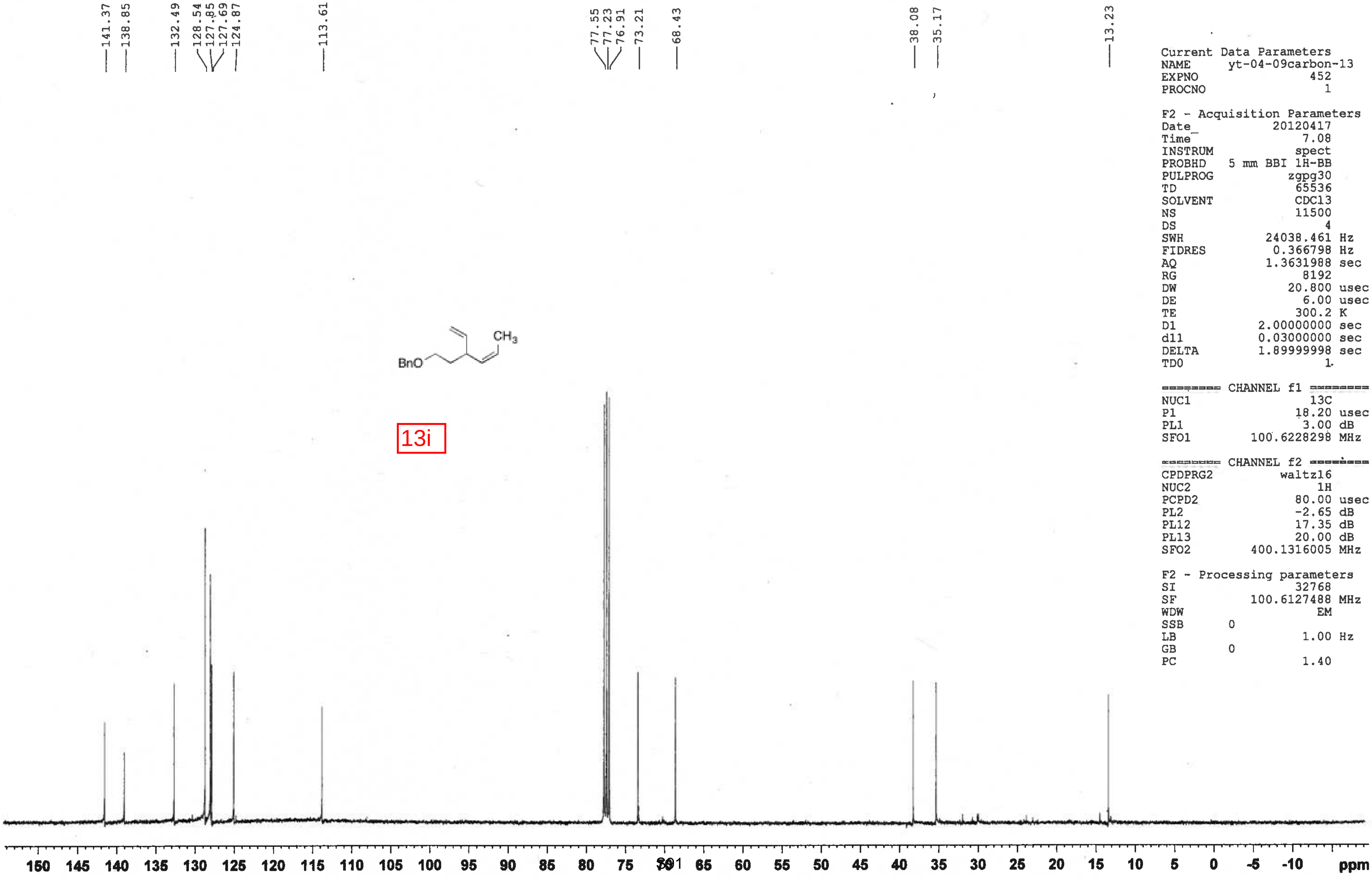

13i

```
Current Data Parameters
NAME       yt-04-09carbon-13
EXPNO                    452
PROCNO                     1

F2 - Acquisition Parameters
Date_               20120417
Time                    7.08
INSTRUM                spect
PROBHD      5 mm BBI 1H-BB
PULPROG              zgpg30
TD                     65536
SOLVENT                CDCl3
NS                     11500
DS                         4
SWH               24038.461 Hz
FIDRES             0.366798 Hz
AQ                1.3631988 sec
RG                      8192
DW                    20.800 usec
DE                      6.00 usec
TE                     300.2 K
D1                2.00000000 sec
d11               0.03000000 sec
DELTA             1.89999998 sec
TD0                        1

======== CHANNEL f1 ========
NUC1                     13C
P1                     18.20 usec
PL1                     3.00 dB
SFO1            100.6228298 MHz

======== CHANNEL f2 ========
CPDPRG2              waltz16
NUC2                      1H
PCPD2                  80.00 usec
PL2                    -2.65 dB
PL12                   17.35 dB
PL13                   20.00 dB
SFO2            400.1316005 MHz

F2 - Processing parameters
SI                     32768
SF              100.6127488 MHz
WDW                       EM
SSB       0
LB                      1.00 Hz
GB        0
PC                      1.40
```

Hydrovinylation of (*E/Z*)-hexa-3,5-dienylbenzene (12j)

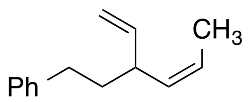

Hydrovinylation of (*E/Z*)-hexa-3,5-dienylbenzene using[dppb]$CoCl_2$ (Table 3, Entry 16). To an oven dried round bottom flask with a sidearm, was added [dppb]$CoCl_2$ (18 mg, 0.032 mmol) and methylaluminoxane (37 mg, 0.64 mmol) under argon. The color of the solution changed from deep blue to red brown with the formation of white fumes over the addition of dichloromethane solvent (1 mL). After 2 to 3 minutes, the reaction vessel was carefully evacuated and then refilled with ethylene balloon. The reaction vessel was kept at -10 ^oC and (*E/Z*)-hexa-3,5-dienylbenzene (52 mg, 0.32 mmol), mixture of *E*:*Z* in the ratio of 53:47, was added via syringe under ethylene and the mixture was stirred for 8 h. The ethylene balloon was removed and 0.1 mL methanol was added into the flask. The reaction solution was diluted with pentane (2 mL) and warmed to room temperature and subsequently passed through a silica plug. The plug was washed with pentane (3 x 5 mL). Removal of the solvent yielded the product as colorless oil (51 mg, 84%). GC and NMR analysis showed that the product (**13j**) was essentially pure. ^1H NMR ($CDCl_3$, 400 MHz): 1.58 (dd, J = 7, 2 Hz, 3 H), 1.57 – 1.74 (m, 1 H), 1.75 – 1.79 (m, 1 H), 2.54 – 2.63 (m, 2 H), 3.03 - 3.06 (m, 1 H), 4.94 – 5.02 (m, 2 H), 5.22 - 5.29 (m, 1 H), 5.49 – 5.58 (m, 1 H), 5.72 (ddd, J = 17, 10, 7 Hz, 1 H), 7.14 – 7.27 (m, 5 H, aromatic). ^{13}C NMR ($CDCl_3$, 125.02 MHz): 13.33, 33.64, 37.14, 41.03, 113.65, 124.72, 125.87, 128.49, 128.67, 132.91, 141.56, 142.72.

Gas Chromatography: CSP GC (Cyclosil, OT 100 ^oC/ isothermal, H_2 carrier gas): starting material R_T = 37.70 min. for *E*-diene and 36.07 min. for *Z* diene. R_T for product = 54.64 min and 57.65 min (racemic).

Data File C:\CHEM32\1\DATA\DUVVURI\YT-1-CHIRAL 2014-04-04 12-30-08\YT-06-122.D
Sample Name: YT-06-122.D

===

Acq. Operator	: YAM	Seq. Line :	1
Acq. Instrument	: Babu	Location :	Vial 201
Injection Date	: 4/4/2014 12:31:15 PM	Inj :	1
		Inj Volume :	1 µl

Different Inj Volume from Sequence !   Actual Inj Volume : 4 µl
Acq. Method   : C:\CHEM32\1\DATA\DUVVURI\YT-1-CHIRAL 2014-04-04 12-30-08\YT_85_C_CHIRAL.M
Last changed   : 4/4/2014 12:30:06 PM by YAM
Analysis Method : C:\CHEM32\1\METHODS\YT_85_C_CHIRAL.M
Last changed   : 4/4/2014 12:30:06 PM by YAM

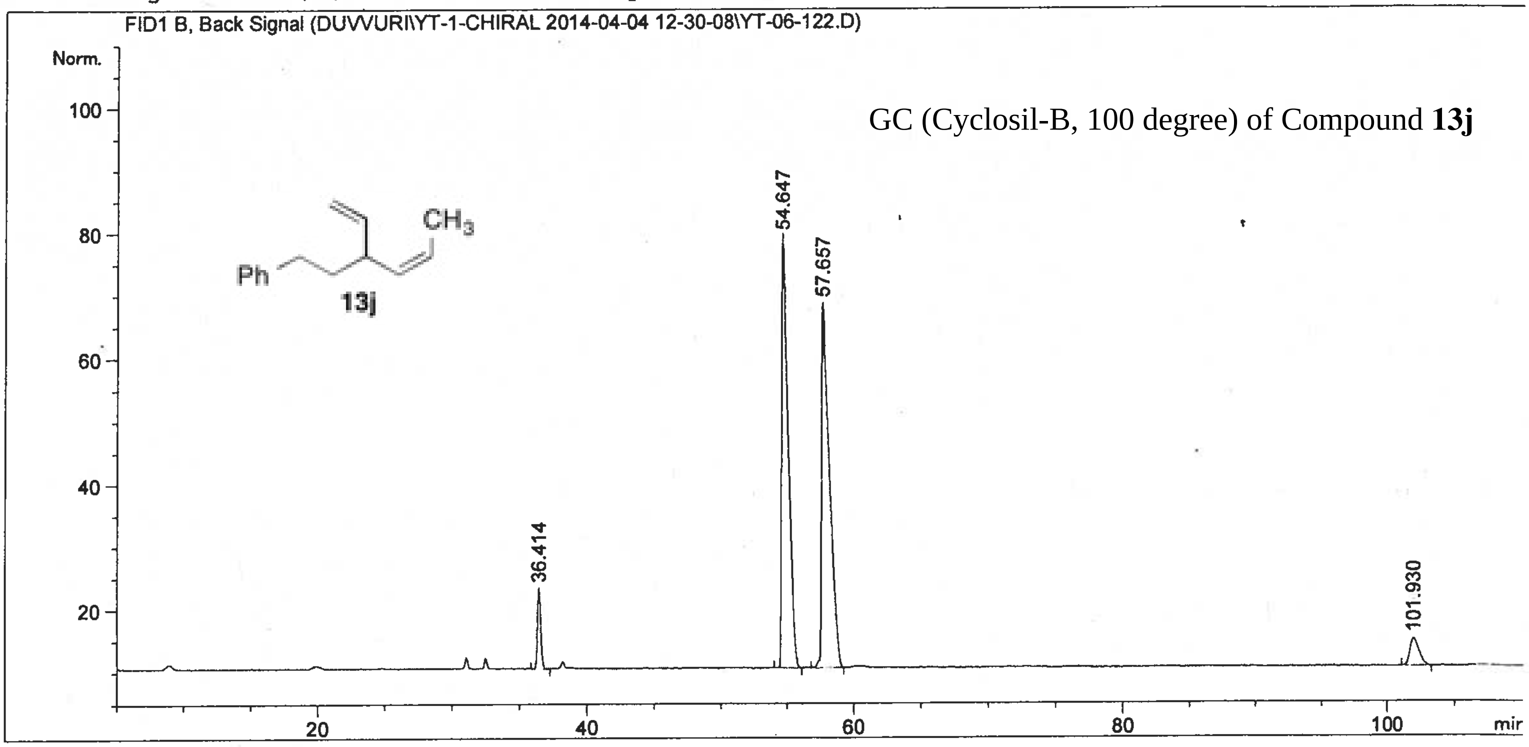

===

Area Percent Report

===

Sorted By   :   Signal
Multiplier: :   1.0000
Dilution: :   1.0000
Use Multiplier & Dilution Factor with ISTDs

Signal 1: FID1 B, Back Signal

Peak #	RetTime [min]	Type	Width [min]	Area [pA*s]	Height [pA]	Area %
1	36.414	BB	0.2539	217.92847	12.84430	4.19654
2	54.647	BB	0.4428	2367.73975	68.85545	45.59434
3	57.657	BB	0.5236	2403.51611	58.17712	46.28327
4	101.930	BB	0.5638	203.87204	4.34213	3.92586

Proton NMR (400 MHz, D-Chloroform) spectrum of compound **13j** (HV using Cl2Co(DPPB) (Entry 16, Table 3)

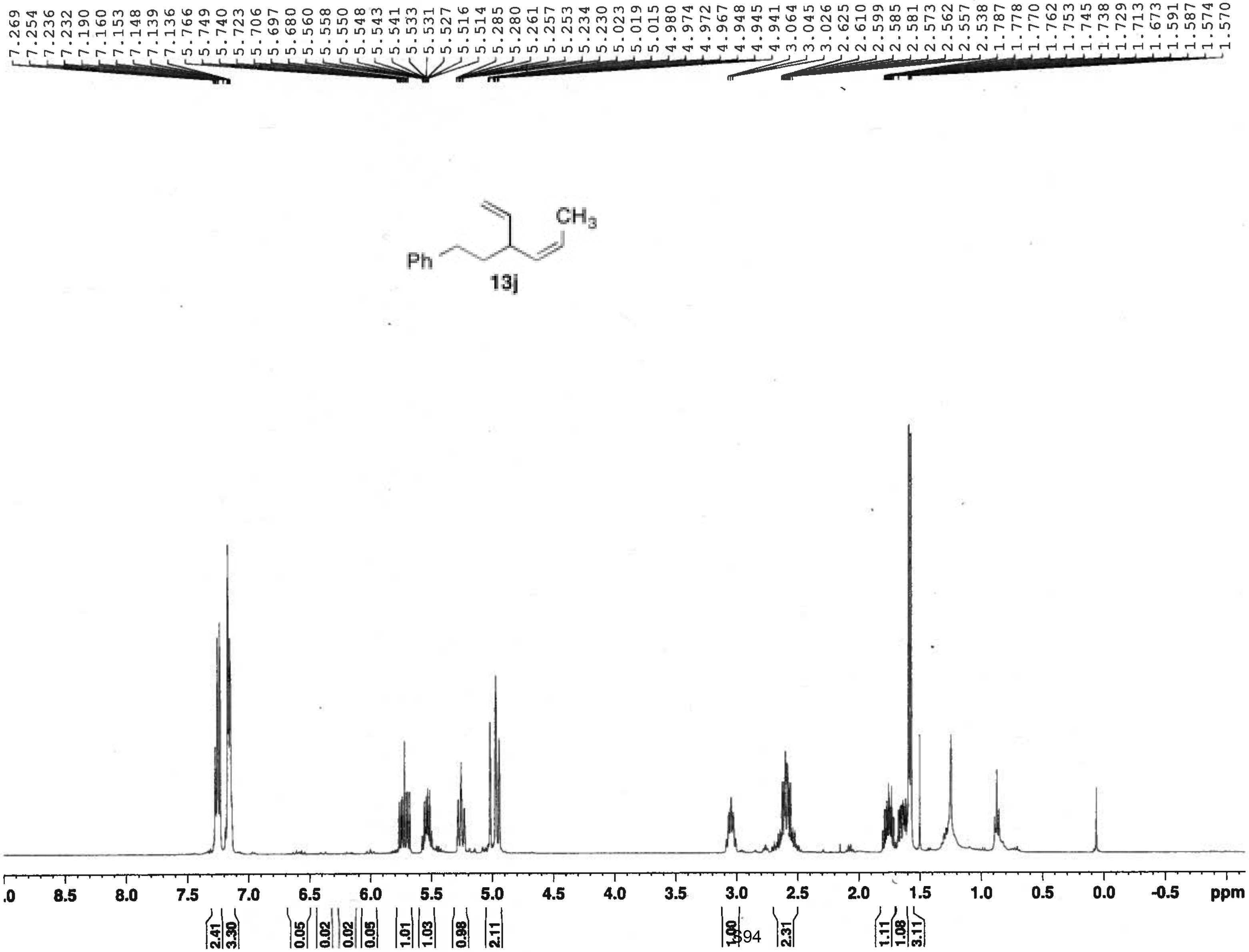

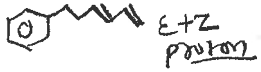


```
Current Data Parameters
NAME            YT-06-122
EXPNO                   1
PROCNO                  1

F2 - Acquisition Parameters
Date_            20140404
Time                19.17
INSTRUM             spect
PROBHD   5 mm PABBO BB/
PULPROG              zg30
TD                  65536
SOLVENT             CDCl3
NS                     16
DS                      2
SWH              8223.685 Hz
FIDRES           0.125483 Hz
AQ              3.9845889 sec
RG                 112.47
DW                 60.800 use
DE                   6.50 use
TE                  300.0 K
D1             1.00000000 sec
TD0                     1

======== CHANNEL f1 ========
NUC1                   1H
P1                  15.00 use
PLW1          12.52000046 W
SFO1          400.1724712 MHz

F2 - Processing parameters
SI                  65536
SF            400.1700196 MHz
WDW                    EM
SSB       0
LB                   0.30 Hz
GB        0
PC                   1.00
```

Proton NMR (400 MHz, D-Chloroform) spectrum of compound **13j** (HV using Cl2Co(DPPB(Alkene Region Expansion)

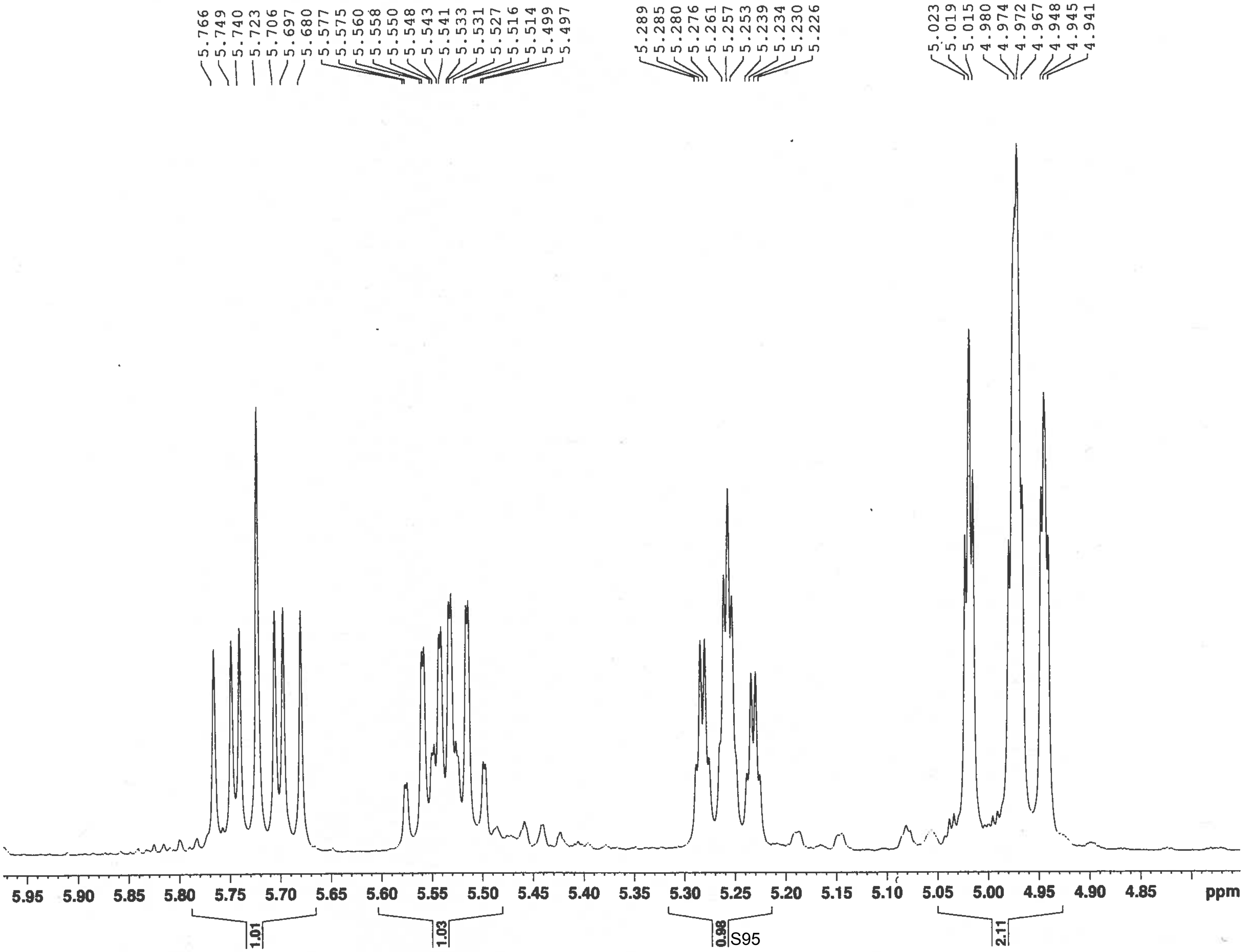

Current Data Parameters
NAME YT-06-122
EXPNO 1
PROCNO 1

F2 - Acquisition Parameters
Date_ 20140404
Time 19.17
INSTRUM spect
PROBHD 5 mm PABBO BB/
PULPROG zg30
TD 65536
SOLVENT CDCl3
NS 16
DS 2
SWH 8223.685 Hz
FIDRES 0.125483 Hz
AQ 3.9845889 sec
RG 112.47
DW 60.800 use
DE 6.50 use
TE 300.0 K
D1 1.00000000 sec
TD0 1

======= CHANNEL f1 =======
NUC1 1H
P1 15.00 use
PLW1 12.52000046 W
SFO1 400.1724712 MHz

F2 - Processing parameters
SI 65536
SF 400.1700196 MHz
WDW EM
SSB 0
LB 0.30 Hz
GB 0
PC 1.00

Carbon NMR (400 MHz, D-chloroform) spectrum of compound 13j (HV using Cl2Co(DPPB)

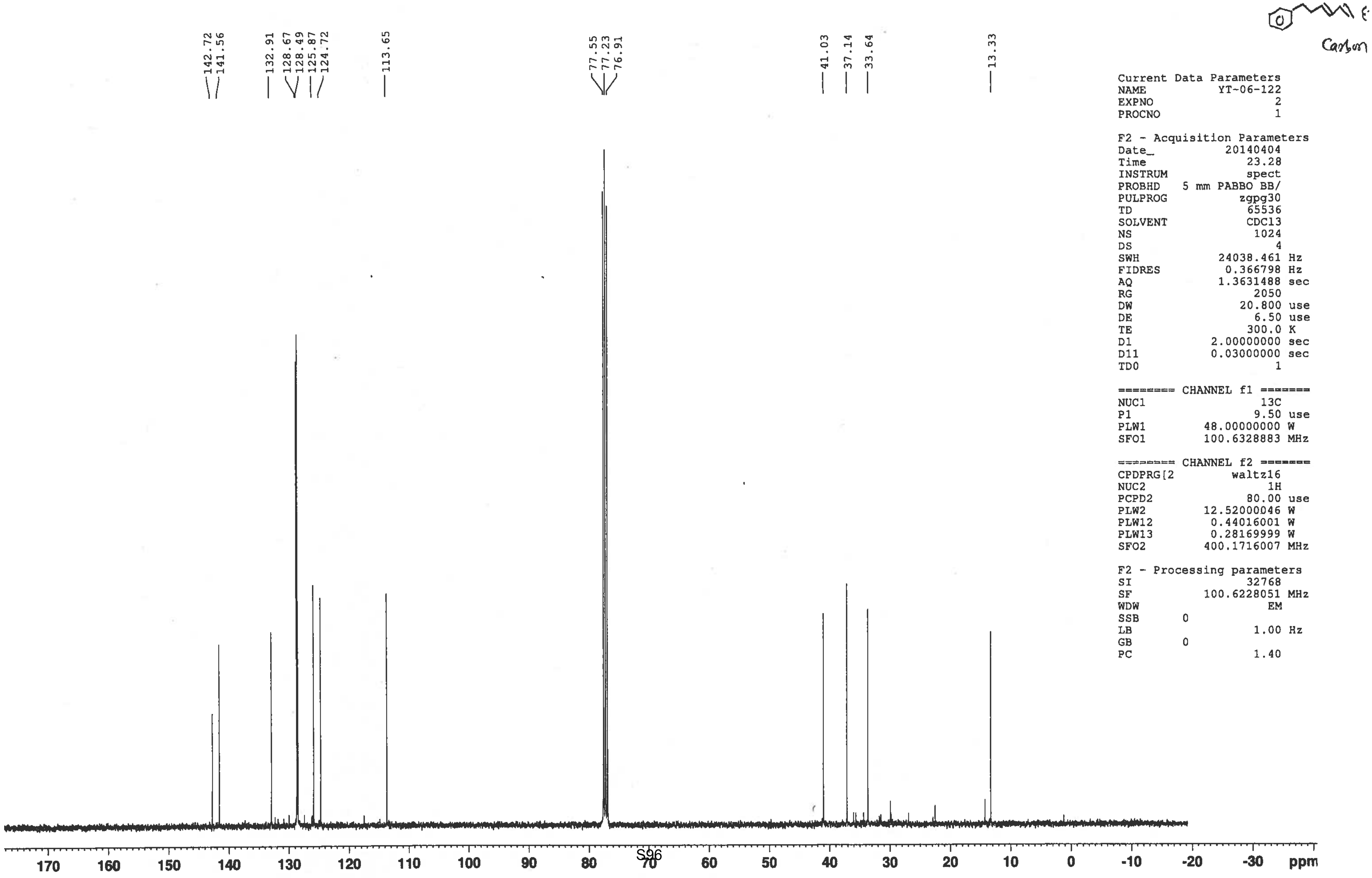

Hydrovinylation of (*E*)-4-(buta-1,3-dienyl)-*N*,*N*-dimethylaniline (12k)

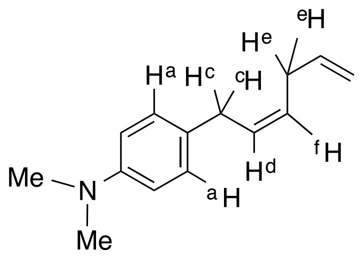

Hydrovinylation of (*E*)-4-(buta-1,3-dienyl)-*N*,*N*-dimethylaniline using DPPBCoCl$_2$ (Table 3, Entry 17): To an oven dried round bottom flask with a sidearm, was added [DPPB]CoCl$_2$ (36 mg, 0.065 mmol) and methylaluminoxane (76 mg, 1.30 mmol) under argon. The color of the solution changed from deep blue to red brown with the formation of white fumes over the addition of dichloromethane solvent (1 mL). After 2 to 3 minutes, the reaction vessel was carefully evacuated and then refilled with ethylene balloon. The reaction vessel was kept at 0 ^{0}C and (*E*)-4-(buta-1,3-dienyl)-*N,N*-dimethylaniline (113 mg, 0.65 mmol) in dichloromethane (1 mL) added via syringe under ethylene and the mixture was stirred for 13 h at ambient temperature. The ethylene balloon was removed and 0.1 mL methanol was added into the flask. The reaction solution was diluted with pentane (2 mL) and diethyl ether (2 mL) and warmed to room temperature. After this, the reaction solution was subsequently passed through a silica plug. The plug was washed with diethyl ether (3 X 5 mL). Flash column chromatography with (5:1, hexane: ethyl acetate) yielded the product as yellowish oil (104 mg, 79%). GC and NMR analysis showed that the product (**16k**) was essentially pure. ^{1}H NMR (CDCl$_3$, 400 MHz): 2.91 (s, 6 H), 2.91 - 2.95 (overlap with 2.91) (m, 2 H), 3.32 (d, J = 7 Hz, 2 H), 5.01 (dq, J = 10, 1.6 Hz, 1 H), 5.09 (dq, J = 17, 1.6 Hz, 1 H), 5.47 - 5.54 (m, 1 H), 5.59 - 5.67 (m, 1 H), 5.86 (ddt, J = 17, 10, 6 Hz, 1 H), 6.72 (d, J = 8 Hz, 2 H, aromatic), 7.07 (d, J = 9 Hz, 2 H, aromatic).

^{13}C NMR (CDCl$_3$, 125.02 MHz): 31.71, 32.60, 41.25, 113.52, 114.99, 127.14, 129.15, 130.32, 137.13.

Gas Chromatography: Methylsilicone SP GC (OT 150 ^{o}C/ isothermal, He carrier gas): R_T for product = 23.02 min. R_T for starting material = 18.68 min. ESI-MS; m/z 202.1593 [M + H]; mass calculated for $C_{14}H_{20}N_1$, 202.1590.

* RUN # 165 MAR 1, 1901 00:09:44

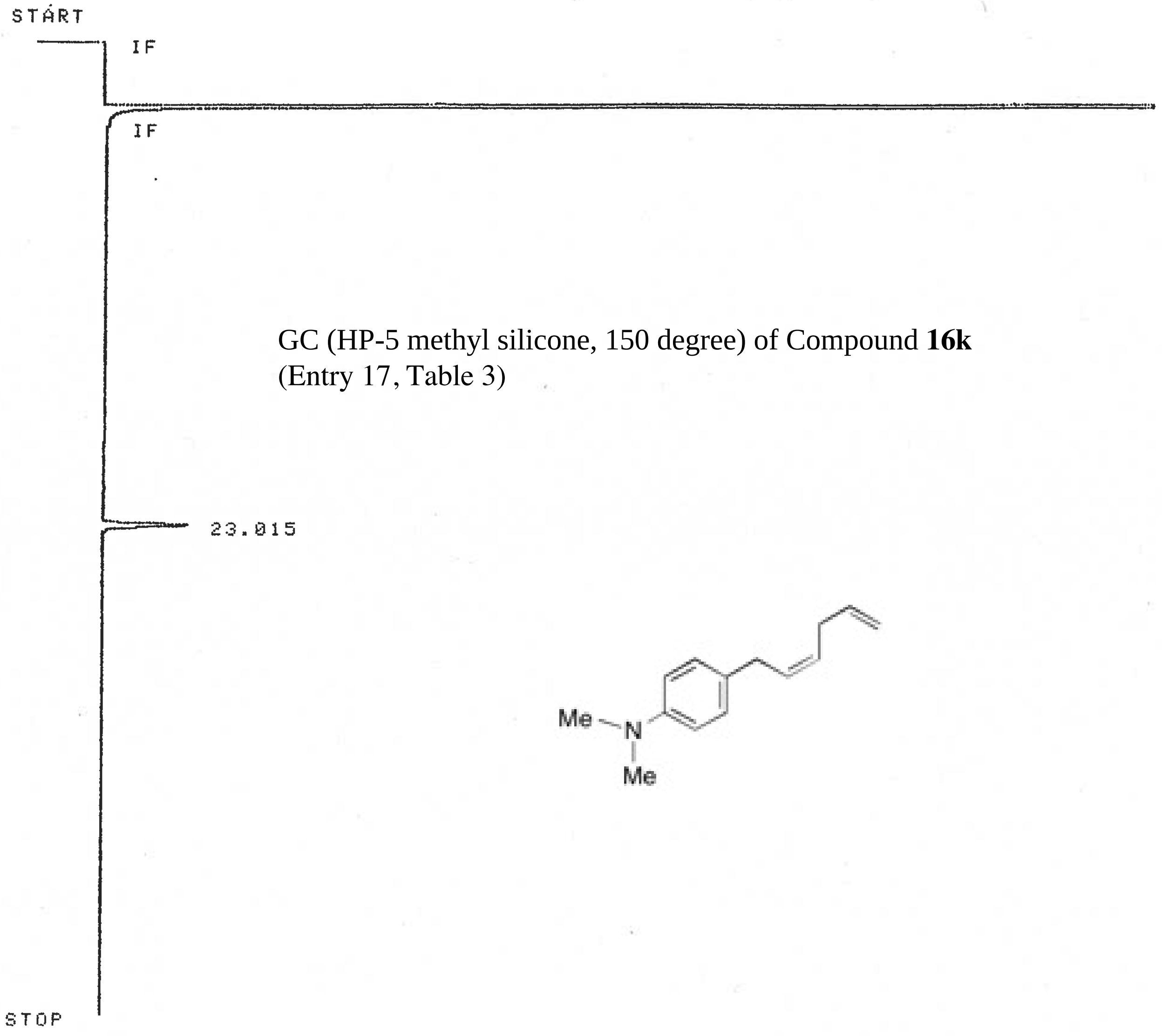

GC (HP-5 methyl silicone, 150 degree) of Compound **16k**
(Entry 17, Table 3)

RUN# 165 MAR 1, 1901 00:09:44

AREA%

RT	AREA	TYPE	WIDTH	AREA%
23.015	258275	BP	.205	100.00000

TOTAL AREA= 258275
MUL FACTOR=1.0000E+00

```
Current Data Parameters
NAME     yt-04-178re12pure
EXPNO                   1
PROCNO                  1

F2 - Acquisition Parameters
Date_            20130108
Time                11.08
INSTRUM             spect
PROBHD   5 mm BBI 1H-BB
PULPROG              zg30
TD                  65536
SOLVENT             CDCl3
NS                     16
DS                      2
SWH              8250.825 Hz
FIDRES           0.125898 Hz
AQ              3.9715316 sec
RG                   45.3
DW                 60.600 usec
DE                   6.00 usec
TE                  300.2 K
D1             1.00000000 sec
TD0                     1

======== CHANNEL f1 ========
NUC1                   1H
P1                   8.00 usec
PL1                 -2.65 dB
SFO1          400.1324710 MHz

F2 - Processing parameters
SI                  32768
SF            400.1300174 MHz
WDW                    EM
SSB      0
LB                   0.30 Hz
GB       0
PC                   1.00
```

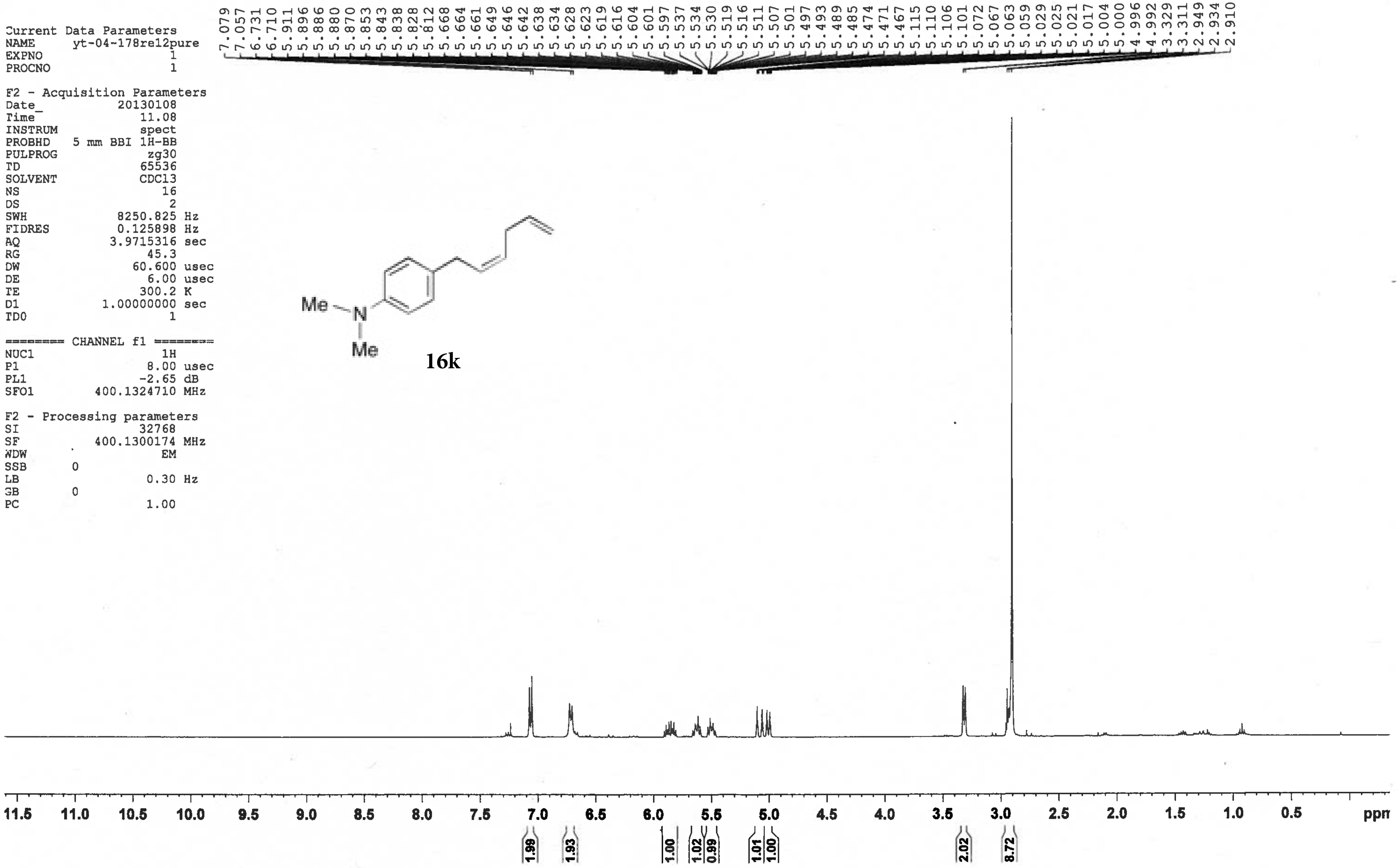

Proton NMR (400 MHz, D-Chloroform) Spectra of Compound **16k** (Entry 17, Table 3)

Proton NMR (400 MHz, D-chloroform) spectrum of compound **16k**

```
Current Data Parameters
NAME     yt-04-178re12pure
EXPNO                   1
PROCNO                  1

F2 - Acquisition Parameters
Date_            20130108
Time                11.08
INSTRUM             spect
PROBHD   5 mm BBI 1H-BB
PULPROG              zg30
TD                  65536
SOLVENT             CDCl3
NS                     16
DS                      2
SWH              8250.825 Hz
FIDRES           0.125898 Hz
AQ              3.9715316 sec
RG                   45.3
DW                 60.600 usec
DE                   6.00 usec
TE                  300.2 K
D1             1.00000000 sec
TD0                     1

======== CHANNEL f1 ========
NUC1                   1H
P1                   8.00 usec
PL1                 -2.65 dB
SFO1          400.1324710 MHz

F2 - Processing parameters
SI                  32768
SF            400.1300174 MHz
WDW                    EM
SSB      0
LB                   0.30 Hz
GB       0
PC                   1.00
```

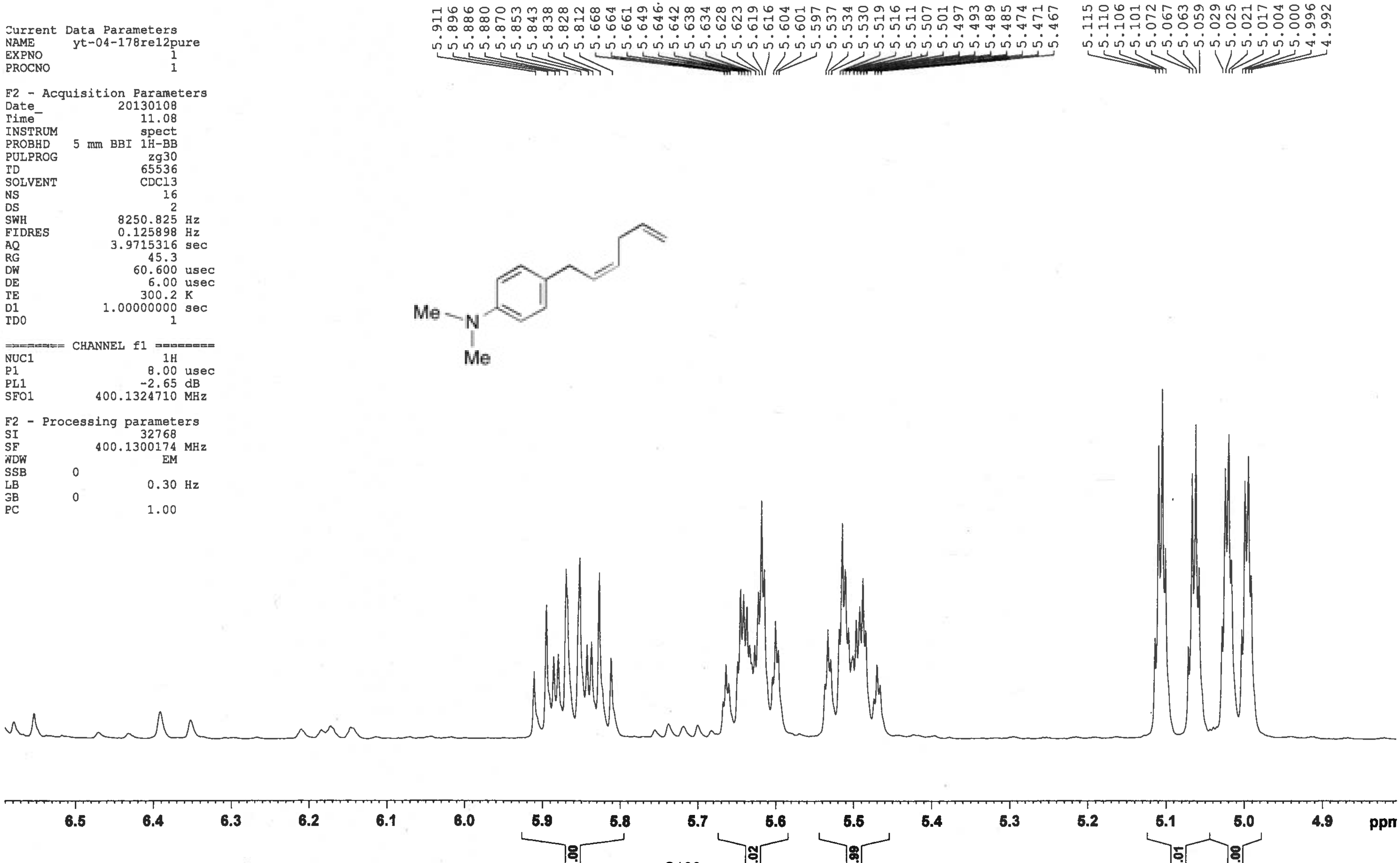

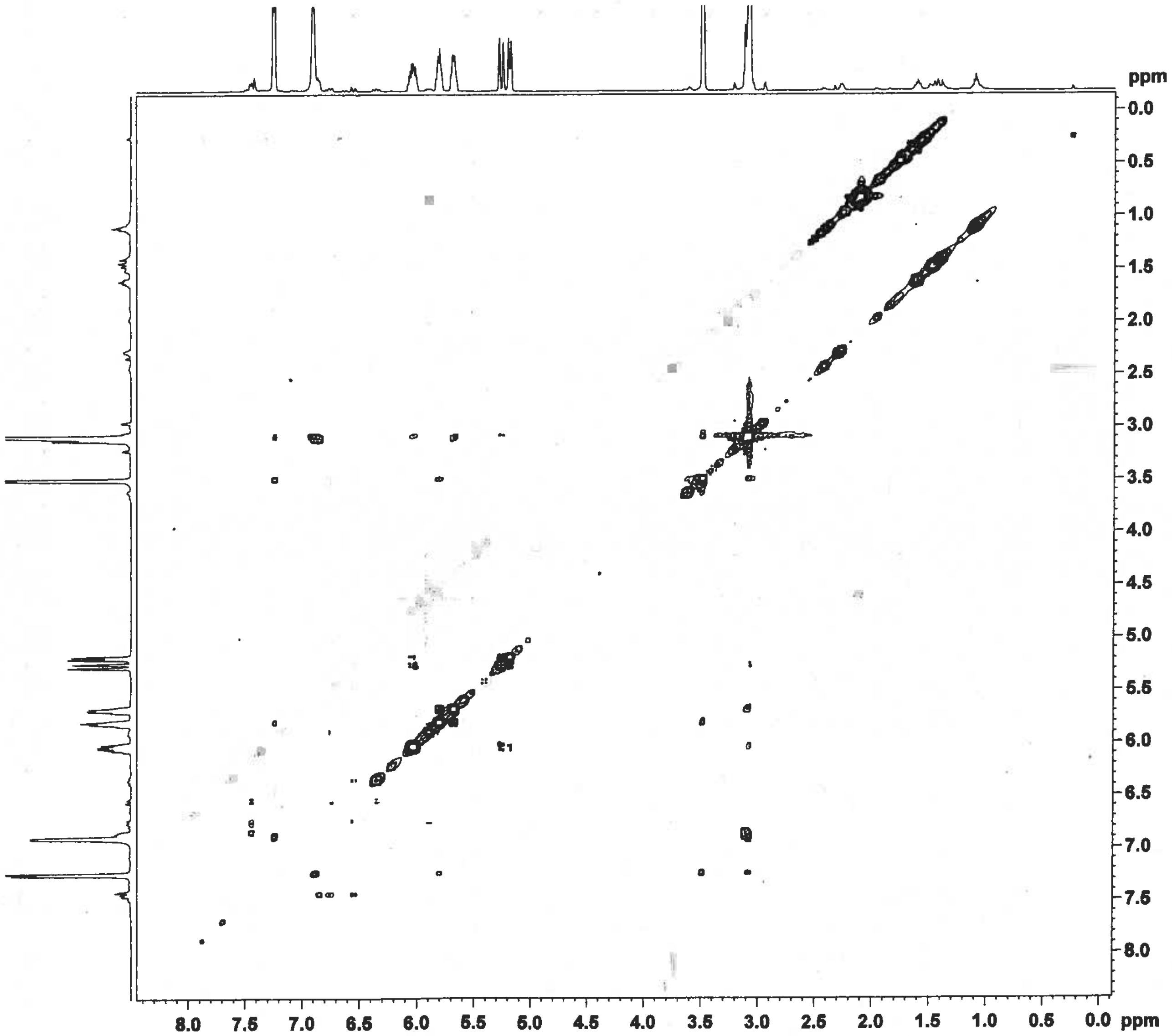

NOESY (500 MHz, D-chloroform) 2D
spectrum of compound **16k**

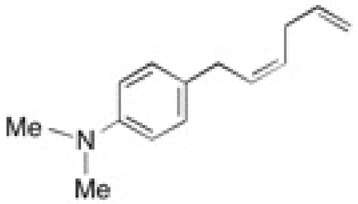

Carbon NMR (125 MHz, D-Chloroform) Spectra of Compound **16k**

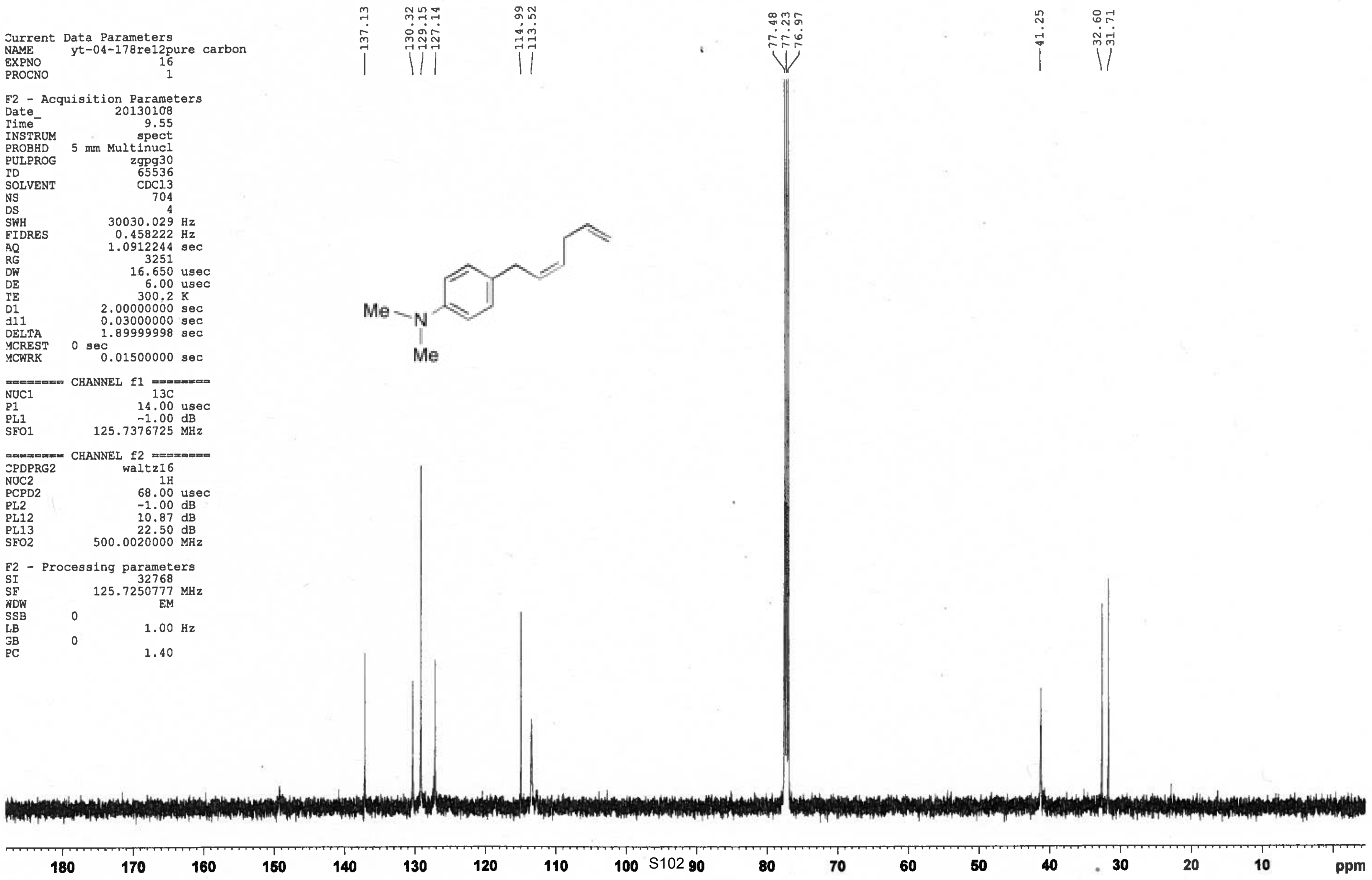

Hydrovinylation of (*E*)-1-phenyl-2-methyl-1,3-butadiene (12l)

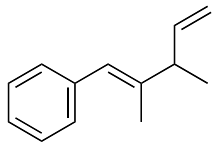

Hydrovinylation of (*E*)-1-phenyl-2-methyl-1,3-butadiene using [dppm]$CoCl_2$ (Table 3, Entry 18 and 19). To an oven-dried 10 mL round-bottom flask with a sidearm, was added [dppm]$CoCl_2$ (35.6 mg, 0.0693 mmol) under argon and it was dissolved in a mixture of degassed dichloromethane (4.0 mL) and toluene (1 mL), at 0 ^{o}C. Trimethylaluminum solution (2M) in toluene (15.0 mg, 104 μl, 0.208 mmol) was added dropwise as color of the solution changed from green to brown with the formation of white fumes over the solution. When all the fumes disappeared, the reaction vessel was carefully evacuated and then refilled with ethylene from a balloon. The filled balloon was used to maintain the ethylene atmosphere, while a vigorous reaction with evolution of fumes was observed. This evolution stopped in typically within 5 min. The reaction vessel was cooled to -10 ^{o}C and (*E*)-2-methyl-1-phenyl-1,3-butadiene (100.0 mg, 0.6935 mmol) was added under ethylene and the mixture was stirred for 6 h (color of the reaction solution turned dark green again at the end of the reaction). The ethylene balloon was removed and 0.2 mL of methanol was introduced into the flask and stirring was continued for 5 minutes. The solution was warmed to room temperature and was subsequently passed through a silica plug. The plug was washed with hexane (3 X 20 mL). Concentration and removal of last traces of solvent yielded the product as a colorless oil (**15l**, 116.9 mg; 97 %). Isomeric compositions were determined by gas chromatography and NMR spectroscopy. ^{1}H NMR ($CDCl_3$, 500 MHz): 1.184 (d, 7 Hz, 3 H), 1.816 (dd, 1.5, 1.5, 3 H), 2.922 (m, 1 H), 5.044 (dm, 17 Hz, 1 H), 5.009 (dm, 12 Hz, 1 H), 6.295 (s, 1H), 5.842 (ddd, 17, 11, 1.5), 6.295 (s, 1 H), 7.10-7.30 (m, aromatic). NOESY: PhC*H* —> C_{sp3}-CH_3, bis-allylic C*H*. ^{13}C NMR ($CDCl_3$): 15.72, 18.16, 46.88, 113.71, 124.61, 125.98, 128.05, 128.98, 138.61, 141.84, 142.31.

Since the HV product itself could not be separated on Cyclodex-B column, further derivatization was employed to prepare an alcohol, which was analyzed as the TMS ether. The hydrocarbon **15l** was subjected to hydroboration/oxidation using 9-BBN and the enantiomeric mixture of alcohols were converted into the corresponding –TMS ethers which were analyzed on Cyclodex-B column. The product was ascertained to be nearly racemic. Optical rotation measurements further confirmed these observations.

* RUN # 589 APR 18, 1901 10:52:03

START

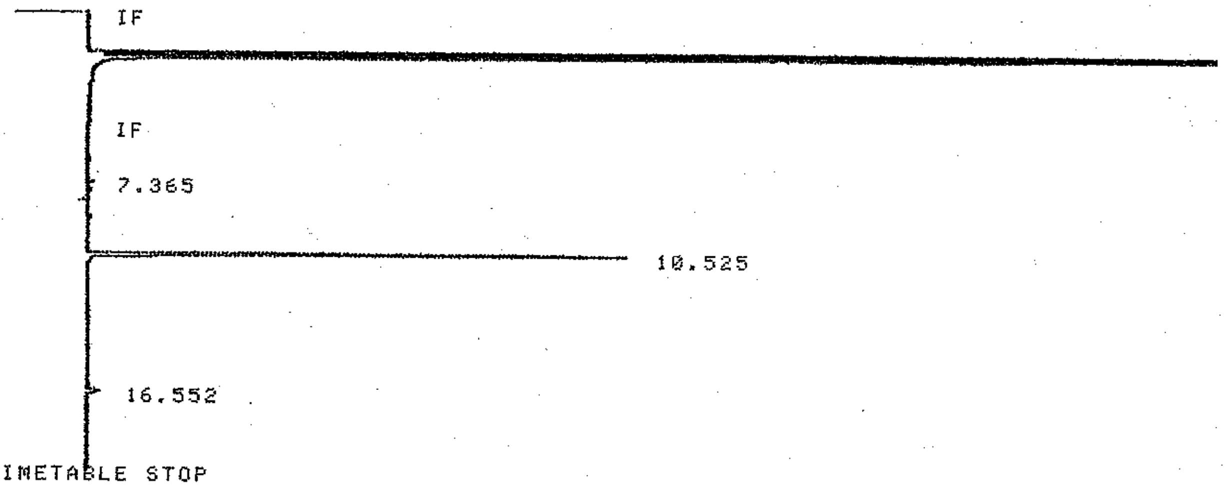

TIMETABLE STOP

RUN# 589 APR 18, 1901 10:52:03

AREA%

RT	AREA	TYPE	WIDTH	AREA%
10.525	305659	PV	.082	96.16272
16.552	12197	PV	.131	3.83727

TOTAL AREA= 317856
MUL FACTOR=1.0000E+00

(Entry 18, Table 3)

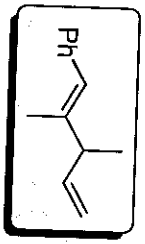

150°C
(Polydimethylsiloxane)

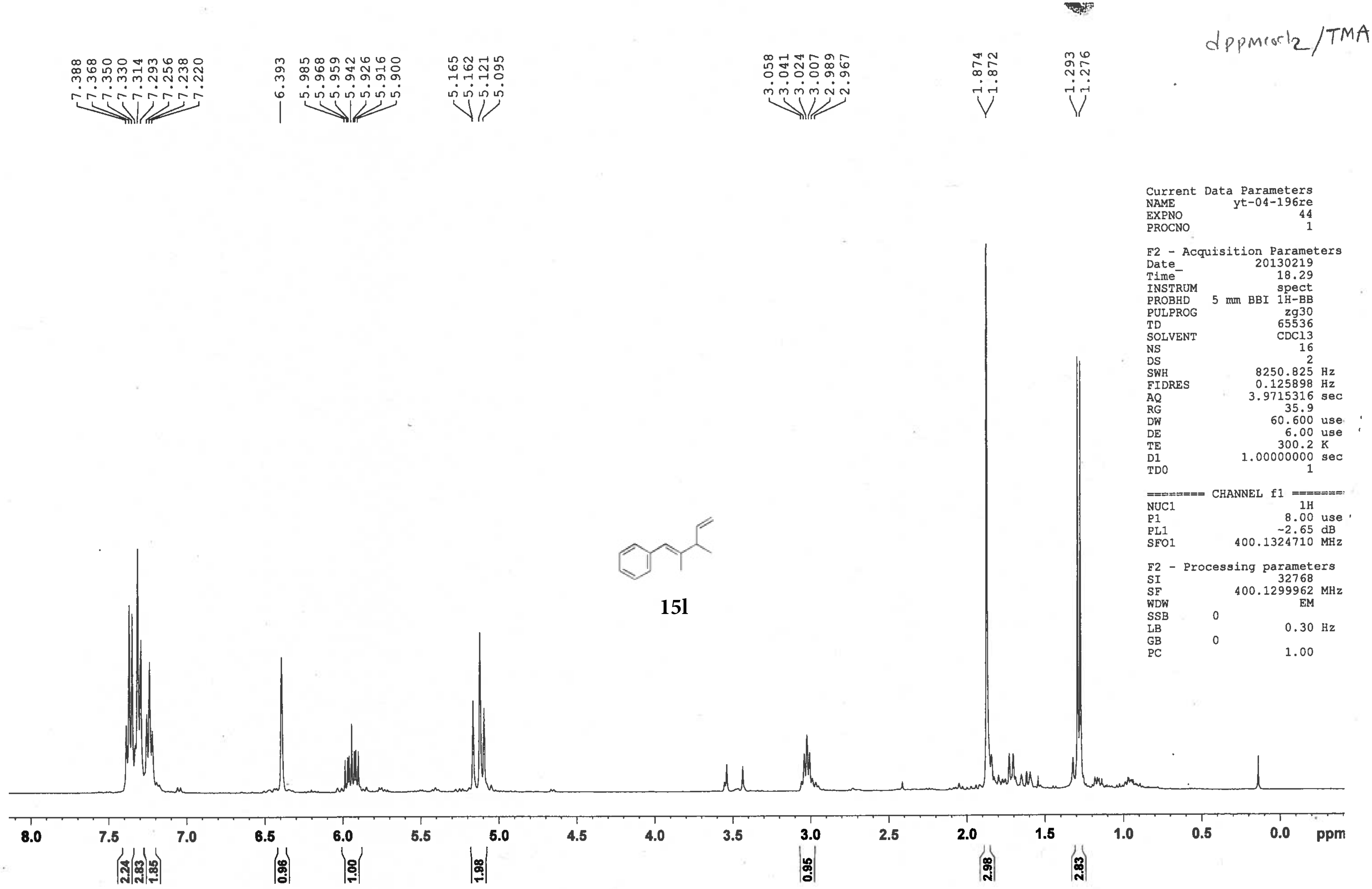

Proton NMR (400 MHz, D-chloroform) Spectra of compound **15l** (HV using Cl2Co(dppp) (Entry 19, Table 3)

Proton NMR (500 MHz, D-chloroform) Spectra of compound **15l** (Alkene Region)

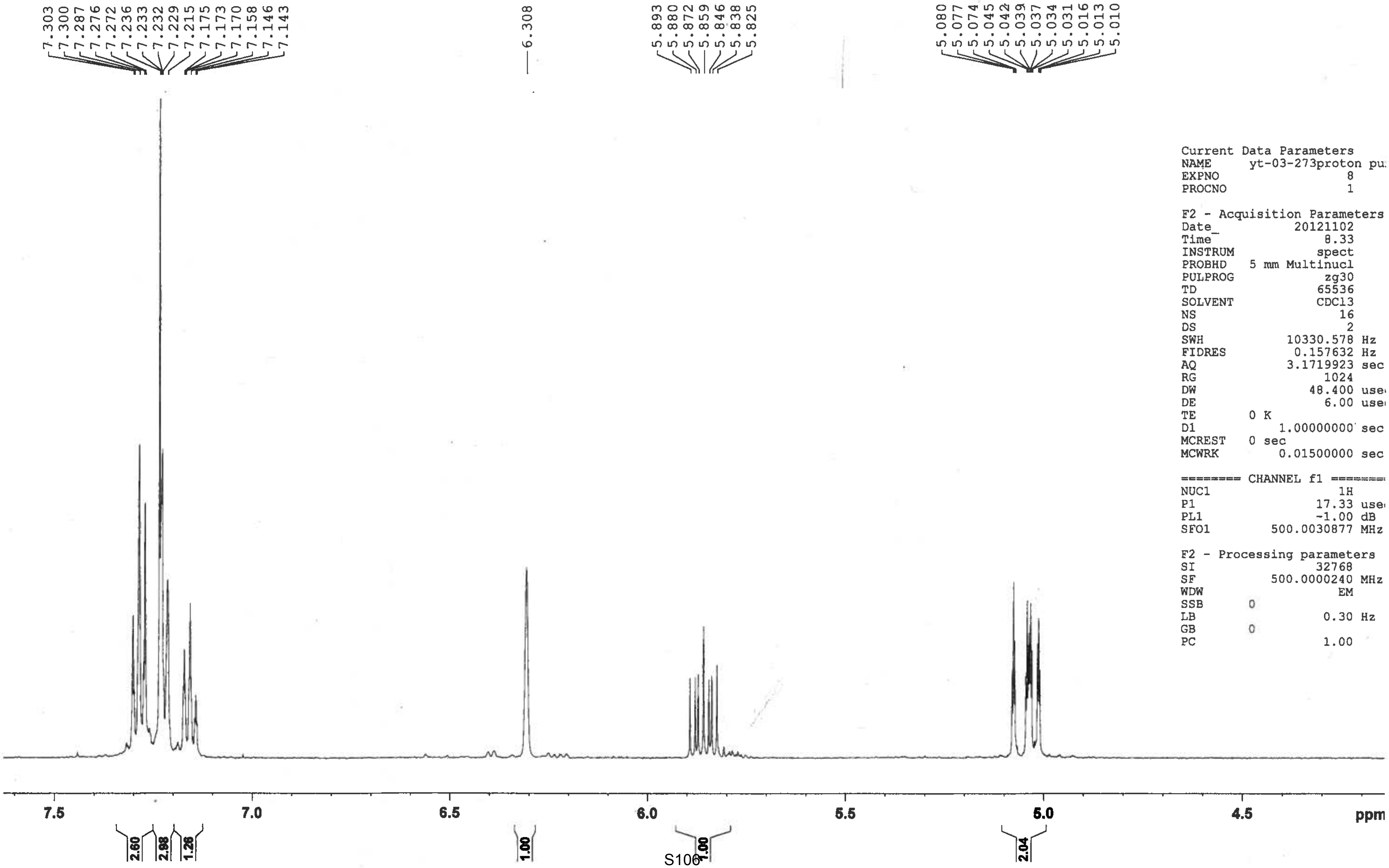

Carbon NMR (125 MHz, D-Chloroform) of compound **15l** (Entry 19, Table 3)

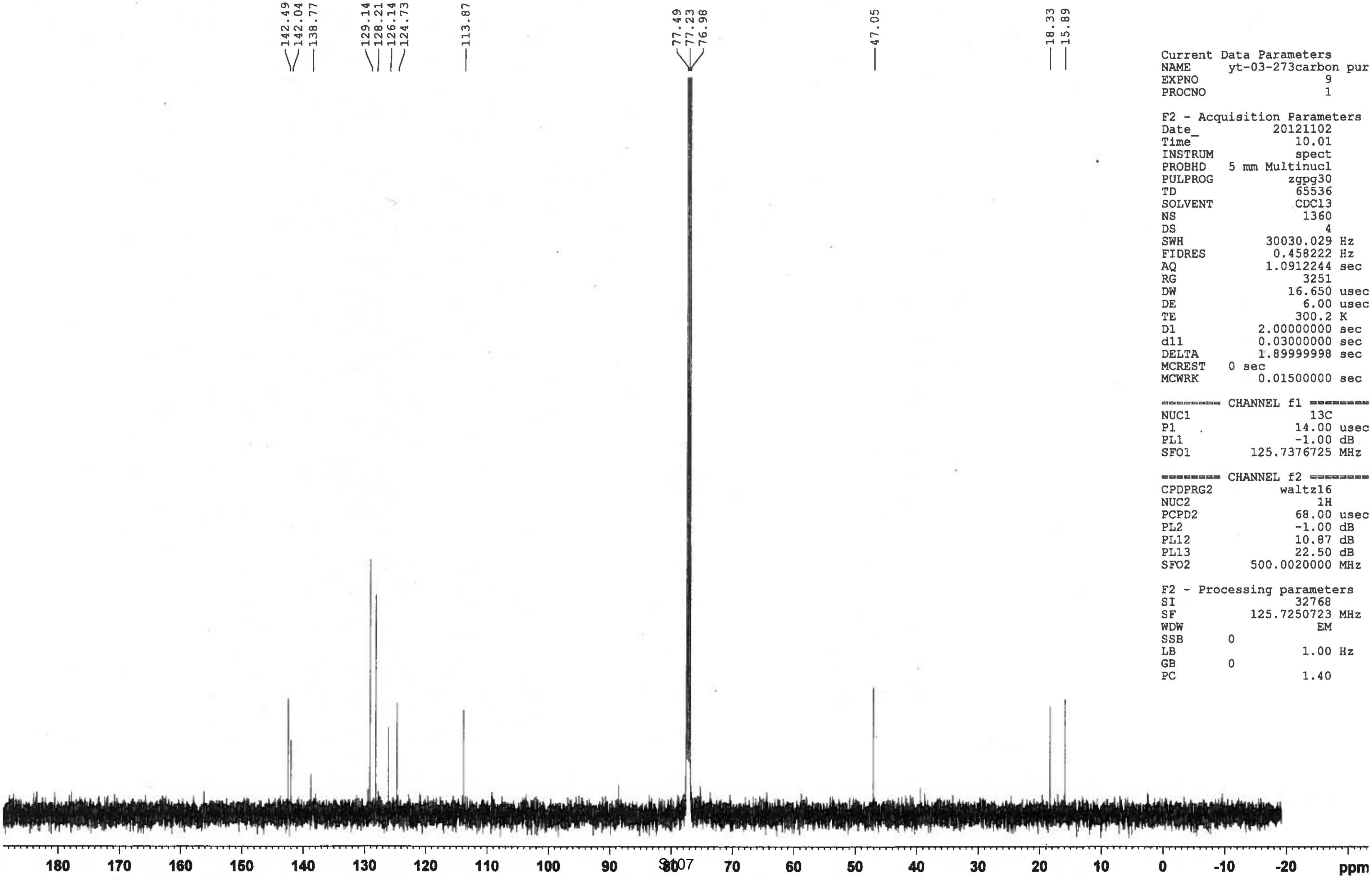

Hydrovinylation of 7-methyl-3-methyleneocta-1,6-diene (β-myrcene, 12m)

Hydrovinylation of (β-myrcene) using [dppp]$CoCl_2$ (Table 3, Entry 21). To an oven dried round bottom flask with a sidearm, was added [DPPP]$CoCl_2$ (49 mg, 0.090 mmol) dissolved in dichloromethane (1 mL) at room temperature. Trimethylaluminium as a 2M solution in toluene (0.14 mL, 0.27 mmol) was added dropwise and the color of the solution changed from deep blue to red brown with the formation of white fumes over the solution. After 2 to 3 minutes, the reaction vessel was carefully evacuated and then refilled with ethylene from a balloon. The reaction vessel was kept at room temperature and 7-methyl-3-methyleneocta-1,6-diene (0.15 mL, 0.90 mmol) added under ethylene and the mixture was stirred for 1 h. The ethylene balloon was removed and 0.1 mL of methanol was added to the flask. The reaction solution was diluted with pentane (2 mL) and subsequently passed through a silica plug. The plug was washed with pentane (3 x 5 mL). Removal of solvent yielded the product as colorless oil (123 mg, 83%). GC and NMR analysis showed that the product (**16m**) was essentially pure. ^{1}H NMR ($CDCl_3$, 400MHz): 1.57 – 1.58 (m, 6 H), 1.66 (s, 3 H), 1.95 – 2.07 (m, 4 H), 2.77 (d, J = 6.4 Hz, 2 H), 4.94 - 5.04 (m, 2 H), 5.06 - 5.09 (m, 1 H), 5.28 (q, J = 7 Hz, 1 H), 5.68 - 5.78 (m, 1 H). NOESY: H_g ---> H_h, H_i; H_f ---> H_e, H_d; H_e ---> H_f, H_c; H_d ---> H_f, H_c. ^{13}C NMR ($CDCl_3$, 125.02 MHz): 13.43, 17.90, 25.92, 26.99, 34.81, 37.29, 115.11, 119.85, 124.59, 131.60, 136.38, 137.76. Gas Chromatography: CSP GC (Cyclosil, OT 100 °C/ isothermal, H_2 carrier gas) starting material R_t = 6.571 min. R_T for product = 9.954 min. The following table shows the additional experiments conducted by using β-myrcene employing 10 mol % catalyst and trimethylaluminium as an activator. The isomeric product has not been identified.

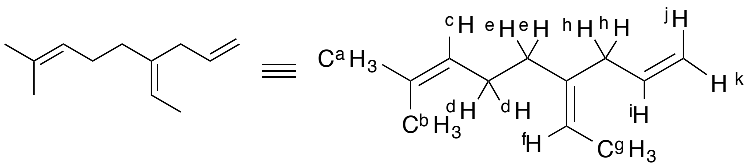

Entry	Catalyst	Temp(°C)/Time(h)	Conv.(%)	Product
1.	[DPPM]$CoCl_2$	rt/4	71%	(55%) isomer(3%)
2.	[DPPE]$CoCl_2$	-20/3	8%	8%
3.	[DPPB]$CoCl_2$	-20/4	91%	(82%)+ iso(9%)
4.	(*SS*)-[DIOP]$CoCl_2$	-20/7	91%	(62%) +iso(15%)
5.	(*SS*)-[BDPP]$CoCl_2$	-20/7	94%	(36%)+ iso(57%)

Data File C:\CHEM32\1\DATA\YAM\YT-04-194MYRO.D
Sample Name: YT-04-194MYRO

dppp/-20°c/4hrs.

===

Acq. Operator : YAM
Acq. Instrument : Babu Location : Vial 201
Injection Date : 11/28/2012 3:59:20 PM
Inj Volume : 1 µl
Different Inj Volume from Sequence ! Actual Inj Volume : 3 µl
Acq. Method : C:\CHEM32\1\METHODS\YT_ISO120_CYCLODEX-B.M
Last changed : 11/28/2012 3:41:27 PM by YAM
(modified after loading)
Analysis Method : C:\CHEM32\1\METHODS\BAKE.M
Last changed : 11/28/2012 4:43:57 PM by Kendra Dewese
(modified after loading)

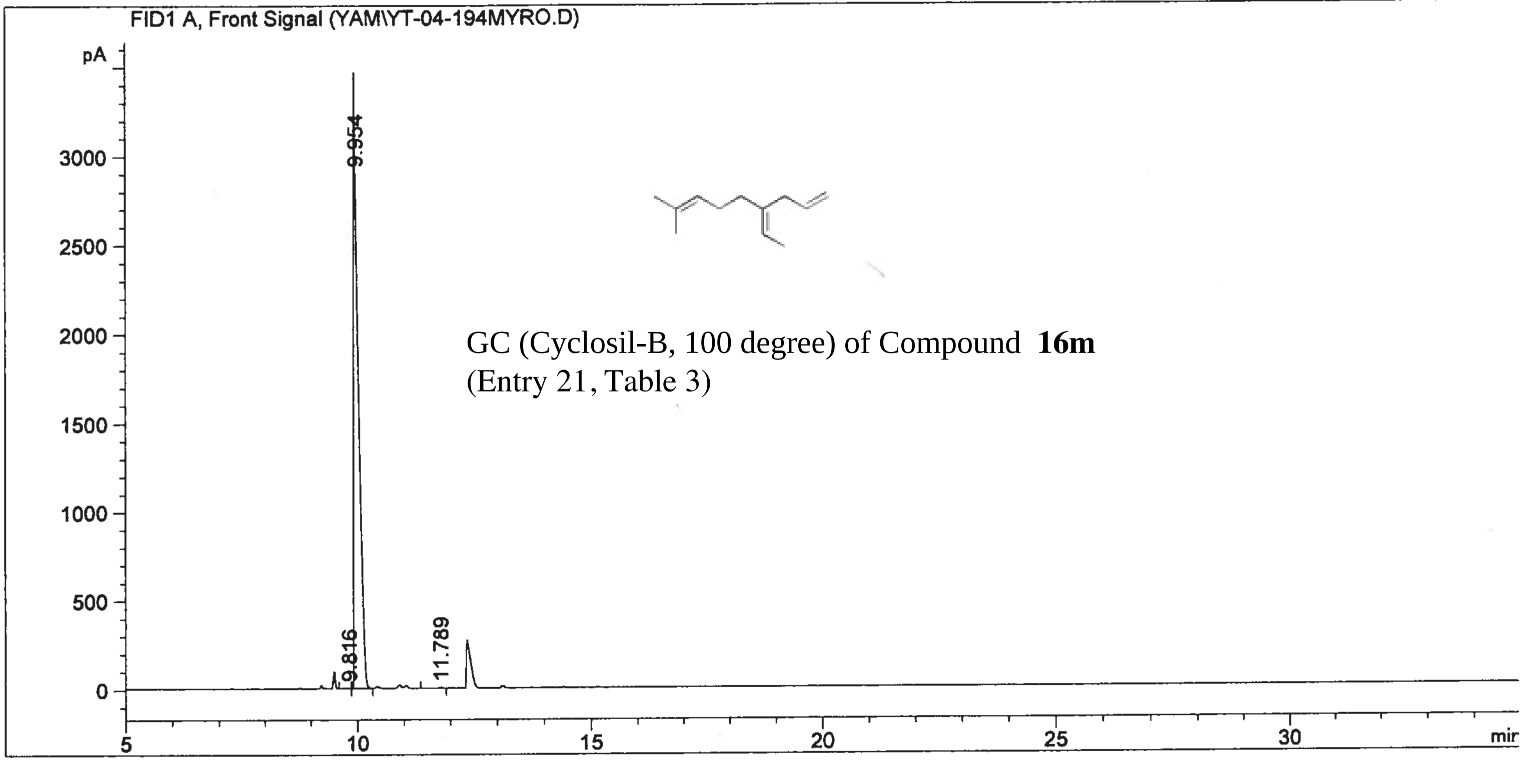

GC (Cyclosil-B, 100 degree) of Compound **16m**
(Entry 21, Table 3)

===

Area Percent Report

===

Sorted By : Signal
Multiplier: : 1.0000
Dilution: : 1.0000
Use Multiplier & Dilution Factor with ISTDs

Signal 1: FID1 A, Front Signal

Peak #	RetTime [min]	Type	Width [min]	Area [pA*s]	Height [pA]	Area %
1	9.816	VV	0.0948	37.49633	5.54818	0.15672
2	9.954	VV	0.1119	2.38449e4	2900.67920	99.66528
3	11.789	BV	0.1006	42.58645	6.35175	0.17800

Totals : 2.39250e4 2912.57913

Proton NMR (400 MHz, D-chloroform) spectra of compound **16m**
(Entry 21, Table 3)

```
Current Data Parameters
NAME     yt-04-193at rt6,7pro
EXPNO                  73
PROCNO                  1

F2 - Acquisition Parameters
Date_            20121227
Time                16.08
INSTRUM             spect
PROBHD   5 mm BBI 1H-BB
PULPROG              zg30
TD                  65536
SOLVENT             CDCl3
NS                     16
DS                      2
SWH             8250.825 Hz
FIDRES          0.125898 Hz
AQ             3.9715316 sec
RG                  101.6
DW                 60.600 usec
DE                   6.00 usec
TE                  300.2 K
D1             1.00000000 sec
TD0                     1

======== CHANNEL f1 ========
NUC1                   1H
P1                   8.00 usec
PL1                 -2.65 dB
SFO1          400.1324710 MHz

F2 - Processing parameters
SI                  32768
SF            400.1300175 MHz
WDW                    EM
SSB      0
LB                   0.30 Hz
GB       0
PC                   1.00
```

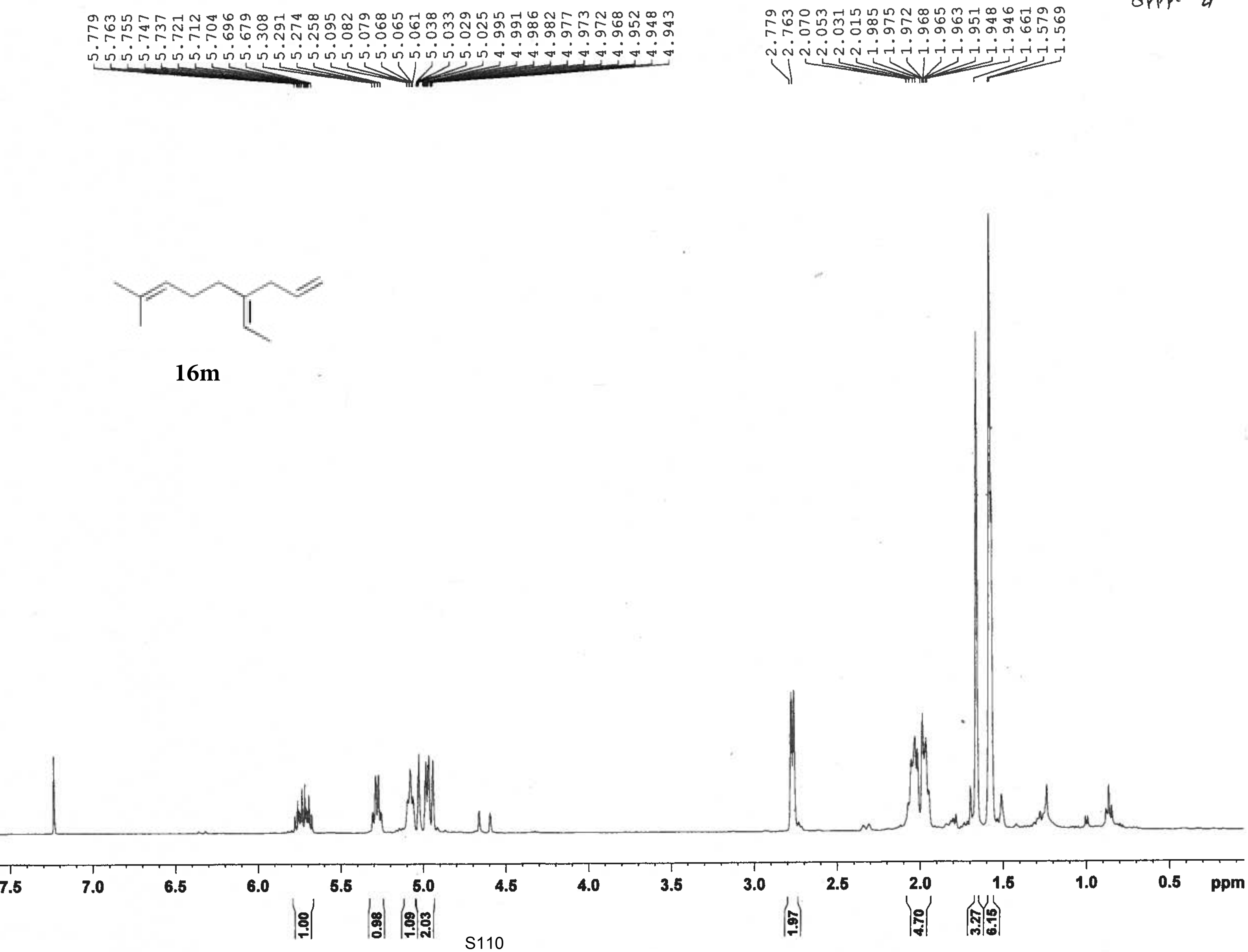

16m

dpppcacl2/ TMA/ β-[illegible]

Proton NMR (400 MHz, D-chloroform) spectra of compound **16m** (Expansion)

```
Current Data Parameters
NAME     yt-04-193at rt6,7pro
EXPNO                 73
PROCNO                 1

F2 - Acquisition Parameters
Date_           20121227
Time               16.08
INSTRUM            spect
PROBHD   5 mm BBI 1H-BB
PULPROG             zg30
TD                 65536
SOLVENT            CDCl3
NS                    16
DS                     2
SWH             8250.825 Hz
FIDRES          0.125898 Hz
AQ             3.9715316 sec
RG                 101.6
DW                60.600 usec
DE                  6.00 usec
TE                 300.2 K
D1            1.00000000 sec
TD0                    1

======== CHANNEL f1 ========
NUC1                  1H
P1                  8.00 usec
PL1                -2.65 dB
SFO1         400.1324710 MHz

F2 - Processing parameters
SI                 32768
SF           400.1300175 MHz
WDW                   EM
SSB      0
LB                  0.30 Hz
GB       0
PC                  1.00
```

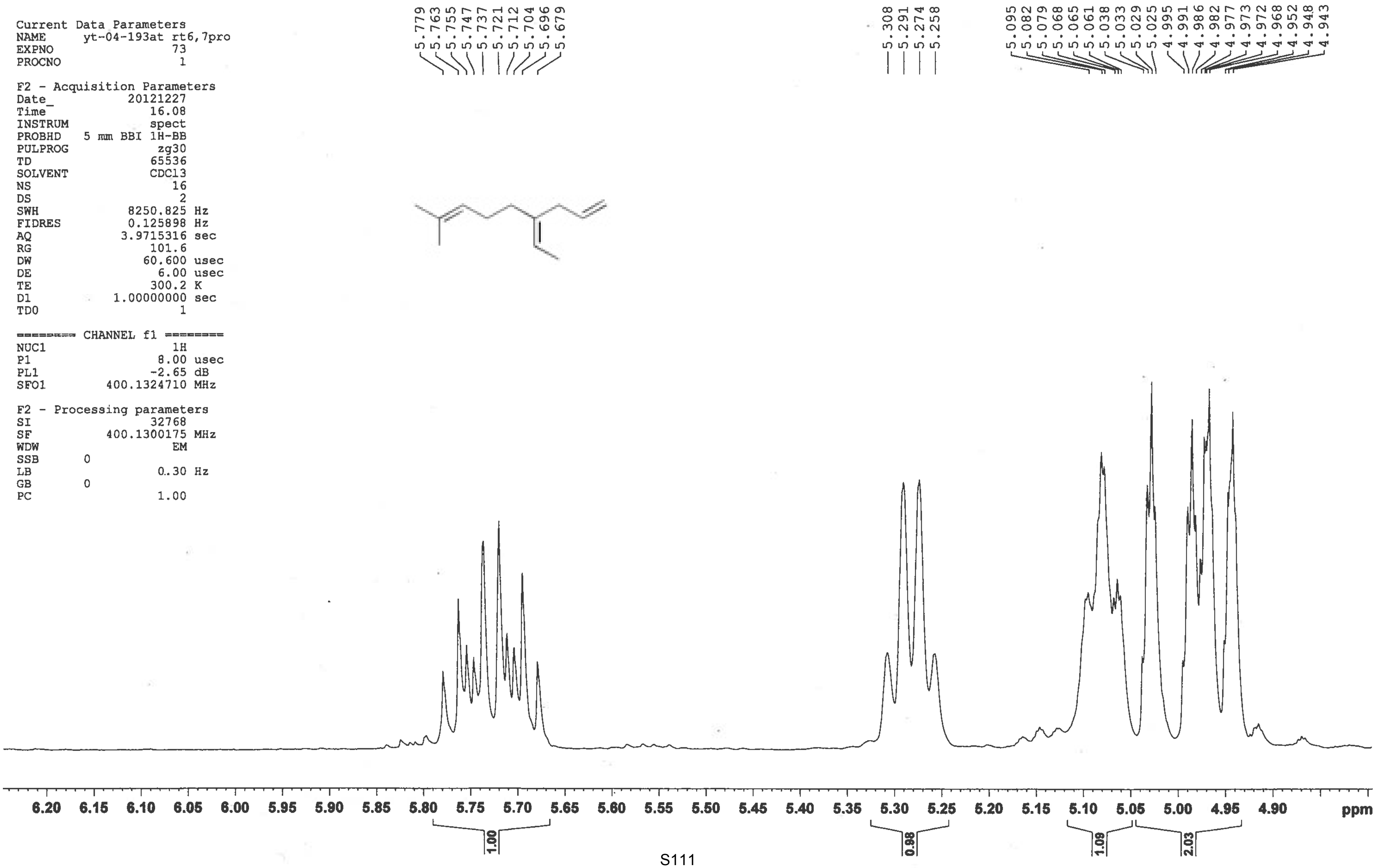

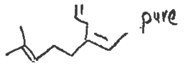

Carbon NMR (125 MHz, D-chloroform) spectra of compound **16m**

```
Current Data Parameters
NAME     yt-04-193rt1car
EXPNO                 14
PROCNO                 1

F2 - Acquisition Parameters
Date_          20121227
Time              12.56
INSTRUM           spect
PROBHD   5 mm Multinucl
PULPROG         zgpg30
TD               65536
SOLVENT          CDCl3
NS                 871
DS                   4
SWH          30030.029 Hz
FIDRES        0.458222 Hz
AQ           1.0912244 sec
RG              3649.1
DW              16.650 usec
DE                6.00 usec
TE               295.2 K
D1          2.00000000 sec
d11         0.03000000 sec
DELTA       1.89999998 sec
MCREST   0 sec
MCWRK       0.01500000 sec

======== CHANNEL f1 ========
NUC1               13C
P1               14.00 usec
PL1              -1.00 dB
SFO1       125.7376725 MHz

======== CHANNEL f2 ========
CPDPRG2        waltz16
NUC2                1H
PCPD2            68.00 usec
PL2              -1.00 dB
PL12             10.87 dB
PL13             22.50 dB
SFO2       500.0020000 MHz

F2 - Processing parameters
SI               32768
SF         125.7250747 MHz
WDW                 EM
SSB      0
LB                1.00 Hz
GB       0
PC                1.40
```

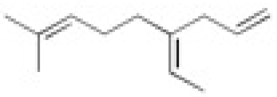

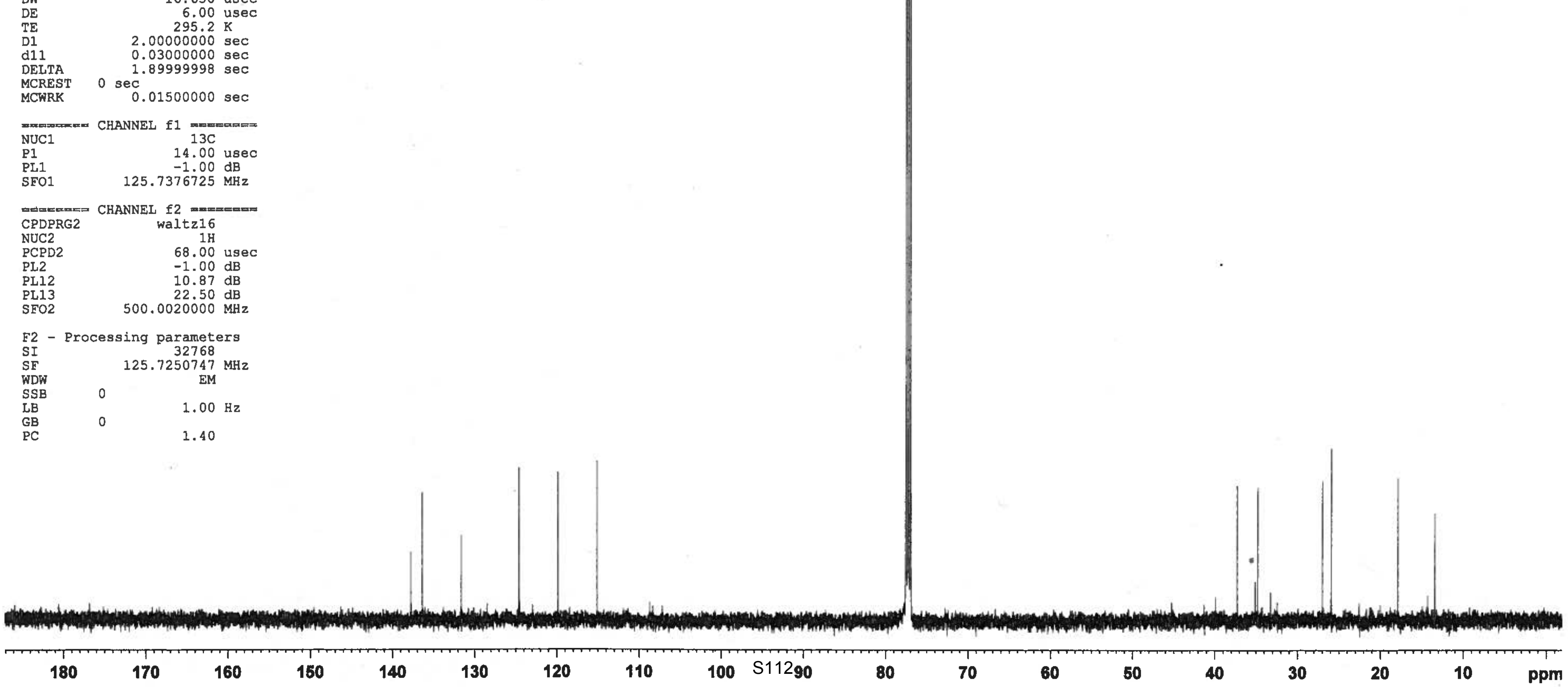

NOESY (500 MHz, D-chloroform) 2D spectra of compound **16m**

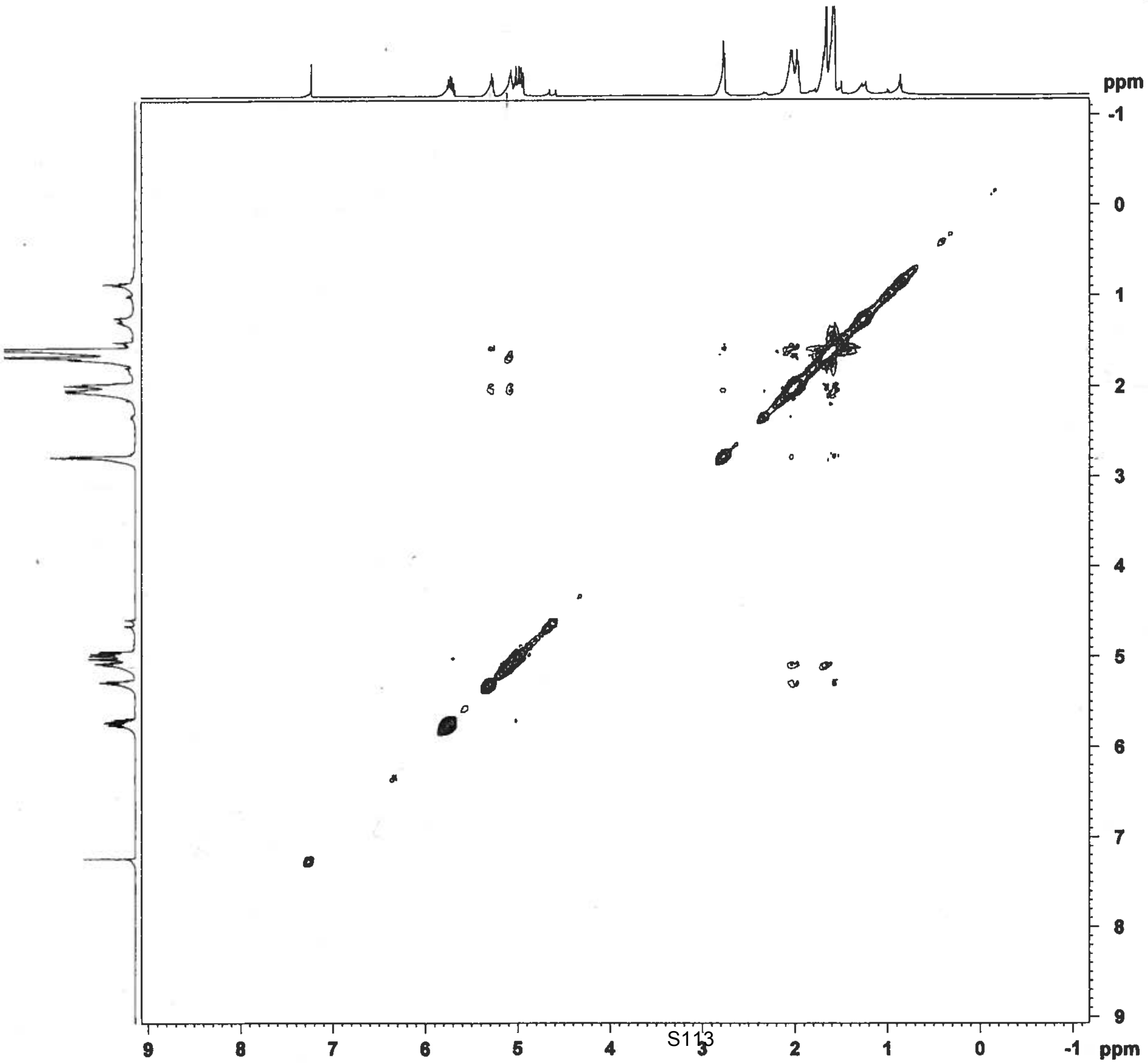


```
Current Data Parameters
NAME        yt-04-193,67noe
EXPNO                     2
PROCNO                    1

F2 - Acquisition Parameters
Date_              20130109
Time                  14.46
INSTRUM               spect
PROBHD    5 mm Multinucl
PULPROG           noesygpph
TD                     2048
SOLVENT               CDCl3
NS                        4
DS                       16
SWH              5122.951 Hz
FIDRES           2.501441 Hz
AQ              0.1999348 sec
RG                      256
DW                   97.600 usec
DE                     6.00 usec
TE                    300.2 K
d0             0.00007553 sec
D1             2.00000000 sec
D8             2.00000000 sec
D16            0.00020000 sec
IN0            0.00019520 sec
MCREST    0 sec
MCWRK          2.00000000 sec
TAU            0.99879998 sec

========== CHANNEL f1 ==========
NUC1                     1H
P1                    17.33 usec
P2                    34.66 usec
PL1                   -1.00 dB
SFO1            500.0020000 MHz

====== GRADIENT CHANNEL =====
GPNAM1             SINE.100
GPNAM2             SINE.100
GPX1      0 %
GPX2      0 %
GPY1      0 %
GPY2      0 %
GPZ1                  40.00 %
GPZ2                 -40.00 %
P16                 1000.00 usec

F1 - Acquisition parameters
TD                      248
SFO1                500.002 MHz
FIDRES            20.657061 Hz
SW                   10.246 ppm
FnMODE                 TPPI

F2 - Processing parameters
SI                     1024
SF              500.0000259 MHz
WDW                   QSINE
SSB                       2
LB        0 Hz
GB        0
PC                     1.00

F1 - Processing parameters
SI                     1024
MC2                    TPPI
SF              500.0000259 MHz
WDW                      TR
SSB                       2
LB        0 Hz
GB        0
```

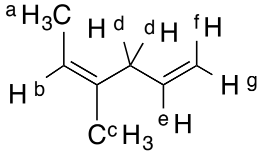

Hydrovinylation of Isoprene using [dppp]$CoCl_2$ (Table 3, Entry 22): To an oven dried round bottom flask with a sidearm, was added [dppp]$CoCl_2$ (50 mg, 0.092 mmol) dissolved in dichloromethane (1 mL) at room temperature. Trimethylaluminium as a 2M solution in toluene (0.14 mL, 0.28 mmol) was added dropwise and the color of the solution changed from deep blue to red brown with the formation of white fumes over the solution. After 2 to 3 minutes, the reaction vessel was carefully evacuated and then refilled with ethylene balloon. The reaction vessel was cooled to -20 ^{o}C and isoprene (63 mg, 0.92 mmol) added under ethylene and the mixture was stirred for 4 h. The ethylene balloon was removed and 0.1 mL methanol was added into the flask. The reaction solution was diluted with pentane (2 mL) and warmed to room temperature and subsequently passed through a silica plug very quickly. The plug was washed with pentane (3 x 5 mL). Removal of solvent was difficult due to high volatility of the product so GC and NMR analysis showed that the product was essentially pure (**16n**).

^{1}H NMR ($CDCl_3$, 400 MHz): 1.64 (d, J = 6.8 Hz, 3 H, H^a), 1.73 (m, 3 H, H^c), 2.83 (d, J = 6.4 Hz, 2 H, H^d ??), 5.02 (dm, J = 10 Hz, 1 H, H^g), 5.08 (dm, J = 17 Hz, 1 H, H^f), 5.35 (qm, J = 6.8 Hz, 1 H, H^b), 5.79 (ddt, J = 6, 10, 17 Hz, 1 H, H^e). ^{13}C NMR ($CDCl_3$, 125.02 MHz): 13.31, 23.50, 36.47, 115.09, 120.20, 134.04, 136.16.

Gas Chromatography: Methylsilicone SP GC (OT 35 ^{o}C/ isothermal, He carrier gas) R_T for product = 5.14 min. CSP GC (Cyclosil, OT 50 ^{o}C/ isothermal, H_2 carrier gas). R_T for product = 16.89 min.

Data File C:\CHEM32\1\DATA\YAM\YT-04-202A.D
Sample Name: YT-04-202A

===

Acq. Operator : YAM
Acq. Instrument : Babu   Location : Vial 201
Injection Date : 11/28/2012 10:33:22 AM
                                                        Inj Volume : 1 µl
Different Inj Volume from Sequence !   Actual Inj Volume : 3 µl
Acq. Method : C:\CHEM32\1\METHODS\YT_ISO120_CYCLODEX-B.M
Last changed : 11/28/2012 10:31:30 AM by YAM
(modified after loading)
Analysis Method : C:\CHEM32\1\METHODS\YT_ISO120_CYCLODEX-B.M
Last changed : 11/28/2012 11:27:32 AM by YAM
(modified after loading)

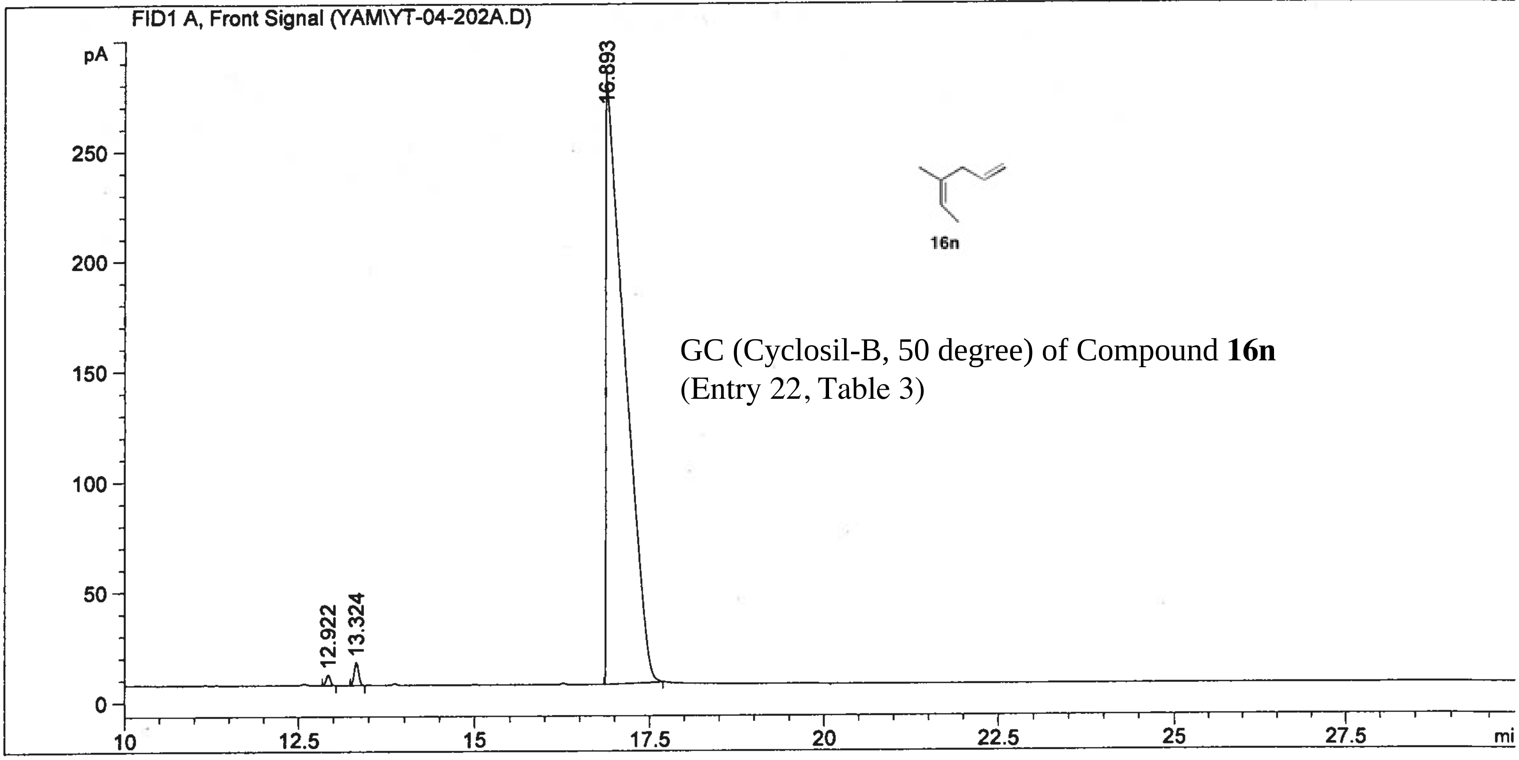

===

Area Percent Report

===

Sorted By : Signal
Multiplier: : 1.0000
Dilution: : 1.0000
Use Multiplier & Dilution Factor with ISTDs

Signal 1: FID1 A, Front Signal

Peak #	RetTime [min]	Type	Width [min]	Area [pA*s]	Height [pA]	Area %
1	12.922	BB	0.0647	20.02247	4.79711	0.38776
2	13.324	BB	0.0674	44.62691	10.29189	0.86425
3	16.893	BB	0.2242	5099.00146	277.23431	98.74799

Totals : 5163.65084 292.32332

Proton NMR (400 MHz, D-chloroform) spectra of compound 16n HV of isoprene using Cl2Co(dppp) {Entry 22, Table 3)

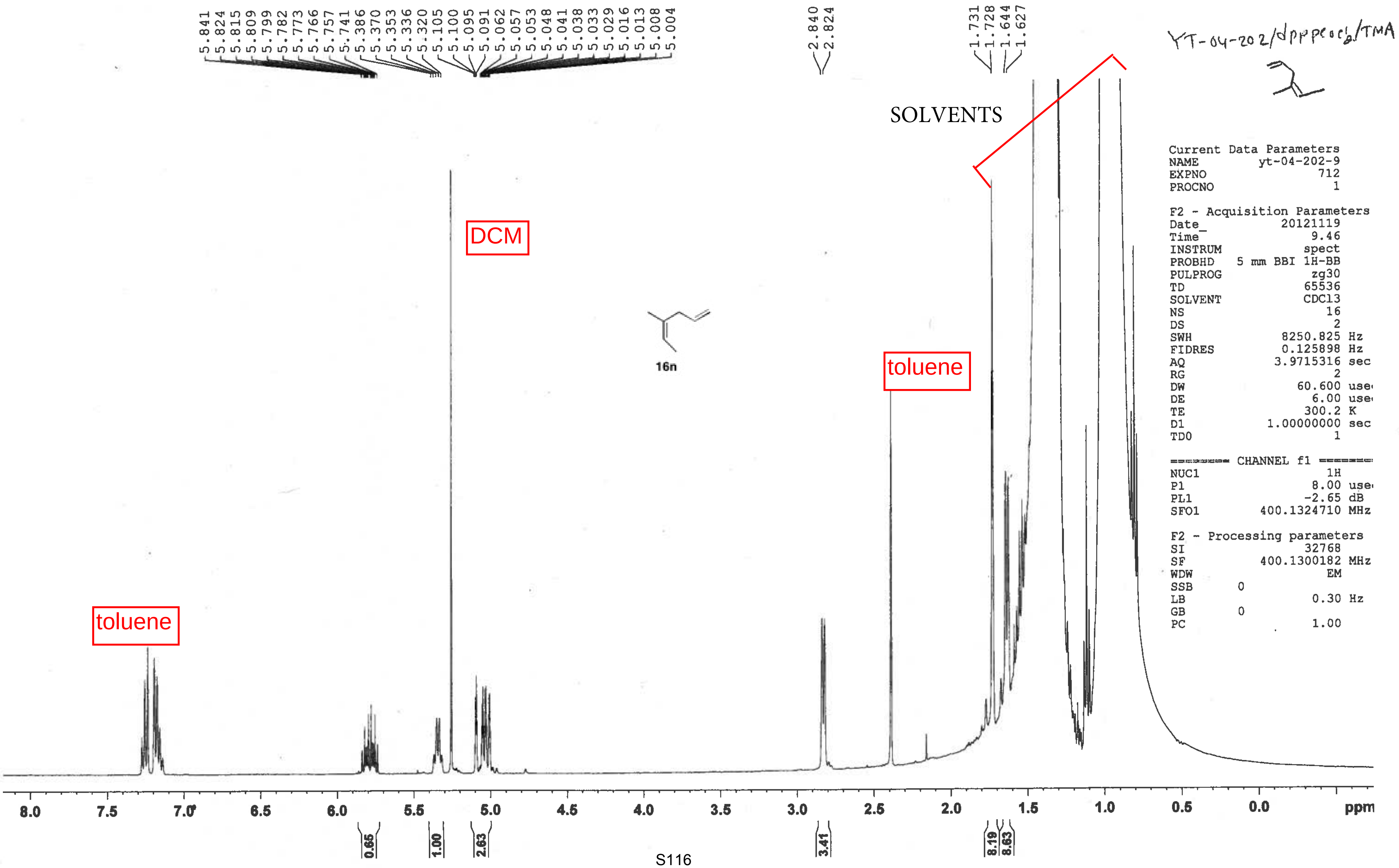

Proton NMR (400 MHz, D-Chloroform) Spectra of Compound 16n (Expansion of Alkene Region)

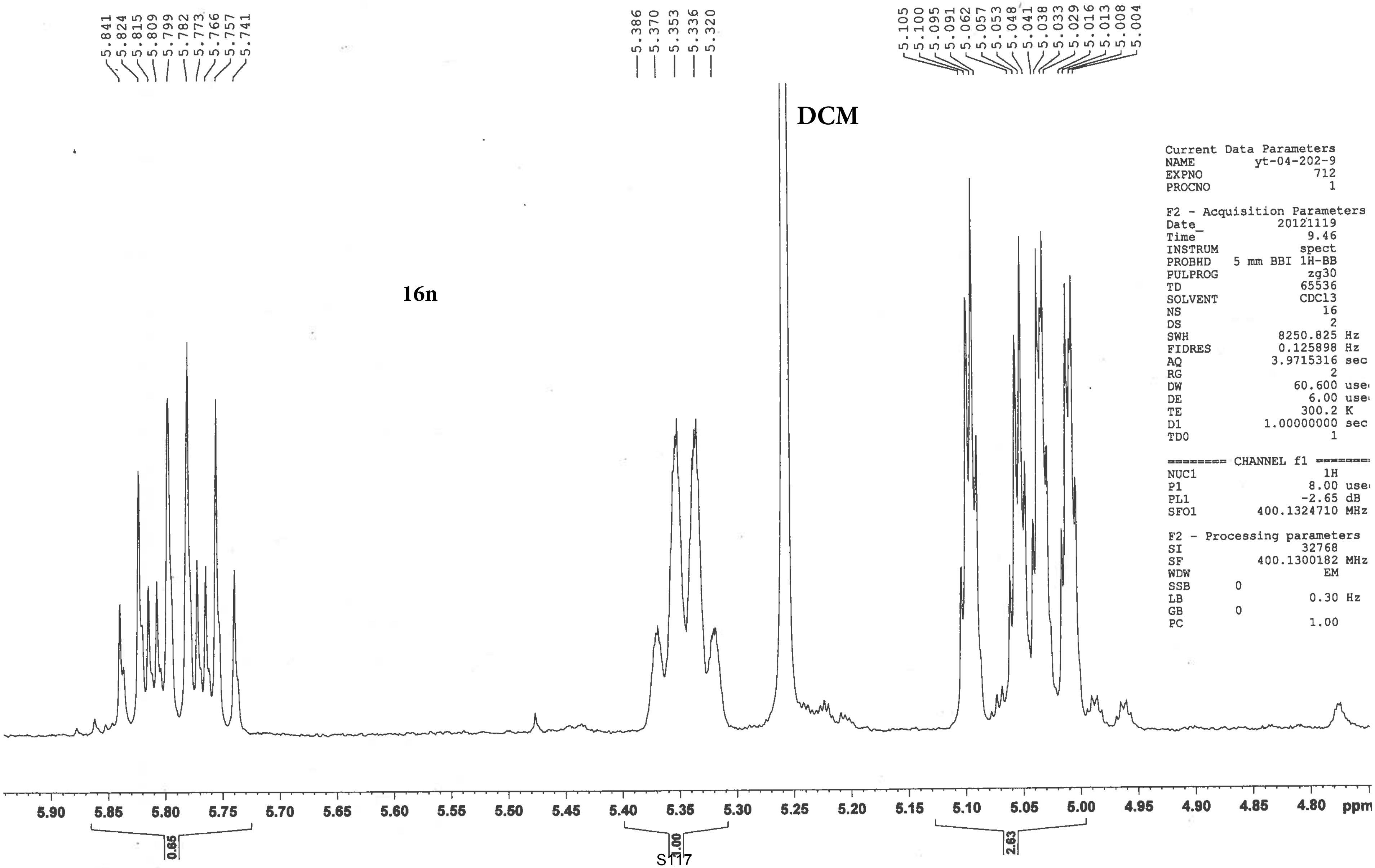

Carbon NMR (125 MHz, D-chloroform) spectra of **16n** Cl2Co(DPPP) complex with solvent toulene and pentane

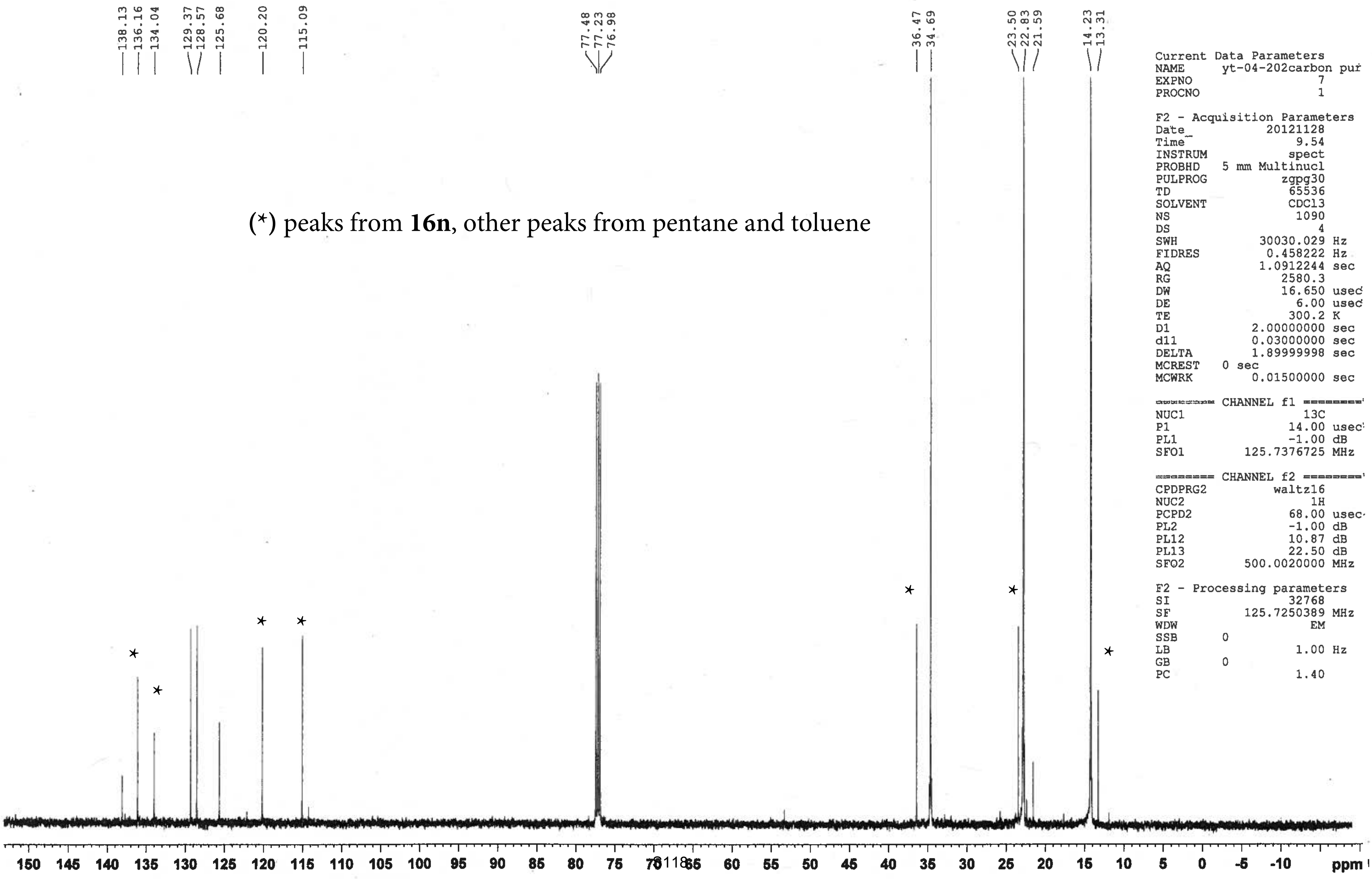

References

1. Grutters, M. M. P.; Mueller, C.; Vogt, D. *J. Am. Chem. Soc.* 2006, **128**, 7414.
2. Sharma, R. K.; RajanBabu, T. V., *J. Am. Chem. Soc.* 2010**, 132**, 3295.
3. (a) Reddy, S. S.; Radhakrishnan, K.; Sivaram, S. *Polymer Bulletin* 1996, **36**, 165-171. (b) Sangokya, S. A. Preparation of Methylaluminoxanes. U.S. Patent 5,248,801, Sep 28, 1993.
4. **13f:** Ehlers, J.; König, W. A.; Lutz, S.; Wenz, G.; tom Dieck, H. *Angew. Chem. Int. Ed. Engl.* 1988, **27**, 1556.
5. Blumberg, L. C.; Costa, B.; Goldstein, R. T*etrahedron Lett.* 2011, **52**, 872.
6. House, H. O.; Rasmusson, G. H. *J. Org.Chem.* 1961, **26**, 4278.
7. Piers, E.; Jung, G. L.; Ruediger, E. H. *Can. J. Chem.* 1987, **65**, 670.
8. Hoye, T. R.; Magee, A. S.; Trumper, W. S. *Synthetic Communications* 1982, **12**, 183.
9. Martin, S. F.; Tu, C.; Chou, T. *J. Am. Chem. Soc.* 1980, **102**, 5274.
10. Ikeda, Y.; Ukai, J.; Ikeda, N.; Yamamoto, H. *Tetrahedron* 1987, **43**, 723.
11. Wang, Y.; West, F. G. *Synthesis* 2002, 99.
